# Cell‐Type Specific Circuits in the Mammillary Body for Place and Object Recognition Memory

**DOI:** 10.1002/advs.202409397

**Published:** 2025-02-10

**Authors:** Lanfang Li, Yiqing Guo, Wei Jing, Xiaomei Tang, Jinyu Zeng, Zhenye Hou, Yige Song, Aodi He, Hao Li, Ling‐Qiang Zhu, Youming Lu, Xinyan Li

**Affiliations:** ^1^ Wuhan Center of Brain Science Huazhong University of Science and Technology Wuhan 430030 China; ^2^ Innovation Center of Brain Medical Sciences Ministry of Education of the People's Republic of China Wuhan 430030 China; ^3^ Department of Pathophysiology School of Basic Medicine and Tongji Medical College Huazhong University of Science and Technology Wuhan 430030 China; ^4^ Department of Anatomy School of Basic Medicine and Tongji Medical College Huazhong University of Science and Technology Wuhan 430030 China; ^5^ Department of Physiology School of Basic Medicine and Tongji Medical College Huazhong University of Science and Technology Wuhan 4030030 China

**Keywords:** hippocampus, mammillary body, memory

## Abstract

Mammillary body (MB) is traditionally viewed as a structural node of an anatomic circuit for emotion and memory. However, little is known about its molecular and cellular organizations. Here, a discovery that MB contains four subtypes of neurons that occupy different spatial subregions is reported. Of these, two subtypes of neurons are tagged by parvalbumin (PV) and dopamine receptor‐D2 (Drd2) markers. PV neurons are spontaneously active, whereas Drd2 neurons are inactive at rest and generate rebound bursts. These two distinct electrophysiological properties are encoded by *Kcnn4* and *Cacna1h*. PV and Drd2 neurons generate two distinct cell‐type specific circuits by receiving inputs from two discrete subiculum neuronal classes. Gain‐ and loss‐of‐function studies on these cortical‐subcortical circuits demonstrate their differential roles for place and object recognition memory. This finding provides a comprehensive molecular and structural atlas of MB neurons at single‐cell resolution and reveals that MB contains molecularly, structurally, and functionally dissociable streams within its serial architecture.

## Graphical abstract

Li et al. defined two novel glutamatergic neuronal subtypes in the medial mammillary nucleus, pars lateralis‐located parvalbumin (PV) neurons, and basalis‐located Drd2 neurons. PV and Drd2 neurons differ in their electrophysiological properties encoded by *Kcnn4* and *Cacna1h*, and selectively modulate place and object recognition memory by two distinct cell‐type specific subcircuits.

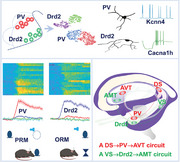

## 1 Introduction

The mammillary body (MB) on the posteroinferior aspect of the hypothalamus has been traditionally viewed as a structural node of an anatomic circuit for emotion^[^ ^1^ ^]^ and memory,^[^ ^2^ ^]^ involving a pathway from the hippocampus to the thalamus.^[^ ^2^, ^3^ ^]^ While this traditional view has largely relied on extrapolation from the lesion studies in rodents^[^ ^4^ ^]^ and the pathological observations in the patients of amnesic Korsakoff's syndrome.^[^ ^5^ ^]^ Little has been known about the molecular and cellular organizations of the mammillary nucleus subdivisions. How the individual neurons in MB are structurally and molecularly assembled to engage their behavioral function remain unknown.^[^ ^6^ ^]^

MB contains the medial (MM) and the lateral subdivisions (LM) and MM is larger than LM and can be further divided up to five smaller subdivisions, varying among the species.^[^ ^7^ ^]^ To date, a major experimental approach to the function of MB was to test the behavioral consequences of all subdivision lesions.^[^ ^6^ ^]^ Early studies in the 1970s indicated that lesions of whole MB caused hyperactivity in the open field,^[^ ^8^ ^]^ reduced the anxiety‐like behaviors in the elevated plus maze tests.^[^ ^9^ ^]^ Subsequent studies indicated that this lesion impaired the performance on tests of both reinforced and spontaneous T‐maze alternation.^[^ ^4^, ^10^ ^]^ The surgical lesions of all MB subdivisions were also reported to disrupt reference memory in Morris water maze tests, as well as on delayed matching‐to‐place in the water‐maze.^[^ ^4^, ^11^ ^]^ While different behavioral assays and lesion methods may contribute to the differences of the results, they may also result from traditional consideration of MB as a single brain structure. According to the previous research on electrophysiology and neurocircuitry tracing, the neurons in different subdivisions of MB exhibited distinct electrophysiological properties and consisted of different neurocircuits.^[^ ^6^, ^12^ ^]^ However, these results were not well verified and applied due to a lack of methods for precisely targeting and manipulating the subdivided neurons. Hence, the function controlled by each subdivision of MB is even less clear. A recent study using single cell transcriptomics identified multiple neuronal types in the ventral posterior hypothalamus including MB.^[^ ^13^ ^]^ Although one of these subtypes is found to be vulnerable to Alzheimer's disease, their electrophysiology, connectivity, and behavior are yet to be studied.

Here, we identified a total of 21 cell clusters in the MB using single‐cell RNA‐seq analysis. Of these, four subtypes of cells are characterized as neurons in MM; with their specific gene expression profiles, including *parvalbumin* (PV), *dopamine receptor‐d2* (Drd2), *Neurotensin* (NTS), and *nitric oxide synthase 1* (Nos1). Combined with genetically guided multimodal identification and fluorescence in situ hybridization (FISH), we found that these four subtypes of neurons are spatially segregated throughout the medial (MM) subdivisions of the MB. Subsequently, we focused our studies on two distinct subtypes of neurons that are specifically tagged by PV and Drd2 (hereafter named as PV and Drd2 neurons). PV neurons mainly express *Kcnn4* and *Snap25* genes, whereas Drd2 neurons specifically express *Cacna1h* and *Calbindin‐1* genes. Genetic labeling combined with electrophysiological recordings revealed that PV neurons are spontaneously active, whereas Drd2 neurons are inactive at rest and generate rebound bursts. A CRISPR‐Cas9 in vivo gene knockdown identified that *Kcnn4* and *Cacna1h* encode these two distinct electrophysiological properties of PV versus Drd2 neurons. Synaptic tracing studies demonstrated that PV and Drd2 neurons generate two distinct cell‐type specific subcircuits by receiving synaptic inputs directly from two discrete subiculum neuronal classes and routing their axon terminals onto the different anterior thalamic neuronal subtypes. Gain‐ and loss‐of function combined with image studies on these two subcircuits in vivo found their differential roles in place and object recognition memory. Together, this study provides a comprehensive molecular and structural architecture of PV and Drd2 neurons at single‐cell resolution and reports a discovery of two distinct cell‐type specific subcircuits to selectively modulate two distinct components of memory.

## 2 Results

### 2.1 Molecular and Anatomical Characterization of MB Cell Types

**Figure 1:**
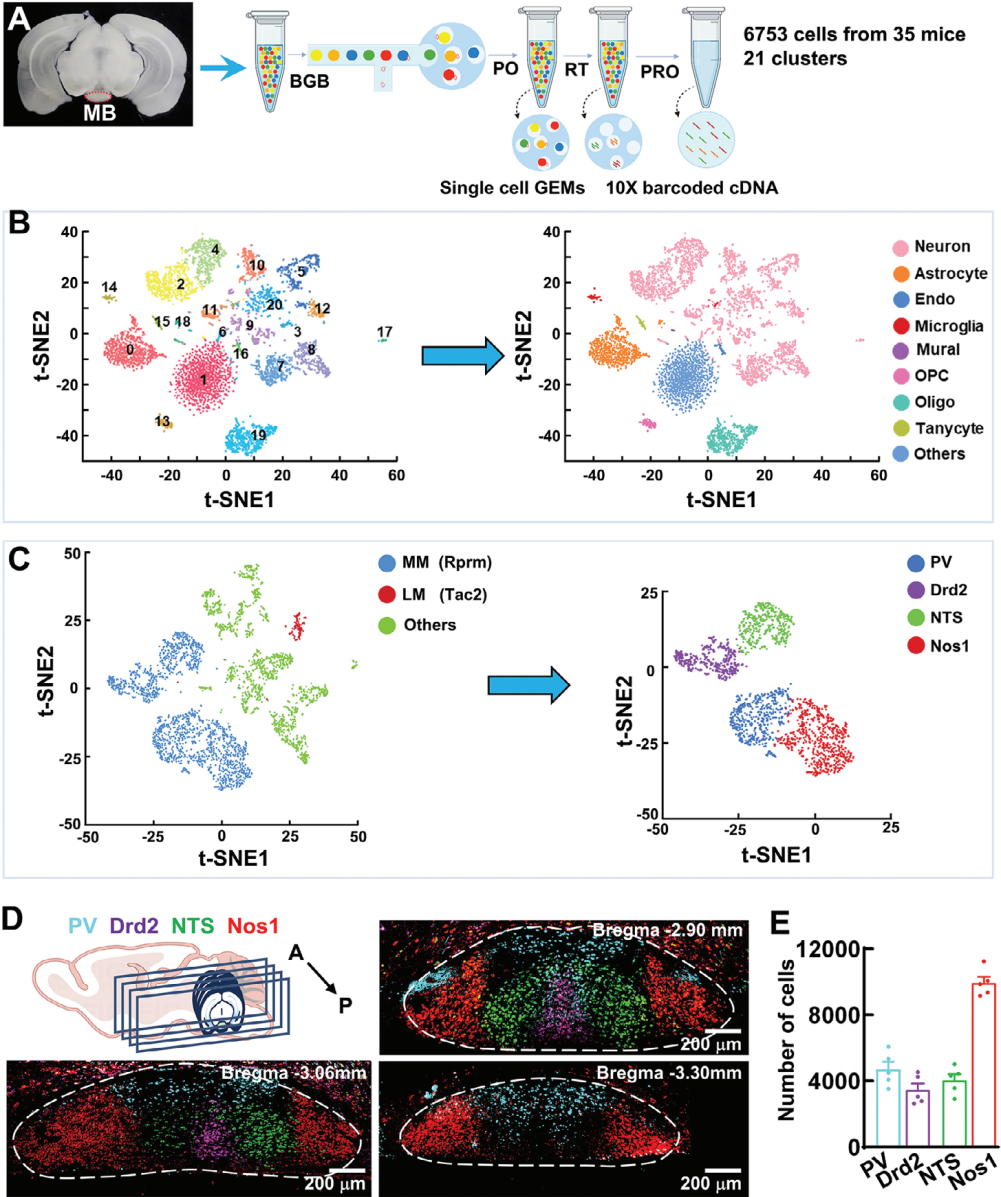
Molecular and anatomical classification of MB cell types. A)Workflow of single‐cell RNA‐seq from the MB. BGB: 10× barcoded gel beads, PO: GEMs in partitioning oil, RT: reverse transcription, PRO: pool remove oil. B) The *t*‐distributed stochastic neighbor embedding (t‐SNE) plots showing 21 (0–20) clusters of cell subtypes in the MB of C57BL/6 mice (left) and the clusters in different cell types or neurons (right). Endo: endothelial cells, OPC: oligodendrocyte precursor cells, Oligo: oligodendrocytes. C) The t‐SNE plots showing neuronal clusters in MM and LM, based on the expression of marker genes such as *Rprm* and *Tac2* (left) and four neuronal clusters in MM are defined with *PV*, *Drd2*, *NTS*, and *Nos1* (right). D) Four molecularly defined subtypes of neurons are spatially segregated in the MM. Illustration and representative images of FISH showing the brain sections from anterior (A) to posterior (P) staining with PV (blue), Drd2 (purple), NTS (green), and Nos1 (red). E) A bar graph showing the numbers of PV (4698 ± 451.6), Drd2 (3450 ± 393.5), NTS (4041 ± 364.4), and Nos1 (9932 ± 364.3) neurons. Data are mean ± SEM (*n* = 5 mice per group).

We first sought to assess the extent of heterogeneity within the MB region and identify potential marker genes for cell‐type specific access. To do this, we began by single‐cell RNA‐seq guided multimodal classification to examine gene expression differences in cells (a total of 6753 cells with an average of 2772 genes per cell were detected) from the mouse MB (*n* = 35 mice, **Figure** **1**A). We found a total of 21 molecularly distinct cell clusters, comprising all known major cell types (nine cell types) in the other cortical regions (Figure 1B; Figure S1A, Supporting Information). We identified several marker genes, which allowed alignment with known cell types and categorized 1893 neurons into PV, Drd2, NTS, and Nos1 neuronal subtypes (Figure 1C), as validated by fluorescence in situ hybridization (FISH), and more impressively, these four subtypes of neurons were spatially segregated in MM (Figure 1D,E; Figure S1B, Supporting Information).

### 2.2 Molecular and Anatomical Identification of Two Distinct MM Cell Types

**Figure 2:**
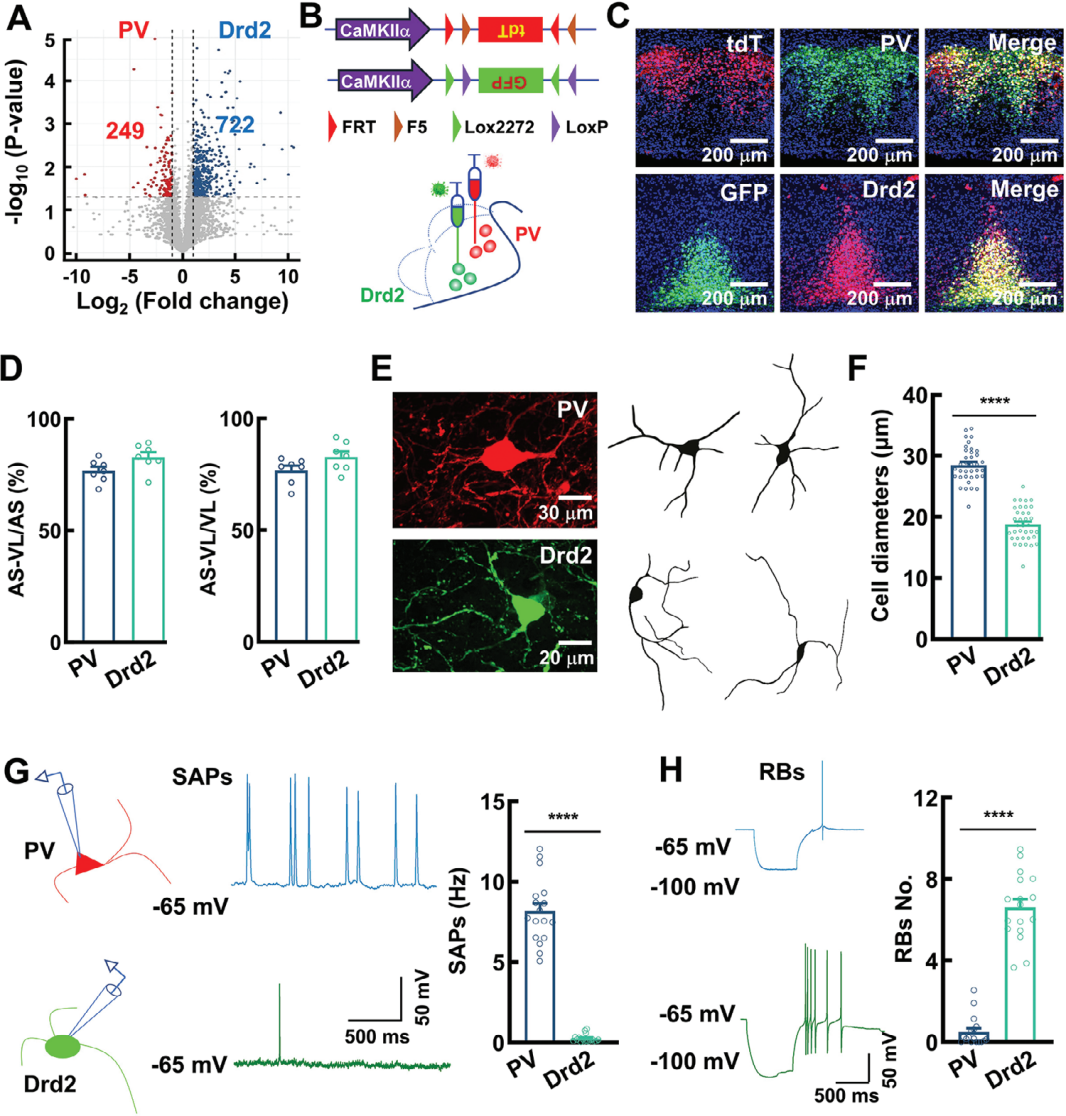
Distinct morphological and physiological properties of PV and Drd2 neurons. A) A volcano plot showing the genes enriched in PV (249 genes, red) versus Drd2 (722 genes, blue) neurons and both (gray) from the population cell RNA‐seq (*n* = 3 samples per group, three PV samples, respectively, contain 37, 45, and 39 neurons; three Drd2 samples, respectively, contain 48, 39, and 51 neurons, each sample from one individual mouse). B) A strategy for genetically labeling PV and Drd2 neurons in PV^FLP^ and Drd2^CRE^ mice in vivo. C) Representative images showing the virus labeled (VL) and antibody stained (AS) PV neurons in the pars lateralis and Drd2 neurons in the basalis. D) Bar graphs showing the percentage of AS‐VL neurons versus a total of AS neurons and the percentage of AS‐VL neurons versus a total of VL neurons. Data are mean ± SEM (*n* = 7 mice per group). E,F) Representative images and a bar graph showing a large pallidus pyramidal cell‐like morphology versus a small reticular granule cell‐like morphology reconstructed from PV versus Drd2 neurons. Data are mean ± SEM (*n* = 36 neurons per group, ****p < 0.0001, *t*‐test). G,H) Representative traces and bar graphs showing spontaneous action potentials (SAPs, G) and rebound bursts (RBs, H) intracellularly recorded from PV (blue) and Drd2 (green) neurons. Data are mean ± SEM (*n* = 17 neurons per group, ****p < 0.0001, *t*‐test).

Neurons in the lateral MB subdivision (LM) were reported to be vulnerable to Alzheimer's disease, whereas the physiological properties and behavioral function of neurons in the medial MB subdivision (MM) remain largely unknown. Distributions of these molecularly defined neuronal subtypes across the MM were spatially divisible; both NTS and Nos1 neurons were divided into two bilaterally symmetrical subgroups in the MM. PV neurons were mainly located in the dorsal pars lateralis, whereas Drd2 neurons were concentrated in the ventral basalis (Figure 1D; Figure S1B, Supporting Information). Here, we focused our studies on PV and Drd2 neurons because these two types of neurons can be dissected and specifically targeted by our established genetic strategies, in which PV^FLP^ mice, in which FLP was expressed under control of PV promoter and Drd2^CRE^ mice, in which CRE was expressed under control of Drd2 promoter, were used. To lucubrate the genetic differences, we isolated PV and Drd2 neurons from brain and analyzed their gene expression patterns using population cell RNA‐seq (a total of 259 cells with an average of 12522 genes per neuronal type were detected, **Figure** **2**A; Figure S2A,B, Table S1 and Table S2, Supporting Information). Both PV and Drd2 neurons expressed *5‐hydroxytryptamine receptor 2a* (*Htr2a*). PV has been widely used as a molecular maker for a subset of γ‐aminobutyric acid (GABA) inhibitory interneurons.^[^ ^14^ ^]^ Here, we found that nearly all PV and Drd2 neurons were expressed with excitatory glutamatergic neuronal markers, such as Ca^2+^/calmodulin‐dependent kinase‐IIα (*CaMK‐IIα*) and vesicular glutamate trabsporter‐2 (*vGluT2*), but not with GABAergic inhibitory neuronal markers such as *GAD1/2* and vesicular GABA transporter (*vGAT*) (Figure S2A,C, Supporting Information). Hence, we classified PV neurons in the MM as a subtype of excitatory glutamatergic neurons. The genes expressed in the two subtypes of glutamatergic neurons were largely overlapped with these expressed in excitatory pyramidal neurons in the hippocampus, such as *Thy1*, *CCK*, and *Epha7* and granule neurons in the dentate gyrus, such as *Rph3a*, *Calb1*, and *Rprm*.^[^ ^15^ ^]^ Many of these genes encode ion channels and neurotransmitter receptors and have been shown to play functional roles in synaptic transmission and plasticity.^[^ ^14a^ ^]^ Some were novel, such as *Pacc1* (proton activated chloride channel 1) that involves in chloride transport and *Chrm3* (M‐type acetylcholine receptor) that affects many of the roles of acetylcholine in the central and peripheral nervous system.^[^ ^16^ ^]^

### 2.3 Distinct Morphological and Physiological Properties

To analyze the morphological and physiological properties of PV versus Drd2 neurons and visualize these neuronal types in adult mice in vivo, we used the transgenic lines, in which FLP and CRE recombinases were expressed in PV (PV^FLP^) and Drd2 (Drd2^CRE^) neurons under the control of PV and Drd2 promoters, respectively (Figure 2B). Targeting strategies and corresponding genotypic assays for generation of PV^FLP^ and Drd2^CRE^ mice were described in the methods. We applied the rAAV2/9‐CaMK‐IIα‐fDIO‐tdT or the rAAV2/9‐CaMK‐IIα‐DIO‐GFP infectious virus particles into MM of PV^FLP^ or Drd2^CRE^ mice, resulting in the expression of tdT in PV neurons (PV^tdT^) and GFP in Drd2 neurons (Drd2^GFP^, Figure 2C; Figure S2D, Supporting Information). After the virus injection, we stained the sections with anti‐bodies against PV and Drd2. In PV^FLP^ mice, 76.7% tdT‐expressing neurons were stained with anti‐PV, 3.1% tdT‐expressing neurons were stained with anti‐Drd2. In Drd2^CRE^ mice, 82.9% GFP‐expressing neurons were labeled with anti‐Drd2, while 1.3% GFP‐expressing neurons were labeled with anti‐PV. These data revealed the specificity of FLP or CRE‐dependent virus infection (Figure 2D; Figure S2D–F, Supporting Information).

We next determined the morphological and electrophysiological properties of PV and Drd2 neurons by somatic intracellular and whole‐cell patch‐clamp recordings in the slices in vitro. We recorded a total of 129 neurons (68 PV neurons versus 61 Drd2 neurons) in the MM from PV^FLP^ and Drd2^CRE^ mice (*n* = 18 mice at 90 ± 2 days old of age) and found that PV neurons displayed large pallidus pyramidal cell‐like morphologies, with somatic diameter ranging from 26 to 35 µm (28.51 ± 0.49 µm, Figure 2E,F). Dendrites were densely spinous and ramified mostly in the pars lateralis. Drd2 neurons revealed small reticular granule cell‐like morphologies, with cell body diameter ranging from 15 to 23 µm (18.77 ± 0.48 µm, Figure 2E,F). PV neurons were spontaneously active and fired spontaneous action potentials at a range of theta rhythms (8.17 ± 0.49 Hz in PV neurons vs 0.27 ± 0.06 Hz in Drd2 neurons, Figure 2G; Table S3, Supporting Information), whereas Drd2 neurons were largely silent at rest and generated rebound bursts (RBs); the numbers of RBs were 6.62 ± 0.40 in Drd2 neurons vs 0.50 ± 0.18 RBs in PV neurons (Figure 2H; Table S3, Supporting Information). Drd2 neurons fired high‐frequency repetitive spikes in response to depolarizing currents; the maximal frequencies of the firings were 183.08 ± 7.86 Hz in Drd2 neurons vs 105.19 ± 12.26 Hz in PV neurons (Figure S3A‐C; Table S3, Supporting Information). These results indicate that although both PV and Drd2 neurons expressed excitatory glutamatergic neuronal markers, their electrophysiological features shared the similarity with those seen in classical inhibitory GABAergic interneurons in the other brain regions.^[^ ^17^ ^]^

### 2.4 Electrophysiological Properties of *Kcnn4* and *Cacna1h* in PV and Drd2 Neurons

**Figure 3:**
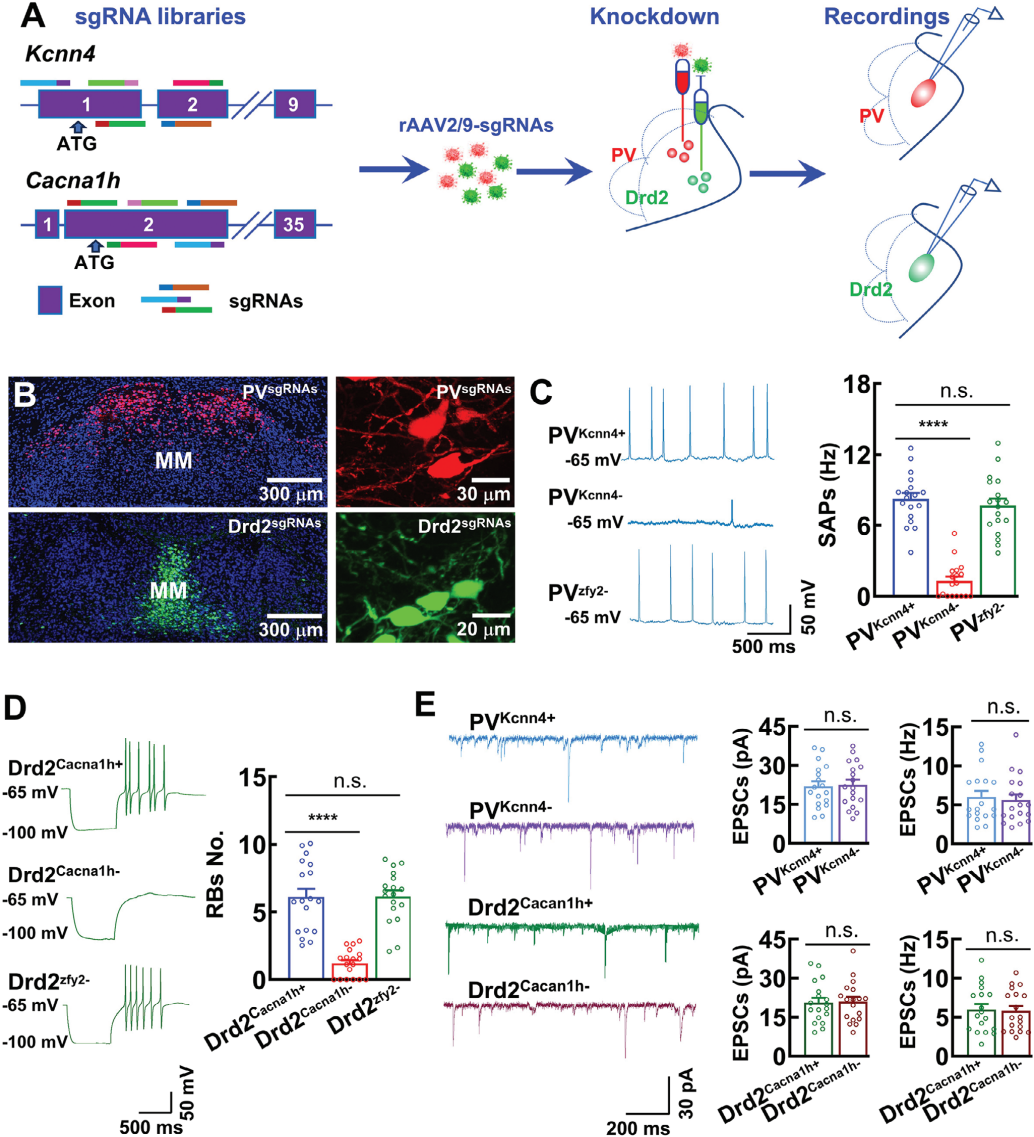
Electrophysiological properties of *Kcnn4* and *Cacna1h* in PV and Drd2 neurons. A) A strategy for rAAV2/9‐mediated CRISPR‐Cas9 in vivo gene knockdown. B) Representative images showing the expression of *Kcnn4* and *Cacna1h* sgRNAs in PV (red) and Drd2 (Green) neurons. C) Representative traces and a bar graph showing SAPs in PV neurons with (PV^Kcnn4−^) or without (PV^Kcnn4+^) *Kcnn4* knockdown, sgRNAs target to zfy2 as the negative control (PV^zfy2−^). Data are mean ± SEM (*n* = 18 neurons per group, adjusted ****p < 0.0001, one‐way ANOVA followed with Bonferroni's post hoc test). D) Representative traces and a bar graph showing RBs in Drd2 neurons with (Drd2^Cacan1h−^) and without (Drd2^Cacan1h+^) *Cacan1h* knockdown, sgRNAs target to zfy2 as the negative control (Drd2^zfy2−^). Data are mean ± SEM (*n* = 18 neurons per group, adjusted **** P < 0.0001, one‐way ANOVA followed by Bonferroni's post hoc test). E) Representative traces and bar graphs showing spontaneous miniature EPSCs in PV and Drd2 neurons with (PV^Kcnn4−^ and Drd2^Cacna1h−^) or without (PV^Kcnn4+^ and Drd2^Cacna1h+^) *Kcnn4* or *Cacna1h* knockdown. Data are mean ± SEM (*n* = 18 neurons per group, *t*‐test).

In our present study, we have shown that PV and Drd2 neurons notably differed by their electrophysiological properties and gene expression patterns; PV neurons are spontaneously active, whereas Drd2 neurons are inactive at rest and generate rebound bursts. We then examined whether these differentially expressed genes in PV versus Drd2 neurons could covary with their specific electrophysiological properties. As shown in Figure 2A, over hundreds of genes were differentially expressed in PV and Drd2 neurons. Of these, *Kcnn4* is known to encode a hyperpolarization‐activated and cyclic‐AMP regulated type‐3 cation (HCN3) channel and is responsible for spontaneous action potential firings,^[^ ^18^ ^]^ whereas *Cacna1h* encodes a pore‐forming α subunit of low voltage‐gated T‐type Ca^2+^ channel for bursting firings.^[^ ^19^ ^]^ We then examined whether expression of *Kcnn4* determines spontaneous action potential firings from PV neurons and expression of *Cacna1h* contributes to rebound bursts in Drd2 neurons. We developed a rAAV2/9 virus‐mediated CRISPR‐Cas9 in vivo knockdown by targeting *Kcnn4* and *Cacna1h* genes in PV^FLP^ or Drd2^CRE^ mice (**Figure** **3**A). We injected the rAAV2/9‐CaMK‐IIα‐fDIO‐SpCas9 and rAAV2/9‐CaMK‐IIα‐fDIO‐tdT or the rAAV2/9‐CaMK‐IIα‐DIO‐SpCas9 and rAAV2/9‐CaMK‐IIα‐DIO‐GFP, mixed with the rAAV2/9‐U6‐sgRNA‐*Kcnn4* or *Cacna1h* virus particles into the MM of PV^FLP^ or Drd2^CRE^ mice (Figure 3B). rAAV2/9‐U6‐sgRNA‐*zfy2* was used as negative control because *zfy2* was not expressed in neurons of rodent brain. Then, we applied population cell qPCR analysis and revealed the efficient knockdown of *Kcnn4* and *Cacna1h* genes (Figure S4, Supporting Information). We found that knockdown of *Kcnn4* (PV^Kcnn4−^ mice) completely eliminated spontaneous action potential (SAP) firings (8.24 ± 0.51 Hz in PV^Kcnn4+^ neurons vs 1.30 ± 0.36 Hz in PV^Kcnn4−^ neurons, Figure 3C), whereas knockdown of *Cacna1h* gene (Drd2^Cacna1h−^ mice) abolished RBs that were normally occurred in Drd2 neurons (6.11 ± 0.60 in Drd2^Cacna1h+^ neurons vs 1.20 ± 0.24 in Drd2^Cacna1h−^ neurons, Figure 3D). Neither knockdown of *Kcnn4* or *Cacna1h* in PV or Drd2 neurons affected the frequencies and the mean amplitudes of the spontaneous miniature excitatory synaptic currents (EPSCs), which reflect postsynaptic responses of PV or Drd2 neurons to glutamate transmitter released from their respective presynaptic neurons (Figure 3E).

### 2.5 Distinct Roles of PV and Drd2 Neurons in Place and Object Recognition Memory

Observed electrophysiological properties and their molecular mechanisms in PV versus Drd2 neurons motivated us to examine the behavioral phenotypes that might be associated with these two distinct neuronal subtypes. Through lesion studies, the mammillary nucleus has previously been shown to play roles in spatial working memory.^[^ ^4^, ^20^ ^]^ A complete ablation of the mammillary subdivisions reduced the anxiety‐like behaviors in the elevated plus maze tests^[^ ^9^ ^]^ and caused hyperactivity in the open field.^[^ ^8^ ^]^ However, all these previous lesion studies were limited by lacking both the temporally acute silencing and neuronal subclass specificity.

**Figure 4:**
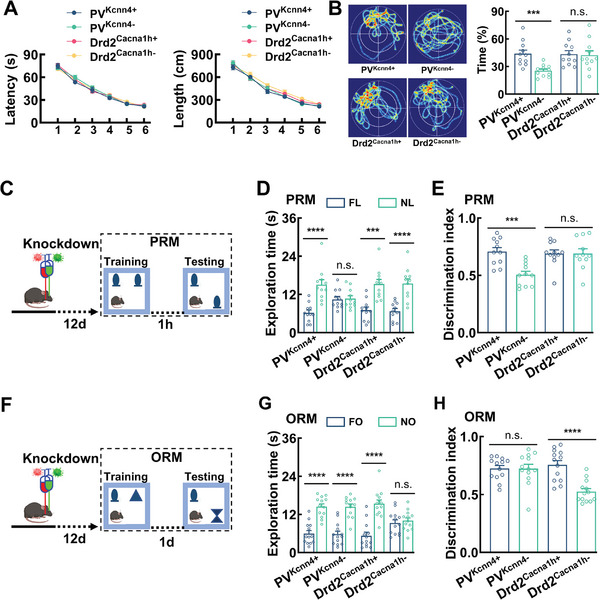
Distinct roles of PV and Drd2 neurons in place and object recognition memory. A) The latency and the length of swim path to reach a hidden platform and B) the percentage of time spent in searching of a hidden platform in targeting quadrant (quadrant 2) during the probe trial of PV^Kcnn4+^, PV^Kcnn4−^, Drd2^Cacna1h+^, and Drd2^Cacna1h−^ mice. Data are mean ± SEM (*n* = 11 mice per group, repeated measures two‐way ANOVA, followed with Bonferroni's post hoc test, ***P < 0.001, *t*‐test). C–E) Knockdown of *Kcnn4* in PV neurons impairs PRM. Experimental procedures (C) and bar graphs showing total exploration time (D) with the familiar (blue bars) versus the novel place (green bars) and the discrimination index (E) after knockdown of *Kcnn4* in PV neurons (PV^Kcnn4−^mice) or *Cacna1h* in Drd2 neurons (Drd2^Cacna1h−^ mice). Data are mean ± SEM (*n* = 11 mice per group, ***P < 0.001, ****p < 0.0001, *t*‐test). F–H) Knockdown of *Cacna1h* in Drd2 neurons impairs ORM. Experimental procedures (F) and bar graphs showing total exploration time (G) with the familiar (blue bars) versus novel object (green bars) and the discrimination index (H) after knockdown of *Cacna1h* in Drd2 neurons (Drd2^Cacna1h−^mice) or *Kcnn4* in PV neurons (PV^Kcnn4−^ mice). Data are mean ± SEM (*n* = 13 mice per group, ****P < 0.0001, *t*‐test).

Accordingly, we used a large battery of the behavioral screening tests to determine the behavioral phenotypes of PV^Kcnn4‐^ and Drd2^Cacna1h−^ mice. We found that all mice including PV^Kcnn4−^ and Drd2^Cacna1h−^ mice and their respective control littermates (PV^Kcnn4+^ vs Drd2^Cacna1h+^ mice) performed normally in their home cage, open field and elevated plus maze tests (Figure S5A–D, Supporting Information). Next, we tested these mice in a water maze task,^[^ ^21^ ^]^ with four trials per day as previously described.^[^ ^22^ ^]^ No significant difference in path length and latency was observed among groups (**Figure** **4**A), indicating that neither deletion of *Kcnn4* in PV neurons nor deletion of *Cacna1h* in Drd2 neurons affect spatial learning. When tested during probe trials performed on the next day after the training session, PV^Kcnn4−^ mice spend significant less amount of time in the quadrant that harbored the platform, and crossed it with significant less amount of time in this virtual location, as compared with the other three groups (the percentage of time 43.94 ± 4.00% in PV^Kcnn4+^ mice vs 25.46 ± 1.91% in PV^Kcnn4−^ mice, Figure 4B; Figure S5E,F, Supporting Information). These data indicate that spontaneous activity of PV neurons affects spatial memory.

To further examine spatial memory, we tested the behavioral performance of the mice in spontaneous recognition memory (SRM) tasks that were developed on animals' natural preference for novelty (Figure 4C). The tasks involved the spontaneous exploration of object sets in a chamber, with two different categories of novelty (place versus object) introduced in each task; place (PRM) versus object (ORM) recognition memory.^[^ ^23^ ^]^ The tasks consisted of a 10‐min training or initial exposure session, followed by a 5‐min testing or sample session, in which novelty (place versus object) was introduced. We tested different delays between the training and testing sessions and found that in the PRM task, at a delay of more than 2 h, most mice were unable to discriminate between the familiar and novel places. In the ORM task, at a delay of less than 18 h, all mice were capable of discriminating between the familiar and novel objects. To effectively probe the deficits and enhancement of memories, we used 1 h delay between the training and testing sessions for PRM task and 24 h delay for ORM task (Figure S6A,B, Supporting Information).

In the training session, total exploration time was comparable among groups and the mice of each group showed no difference in their explorations with each of the two objects in both PRM and ORM tasks (Figure S6C,D, Supporting Information). In the PRM task, we moved one of the familiar objects to a novel spatial place one hour after the training session (Figure 4C). Our data revealed that PV^Kcnn4−^ mice explored both the familiar and the novel places equally, whereas all the other groups, including PV^Kcnn4+^, Drd2^Cacna1h−^, and Drd2^Cacna1h+^ mice, spent more time exploring the object in the novel place (6.24 ± 0.87 s with the familiar place vs 15.08 ± 1.56 s with the novel place in PV^Kcnn4+^ mice; 10.45 ± 0.90 s with the familiar place and 10.68 ± 0.91 s with the novel place in PV^Kcnn4−^ mice, Figure 4D). To explore whether mice were individually expressing a preference for the novel over the familiar place, we analyzed a discrimination index (DI) by dividing the amount of time spent exploring the novel place by the total time spent exploring both the familiar and the novel places (Figure 4E). This analysis allowed for controlling the individual differences in exploration. We found that the discrimination index in PV^Kcnn4−^ mice was close to a chance level (0.51 ± 0.03, Figure 4E), with evidence of the discrimination emerging in all the other groups (0.71 ± 0.03 in PV^Kcnn4+^, Figure 4E). Importantly, when we conducted the task with a short delay (5 min between the training and testing sessions, Figure S6E, Supporting Information), all mice including PV^Kcnn4−^ mice displayed their preference for a novel place (Figure S6F,G, Supporting Information). This behavioral deficit was consistent with it seen in the Morris water maze tests (Figure 4B), showing an essential role of PV neurons in spatial memory.

In the ORM task, we presented the mice with a novel object in the same spatial place on the next day after the training session (Figure 4F). Drd2^Cacna1h−^ mice explored both the familiar and the novel objects equally (9.29 ± 1.00 s vs 10.03 ± 0.86 s, Figure 4G), while all the other groups including Drd2^Cacna1h+^, PV^Kcnn4−^, and PV^Kcnn4+^ mice, spent significantly more time exploring the novel object. Comparison of discrimination indexes revealed that Drd2^Cacna1h−^ mice did not differentiate the familiar from the novel object (0.76 ± 0.04 in Drd2^Cacna1h+^ mice vs 0.52 ± 0.03 in Drd2^Cacna1h−^ mice; Figure 4H), whereas all the other groups showed their ability to identify between the objects (Figure 4H). Notably, when we conducted the task with a minimum delay (10 min between the training and the testing sessions, Figure S6E, Supporting Information), Drd2^Cacna1h−^ mice displayed their preference for the novel object (Figure S6F,G, Supporting Information), indicating that the performance in the task was driven by the ability of object recognition memory, but not by changing sensory or motor abilities necessary to complete the task.

### 2.6 Two Distinct Cell‐Type Specific Subcircuits

**Figure 5:**
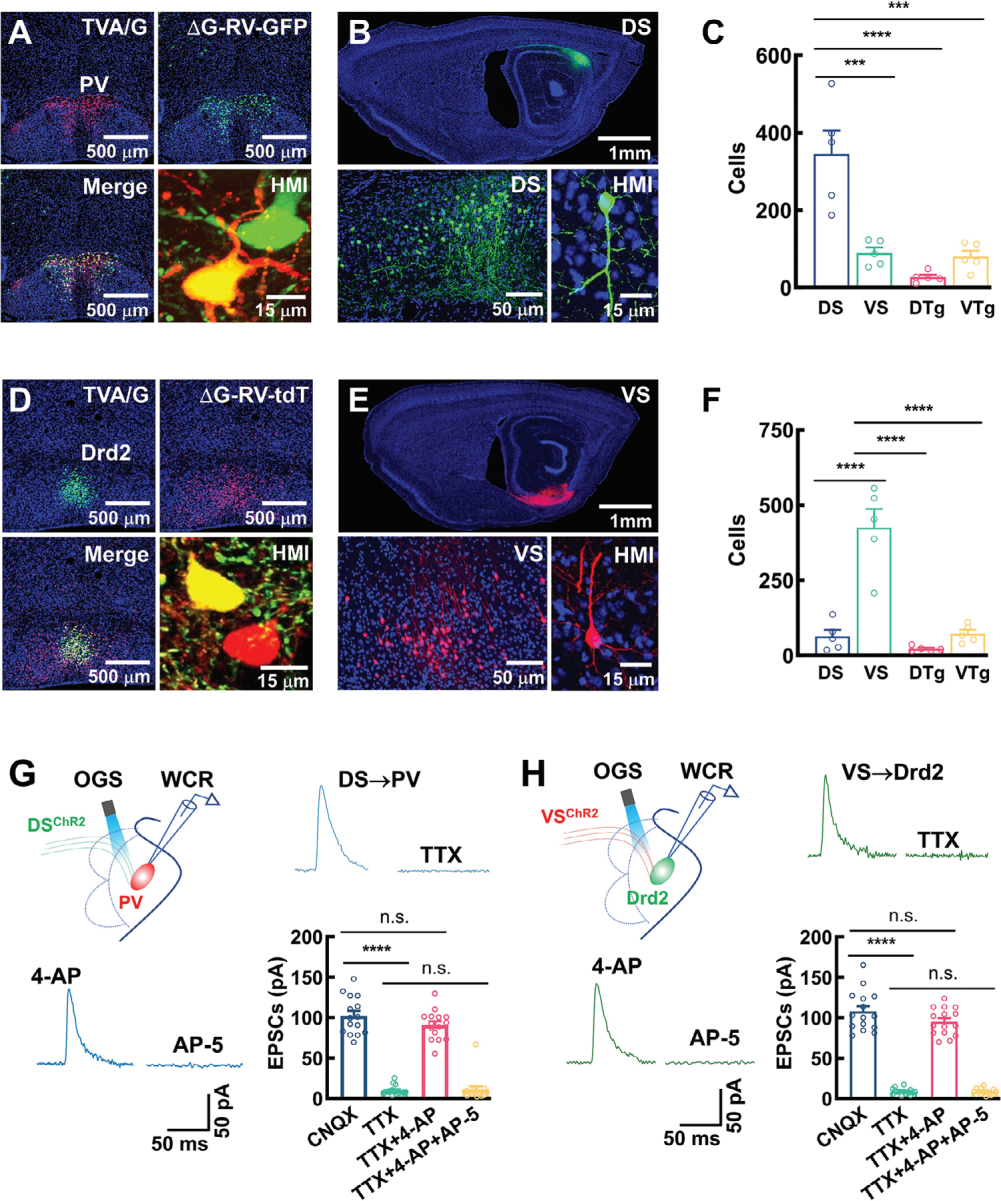
Structurally and functionally distinct cell‐type specific subcircuits. A–C) Representative images (A and B) showing GFP (green) in PV^TVA/G^ neurons (A) and their presynaptic pyramidal neurons (B) in the dorsal subiculum (DS). A bar graph (C) showing the numbers of GFP‐expressing neurons in each of these brain regions. Data are mean ± SEM (*n* = 5 mice per group, adjusted ***P < 0.001, ****P < 0.0001, one‐way ANOVA followed with Bonferroni's post hoc test). HMI: high‐magnification image showing single DS neuron. D–F) Representative images (D and E) showing tdT (red) in Drd2^TVA/G^ neurons (D) and their presynaptic pyramidal neurons (E) in the ventral subiculum (VS). A bar graph (F) showing the numbers of tdT‐expressing neurons in each of these brain regions. Data are mean ± SEM (*n* = 5 mice per group, adjusted ****P < 0.0001, one‐way ANOVA followed with Bonferroni's post hoc test). HMI: high‐magnification image showing single VS neuron. G,H) Experimental designs and whole‐cell patch‐clamp recordings from PV (G) and Drd2 (H) neurons. Representative traces of NMDA receptor‐mediated postsynaptic currents (EPSCs) at a holding potential of +60 mV were evoked by stimulating DS^ChR2^ (DS→PV, blue) or VS^ChR2^ (VS→Drd2, green) terminals in the presence of 20 × 10^−6^ m CNQX. EPSCs were blocked by 1 × 10^−6^ m TTX and rescued by 100 × 10^−6^ m 4‐AP and were sensitive to 50 × 10^−6^ m AP‐5. OGS: Optogenetic stimulation. Bar graphs showing the mean amplitudes of EPSCs. Data are mean ± SEM (*n* = 15 neurons per group, adjusted ****P < 0.0001, one‐way ANOVA followed with Bonferroni's post hoc test).

We next asked whether task‐specific information with two distinct elements—for example, PRM and ORM—is differentially driven by synaptic inputs onto PV versus Drd2 neurons. Although the mammillary nucleus has a very longstanding association with memory,^[^ ^24^ ^]^ remarkably little is known about the organization principles that underlie its behavioral selection. Functional models of the mammillary nucleus have inevitably focused on their dense hippocampal inputs,^[^ ^3b^ ^]^ so that the mammillary nuclei are principally thought to relay hippocampus to the thalamus via the mammillothalamic tract.^[^ ^2^ ^]^ However, this notion has relied mainly on extrapolation from the nonspecific lesion studies in rodents. Whether there is a direct cell‐type specific connection between the mammillary nuclei and the hippocampal formation has remained elusive. Notably, a cell‐type specific circuit in the mammillary nucleus is difficult to study owing to the small size and unknown cell types of mammillary nucleus. We then created the transgenic lines with the expression of avian viral receptor TVA and rabies G proteins in PV (PV^TVA/G^ mice) and Drd2 (Drd2^TVA/G^ mice) neurons by injecting the rAAV2/9‐CaMK‐IIα‐fDIO‐TVA/G‐tdT or the rAAV2/9‐CaMK‐IIα‐DIO‐TVA/G‐GFP virus particles into the MM of PV^FLP^ or Drd2^CRE^ mice. Synaptic retrograde ΔG‐rabies viruses encoding GFP (ΔG‐RV‐GFP) or tdT (ΔG‐RV‐tdT) were then injected into the same region (Figure S7, Supporting Information). This injection caused the labeling of GFP or tdT in PV^TVA/G‐tdT^ versus Drd2^TVA/G‐GFP^ neurons and their respective presynaptic neurons in several brain regions, including the dorsal (dSub) and the ventral (vSub) subiculum and the Gudden's dorsal (DTg) and ventral (VTg) tegmental nuclei (**Figure** **5**A–F; Figure S7, Supporting Information). Specifically, we found that PV neurons received synaptic inputs mainly from pyramidal neurons that were located in the dSub (hereafter named as DS neurons, Figure 5B,C), whereas Drd2 neurons were innervated by pyramidal neurons that were distributed in the vSub (hereafter named as VS neurons, Figure 5E,F). The subiculum is widely considered as the primary output subfield of the hippocampal formation and sends its projections mainly onto the medial entorhinal cortex.^[^ ^25^ ^]^ Here, we discovered that PV and Drd2 neurons form two distinct subcircuits (DS→PV vs VS→Drd2) by receiving long‐range synaptic inputs directly from two distinct, spatially‐divided subiculum neuronal classes (DS vs VS neurons). This finding provides two cell‐type specific circuits that directly links the subiculum to the mammillary nucleus, likely for selectively processing different components of mnemonic information.

To determine whether DS→PV and VS→Drd2 connections are functional, we engineered DS and VS neurons with the expression of channelrhodopsin‐2‐H134R, a modified version of a light‐gated ion channel (ChR2). First, we injected ΔG‐RV‐DRE virus particles into the MM of PV^TVA/G^ and Drd2^TVA/G^ mice, resulting in the expression of DRE in DS or VS neurons. The rAAV2/9‐CaMK‐IIα‐dDIO‐ChR2‐GFP or rAAV2/9‐CaMK‐IIα‐dDIO‐ChR2‐tdT virus particles were then injected into the dSub or the vSub, resulting in the expression of ChR2 in DS (DS^ChR2^ mice) versus VS (VS^ChR2^ mice) neurons.

Next, we carried out whole‐cell patch‐clamp recordings from either PV or Drd2 neurons in the DS^ChR2^ or VS^ChR2^ mice (Figure 5G,H). To eliminate polysynaptic responses, we recorded NMDA receptor‐mediated EPSCs at a holding potential of +60 mV in the presence of 20 × 10^−6^ m of the AMPA‐receptor antagonist 6‐cyano‐7‐nitroquinoxaline‐2,3‐dione (CNQX). EPSCs were evoked by illumination of DS^ChR2^ or VS^ChR2^ afferent axon fibers. Evoked EPSCs were sensitive to TTX and NMDA‐receptor antagonist (2 *R*)‐amino‐5‐phosphonopentanoate (AP‐5) and reversed by 4‐AP. These data demonstrate that PV and Drd2 neurons form functional excitatory synaptic connections with DS and VS neurons, respectively.

Parallelly, we identified downstream synaptic targets of PV and Drd2 neurons using a genetically modified virus tracing strategy. First, we applied the rAAV2/9‐CaMK‐IIα‐fDIO‐TK‐tdT or the rAAV2/9‐CaMK‐IIα‐DIO‐TK‐GFP virus particles into the MM of PV^FLP^ or Drd2^CRE^ mice, resulting in the expression of thymidine kinase (TK) in PV (PV^TK^) versus Drd2 (Drd2^TK^) neurons (Figure S8, Supporting Information). Next, we injected a genetically modified version of Herpes simplex virus type 1 strain 129 (H129ΔTK‐GFP, or H129ΔTK‐tdT), in which TK was deleted. Consistent with the previous reports,^[^ ^26^ ^]^ this injection caused the expression of GFP or tdT in PV ^TK^ (PV^tdT‐GFP^) or Drd2^TK^ (Drd2^GFP‐tdT^) neurons, as well as in their postsynaptic neurons in the anterior thalamic nuclei (ATN) including anteroventral and the anteromedial thalamus (hereafter named as AVT and AMT neurons, respectively, Figure S8, Supporting Information). This finding is consistent with a recent work,^[^ ^13^ ^]^ showing that neurons in the MM project directly to AMT and AVT, whereas those in the LM project to the antero‐dorsal thalamus (ADT). Together, these data revealed that PV and Drd2 neurons form two distinct subnetworks (DS→PV→AVT vs VS→Drd2→AMT) by receiving long‐range excitatory synaptic inputs from DS and VS and projecting their excitatory synapses onto two different neuronal classes in the ATN.

### 2.7 Two Cell‐Type Specific Circuits for Place and Object Recognition Memory

**Figure 6:**
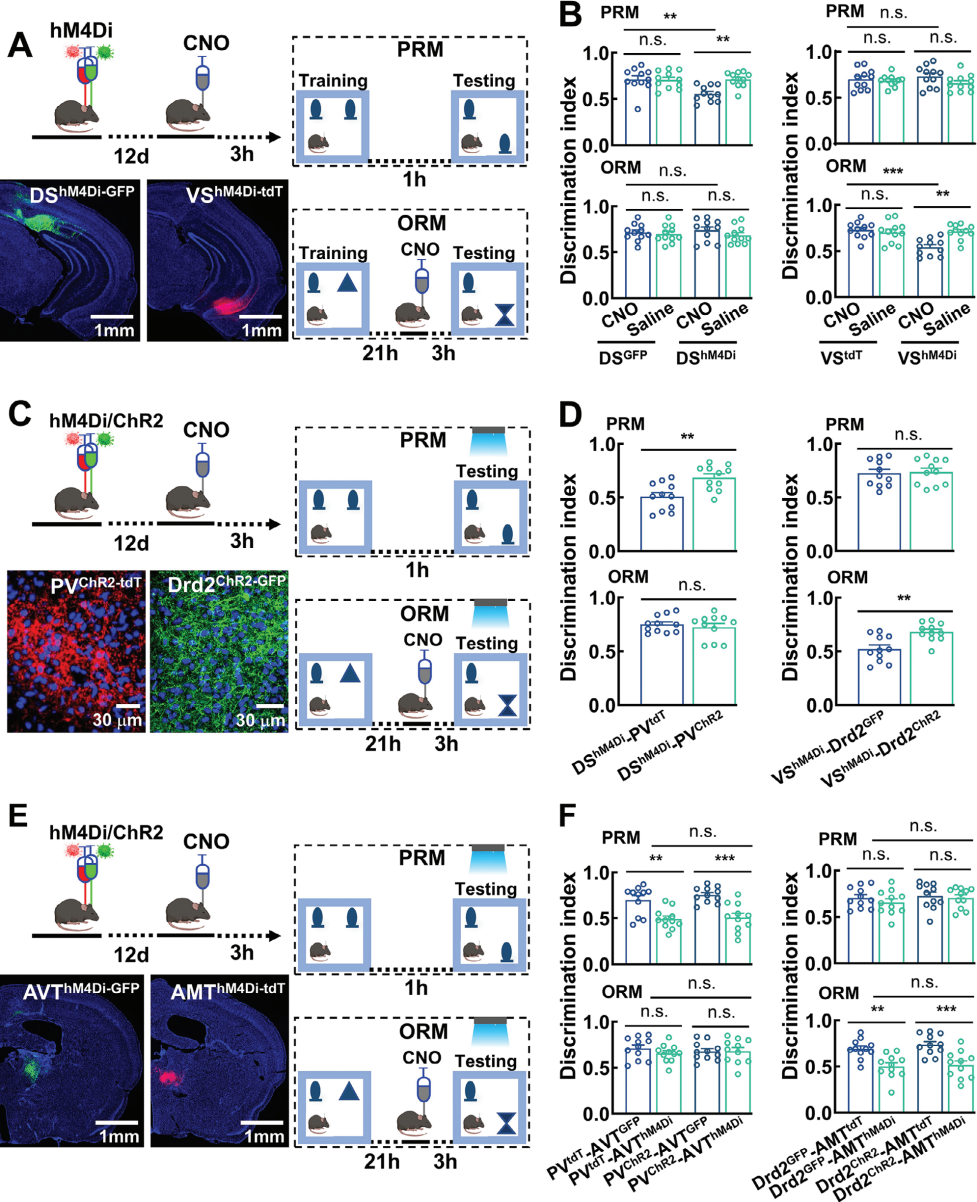
Distinct roles of DS→PV→AVT and VS→Drd2→AMT circuits in selective memory. A) Experimental procedures and representative images showing the expression of hM4Di in DS (DS^hM4Di^) and VS (VS^hM4Di^) neurons. B) Bar graphs showing that terminal inhibition of DS^hM4Di^ and VS^hM4Di^ by application of CNO but not saline impairs PRM (DS^hM4Di^ terminal inhibition) and ORM (VS^hM4Di^ terminal inhibition). Data are mean ± SEM (*n* = 11 mice per group, adjusted **P < 0.01 ***P < 0.001, two‐way ANOVA followed with Tukey's test). C) Experimental procedures and representative images showing the expression of ChR2 in PV (PV^ChR2^) or Drd2 (Drd2^ChR2^) neurons in DS^hM4Di^ or VS^hM4Di^ mice. D) Bar graphs showing that optogenetic stimulation of PV^ChR2^ or Drd2^ChR2^ neurons during the testing session reversed the terminal inhibition of DS^hM4Di^ or VS^hM4Di^ neurons. Data are mean ± SEM (*n* = 11 mice per group, **P < 0.01, *t*‐test). E) Experimental procedures and representative images showing the expression of hM4Di in AVT or AMT neurons in PV^ChR2^ or Drd2^ChR2^ mice. F) Bar graphs showing that chemogenetic inhibition of AVT or AMT neurons occludes the effects of optogenetic activation of PV or Drd2 neurons in PRM or ORM. Data are mean ± SEM (*n* = 11 mice per group, adjusted **P < 0.01, ***P < 0.001, two‐way ANOVA followed with Tukey's test).

Subsequently, we asked whether the direct projections from DS→PV→AVT and VS→Drd2→AMT neurons are essential for spatial and object recognition memory, we first engineered DS and VS neurons with the expression of inhibitory G‐protein coupled receptor hM4Di‐GFP/tdT by injecting ΔG‐RV‐DRE virus particles into the MM of PV^TVA/G^ and Drd2^TVA/G^ mice followed with rAAV2/9‐CaMK‐IIα‐dDIO‐hM4Di‐GFP or rAAV2/9‐CaMK‐IIα‐dDIO‐hM4Di‐tdT virus particles into the dSub or the vSub (**Figure** **6**A). Electrophysiological recordings confirmed the high efficacy of local application of hM4Di receptor agonist clozapine N‐oxide (CNO) in inhibition of action potential firings (Figure S9A, Supporting Information) and DS→PV and VS→Drd2 synaptic transmission (Figure S9B, Supporting Information). We then locally applied CNO (1 µL of 5 × 10^−3^ m) onto the MM of DS^hM4Di‐GFP^or VS^hM4Di‐tdT^ mice and deactivated either DS→PV or VS→Drd2 projections in the SRM tasks. This deactivation showed no significant change in exploration with objects in the training session (Figure S9C, Supporting Information), but it significantly decreased the number and duration of investigations with the novel place in the PRM task (0.56 ± 0.03 in DS^hM4Di^ mice vs 0.71 ± 0.04 in DS^GFP^ mice, Figure 6B; Figure S9D, Supporting Information) and novel object in the ORM task (0.54 ± 0.3 in VS^hM4Di^ mice vs 0.73 ± 0.03 in VS^tdT^ mice, Figure 6B; Figure S9D, Supporting Information). These behavioral phenotypes induced by inhibition of DS→PV and VS→Drd2 projections captured the behavioral phenotypes observed in PV^Kcnn4−^ and Drd2^Cacna1h−^ mice.

Deficits of place and object recognition memory caused by inhibition of DS→PV and VS→Drd2 subcircuits led us to assess whether activation of either circuit affects these different memory elements in a feature (where vs what)‐dependent way. We performed optogenetic activation and examined whether this activation counteracts the effects of DS→PV and VS→Drd2 terminal inhibition. We engineered PV and Drd2 neurons with the expression of ChR2 in DS^hM4Di^ and VS^hM4Di^ mice, resulting in DS^hM4Di^‐PV^ChR2^ and VS^hM4Di^‐Drd2^ChR2^ mice (Figure 6C). DS^hM4Di^‐PV^tdT^ and VS^hM4Di^‐Drd2^GFP^ mice were used as controls. The functionality of ChR2 channel was confirmed by using electrophysiological recordings, and optogenetic activation of PV and Drd2 neurons respectively enhanced the firings of AVT and AMT neurons (Figure S10A–D, Supporting Information). We then performed two independent optogenetic experiments; one activated on the training and another activated on the testing session of the tasks. Optogenetic activation on the training session did not modulate the behavioral performance (Figure S10E,F, Supporting Information), while on the testing session it completely rescued the behavioral deficits induced by DS→PV and VS→Drd2 terminal inhibitions. In the PRM task, discrimination indexes were 0.51 ± 0.04 in DS^hM4Di^‐PV^tdT^ mice vs 0.69 ± 0.03 in DS^hM4Di^‐PV^ChR2^ mice (Figure 6D; Figure S10G,H, Supporting Information). In the ORM task, the discrimination indexes were 0.52 ± 0.04 in VS^hM4Di^‐Drd2^GFP^ mice vs 0.68 ± 0.03 in VS^hM4Di^‐Drd2^ChR2^ mice (Figure 6D; Figure S10G,H, Supporting Information). Notably, the effects of optogenetic activation of PV and Drd2 neurons on place and object recognition memory can be reversed by chemogenetic inhibitions of AVT and AMT neurons (by expressing hM4Di and local injection of CNO, Figure 6E,F; Figure S11, Supporting Information). Together, these data revealed that PV and Drd2 neurons functionally constitute two distinct DS→PV→AVT and VS→Drd2→AMT subcircuits for the retrieval of place and object recognition, respectively.

### 2.8 Cell‐Type Specific Neural Dynamics in Place and Object Recognition Memory

**Figure 7:**
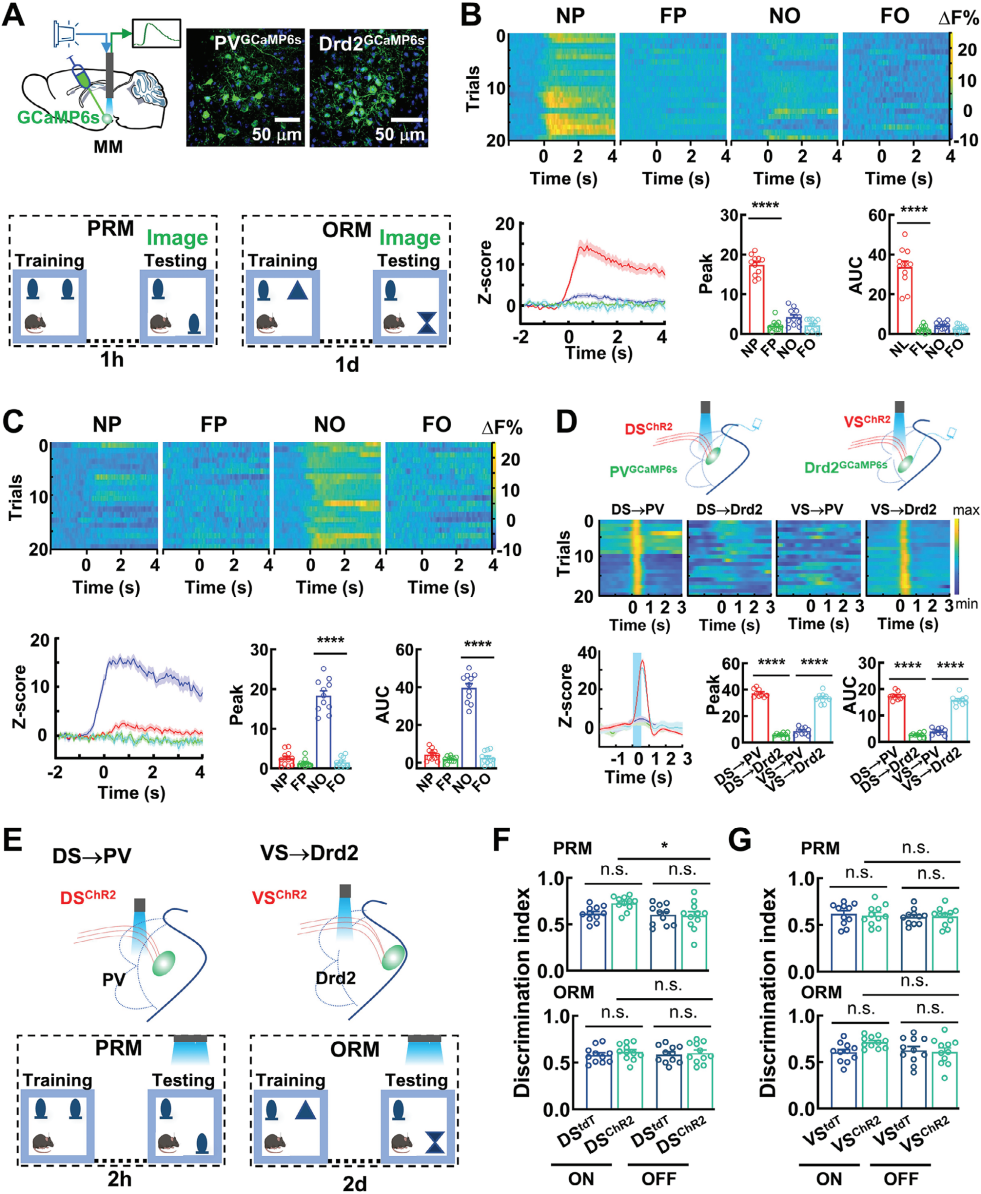
Cell‐type specific neural dynamics in place and object recognition memory. A) Experimental schematic diagram (top, left) of the viral vector strategy and optical fiber placement, and representative images (top, right) procedures (bottom) for calcium photometry in vivo, of GCaMP6s expression in PV (PV^GCaMP6s^) and Drd2 (Drd2^GCaMP6s^) neurons. B,C) Activation of PV and Drd2 neurons, respectively, processes place and object information. Heat maps, Z‐score, mean peak, and AUC showing calcium signals of PV^GCaMP6s^ and Drd2^GCaMP6s^ neurons during exploration with novel (NP, red) and familiar (FP, green) places, or novel (NO, blue) and familiar (FO, light blue) objects in the testing session of the SRM tasks. Time 0 s indicates start of exploration events. Data are mean ± SEM (*n* = 11 mice per group, adjusted ****P < 0.0001, one‐way ANOVA followed with Bonferroni's post hoc test). D) Optogenetic stimulation of DS and VS neurons effectively activate PV and Drd2 neurons. Heat maps, Z‐score, mean peak, and AUC showing calcium signals in PV^GCaMP6s^ and Drd2^GCaMP6s^ neurons in response to deliveries of blue light onto of DS^ChR2^ and VS^ChR2^ neurons (DS→PV, red; DS→Drd2, green; VS→PV, blue; VS→Drd2, light blue). Time 0 s indicates start of light stimulation. Data are mean ± SEM (*n* = 9 mice per group, ****P < 0.0001, *t*‐test). E) Experimental procedures for the PRM and ORM tasks with longer delays. F,G) Activation of DS→PV and VS→Drd2 subcircuits for place and object recognition memory. Bar graphs showing the discrimination index of DS^ChR2^ versus DS^tdT^ mice (F) and VS^ChR2^ versus VS^tdT^ mice (G) for PRM and ORM, with (on) or without (off) optogenetic stimulation of axon terminals from DS^ChR2^ or VS^ChR2^ neurons. Data are mean ± SEM (*n* = 11 mice per group, adjusted *P < 0.05, two‐way ANOVA followed with Tukey's test).

We next asked whether activation of PV and Drd2 neurons can be used to selectively affect these two distinct components of memory by examining calcium (Ca^2+^) dynamics as mice went through the SRM tasks. For this purpose, we engineered PV and Drd2 neurons with the expression of Ca^2+^ indicator GCaMP6s by injecting the rAAV2/9‐CaMK‐IIα‐fDIO‐GCaMP6s and the rAAV2/9‐CaMK‐IIα‐DIO‐GCaMP6s virus particles into the MM of PV^FLP^ or Drd2^CRE^ mice. The recording fiber was placed upon the MM and light at 465 nm was used to excite GCaMP6s (**Figure** **7**A). We showed that PV neurons increased their Ca^2+^ activity just in the testing session of the PRM task. The average activity change across the population was much higher for exploring novel place than novel object (Figure 7B; Figure S12A, Supporting Information). By contrast, Drd2 neurons exhibited a larger increase in Ca^2+^ dynamics in response to novel object in the testing session of the ORM task (Figure 7C; Figure S12B, Supporting Information). No significant change in Ca^2+^ transient from either PV or Drd2 neurons when mice explored a familiar place or object (Figure 7B,C). Ca^2+^ activity in PV and Drd2 neurons was increased in response to novel object or place in the testing trials. This increase of Ca^2+^ dynamics was completely abolished by knockdown of *Kcnn4* or *Cacna1h* in these neurons (Figure S13, Supporting Information). These results together demonstrate that Ca^2+^ dynamics in PV and Drd2 neurons contain signals essential for the retrieval of place and object recognition memory.

We next developed optogenetics to dissect the contributions of single unit Ca^2+^ dynamics from PV and Drd2 neurons in place and object recognition memory by delivering light to either DS or VS axon terminals. Spontaneous recognition memory was assessed using two additional delays (2 h for PRM task and 2 d for ORM task) for the testing session. Longer delays represent more demanding versions of the tasks, which would avoid any issues due to a potential ceiling effect induced by optogenetic stimulation on the animals' performance.^[^ ^27^ ^]^ Blue light were delivered to activate DS or VS neurons. This activation caused an increase of Ca^2+^ transients in PV or Drd2 neurons (Figure 7D). The mice, in which DS→PV and VS→Drd2 circuits were activated, displayed their behavioral changes in the PRM and the ORM tasks; an increase in their explorations with novel place (DS→PV activation; 0.62 ± 0.02 in DS^tdT^ mice vs 0.73 ± 0.02 in DS^ChR2^ mice) and novel object (VS→Drd2 activation; 0.60 ± 0.03 in VS^tdT^ mice vs 0.71 ± 0.02 in VS^ChR2^ mice, Figure 7E–G). Identical sets of experiments were performed with DS^ChR2^ and VS^ChR2^ mice in the absence of light delivery and no changes were found in behavior (DS→PV inactivation; 0.60 ± 0.03 in DS^tdT^ mice vs 0.59 ± 0.05 in DS^ChR2^ mice, VS→Drd2 inactivation; 0.63 ± 0.04 in VS^tdT^ mice vs 0.61 ± 0.04 in VS^ChR2^ mice, Figure 7F,G).

## 3 Discussions

Our data in the present study have provided cell‐type specific structural and functional evidence underlying how two different subtypes of neurons (PV versus Drd2) in the MM are organized to process mnemonic information. For the structural standpoint, we have generated a comprehensive single‐cell‐resolution atlas of two excitatory glutamatergic neuronal subtypes located in two discrete subregions (the pars lateralis vs the basalis) of the MM, revealing their molecular and cellular heterogeneity in relation to behaviorally distinct subcircuits. From a functional perspective, our work has illustrated the selectivity that brain can employ to mediate different elements (where and what) of episodic memory, exploiting dissociations in architecture of neuronal subtypes (e.g., PV neurons in the pars lateralis vs Drd2 neurons in the basalis of the MM) and memory components. Together, this work has provided a multiscale demonstration that the molecular and cellular heterogeneity within the MM can be the biological substrate for place and object recognition memory.

In this study, we have examined the behaviors of mice in a series of spontaneous recognition tasks, based on the previous studies.^[^ ^28^ ^]^ The tasks included the training and the testing sessions and involved spontaneous exploration of object sets in a chamber, with two different categories of novelty (place versus object) introduced in each task; place versus object recognitions. During the training session, we allowed mice to explore two identical objects in the chamber. Our data revealed that genetic manipulations (inhibition or activation) of PV or Drd2 neurons did not alter the total exploration times with each of the two objects, indicating that neither PV nor Drd2 neurons are associated with spontaneous memory formation or learning. In the testing session, animals encountered one novel object and a displaced familiar one. To explore whether mice were individually expressing a preference for the novel over the familiar place or object, we analyzed a discrimination index by dividing the amount of time spent exploring the novelty by the total time spent exploring both the familiar and the novel place or object. This analysis allowed for controlling the individual differences simply in exploratory behavior. Since memory decays over time, we also varied the time interval between the training and testing sessions in order to explore the comparative decay of information for object versus place. We asked whether manipulations of PV or Drd2 neurons would alter overall explorations of both, the novel and the familiar objects, or, in the case of an action on spontaneous recognition memory for place versus object, would bias exploration toward either of these options. Based on our data inhibition or activation of PV or Drd2 neurons decreased or increased the behavioral preference for exploring with either novel place or object. Importantly, we have also conducted the tasks with a short delay between the training and the testing session. Together with our findings, we conclude that the behaviors of PV and Drd2 neurons in the tasks are driven by the retrieval ability of object (ORM) or place recognition memory (PRM), but not by changing sensory or motor abilities necessary to complete the tasks.

Our work has used a state‐of‐the‐art imaging with genetically modified virus tracing approach and investigated the extent and organizational principles of neuronal cell types in the MM. We have identified PV neurons in this brain structure as excitatory glutamatergic projection neurons with molecular signatures distinct from those of their counterparts in the other brain regions.^[^ ^29^ ^]^ Specifically, our data have revealed that all PV neurons express excitatory glutamatergic neuronal marker genes such as *vGluT2* and *CAMK‐IIα* and release an excitatory glutamate neurotransmitter, while their firing patterns and intrinsic excitability such as fast‐spiking with a large afterhyperpolarization shared the similarity with those seen in classical GABAergic inhibitory interneurons.^[^ ^17^ ^]^ These findings are contrary to the long‐held belief that PV represents a specific marker for a subset of GABAergic interneurons,^[^ ^29^ ^]^ hence providing a concept of GABAergic neuronal classification and function.

Our work has also shown a sharp spatial division of PV versus Drd2 neurons in the MM, partitioning the pars lateralis and the basalis into two distinct subregions. Using unbiased single and population cell RNA‐seq, we have identified over hundreds of genes that are differentially expressed in these spatially divided neuronal subtypes, showing the anatomical spatial organizational scheme of two distinct neuronal subtypes was conserved across gene expression patterns. These results are consistent with recent studies from ours^[^ ^14a^ ^]^ and others^[^ ^30^ ^]^ in which a marked degree of transcriptional variability in multiple brain regions and neuronal cell types was found, although the extent of this variability notably differ between neuronal types and brain subregions, in retina, a single or a few genes involving the developmental cues are reported, whereas in the striatum, the CA1 hippocampus and the thalamic reticular nucleus^[^ ^14^, ^30^ ^]^ similar to the MM in our present study, over hundreds of genes exhibiting variation (249 genes in PV neurons vs 722 genes in Drd2 neurons) were found. Notably, this transcriptional variability between two distinct neuronal subtypes correlates with their electrophysiological properties, connectivity, anatomical organization and place and object recognition memory, illustrating that the mammillary nucleus cognitive hub contains structurally parallel pathways, embedded within two distinct neuronal subtypes, which differentially process place and object information. This finding likely provides a general organizational principle of cell‐type specific circuits across wide brain regions for sensation, action, and cognition.

In our present study, we have analyzed Ca^2+^ signals from freely behaving mice and demonstrated that PV neurons increase their Ca^2+^ activity in the PRM task. The average activity change across the population is much higher for exploring novel place than novel object. By contrast, most Drd2 neurons exhibit a larger increase in Ca^2+^ dynamics in response to novel object than novel place in the ORM. Representations of place and object recognition memory in PV and Drd2 neurons may be inherited from activations of their respective presynaptic neurons from the dorsal versus the ventral subiculum. Consistent with this notion, a recent work indicates that dorsal subiculum neurons projecting to MB selectively process spatial information.^[^ ^31^ ^]^ Further studies will be required to examine Ca^2+^ dynamics of the ventral subiculum neurons in processing object information. Together, it is plausible that Ca^2+^ dynamics in PV and Drd2 neurons that are driven by DS and VS neurons may contain signals essential for place and object recognition memory.

Previous studies indicated that recognition memory can be processed through interactions among multiple brain regions including the cortex, the thalamus, and the hippocampus. Novel object recognition memory is generally documented in the perirhinal cortex, whereas novel place recognition memory primarily relies on the hippocampus.^[^ ^28^ ^]^ The hippocampus can be functionally and structurally divided into the dorsal and ventral subregions.^[^ ^32^ ^]^ The dorsal hippocampus is associated with place recognition memory.^[^ ^33^ ^]^ Consistent with this notion, we found that PV neurons receive projections mainly from the dorsal subiculum for the retrieval of place recognition memory, while Drd2 neurons are selectively innervated by the ventral subiculum for the retrieval of object recognition memory. These findings are in line with the existing evidence, showing that PV and Drd2 neurons can integrate the spatial with the nonspatial components of memory through distinct synaptic inputs from the dorsal and the ventral hippocampus.^[^ ^33^, ^34^ ^]^ Earlier studies revealed that the perirhinal cortex plays roles in object recognition memory.^[^ ^28^, ^35^ ^]^ Although we did not observe a direct connection between Drd2 and the perirhinal cortex, neurons in the perirhinal cortex may project their axon fibers onto either the ventral hippocampus or the downstream targets of Drd2 neurons in the thalamus for processing novel object recognition.

In this study, we chose male mice to perform the experiments, while female mice can also be used for SRM tasks.^[^ ^36^ ^]^ Therefore, our results need to be further verified using female mice in the future.

## 4 Conclusion 

Our study revealed two subtypes of glutamatergic neurons PV and Drd2 neurons in the medial mammillary nucleus form two distinct cell‐type specific circuits for the retrieval of place and object recognition memory.

## 5 Experimental Section

### Mice

Male mice at 120 ± 5 days old of age were used in this study. Mice were bred and reared under the same conditions in accordance with institutional guidelines and the Animal Care and Use Committee of the animal core facility at Huazhong University of Science and Technology, Wuhan, China, and housed in groups of three to five mice/cage under a 12‐h light‐dark cycle, with lights on at 8:00 am, at a consistent ambient temperature (21 ± 1 °C) and humidity (50 ± 5%). All behavioral tests were conducted during the light phase of the cycle. Animals were randomly allocated to the different experimental conditions in this study, as described before.^[^ ^14^ ^]^ Drd2^CRE^ (MMRRC: 032108) mice were provided by Dr. Yisheng Lv at Department of Physiology, Huazhong University of Science and Technology, Wuhan, China. PV^FLP^ (PV‐T2A‐Flpo) mice were generated by Cyagen Biosciences Inc., Guangzhou, China.

### Single‐Cell RNA‐seq

The clustering was used to identify molecularly distinct types of cells in the mammillary nucleus from mice. The mammillary nucleus from adult male mice (120 ± 5 days old of age) with C57BL/6 genetic background was dissociated into a single‐cell suspension and kept in cold HBSS solution (SIGMA) with 0.2% BSA and 0.3% glucose, as described before.^[^ ^14a^ ^]^ The mammillary body on the acute brain slices in the artificial cerebrospinal fluid (ACSF in mm: 124 NaCl, 3 KCl, 26 NaHCO_3_, 1.2 MgCl_2_, 1.25 NaH_2_PO_4_2H_2_O, 10 C_6_H_12_O_6_, and 2 CaCl_2_ at pH 7.4, 305 mOsm) was precisely isolated under a stereoscope. A total of 35 male C57 mice at 3 months of age were collected with their mammillary body.

Using single‐cell 3′ Library and Gel Bead Kit v3.1 (10× Genomics, 1000268) and Chromium Single Cell G Chip Kit (10× Genomics, 1000120), the samples (700–1200 living cells per microliter determined by Count Star) were loaded onto the Chromium single cell controller (10× Genomics) to generate single‐cell gel beads in the emulsion according to the manufacturer's protocol. Captured cells were lysed and the released RNA were barcoded through reverse transcription (RT) in individual GEMs. RT was performed on a S1000TM Touch Thermal Cycler (Bio Rad) at 53 °C for 45 min, followed by 85 °C for 5 min, and hold at 4 °C. The cDNA was generated and then amplified, and quality assessed using an Agilent 4200 (performed by CapitalBio Technology, Beijing).

The sequencing data were processed by 10× cell ranger. The Cell Ranger software was obtained from 10× Genomics website https://support.10xgenomics.com/single‐cell‐gene‐expression/software/downloads/latest. Single‐cell RNA‐seq libraries were constructed using Single Cell 3′ Library and Gel Bead Kit v3.1. The initial analysis using the 10× genomic pipeline (cell ranger) yielded 6753 cells with a median of 2772 genes per cell. The libraries were finally sequenced using an Illumina Novaseq6000 sequencer with a sequencing depth of at least 55618 reads per cell with pair‐end 150 bp (PE150) reading strategy (performed by CapitalBio Technology, Beijing). Cells whose gene number was less than 200, or gene number ranked in the top 1%, or mitochondrial gene ratio was more than 25% were regarded as abnormal and filtered out.

Dimensionality reduction was performed using PCA, and visualization was realized by t‐SNE. The first ten major components were used to generate clusters by K‐means algorithm and graph‐based algorithm, respectively. Cells were selected with the FindClusters function (setting resolution = 0.6) and the same criterion applies to the neuron re‐clustering. While FindAllmarkers function (with default parameters, except setting min.pct = 0.1, logfc.threshold = 0.25) in Seurat 3.0 (R package) was used to find out the top genes per cluster. Clusters were identified based on marker gene expression.

Of the 6753 cells, 3694 cells were neurons and clustered into three neuronal subclusters. Specifically, 1893 Rprm‐expressing neurons in the MM and 126 Tac2‐expressing neurons in the LM were estimated. In this study, the numbers of neurons in the MM versus the LM based on their expression of *Rprm* and *Tac2* marker genes were estimated, as reported by a recent work.^[^ ^13^ ^]^ Rprm‐expressing neurons were divided into four clusters, and PV, Drd2, NTS, and Nos1 were the specific top genes of these four clusters.

### Population Cell RNA‐seq

PV and Drd2 neurons were purified from the MM from mice (*n* = 3 mice per group from three different litters). The MM slices were prepared and digested in buffer that contained 10 × 10^−3^ m Tris‐Cl (pH 7.6), 50 × 10^−3^ m NaF, 1 × 10^−3^ m Na_3_VO_4_, 1 × 10^−3^ m edetic acid, 1 × 10^−3^ m benzamidine, 1 × 10^−3^ m PMSF, 1 mg/10 mL papain, and a mixture of aprotinin, leupeptin, and pepstatin A (10 mg mL^−1^ each) for 30 min. Suspended PV^tdT^ and Drd2^GFP^ neurons were automatically isolated using an S3e Cell Sorter (Bio‐Rad), homogenized, and diluted with ice‐cold RNA‐later solution (Sigma‐Aldrich, #R0901). Single cells were lysed in 1 µL of cell lysis buffer containing RNasein Inhibitor and the RNA were released. Converted mRNA to cDNA by RT reaction with annealing of oligo‐DT primers of known sequence. PCR amplification was performed using these cDNA products. The amplified cDNA was incubated with Tn5 transposase to fragment the almost full‐length double‐stranded cDNA. The product was purified by a bead cleanup and then sent for Illumina sequencing.

PCR product was purified using Agencourt AMPure XP beads (Beckman Coulter, A63880) and eluted in EB buffer (Qiagen, 19086). Purified cDNA was measured on the Agilent 2100 BioAnalyzer with high sensitivity DNA Kit (Agilent, 5067‐4626). Sequencing libraries were prepared using a NexteraXT DNA Library Preparation Kit (Illumina, FC‐131‐1024). RNA‐Seq libraries were sequenced on an Illumina Nova Seq platform with average depth of >1 million reads per sample. To estimate genome mapping rate, TopHat (2.1.0) was used to align reads to mouse mm10 UCSC genome with default parameters. To determine the genes expressed, an RNA‐Seq expression estimation (expectation‐Maximization RSEM v1.2.8) was performed with default parameters on alignments created by Bowtie2 (2.2.9) on UCSC gene annotation. Reads mapped to the exon regions of each gene were counted by featureCounts (Bioconductor, Subread‐1.5.1) and then RPKMs were calculated. A total 259 PV neurons (121) and Drd2 (138) neurons were sequenced, and 12522 genes per cell were obtained.

Genes differentially expressed between groups were identified using the edgeR package (version 3.12.1). A *p*‐value cutoff of 0.05 and fold‐change cutoff of 2 were used to judge the statistical significance of gene expression differences.

### Anatomical Locations of Mammillary Nuclei

The perimeter and landmarks of neurons in the mammillary nuclei were traced from a low‐magnification image (10×) of the brain coronal sections stained with DAPI at −2.8 to −3.3 mm anterior‐posterior relative to bregma. Neurons in the mammillary nucleus were located by using a mouse brain atlas,^[^ ^37^ ^]^ for each brain and compared with the brain atlas based on the structure of the hypothalamus, ventricles, and mammillary nuclei.

### Genetically Targeting PV and Drd2 Neurons In Vivo

To determine the specificity of FLP recombination in PV and CRE recombination in Drd2 neurons, it was assessed whether FLP activates the reporter expression only in PV neurons, whereas CRE activates the reporter expression only in Drd2 neurons. 0.1 µL of the rAAV2/9‐CaMK‐IIα‐fDIO‐tdT virus (1.2 × 10^13^ genomic particles mL^−1^) or the rAAV2/9‐CaMK‐IIα‐DIO‐GFP virus (1.5 × 10^13^ genomic particles mL^−1^) was injected into the MM of PV^FLP^ or Drd2^CRE^ mice. The coordinates of the stereotaxic virus injections were AP: −2.8 mm, ML: 0 mm, and DV: 5.3 mm from bregma. To avoid the diffusively infection, the needle (WPI, nanofil) was retained at MM 30 min after the release of the virus and then slowly retracted. This injection resulted in the expression of tdT in PV neurons and GFP in Drd2 neurons only (Figure 2C; Figure S2D, Supporting Information).

### CRISPRE Cas9 Gene Knockdown In Vivo

*Kcnn4* and *Cacna1h* genes in PV and Drd2 neurons, respectively, were targeted. To increase the knockdown efficiency for a single gene, five exon‐targeting sgRNAs with highest predicted on‐target efficiency and lowest predicted off‐target rate were designed (https://benchling.com, Table S4, Supporting Information). The sgRNAs were synthesized by Integrated DNA Technologies (Sangon Biotech Co., Ltd., Shanghai, China) and cloned into the sgRNA‐expressing vector (pAAV‐U6‐sgRNA‐scaffold (SpCas9), Taitool Co., Ltd., Shanghai, China). The sgRNA sequences were individually sequenced from the U6 promoter. To generate sgRNA libraries, sgRNA targeting genes were concentrated by isopropanol precipitation. The libraries were electroporated at ≈75 ng µL^−1^ using Endura Electrocompetent cells (Novo Biotec, 60242‐2) according to the manufacturer's instructions. Midiprep was made using Quick‐DNA Midiprep Plus Kit (Zymo Research, D4075). Five sgRNAs that specifically target to *zfy2* (sgRNAs‐*zfy2*) were used as the negative control. *Zfy2* was used, as it was not expressed in neurons of rodent brain. 0.1 µL of the rAAV2/9‐CaMK‐IIα‐fDIO‐SpCas9 (7.5 × 10^12^ genomic particles mL^−1^) and 0.1 µL of the rAAV2/9‐CaMK‐IIα‐fDIO‐tdT (1.0 × 10^13^ genomic particles mL^−1^) or 0.1 µL of the rAAV2/9‐CaMK‐IIα‐DIO‐SpCas9 (8.5 × 10^12^ genomic particles mL^−1^) and 0.1 µL of the rAAV2/9‐CaMK‐IIα‐DIO‐GFP (1.1 × 10^13^ genomic particles mL^−1^) mixed with 0.1 µL of the rAAV2/9‐U6‐*Kcnn4*, *Cacna1h*, or *zfy2* sgRNAs (2.5 × 10^13^ genomic particles mL^−1^) infectious virus particles were injected into the MM of PV^FLP^ or Drd2^CRE^ mice. qPCR was used for in vivo knockdown validation. The primers are summarized in Table S4 (Supporting Information).

### Mapping Cell‐Type Specific Subcircuits of PV and Drd2 Neurons

To determine presynaptic neurons of PV versus Drd2 neurons, 0.1 µL of the FLP recombination‐dependent rAAV2/9‐CaMK‐IIα‐fDIO‐TVA/G‐tdT (1.5 × 10^13^ genomic particles mL^−1^), or CRE recombination‐dependent rAAV2/9‐CaMK‐IIα‐DIO‐TVA/G‐ GFP virus (2.0 × 10^13^ genomic particles mL^−1^) were injected into the MM of PV^FLP^ or Drd2^CRE^ mice, resulting in the production of PV^TVA/G^ versus Drd2^TVA/G^ mice, in which TVA/G was expressed in PV or Drd2 neurons. Two weeks after the injection, 0.2 µL of a genetically modified version of synaptic retrograde ΔG‐RV‐GFP (3 × 10^8^ genomic particles mL^−1^) or ΔG‐RV‐tdT (2.0 × 10^8^ genomic particles mL^−1^) virus particles were then injected into the same brain region of PV^TVA/G^ or Drd2^TVA/G^ mice. This injection resulted in the expression of GFP or tdT in DS (DS^GFP^) or VS (VS^tdT^) neurons, showing that PV and Drd2 neurons received synaptic inputs directly from DS (DS→PV) and VS (VS→DS) neurons, respectively.

For tracing direct synaptic targets of PV and Drd2 neurons, 0.1 µL of the FLP recombination‐dependent rAAV2/9‐CaMK‐IIα‐fDIO‐TK‐tdT (2.5 × 10^13^ genomic particles mL^−1^), or the CRE recombination‐dependent rAAV2/9‐CaMK‐IIα‐DIO‐TK‐GFP virus (8.5 × 10^12^ genomic particles mL^−1^) were injected into the MM of PV^FLP^ or Drd2^CRE^ mice, resulting in the production of PV^TK^ and Drd2^TK^ mice, in which TK‐tdT and TK‐GFP were expressed in PV (PV^TK^) and Drd2 (Drd2^TK^) neurons, respectively. Two weeks after the injection, 0.3 µL of a genetically modified version of anterograde Herpes simplex virus type 1 strain 129 (H129ΔTK‐GFP) virus (3.0 × 10^9^ genomic particles mL^−1^) for tracing PV^TK^ neuronal synaptic targets or H129ΔTK‐tdT virus (3.5 × 10^9^ genomic particles mL^−1^) for tracing Drd2^TK^ neuronal synaptic targets was then injected into the same region.

Generation of H129ΔTK‐GFP and H129ΔTK‐tdT virus particles was described before.^[^ ^38^ ^]^ Seven days after the injection of H129ΔTK‐GFP or H129ΔTK‐tdT virus particles, the mice were sacrificed and fixed. 24 h after fixation, brain sections were imaged under a laser confocal microscope (Zeiss LSM 800, Zeiss). With the assistance of helper virus, H129ΔTK‐GFP or H129ΔTK‐tdT transmits anterogradely through PV^TK^ and Drd2^TK^ neurons to their postsynaptic neurons, as described before.^[^ ^14a,d^ ^]^

### Electrophysiology and Optogenetics In Vitro

Voltage clamp recordings were performed using the following intracellular solution (in mm): 140 potassium gluconate, 0.05 EGTA, 10 HEPES, 2 Mg‐ATP, 0.2 Na‐GTP. When the optogenetically evoked synaptic responses were tested, the following intracellular solution (in mm) was used: 120 CsCH_3_SO_3_, 20 CsCl, 4 NaCl, 10 Hepes, 0.05 EGTA, 4 Mg‐ATP, 0.2 Na‐GTP, 3 QX314. To record DS→PV and VS → Drd2 synaptic transmission, 0.1 µL of ΔG‐RV‐DRE (2.0 × 10^8^ genomic particles mL^−1^) virus particles was injected into the MM of PV^TVA/G^ or Drd2^TVA/G^ mice, resulting in the expression of DRE in DS (DS^DRE^) or VS (VS^DRE^) neurons. Twelve days after the injection, 0.1 µL of the rAAV2/9‐CaMK‐IIα‐dDIO‐ChR2‐GFP (1.8 × 10^13^ genomic particles mL^−1^) or rAAV2/9‐CaMK‐IIα‐dDIO‐ChR2‐tdT (2 × 10^13^ genomic particles mL^−1^) virus particles was then injected into the dSub (AP: −3.1 mm, ML: ±1.7 mm, DV: 1.6 mm) or the vSub (AP: −3.6 mm, ML: ±2.75 mm, DV: 5.2 mm), resulting in the expression of ChR2 in DS (DS^ChR2^) or VS (VS^ChR2^). The slices from the mammillary nuclei and the subiculum were then prepared and transferred to a holding chamber that contained artificial cerebrospinal fluid (ACSF in mm: 124 NaCl, 3 KCl, 26 NaHCO_3_, 1.2 MgCl_2_, 1.25 NaH_2_PO_4_2H_2_O, 10 C_6_H_12_O_6_, and 2 CaCl_2_ at pH 7.4, 305 mOsm) at 32 °C for 30 min. The temperature was maintained at 22 °C for 60 min. A slice was transferred to a recording chamber, which was continuously perfused with oxygenated ACSF (2 mL min^−1^) at 22 °C. Intracellular and whole‐cell patch clamp recordings from PV^TVA/G^ or Drd2^TVA/G^ neurons, or DS^ChR2^ or VS^ChR2^ neurons, were performed, which were visualized under a fluorescent infrared‐phase‐contrast (IR‐DIC) Axioskop 2FS upright microscopy equipped with a Hamamatsu C2400‐07E infrared camera, as described before.^[^ ^14a^ ^]^ Every group included 3 mice, 4–5 cells per slice, 2–3 slices from each mouse. Signals were low‐pass‐filtered at 2 kHz and sampled at 10–20 kHz with a Digidata 1440A (Molecular Devices). Throughout recordings, series resistances (Rs) were carefully monitored. Recordings were discarded if the series resistance changed >20% and excluded for data analysis. Synaptic currents in PV^TVA/G^ or Drd2^TVA/G^ neurons were evoked by delivery of blue light (3 ms of 473 nm light at an intensity of ≈5 mW cm^−2^, DPSS laser, Inper Co., Ltd., Hangzhou, China) onto DS^ChR2^ or VS^ChR2^ axon terminals in the MM. For recording NMDA receptor‐mediated EPSCs, membrane potentials were hold at +60 mV in the presence of 20 × 10^−6^ m bicuculline (BIC, TOCRIS, 0130) and 20 × 10^−6^ m CNQX (TOCRIS, 0373). EPSCs were blocked by 1 × 10^−6^ m TTX (Abcam, 146038), rescued by 100 × 10^−6^ m 4‐aminopyridine (4‐AP, TOCRIS, 0940) and reversed by 50 × 10^−6^ m AP‐5 (TOCRIS, 0673).

Synaptic responses were evoked by optogenetic stimulation of ChR2 using white light‐emitting diodes (Inper Co., Ltd.), which were controlled by Mightex LED monitors (SLCAA02‐US or BLS‐1000‐2). Laser was collimated and reflected through a 40× water immersion objective, leading to a spot diameter of ≈300 µm and a maximum LED power at the focal plane of 29.1 mW. Blue light with 10 Hz trains of 1 ms flashes was delivered onto DS^ChR2^ or VS^ChR2^ axon terminals by centering the light spot over the recorded postsynaptic PV^TVA/G^ or Drd2^TVA/G^ neurons.

The intrinsic physiological properties of PV^tdT^ or Drd2^GFP^ neurons in the MM were intracellularly recorded by using sharp electrodes. Resting membrane potentials were measured within 2 min of break‐in. Steady‐state potentials were adjusted to −65 or −90 mV with intracellular current to test physiological properties in tonic or burst mode, respectively. Thresholds for action potential firings were measured and verified by visual inspection, as the potential at which the rate of rise became greater than 10 V s^−1^. Repetitive spiking properties in tonic and burst mode were measured using positive current steps (50–300 pA, 50 pA increments, 1 s duration). In burst mode, the frequency was calculated within the first 100 ms period of the spikes, whereas the spike number was calculated within the first 500 ms period of the spikes following hyperpolarization.

### Electrophysiology and Optogenetics In Vivo

0.1 µL of the FLP recombination‐dependent rAAV2/9‐CaMK‐IIα‐fDIO‐ChR2‐tdT (2.8 × 10^13^ genomic particles mL^−1^), or the CRE recombination‐dependent rAAV2/9‐CaMK‐IIα‐DIO‐ChR2‐GFP virus (2.8 × 10^13^ genomic particles mL^−1^) were injected into the MM of PV^FLP^ or Drd2^CRE^ mice, resulting in the expression of ChR2 in PV (PV^ChR2^) or Drd2 (Drd2^ChR2^). Two weeks post injection, mice were anesthetized with isoflurane (1%–3%) and placed in a head stereotaxic apparatus. The optical fiber (200 µm diameter) was lowered to the stimulating site (MM, AP: −2.8 mm, ML: 0 mm, DV: 5.3 mm) and electrodes were lowered to the recording sites (AVT, AP: −0.7 mm, ML: ±1.0 mm, DV: 3.3 mm or AMT, AP: −0.7 mm, ML: ±0.5 mm, DV: 3.8 mm). Plexon (Omniplex, USA) was used for data acquisition and spike sorting and Inper stimulator (Inper Co., Ltd., Hangzhou, China) was used for 473‐nm light stimulation. Light pulses of 2 s (3 mW mm^−2^ at the tips of the optical fiber) were delivered at MM with 15 s interval.

### Chemogenetics and Optogenetics In Vivo

DS and VS neurons were engineered with the expression of DRE recombinase by injecting 0.1 µL of the ΔG‐RV‐DRE virus particles (2.0 × 10^8^ genomic particles mL^−1^) into the MM of PV^TVA/G^ and Drd2^TVA/G^ mice. Following the expression of DRE in DS (DS^DRE^ mice) and VS (VS^DRE^ mice), 0.1 µL of the DRE‐dependent rAAV2/9‐CaMK‐IIα‐dDIO‐hM4Di‐GFP (1.6 × 10^13^ genomic particles/ml) or rAAV2/9‐CaMK‐IIα‐dDIO‐hM4Di‐tdT virus particles (1.2 × 10^13^ genomic particles mL^−1^) was then injected into the dSub or the vSub of the DS^DRE^ and VS^DRE^ mice. This injection resulted in the expression of inhibitory G‐protein coupled receptor hM4Di‐GFP or hM4Di‐tdT in DS (DS^hM4Di‐GFP^) and VS (VS^hM4Di‐tdT^) neurons. Two weeks after the injection, 1 µL of 5 × 10^−3^ m CNO was applied locally onto the MM of DS^hM4Di^ or VS^hM4Di^ mice 3 h before the behavioral tests.

To rescue the behavioral deficits induced by terminal inhibition of DS^hM4Di^ and VS^hM4Di^ neurons, ChR2 was expressed in PV (PV^ChR2^) and Drd2 (Drd2^ChR2^) neurons, resulting in the production of DS^hM4Di^→PV^ChR2^ and VS^hM4Di^→Drd2^ChR2^ mice. Specifically, 0.1 µL of the rAAV2/9‐CaMK‐IIα‐fDIO‐ChR2‐tdT (2.8 × 10^13^ genomic particles mL) or 0.1 µL of the rAAV2/9‐CaMK‐IIα‐DIO‐ChR2‐GFP (2.8 × 10^13^ genomic particles mL^−1^) infectious virus particles were injected into the MM of DS^hM4Di^ or VS^hM4Di^ mice. Two weeks after the injection, 1 µL of 5 × 10^−3^ m CNO was applied onto the dorsal (AP: −3.1 mm, ML: ±1.7 mm, DV: 1.6 mm) or the ventral (AP: −3.6 mm, ML: ±2.75 mm, DV: 5.2 mm) subiculum. 3 h after the CNO, blue light were delivered onto PV^ChR2^ and Drd2^ChR2^ neurons of DS^hM4Di^‐PV^ChR2^ or VS^hM4Di^‐Drd2^ChR2^ mice. Optogenetic stimulation used a 473‐nm wavelength light at power densities ranging from 0.1 to 5 mW mm^−2^ using a DPSS laser (Inper Co., Ltd.).

To block the effects of optogenetic activation, hM4Di was expressed in AVT or AMT neurons of PV^ChR2^ or Drd2^ChR2^ mice. Specifically, 0.1 µL of H129ΔTK‐DRE (5.2 × 10^9^ genomic particles mL^−1^) were injected into the MM of PV^TK‐ChR2^ or Drd2^TK‐ChR2^ mice. Following the expression of DRE in AVT (AVT^DRE^ mice) and AMT (AMT^DRE^ mice), 0.1 µL of the DRE‐dependent rAAV2/9‐CaMK‐IIα‐dDIO‐hM4Di‐GFP (1.6 × 10^13^ genomic particles mL^−1^) or rAAV2/9‐CaMK‐IIα‐dDIO‐hM4Di‐tdT virus particles (1.2 × 10^13^ genomic particles mL^−1^) was then injected into the AVT or the AMT of the AVT^DRE^ and AMT^DRE^ mice, resulting in the production of PV^ChR2^‐AVT^hM4Di^ or Drd2^ChR2^‐AMT^hM4Di^ mice. Two weeks after the injection, 1 µL of 5 × 10^−3^ m CNO was applied locally onto the anterior ventral thalamic (AP: −0.7 mm, ML: ±1.0 mm, DV: 3.3 mm) or the anterior medial thalamic nucleus (AP: −0.7 mm, ML: ±0.5 mm and DV: 3.8 mm) of PV^ChR2^‐AVT^hM4Di^ or Drd2^ChR2^‐AMT^hM4Di^ mice.

In PRM task, CNO was applied 3 h before training session. While in ORM, CNO was applied twice, 3 h before training and testing sessions. Optogenetic stimulation was conducted during training or testing sessions using a 473 nm wavelength light at power densities ranging from 0.1 to 5 mW mm^−2^ using a DPSS laser (Inper Co., Ltd.).

Animals were anesthetized with 1% pentobarbital sodium (50 mg kg^−1^) via intraperitoneal injection. An unjacketed optical fiber (200 µm diameter, Inper Co., Ltd.) bound to the tetrode‐containing silicone tube (150 µm) was implanted into the MM (AP: −2.8 mm, ML: 0 mm, DV: 5.3 mm); locations were validated by lesions of optical fiber and secured to the skull using jeweler's screws and dental cement. The animals for the experiments were used 7 d after the implantation.

### Ca^2+^ Photometry and Data Analysis

Mice were habituated to the experimenters and the experimental environments for 12 d. At least 12 d after the injection of the rAAV2/9‐CaMK‐IIα‐fDIO‐GCaMP6s (1.5 × 10^13^ genomic particles mL^−1^) or the rAAV2/9‐CaMK‐IIα‐DIO‐GCaMP6s (1.5 × 10^13^ genomic particles mL^−1^) viral and the optic fiber implantation, Ca^2+^ activity in PV^GCaMP6s^ or Drd2^GCaMP6s^ neurons were recorded by a fiber photometry system (Inper Co., Ltd.) in freely moving mice. Explorations with the familiar and the novel places or objects were recorded by a video camera (Logitech). During imaging session, two excitation LEDs reflected off di‐chronic plats to record GCaMP6s specific signal (465 nm) and nonspecific autofluorescence signals as a control (405 nm). The excitation blue light at the tip of the optic fiber was adjusted around 30 µW. Real‐time signal processors (RX8 and RZ5P, Trucker‐Davis Technologies) and software (OpenEx.v.2.20 for RX8 and Synapse v.90 for RZ5P, Tucker‐Davis Technologies) were used to control each LED output at a 50‐Hz sample rate to un‐mix signals from each LED. Onset of the behavior labels was marked as time zero to calculate the values of calcium transients change (Z‐score): onset of exploration of the objects. The baseline was taken 2.0 s prior to the start of each exploratory event. Z‐score was calculated by subtracting the mean baseline and further dividing by the standard deviation of the baseline distribution.

### Home Cage Behaviors

To evaluate natural exploratory motivation and repetitive behaviors, individual mice were video‐recorded alone in the PhenoTyper home cages (40 × 40 × 40 cm, Noldus, Holland) which provided a home environment for mice, with sufficient food and water supply. Mice were placed into the PhenoTyper home cages at 10 am in a 12‐h/12‐h light/dark‐ and air‐controlled room. After 24 h of habituation, locomotion and spontaneous behavior were detected by the infrared beam interruption of the animal body over 48 h. Automated video analysis was conducted by EthoVisionXT (Noldus, Holland) to measure the individual behaviors, as described before.^[^ ^14a^ ^]^

### Morris Water Maze

A 1.5 m‐diameter swimming pool was filled with white and nontoxic ink water. Pool temperature was maintained at 25 °C. A mouse was placed at the behavior room where this mouse was housed for the training for 1–2 d before training session, as described before.^[^ ^14a,d^ ^]^ The training session lasted for 6 d. In the first day of training, a mouse was allowed resting on the platform for 30 s and to have 90 s for finding the hidden platform. In case that a mouse did not find the platform within 90 s, this mouse was guided to find and stay at the platform for 30 s. Throughout the period of training session, the animal was required to perform a total of four trials, in which a mouse was released at four different randomized release points of the pool. Immediately, after the 6‐d training session, this mouse was required to perform probe trials at day 2 after the training session. In both training and probe trials, the behavioral tests were performed by an experimenter who was unaware of the genotypes and treatments. Behavior video analysis system (WMT‐100S, Chengdu Techman Co., Ltd., China) was used to analyze the swimming trajectory, latency in each quadrant and annulus.

### Spontaneous Recognition Memory Tests

Spontaneous recognition memory tests were conducted in a 50 × 50 × 38 cm white plexiglass square chamber with a magnetic, glossy, removable base. The base had a 50 × 50 cm black grid composed of 1 × 1 cm squares to allow for accurate object placement. The chamber was elevated 50 cm off the floor and a camera was mounted 75 cm above the chamber using a wall mount rack. A round magnet (35 mm diameter) was glued to the base of the objects to allow for stable attachment to the chamber floor.

Objects were 43 × 43 × 43 × 47 mm (pyramid object) or 43 mm diameter × 47 mm height (egg object) and were equal in surface area with the same materials. The mice were handled and habituated them to the behavioral chamber twice a day for four consecutive days prior to the day of tests. Handling and habituation consisted of 3 min of handling followed by placement into the behavioral chamber for 5 min. Each mouse was only tested in either an ORM or a PRM task. To control for odor cues, the open field arena and the objects were thoroughly cleaned with 10% odorless soap, dried, and ventilated for a few minutes between mice.

For all training and testing sessions, the mice were placed at the chamber with their head facing the wall located opposite the object location. For the training session, objects were allocated in the northwest and northeast corners of the chamber, 3 cm away from each wall. Object type and side of novel stimulus were counterbalanced (the novelty was introduced in the right or left side of the chamber).

PRM task was divided into the training and testing sessions. In the 10‐min training session, mice were allowed to explore two copies of a single object (A vs A′), after which mice were removed and placed back into their transport cage. 60 min after completion of the training, mice were subjected to the 5‐min testing session, in which they were required to explore the same two objects, but with one relocated to a novel place. The novel place was at the opposite corner of the previous place (south corner, counter balanced for side), 3 cm away from each wall. The exploration time for the familiar (FP) or the novel (NP) place was recorded. PRM was operationally defined by the DI that was calculated as the following: exploration time with NP was divided by total exploration time with both FP and NP.

ORM tests were also divided into the training and testing sessions. In the 10‐min training session, a mouse was placed at the chamber containing two different objects (A vs B), after which the mouse was removed and placed back into its home cage. 24 h after completion of the training, this mouse was subjected to the 5‐min testing session in a chamber containing both the previously encountered object (FO) and a novel (NO) object. ORM was operationally defined by the DI that was calculated as the following: exploration time with NO was divided by total exploration time with both FO and NO.

The exploration of the objects was considered as any investigative behavior (head orientation or sniffing occurring) or deliberate contact that occurred with each object in a distance < or = 2 cm or when touching with the nose. Nodas Behavioral Analysis System (17.0) was used to analyze the moving trajectory and the exploration time. All the exploration time during both the training and testing sessions was summarized in Table S5 (Supporting Information).

### Immunofluorescence and Fluorescence In Situ Hybridization

Mice were sacrificed by intraperitoneal injection of an overdose of pentobarbital sodium and were transcardially perfused with 100 mL of saline (0.9% w/v NaCl), followed 4% PFA. Brains were removed and post‐fixed in 4% PFA for 24 h. 30 µm coronal sections were sliced (Leica Microsystems, Wetzlar, Germany). Immunofluorescence was performed on free‐floating brain sections as described previously.^[^ ^14^ ^]^ In brief, staining was performed on 30 µm free‐floating coronal sections and blocked in 3% normal donkey serum (room temperature for 1 h). The sections were then incubated in 50 × 10^−3^ m Tris‐HCl buffer containing 3% donkey serum and 0.3% Triton X‐100 with the following primary antibodies: mouse anti‐PV (1: 1000, Swant PV235), mouse anti‐Drd2 (1: 200, Neuromab, N186/29) at 4 °C for 24 h. Sections were rinsed and then reacted with the conjugate‐adsorbed Alexa Fluor secondary antibodies (Thermo) at room temperature for 1 h. Sections were rinsed, dried, and cover‐slipped with fluorescence mounting medium (Invitrogen, P36961). For FISH, brain sections (20 µm) were made using a cryostat tissue slicer and mounted on glass slides. Slides were subsequently stored at −80 °C until use. RNA probes for PV (421931‐C3), Drd2 (406501‐C3), NTS (420441‐C1), Nos1 (437651‐C2), vGluT2 (319171‐C2), and vGAT (319191‐C2) as well as all other reagents for FISH were ordered from Advanced Cell Diagnostics (ACD, Hayward, CA). As described below, retrieval, pretreatment, hybridization, amplification, and detection were performed according to User Manual for Fixed Frozen Tissue (ACD). Sections were incubated in DAPI for 30 s, and slides were coverslipped with fluorescent mounting medium. Single or multiple labeling was viewed and imaged with a confocal laser‐scanning microscope (Zeiss LSM800 Examiner Z1) and analyzed with a three‐dimensional constructor (Image‐Pro Plus software). A confocal series of images were taken at 0.5 µm intervals through the region of interest, and optical stacks of 6–12 images were produced for the figures using 20× (U Plan XApochromat objective 20×/0.8, WD 0.6 mm). The absolute numbers of single, double, or triple labeled cells was quantified by sampling every section (image stacks) from the experimental animals, as described before.^[^ ^14a,b,d^ ^]^ For manual quantification, colocalization was performed on 20× images using the Cell Counter Image J plugin. All image Z‐stacks were max projected and stitched to obtain the full structure of the sample. In FISH, the counts of fluorescent puncta reflect the RNA expression level. Total PV, Drd2, NTS, and Nos1 neurons were quantified on a per cell basis, where cells were delineated by DAPI staining (Figure S1B, Supporting Information).

### Statistical Analysis

All values in the text and figure legends are represented as the mean ± SEM. *n* indicates number of animals or cells. Comparisons between two‐group distribution data were analyzed by two‐tailed Student's *t*‐test. Multiple group comparisons were assessed using one‐way or two‐way analysis of variance (ANOVA), followed with the Bonferroni's post hoc test or Tukey's test when significant main effects or interactions were detected. Significance was accepted for *p* < 0.05. Power calculations were performed using GraphPad Prism v9.0. Group sizes were estimated based on previous studies.^[^ ^14^ ^]^ Experimenters were blinded for the injection sites in the virus quantifications and for the experimental conditions in Ca^2+^ images and analysis. All electrophysiological recording and data analysis in the first round of CRISPR‐Cas9 studies were performed with the experimenters blinded to treatment conditions. All statistical analysis methods and results are summarized in Table S6 (Supporting Information).

## Conflict of Interest

The authors declare no conflict of interest.

## Author Contributions

L.L., Y.G., and W. J. contributed equally to this work. Y.L. and X.L. conceived and designed the studies and wrote the paper. X.L. and L.L. carried out the experiments including cell typing, synaptic tracing, CRISPR‐Cas9, RNA‐seq, and behavioral tests. Y.G. and W.J. performed the experiments including electrophysiology and Ca^2+^ images. X.T., J.Z., Z.H., Y.S., A.H., H.L., and L.Z. performed the experiments including genotyping, PCR, cell counting, and cloning. All authors contributed to the data analysis and presentation in the paper.

## Ethics Approval Statement

All experiments were done in accordance with guidelines from the National Natural Science Foundation of China and approved by the Animal Care and Use Committee of the Animal Core Facility at Huazhong University of Science and Technology, Wuhan, China (Approval No. 82271486).

## Supporting information



Supporting Information

## Data Availability

The data that support the findings of this study are available from the corresponding author upon reasonable request.
