# Supplementary material for: Cell‐Type Specific Circuits in the Mammillary Body for Place and Object Recognition Memory
Source: Adv Sci (Weinh). 2025 Feb 10;12(13):2409397. doi: 10.1002/advs.202409397 (PMC11967786; doi:10.1002/advs.202409397)
Supplement: Supplementary file 1 — Supporting Information [file ADVS-12-2409397-s001.pdf]

ADVANCED SCIENCE
Open Access

Supporting Information

for *Adv. Sci.*, DOI 10.1002/advs.202409397

Cell-Type Specific Circuits in the Mammillary Body for Place and Object Recognition Memory

Lanfang Li, Yiqing Guo, Wei Jing, Xiaomei Tang, Jinyu Zeng, Zhenye Hou, Yige Song, Aodi He, Hao Li, Ling-Qiang Zhu, Youming Lu and Xinyan Li**

Cell-type Specific Circuits in the Mammillary Body for Place and Object Recognition Memory

Lanfang Li, Yiqing Guo, Wei Jing, Xiaomei Tang, Jinyu Zeng, Zhenye Hou, Yige Song, Aodi He, Hao Li, Ling-Qiang Zhu, Youming Lu*, Xinyan Li*

Figure S1. Molecular defined cell types in the mammillary nucleus of mice

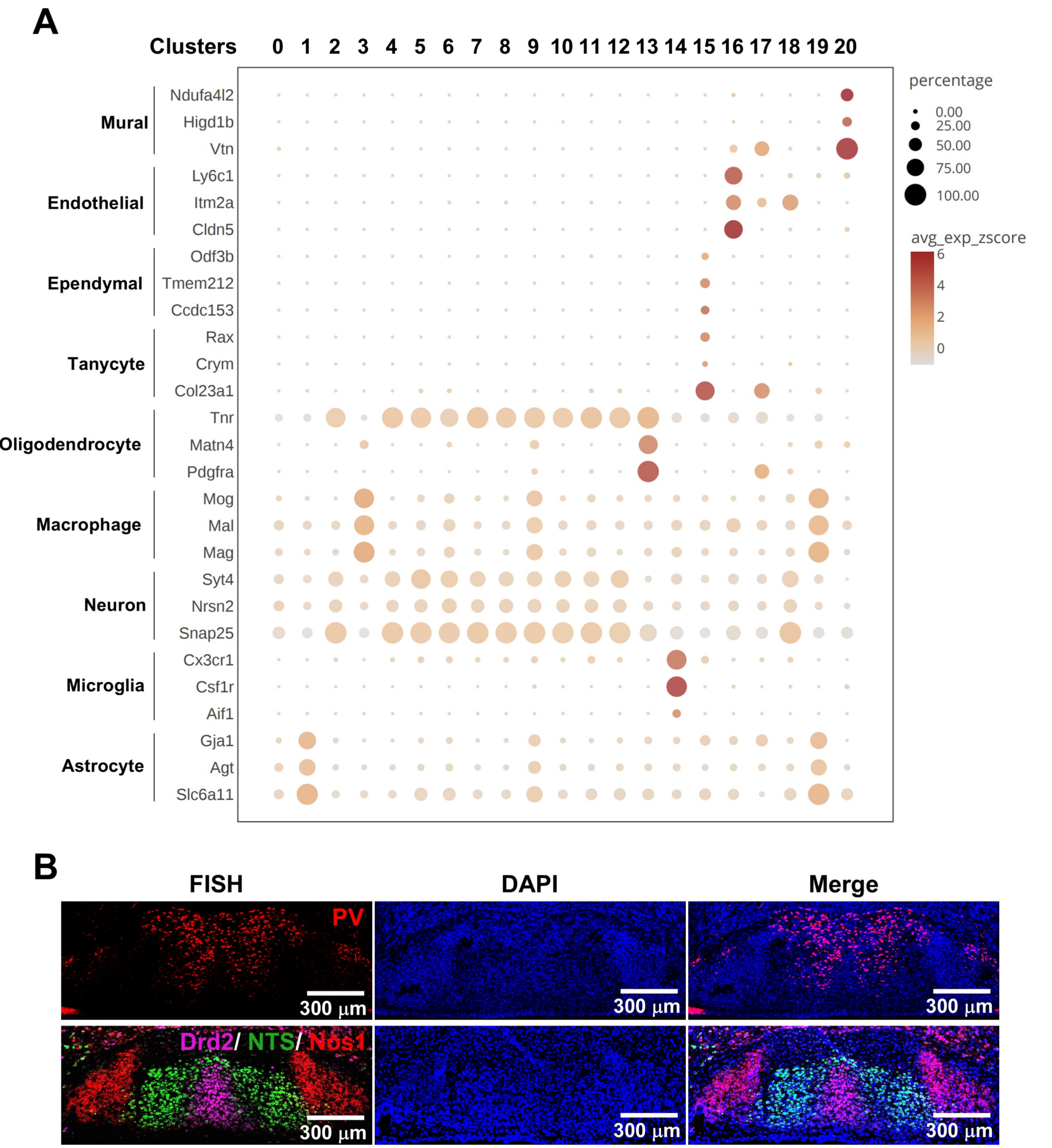

Figure S1. Molecular defined cell types in the mammillary nucleus of mice

(A) Summary of the representative marker genes enriched in each of the clusters in **Figure 1B**. Color of dots illustrate the gene expression levels.

(B) Representative merged images of FISH and DAPI showing the PV (red, upper), Drd2 (purple, bottom), NTS (green, bottom), and Nos1 (red, bottom) neurons of MM. The neuronal numbers in **Figure 1E** were quantified on a per cell basis, where cells were delineated by DAPI staining.

Figure S2. The excitatory glutamatergic PV and Drd2 neurons

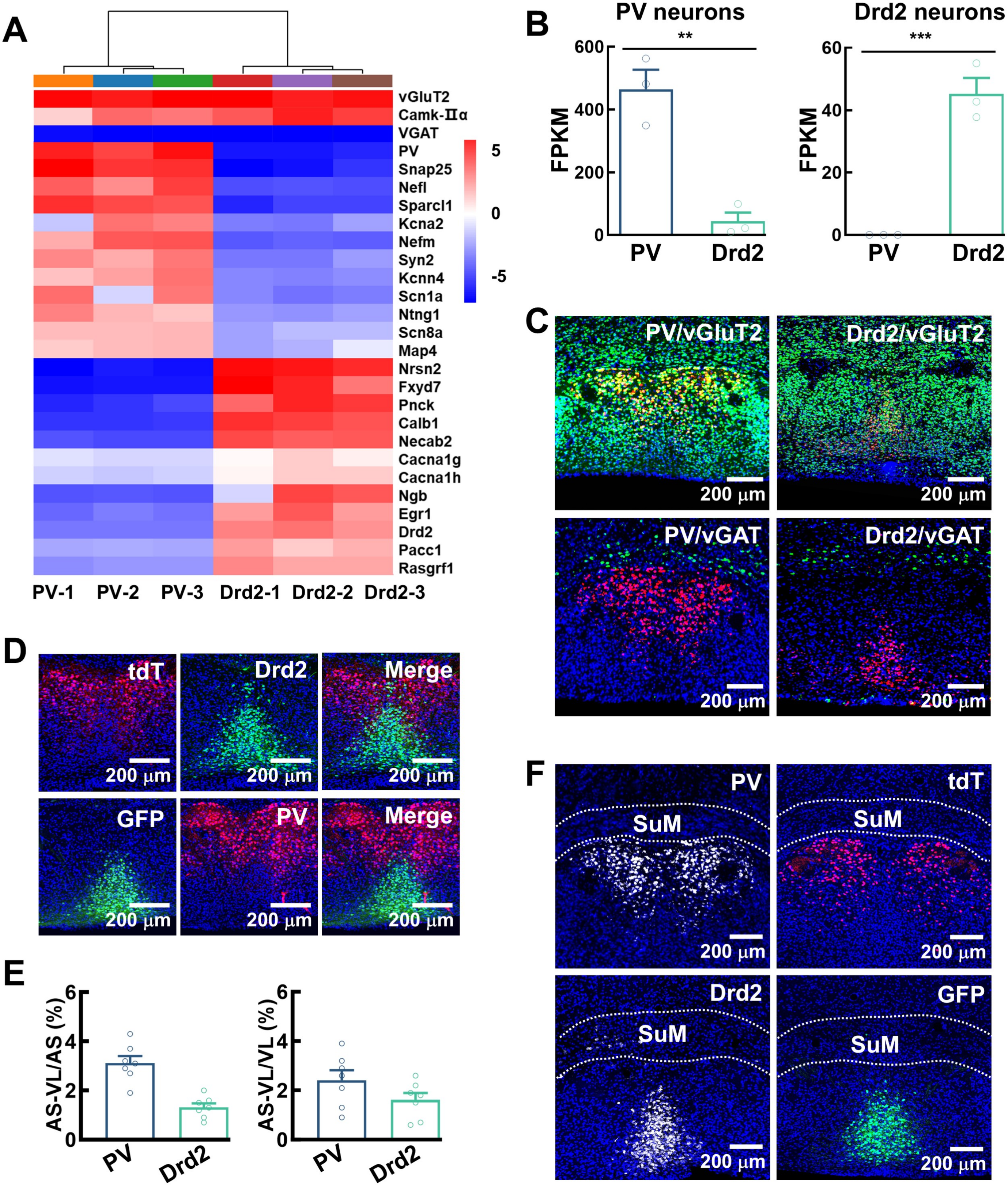

Figure S2. The excitatory glutamatergic PV and Drd2 neurons

(A) Heat map plot showing the genes enriched in PV versus Drd2 neurons (n = 3 biological replicates).

(B) Bar graphs showing the expression levels of *PV* and *Drd2* in the PV versus Drd2 neurons. Data are mean ± SEM (n = 3 per group, $^{**}P < 0.01$, $^{***}P < 0.001$, *t*-test). FPKM: fragments per kilobase of transcript per million fragments mapped reads.

(C) Both PV and Drd2 neurons are glutamatergic neuronal types. Representative merged images of FISH and DAPI showing the PV (red), Drd2 (red), vGluT2 (green), and vGAT (green) neurons of MM.

(**D**) Representative images showing the virus labeled PV or Drd2 neurons with the anti-body stained Drd2 or PV neurons.

(**E**) Bar graphs showing the percentage of AS-VL neurons versus a total of AS neurons and the percentage of AS-VL neurons versus a total of VL neurons. Data are mean ± SEM (n = 7 mice per group).

(F) Representative images showing there was no PV neurons and only a few Drd2 neurons in the supra-mammillary nucleus (SuM) by immunostaining (left) and the virus (right) labeled PV and Drd2 neurons were specifically located in the pars lateralis and basalis of MM.

Figure S3. The distinct electrophysiological properties of PV and Drd2 neurons

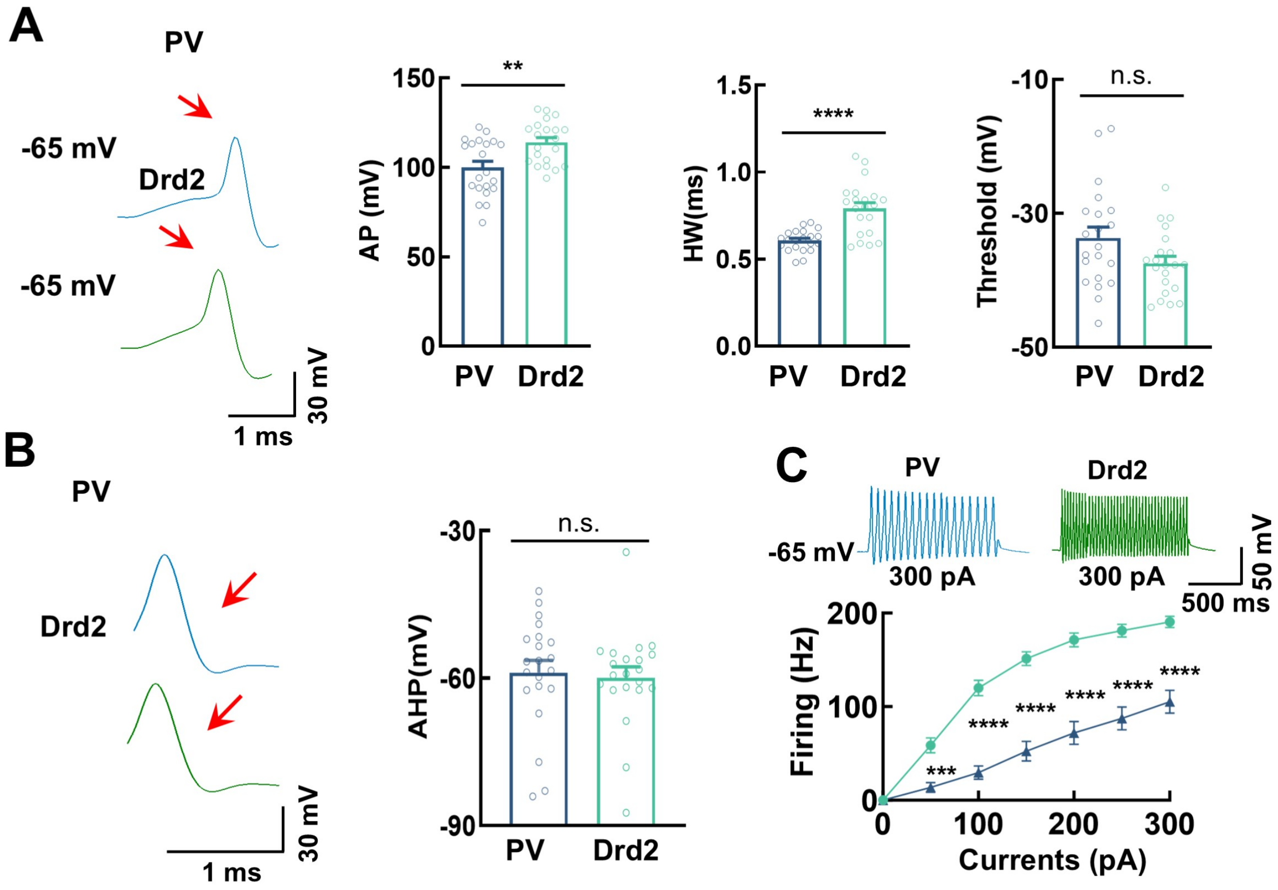

Figure S3. The distinct electrophysiological properties of PV and Drd2 neurons

(A) Representative traces showing action potentials recorded from PV and Drd2 neurons in slices of the MM. Bar graphs showing the mean amplitudes (AP), half width (HW) and threshold for action potential firing (red arrows). Data are mean ± SEM (n = 21 neurons per group, $^{**}P < 0.01$, $^{****}P < 0.0001$, *t*-test).

(**B**) Representative traces showing action potentials recorded from PV and Drd2 neurons in slices of the MM. Bar graphs showing the amplitudes of afterhyperpolarization (AHP). Data are mean ± SEM (n = 21 neurons per group, *t*-test).

(C) Representative traces recorded from PV neurons and Drd2 neurons in response to depolarizing current injection (300 pA). A plot showing the frequencies of action potential firings versus injections of the depolarizing currents. Data are mean ± SEM (n = 27 neurons per group, adjusted $^{***}P < 0.001$, $^{****}P < 0.0001$, two-way ANOVA followed with Bonferroni's post hoc test).

Figure S4. Knockdown of *Kcnn4* and *Cacna1h* in PV and Drd2 neurons

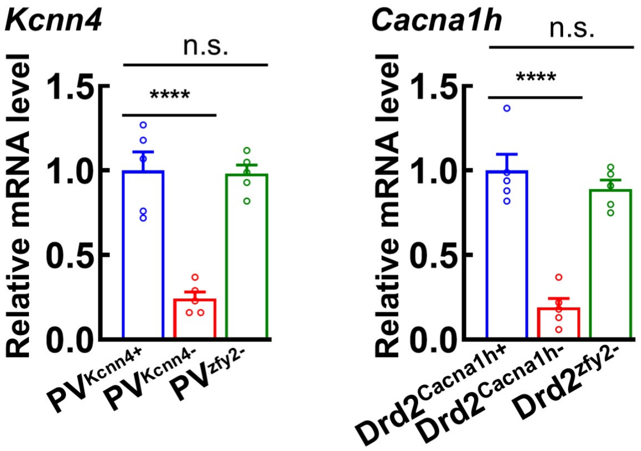

Figure S4. Knockdown of *Kcnn4* and *Cacna1h* in PV and Drd2 neurons

Relative mRNA levels of *Kcnn4* and *Cacna1h* in PV and Drd2 neurons with (PV^{Kcnn4-} and $Drd2^{Cacna1h-}$) or without (PV^{Kcnn4+} and $Drd2^{Cacna1h+}$) the expression of *Kcnn4* or *Cacna1h* sgRNAs, and zfy2 sgRNAs (PV^{zfy2-} versus $Drd2^{zfy2-}$) were used as the negative control. In this study, we injected the rAAV2/9-CaMK-IIα-fDIO-SpCas9, rAAV2/9-CaMK-IIα-fDIO-tdT with the rAAV2/9-U6-sgRNA-*Kcnn4*, or the rAAV2/9-CaMK-IIα-DIO-SpCas9, rAAV2/9-CaMK-IIα-DIO-GFP with the rAAV2/9-U6-sgRNA-*Cacna1h* into the MM of PV-FLP or Drd2-CRE mice at 3 months old of age. 12 days after the injection, the cellular RNA were prepared from the purified PV neurons expressing tdT (PV^{tdT}) and Drd2 neurons expressing GFP ($Drd2^{GFP}$) and analyzed by qPCR. Relative mRNA levels were normalized to the respective control (defined as 1.0). Data are mean ± SEM (n = 5 mice per group, adjusted ****$P < 0.0001$, one-way ANOVA followed with Bonferroni's post hoc test).

The sequences of sgRNAs and primers are listed in **Table S4, Supporting Information**.

Figure S5. Behavior performance of PV^{Kcnn4-} and Drd2$^{Cacna1h-}$ mice in home cage, open field, elevated plus maze and Morris water maze.

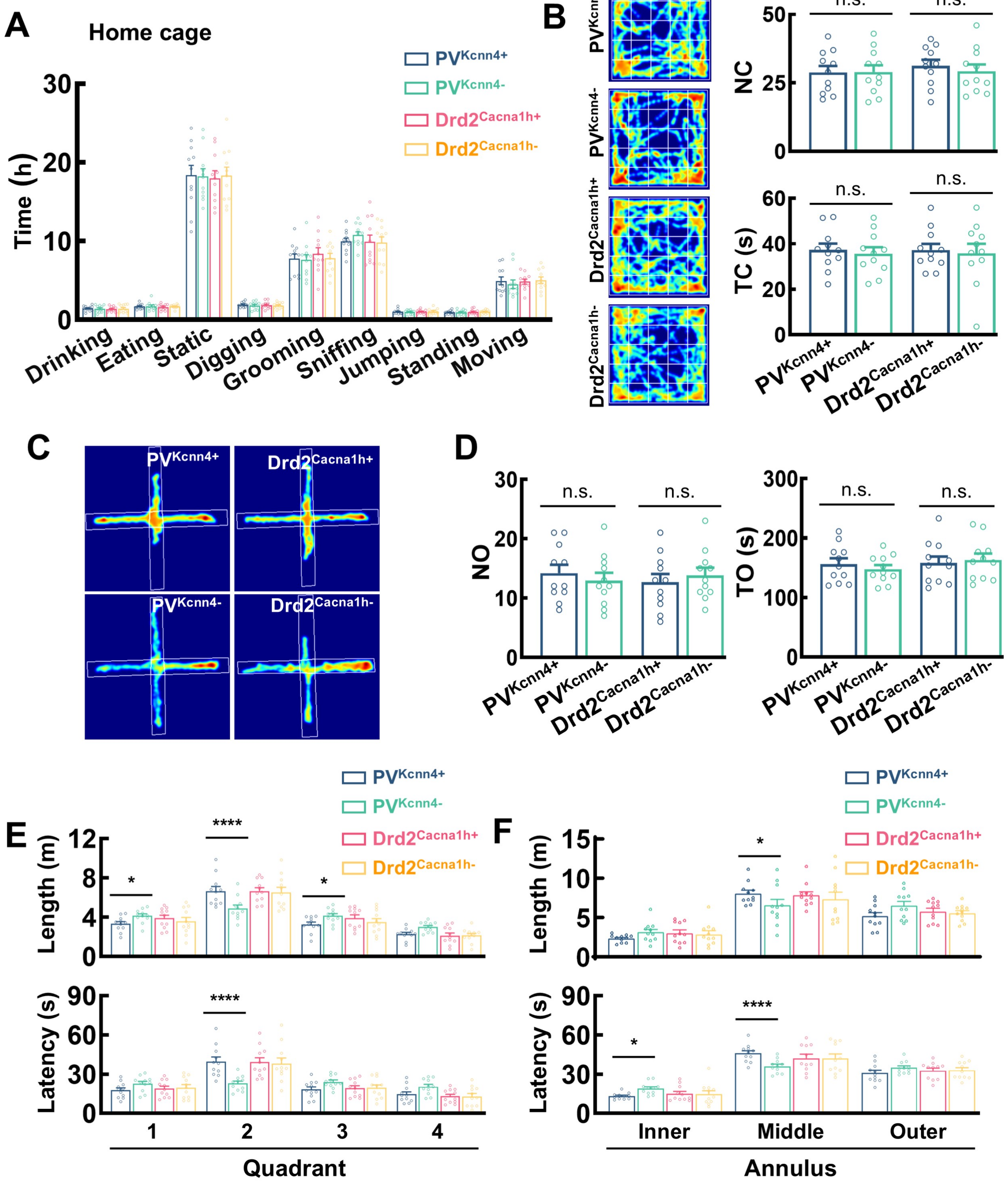

Figure S5. Behavior performance of PV^{Kcnn4-} and $Drd2^{Cacna1h-}$ mice in home cage, open field, elevated plus maze and Morris water maze.

(A) Home cage activities of PV^{Kcnn4+}, PV^{Kcnn4-}, $Drd2^{Cacna1h+}$, and $Drd2^{Cacna1h-}$ mice. Data are mean ± SEM (n = 11 mice per group, *t*-test).

(B) Heat maps and bar graphs showing the number of entrance (NC), and time spent (TC) in the center arena of the open field (OF). Data are mean ± SEM (n = 11 mice per group, t-test).

(C and **D)** Heat maps (**C**) and bar graphs (**D**) showing the number of entrance (NO) and time spent (TO) in open arm of the elevated plus maze (EPM). Data are mean ± SEM (n = 11 mice per group, *t*-test).

(E and **F)** The latency and length of PV^{Kcnn4+}, PV^{Kcnn4-}, $Drd2^{Cacna1h+}$, and $Drd2^{Cacna1h-}$ mice traveled in the individual quadrants (**E**) and annuli (**F**) in the testing session of Morris water maze. Data are mean ± SEM (n = 11 mice per group, adjusted $*P < 0.05$, $****P < 0.0001$, two-way ANOVA followed with Bonferroni's post hoc test).

Figure S6. Normal exploratory behaviors of PV^{Kcnn4-} and Drd2$^{Cacna1h-}$ mice

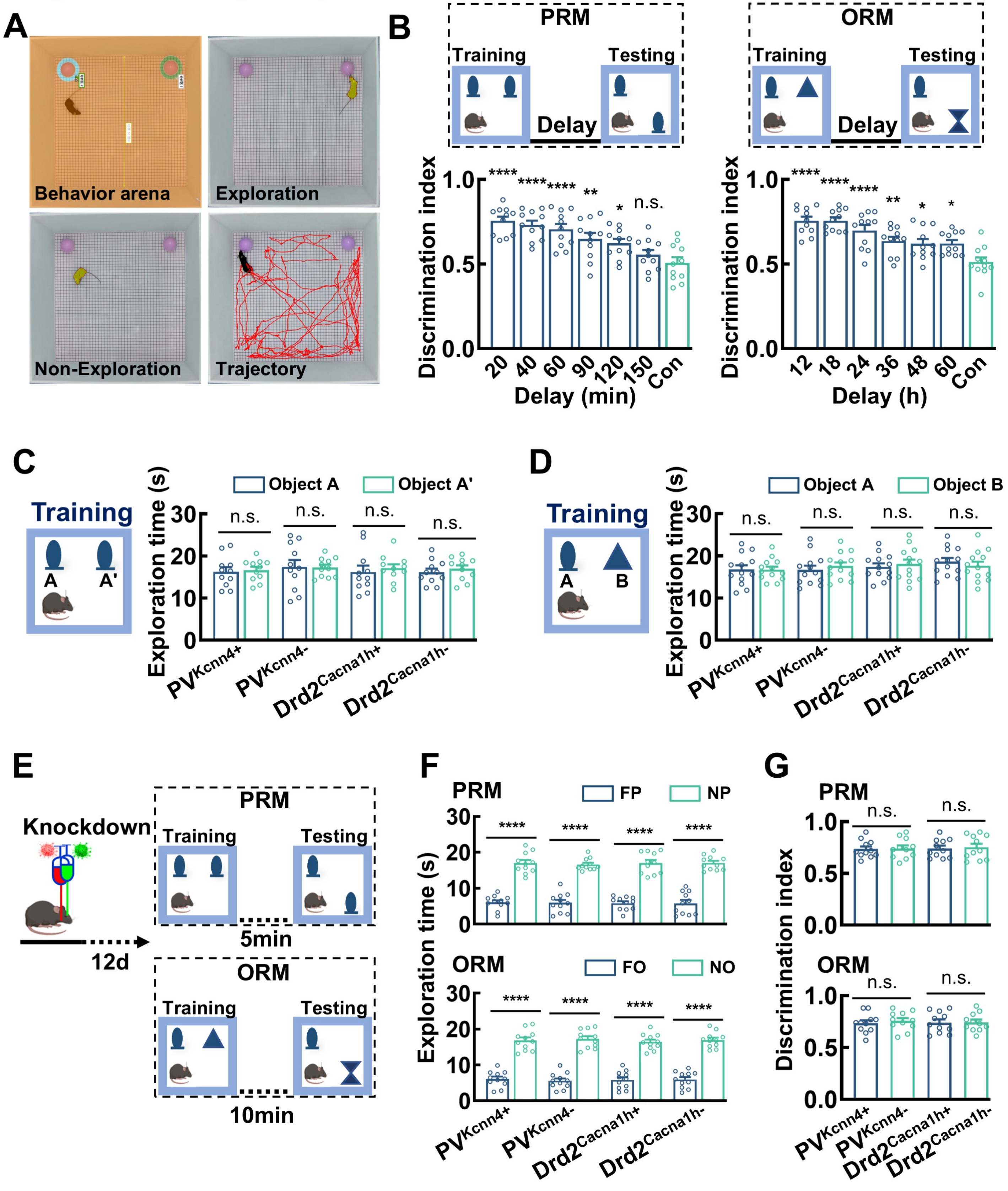

Figure S6. Normal exploratory behaviors of PV^{Kcnn4-} and Drd2$^{Cacna1h-}$ mice

(**A**) Behavioral analysis screenshots showing the setting of the behavioral arena (upper, left), exploration (upper, right), non-exploration (bottom, left), and the moving trajectory (bottom, right).

(B) Experimental procedures for PRM and ORM tasks with different delays(left). Bar graphs showing the discrimination index of C57 mice for PRM and ORM with different delays (20, 40, 60, 90, 120, and 150 minutes for PRM task and 12, 18, 24, 36, 48, and 60 hours for ORM task). Mice performed only testing session were regarded as control. Data are mean ± SEM (n = 11 mice per group, **P* < 0.05, ***P* < 0.01, *****P* < 0.0001, one-way ANOVA followed with Bonferroni's post hoc test).

(C and **D)** Experimental procedures and bar graphs showing the exploration time of each object in the training session of PRM (**C**) and ORM (**D**) tasks. In the training session, a mouse was subjected to explore two copies of a single object (A versus A') in PRM task or two different objects (A versus B) in ORM task. Data are mean ± SEM (n = 11 mice per group in PRM, n = 13 mice per group in ORM, *t*-test).

(E) Experimental procedures for gene knockdown and the behaviors.

(F and **G)** Bar graphs showing the exploration time (**F**) and discrimination index (**G**) for PRM and ORM tasks with shorter delays (5 minutes for PRM task and 10 minutes for ORM task) after knockdown of *Kcnn4* in PV neurons (PV^{Kcnn4-} mice) or *Cacna1h* in Drd2 neurons (Drd2$^{Cacna1h-}$ mice). Data are mean ± SEM (n = 11 mice per group, *t*-test).

Figure S7. Two distinct cell-type specific DS→PV and VS→Drd2 subcircuits

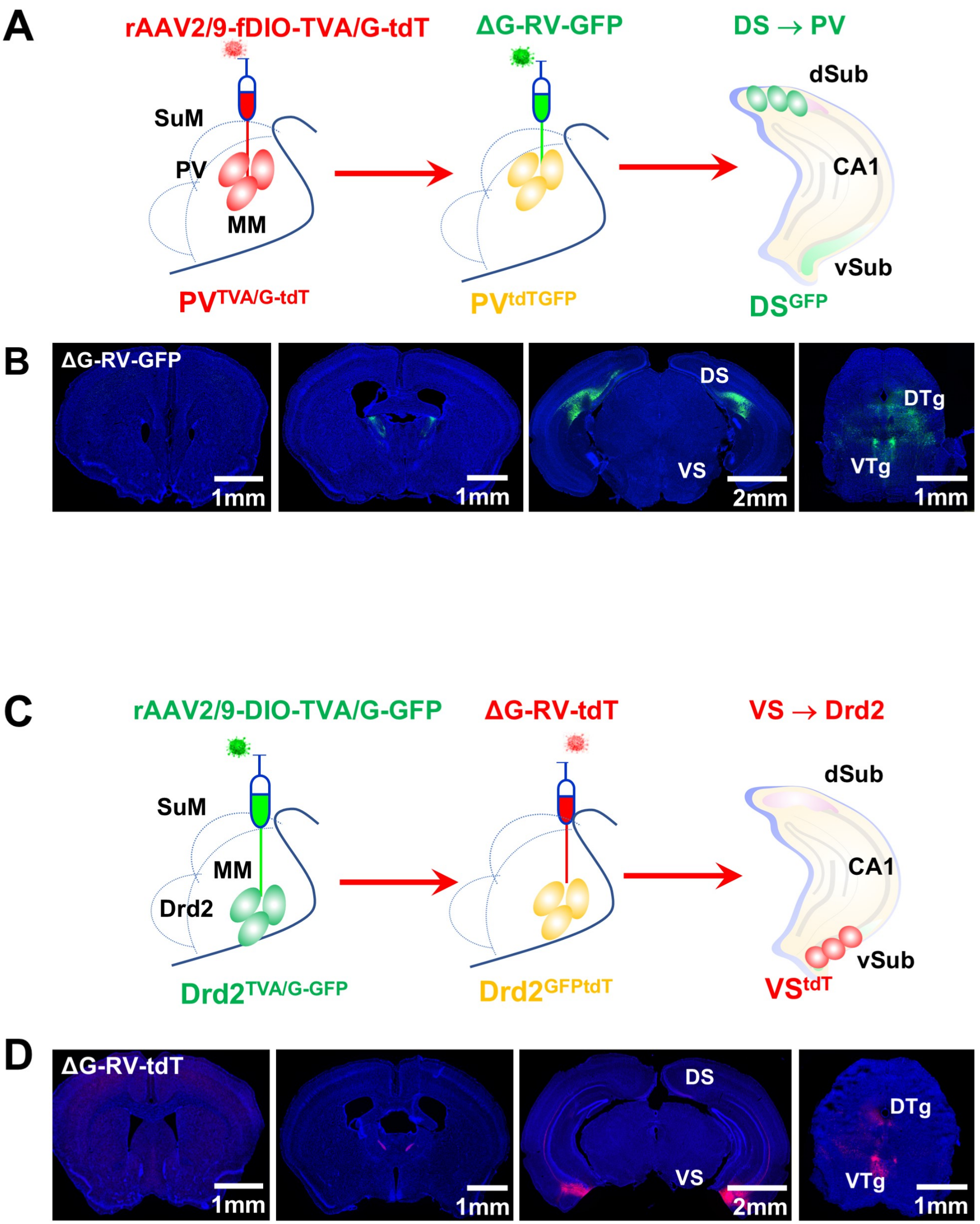

Figure S7. Two distinct cell-type specific DS→PV and VS→Drd2 circuits.

(A) Illustrations showing the labeling of PV presynaptic neurons. In this study, 0.1 μl of AAV2/9-CaMK-IIα-fDIO-TVA/G-tdT virus was injected into the MM of PV^{FLP} mice, resulting in the expression of TVA/G in PV neurons ($PV^{TVA/G}$, red). 12 days after the rAAV2/9 virus injection, 0.2 μl of ΔG-RV-GFP virus was then injected into the same location, leading to the expression of GFP in $PV^{TVA/G}$ neurons ($PV^{TVA/G\text{-}GFP}$, yellow) and their presynaptic DS (DS^{GFP}) neurons.

(B) Representative images showing $PV^{TVA/G}$ presynaptic neurons from anterior to posterior sections of $PV^{TVA/G}$ mice 12 days after the injection of the ΔG-RV-GFP virus. Note: $PV^{TVA/G}$ presynaptic neurons are mainly distributed in the dorsal subiculum (DS).

(C) Illustrations showing the labeling of Drd2 presynaptic neurons. In this study, 0.1 μl of AAV2/9-CaMK-IIα-DIO-TVA/G-GFP virus was injected into the MM of $Drd2^{CRE}$ mice, resulting in the expression of TVA/G in Drd2 neurons ($Drd2^{TVA/G}$, green). 12 days after the rAAV2/9 virus injection, 0.2 μl of ΔG-RV-tdT virus was then injected into the same location, leading to the expression of tdT in $Drd2^{TVA/G}$ neurons ($Drd2^{TVA/G\text{-}tdT}$, yellow) and their presynaptic VS (VS^{tdT}) neurons.

(D) Representative images showing $Drd2^{TVA/G}$ presynaptic neurons from anterior to posterior sections of $Drd2^{TVA/G}$ mice 12 days after the injection of the ΔG-RV-tdT virus. Note: $Drd2^{TVA/G}$ presynaptic neurons are mainly distributed in the ventral subiculum (VS).

Figure S8. The projections from PV and Drd2 neurons to AVT and AMT neurons

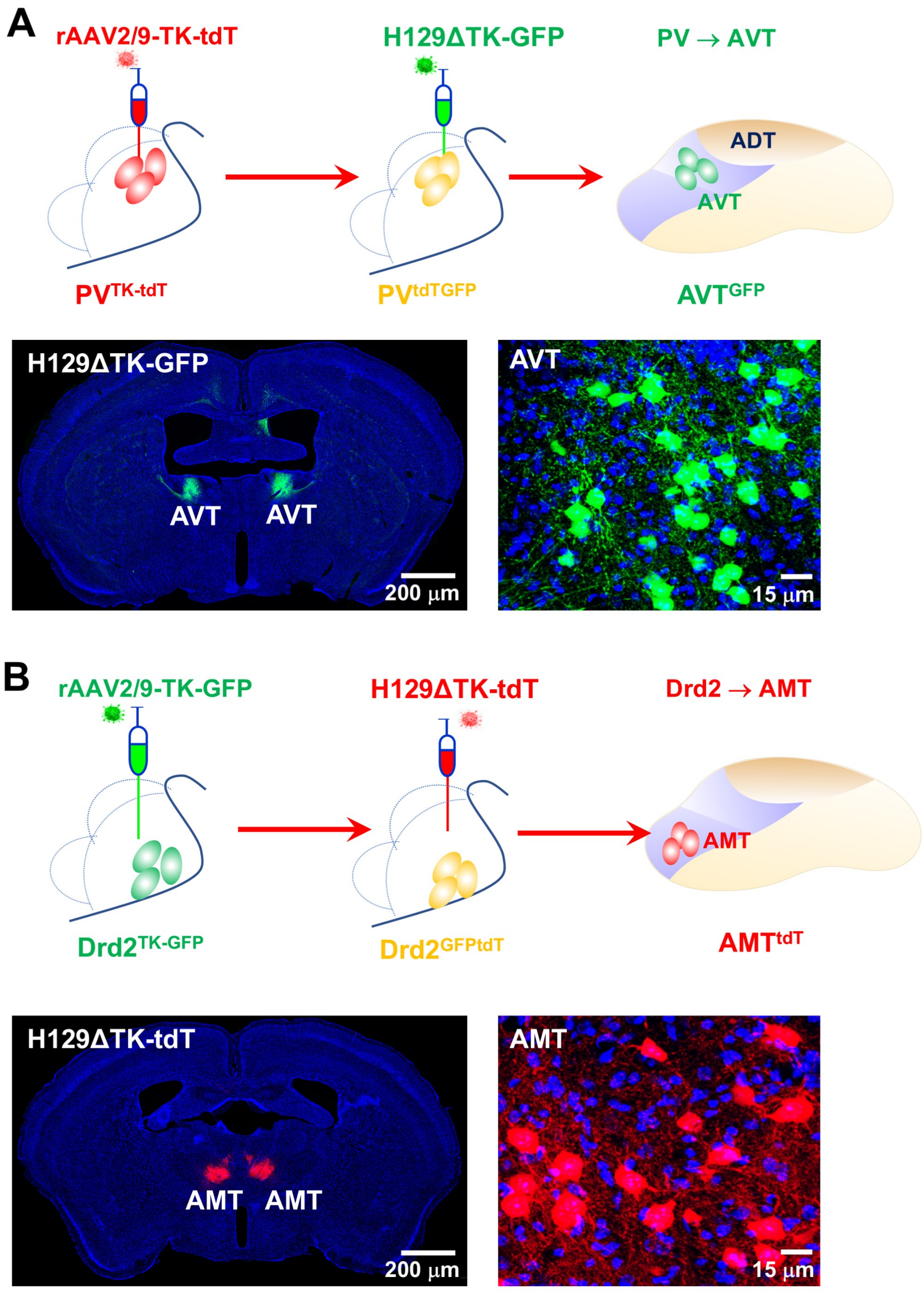

Figure S8. The projections from PV and Drd2 neurons to AVT and AMT neurons

(A) PV neurons project onto AVT neurons only. Illustrations showing the labeling of PV targeting neurons. In this study, 0.1 μl of AAV2/9-CaMK-IIα-fDIO-TK-tdT virus was injected into the MM of PV^{FLP} mice, resulting in the expression of TK in PV neurons (PV^{TK}, red). 12 days after the injection, 0.3 μl of H129ΔTK-GFP virus was then injected into the same location, leading to the expression of GFP in PV^{TK} neurons ($PV^{TK\text{-}GFP}$, yellow) and their targeting neurons in the anterior ventral thalamus (AVT^{GFP}) Representative low and high magnifications of the images showing PV^{TK} targeting AVT. ADT: anterior-dorsal thalamic nucleus.

(B) Drd2 neurons project onto AMT only. Illustrations showing the labeling of Drd2 targeting neurons. In this study, 0.1 μl of AAV2/9-CaMK-IIα-DIO-TK-GFP virus was injected into the MM of $Drd2^{CRE}$ mice, resulting in the expression of TK in Drd2 neurons ($Drd2^{TK}$, green). 12 days after the injection, 0.3 μl of H129ΔTK-tdT virus was then injected into the same location, leading to the expression of tdT in $Drd2^{TK}$ neurons ($Drd2^{TK\text{-}tdT}$, yellow) and their targeting neurons in the anterior medial thalamus (AMT^{tdT}). Representative low and high magnifications of the images showing $Drd2^{TK}$ targeting AMT.

Figure S9. Normal exploratory behaviors with DS and VS terminal inhibition

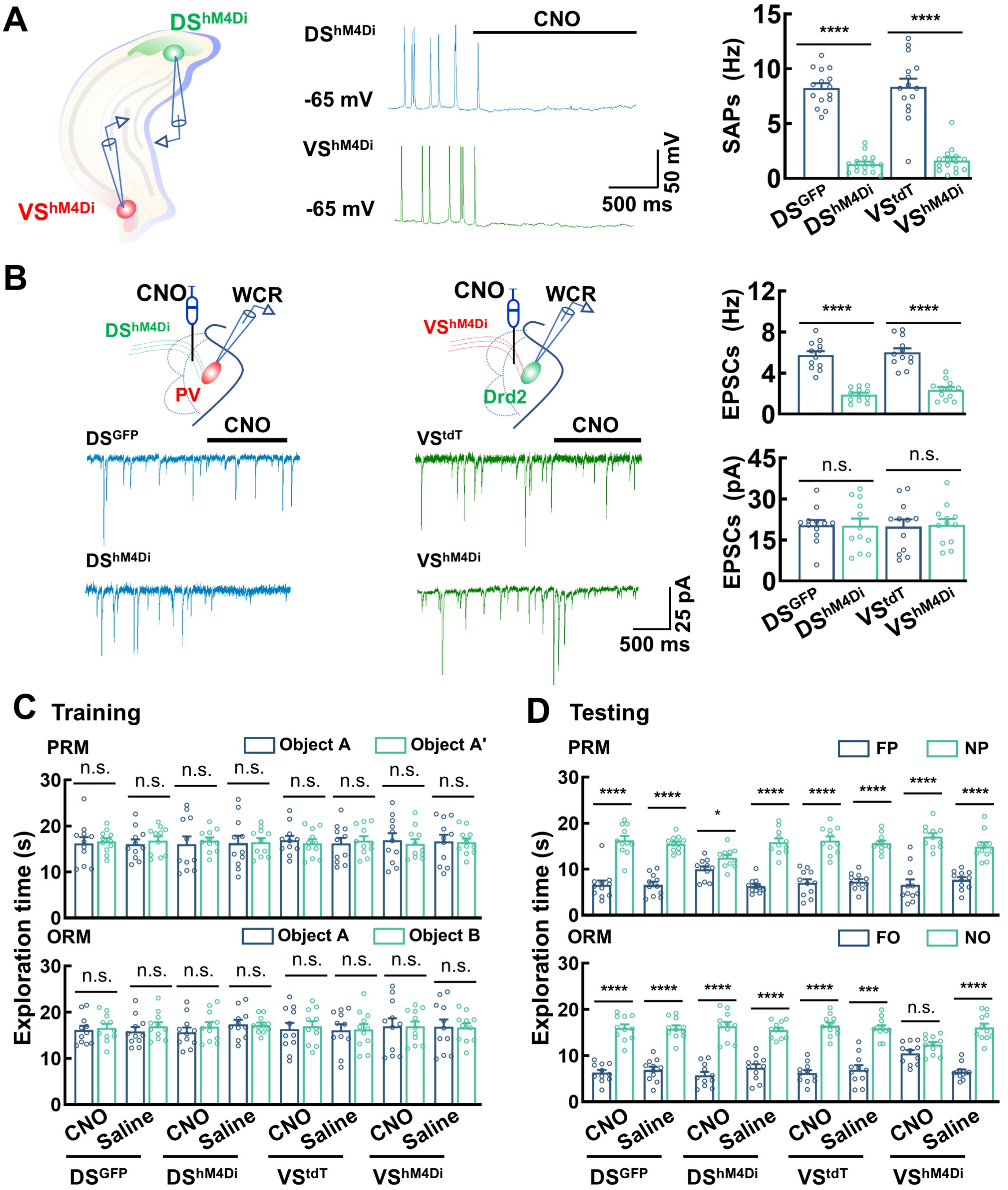

Figure S9. Normal exploratory behaviors with DS and VS terminal inhibition

(A) Representative traces and a bar graph showing spontaneous action potentials intracellularly record from DS^{hM4Di} or VS^{hM4Di} neurons. Bath application of 100 μM CNO effectively inhibits spontaneous action potential firings in DS^{hM4Di} or VS^{hM4Di} neurons. Data are mean ± SEM (n = 15 neurons per group, $^{****}P < 0.0001$, *t*-test).

(B) Representative traces and bar graphs showing the frequency and amplitude of spontaneous miniature excitatory postsynaptic currents (EPSCs) record from PV or Drd2 neurons. Bath application of 100 μM CNO effectively reduces the frequency of EPSCs. Data are mean ± SEM (n = 12 neurons per group, $^{****}P < 0.0001$, *t*-test).

(C and **D)** Bar graphs showing the exploration time in training (**C**) and testing (**D**) sessions of DS^{GFP}, DS^{hM4Di}, VS^{tdT}, and VS^{hM4Di} mice in PRM and ORM tasks with the application of CNO or saline. Data are mean ± SEM (n = 11 mice per group, $P < 0.05$, $^{***}P < 0.001$, $^{****}P < 0.0001$, *t*-test).

Figure S10. Normal behavioral performance with activation of PV and Drd2 neurons during the training session.

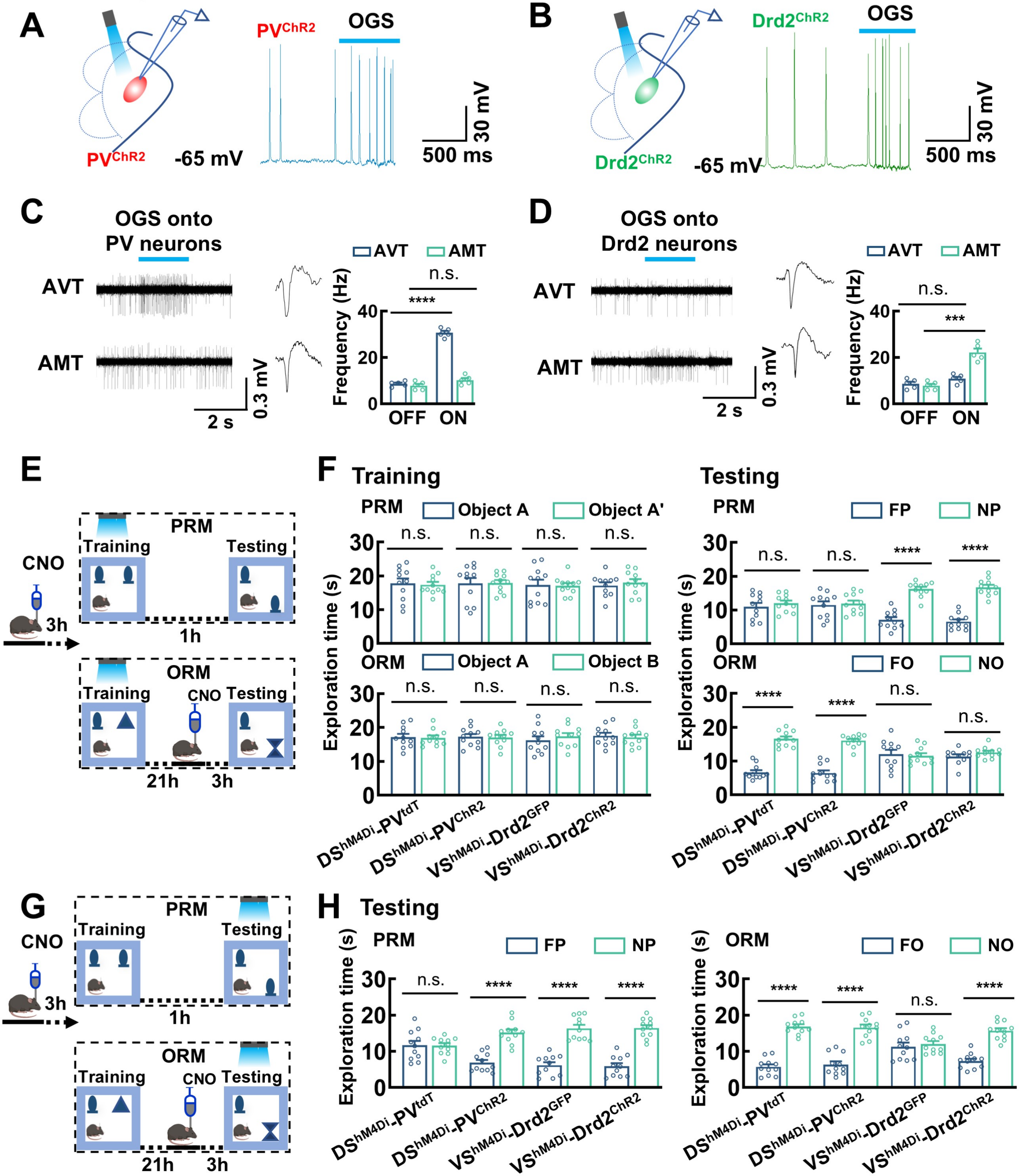

Figure S10. Normal behavioral performance with activation of PV and Drd2 neurons during the training session.

(A and **B)** Representative traces showing spontaneous action potentials intracellularly recorded from $PV^{ChR2\text{-}tdT}$ (**A**) and $Drd2^{ChR2\text{-}GFP}$ (**B**) neurons in the slices. Optogenetic stimulation (OGS) by delivery of blue lights onto $PV^{ChR2\text{-}tdT}$ and $Drd2^{ChR2\text{-}GFP}$ neurons effectively induces action potential firings. The similar recordings were seen in each of the 12 recordings from 6 mice per group.

(C and **D)** Representative traces recorded from AVT and AMT in vivo, waveforms of neuronal firing, and bar graphs (the frequency of firing) showing the excitatory response of AVT and AMT to the optogenetic stimulation (OGS) onto $PV^{ChR2\text{-}tdT}$ (**C**) and $Drd2^{ChR2\text{-}GFP}$ (**D**) neurons, respectively. Data are mean ± SEM (n = 5 mice per group, ***P < 0.001, ****P < 0.0001, *t*-test).

(E and **F)** Experimental procedures (**E**) and bar graphs (**F**) showing the exploration time in the training and testing sessions of DS^{hM4Di}-PV^{tdT}, DS^{hM4Di}-PV^{ChR2}, VS^{hM4Di}-$Drd2^{GFP}$, and VS^{hM4Di}-$Drd2^{ChR2}$ mice in PRM and ORM tasks with the application of CNO in both training and testing sessions and the optogenetic stimulation only in the training session. Data are mean ± SEM (n = 11 mice per group, ****P < 0.0001, *t*-test).

(G and **H)** Experimental procedures (**G**) and bar graphs (**H**) showing the exploration time in the testing session of DS^{hM4Di}-PV^{tdT}, DS^{hM4Di}-PV^{ChR2}, VS^{hM4Di}-$Drd2^{GFP}$, and VS^{hM4Di}-$Drd2^{ChR2}$ mice in PRM and ORM tasks with the application of CNO in both training and testing sessions and the optogenetic stimulation only in the testing session. Data are mean ± SEM (n = 11 mice per group, ****P < 0.0001, *t*-test).

Figure S11. Chemogenetic inhibition of PV→ AVT and Drd2→AMT synaptic transmission

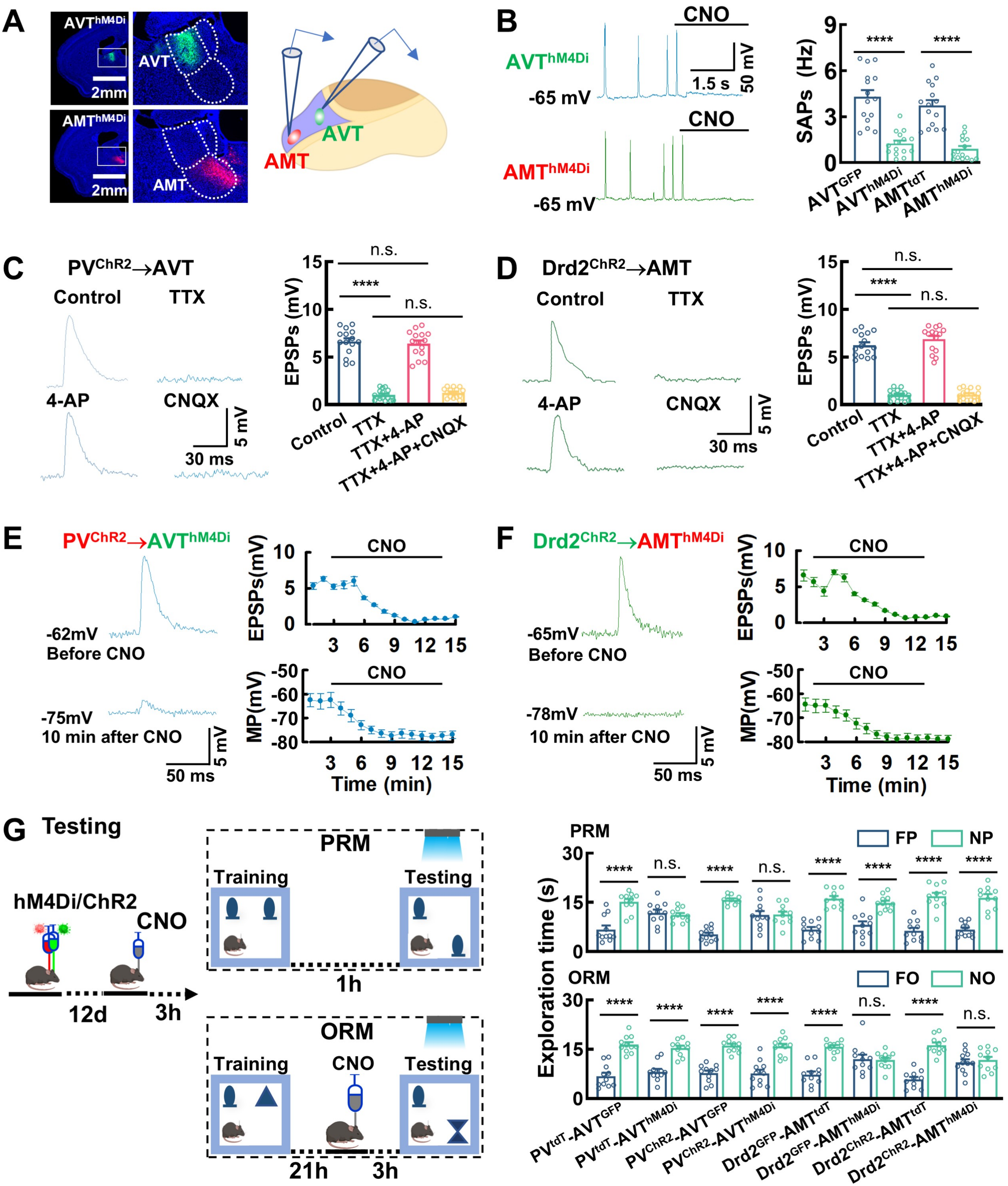

Figure S11. Chemogenetic inhibition of PV→ AVT and Drd2→AMT synaptic transmission

(A and B) Representative images (**A**) showing the expression of hM4Di-GFP or hM4Di-tdT in AVT or AMT neurons. Representative traces and a bar graph (**B**) showing that bath application of 100 μM CNO inhibits spontaneous action potential firings intracellularly recorded from AVT^{hM4Di} or AMT^{hM4Di} neurons, respectively. Data are mean ± SEM (n = 15 neurons per group, ****P < 0.0001, *t*-test).

(C and D) Representative EPSPs intracellularly recorded from AVT^{hM4Di} or AMT^{hM4Di} neurons and evoked by delivery of blue lights onto axon terminals of PV^{ChR2} or $Drd2^{ChR2}$ neurons. The evoked EPSPs were blocked by TTX, reversed by 4-AP and were sensitive to CNQX, showing excitatory mono-synaptic transmission. Bar graphs showing the mean amplitudes of EPSPs. Data are mean ± SEM (n = 15 neurons per group, ****$P < 0.0001$, BF ANOVA followed with Bonferroni's post hoc test).

(**E** and **F**) Representative traces of EPSPs intracellularly recorded from AVT^{hM4Di} (**E**) or AMT^{hM4Di} (**F**) neurons before and 10 min after application of 100 μM CNO. Graphs showing the mean amplitudes of EPSPs (top) and the membrane potentials (MP, bottom,) intracellularly recorded from AVT^{hM4Di} (**E**) or AMT^{hM4Di} (**F**) neurons. Data are mean ± SEM (n = 18 neurons per group), indicating that application of CNO reduces the membrane potentials of AVT^{hM4Di} or AMT^{hM4Di} neurons and in turn changes driving force of the recording neurons, resulting in the reduction of the mean amplitudes of the evoked EPSPs.

(G and H) Experimental procedures **(G)** and bar graph **(H)** showing the exploration time in the testing session of PV^{tdT}-AVT^{GFP}, PV^{tdT}-AVT^{hM4Di}, PV^{ChR2}-AVT^{GFP}, PV^{ChR2}-AVT^{hM4Di}, $Drd2^{GFP}$-AMT^{tdT}, $Drd2^{GFP}$-AMT^{hM4Di}, $Drd2^{ChR2}$-AMT^{tdT}, and $Drd2^{ChR2}$-AMT^{hM4Di} mice in PRM and ORM tasks with the application of CNO in both training and testing sessions and the optogenetic stimulation only in the testing session. Data are mean ± SEM (n = 11 mice per group, ****P < 0.0001, *t*-test).

Figure S12. Ca^{2+} activity of PV and Drd2 neurons in the training session of SRM tasks

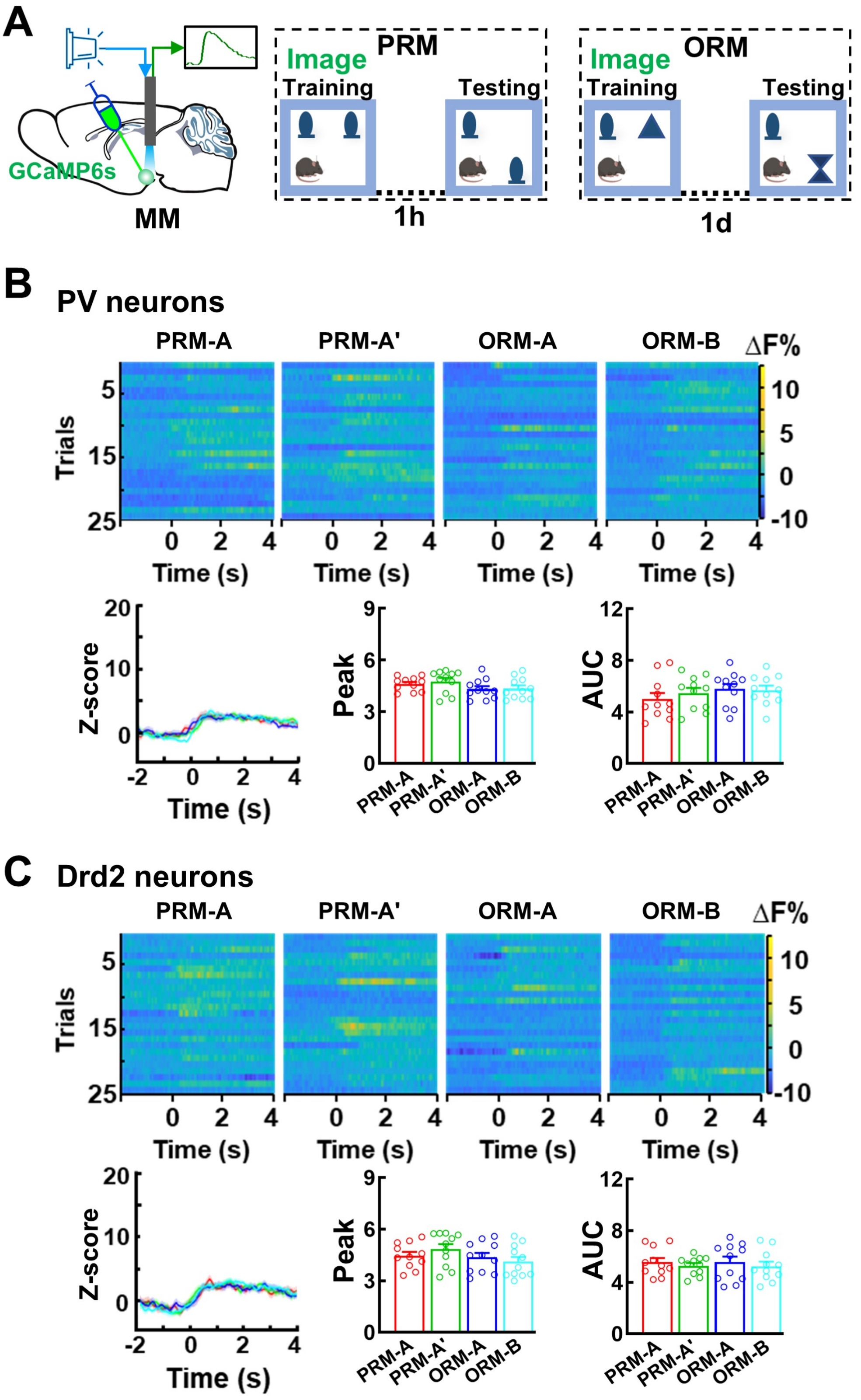

Figure S12. Ca^{2+} activity of PV and Drd2 neurons in the training session of SRM tasks

(A) Experimental procedures for calcium photometry in vivo and schematic diagram of the viral vector and optical fiber placement.

(**B** and **C**) Heat maps (top) and example Z-scores (bottom, left) showing calcium signals of $PV^{GCaMP6s}$ (**B**) and $Drd2^{GCaMP6s}$ (**C**) neurons during exploration with objects in the PRM (A versus A') and ORM (A versus B) tasks. Time 0 s indicates start of exploration events. The bar graphs show the mean peak (bottom, middle) and AUC (bottom, right) during the exploration period. Data are mean ± SEM (n = 11 mice per group, one-way ANOVA followed with Bonferroni's post hoc test).

Figure S13. Low Ca^{2+} activity of PV^{Kcnn4-} and $Drd2^{Cacna1h-}$ neurons during exploring novel place and novel object

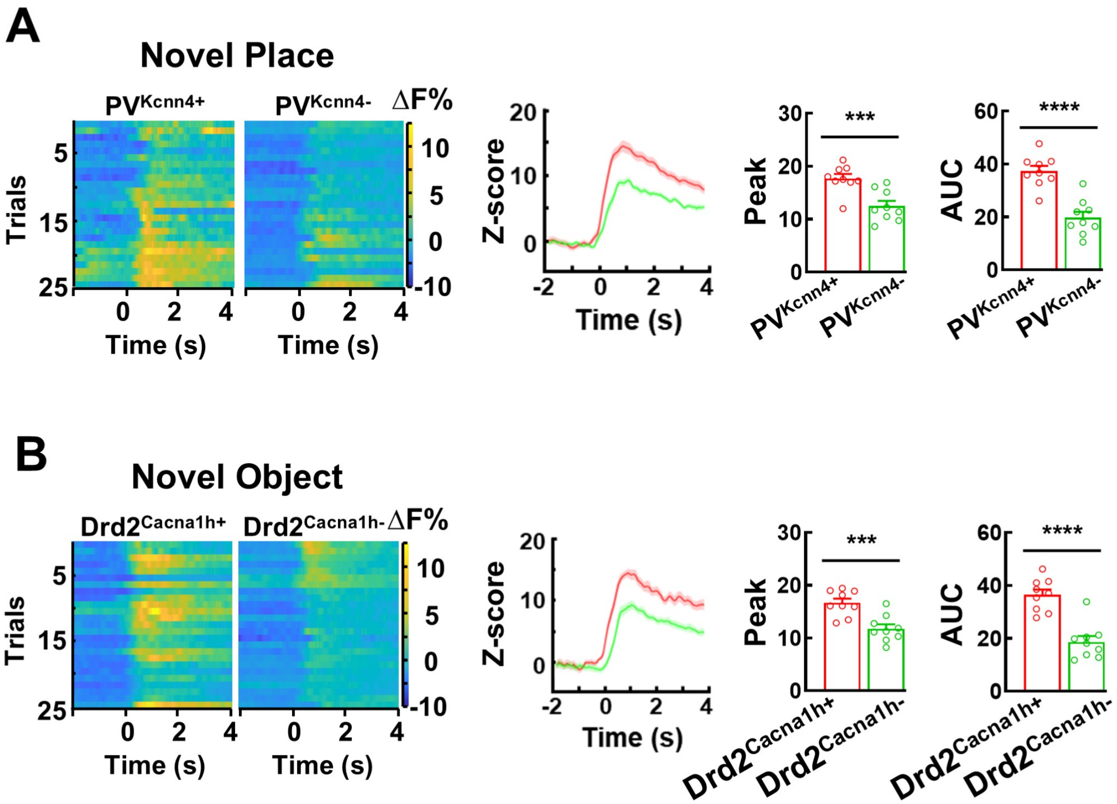

Figure S13. Low Ca^{2+} activity of PV^{Kcnn4-} and $Drd2^{Cacna1h-}$ neurons during exploring novel place and novel object

(**A**) Heat maps and example Z-scores showing calcium signals of PV^{Kcnn4-} and PV^{Kcnn4+} neurons during exploring the novel location in the testing phase of the PRM tasks. Time 0 s indicates start of exploration events. The bar graphs show the mean peak and AUC during the exploration period. Data are mean ± SEM (n=9 mice per group, ****P* < 0.001 , *****P* < 0.0001, *t*-test).

(**B**) Heat maps and example Z-scores showing calcium signals of $Drd2^{Cacna1h+}$ and $Drd2^{Cacna1h-}$ neurons during exploring the novel object in the testing phase of the ORM tasks. Time 0 s indicates start of exploration events. The bar graphs show the mean peak and AUC during the exploration period. Data are mean ± SEM (n=9 mice per group, ****P* < 0.001 , *****P* < 0.0001, *t*-test).

Table S1. Enriched genes in PV neurons

Gene name	Gene ID	PV-1 FPKM	PV-2 FPKM	PV-3 FPKM	Drd2-1 FPKM	Drd2-2 FPKM	Drd2-3 FPKM	log2F.C.	P value
Nefh	ENSMUSG00000020396	79.57207665	74.8815509	82.92530081	12.45203179	14.04647168	11.69035919	2.635968	1.07E-05
Gm39653	ENSMUSG00000115378	62.81069301	62.40166889	69.22780306	7.885720449	0	0	4.62394	5.47E-05
Ubxn2a	ENSMUSG00000020634	37.01909969	36.23567815	36.78147928	18.67646354	15.70034132	20.4398816	1.005292	0.000195
Megf11	ENSMUSG00000036466	3.348258034	3.653713685	3.57697317	0.214714165	0.944521473	0.360189739	2.799598	0.000234
Mfsd14b	ENSMUSG00000038212	4.918725293	5.459528951	5.859966435	1.998763905	1.517854364	2.063032155	1.541147	0.000386
Slc24a2	ENSMUSG00000037996	34.28970025	34.21932658	42.10994601	5.868506896	8.75333762	6.864184871	2.364128	0.000417
Nrp2	ENSMUSG00000025969	14.45842483	14.77209074	13.97981113	3.386328335	1.457262953	5.296108666	2.091361	0.000622
Oip5os1	ENSMUSG00000085438	21.56674993	16.69470463	18.21157032	4.463974527	4.704779852	5.161782733	1.978469	0.000642
Nrip3	ENSMUSG00000034825	58.83019378	74.75266288	76.20638389	15.5801624	11.49233791	23.12943104	2.063126	0.001233
Fbxo38	ENSMUSG00000042211	8.253184921	6.381313215	8.714344828	2.081914197	2.11081951	2.502539157	1.802136	0.001573
Gm30382	ENSMUSG00000106515	9.50777729	7.131199026	6.519578395	0.863750779	0	0.74312932	3.849211	0.001643
Scn8a	ENSMUSG00000023033	19.09838721	19.35428101	20.47506154	6.946231374	10.73645465	10.90413409	1.043597	0.001744
Nacc2	ENSMUSG00000026932	14.29389619	11.54096475	13.62440065	5.593960079	7.106239472	5.56660654	1.11114	0.001908
A930029G22Rik	ENSMUSG00000096988	6.826820847	10.05456756	9.487351623	0	0	1.643688224	4.00382	0.001915
AW047730	ENSMUSG00000097428	5.072810479	4.453775606	3.268359606	0.551627895	0.322940035	0.17892956	3.602315	0.001929
Ppp2r2b	ENSMUSG00000024500	51.81899195	56.93327032	57.2594716	29.68257926	15.24953417	16.87391015	1.425466	0.002079
Atp2b1	ENSMUSG00000019943	11.10544475	14.86067986	13.8542049	5.151389891	5.56044255	4.504382099	1.387896	0.002136
Tmem41b	ENSMUSG00000047554	12.72241455	10.01430716	13.22557441	3.911758009	4.521739051	5.222423434	1.396959	0.002227
Hsph1	ENSMUSG00000029657	26.83277473	31.25364114	30.86088709	9.10855855	16.23084348	10.6118442	1.306909	0.002406
Arglu1	ENSMUSG00000040459	25.00147504	19.51438094	23.40051727	7.41368537	10.68107567	5.93607956	1.498872	0.002435
Slc35f1	ENSMUSG00000038602	6.897857632	7.82937638	8.829540873	3.245179591	2.798120827	4.168764767	1.205867	0.002955
Gm10060	ENSMUSG00000110669	38.38098486	28.310584	40.03417119	11.21457027	11.70509248	12.47187394	1.592432	0.00296
Bmpr1a	ENSMUSG00000021796	5.678082835	4.339452907	5.642023241	1.992700763	1.673330748	2.483627636	1.348465	0.003156
Ktn1	ENSMUSG00000021843	11.706661	14.04868728	14.29356276	7.50832999	5.201519507	7.050289784	1.01917	0.003374
Pvalb	ENSMUSG00000005716	349.3123377	481.9176171	562.6988546	10.44892615	22.49768422	99.49725789	3.395704	0.003507
Cadm2	ENSMUSG00000064115	36.66109423	26.67511261	39.93874484	10.1766227	8.95269713	10.36689339	1.807889	0.003593
Camk2d	ENSMUSG00000053819	7.741747777	8.446774794	9.232167889	0.531023802	1.447039551	3.827108058	2.130593	0.003661
Atp2b2	ENSMUSG00000030302	17.10260964	17.78525894	25.66815678	2.707228054	3.618843701	3.668869816	2.599	0.00367
B830042I05Rik	ENSMUSG00000108847	2.546979617	2.363433598	1.850491893	0	0.760886591	0.517911712	2.402428	0.003962
Sec63	ENSMUSG00000019802	4.642247821	4.538172517	3.487887155	0.870232712	1.562031583	1.847613344	1.565582	0.003979
Ppm1e	ENSMUSG00000046442	16.79781915	18.53986526	12.00865843	4.696522616	4.443920863	2.829272399	1.983864	0.004436
Ppargc1a	ENSMUSG00000029167	15.03241181	18.84375858	19.07328996	2.814579106	7.863676069	7.140299188	1.571236	0.004644
AI593442	ENSMUSG00000078307	42.34820484	41.87063401	51.99682011	21.82550551	26.54060409	18.14772246	1.034166	0.004767
Tmem196	ENSMUSG00000048004	30.7014425	36.03054853	31.31295478	18.50563505	9.544077672	8.157109471	1.437182	0.004862
Gtf2a1	ENSMUSG00000020962	3.515272027	3.050064244	3.012009255	1.082771932	1.927020082	1.633401095	1.044509	0.005113
Ahr	ENSMUSG00000019256	2.688872154	4.864503039	3.828399277	0	0.486500805	0.183785967	4.085803	0.005179
Rnf166	ENSMUSG00000014470	27.52934926	32.40827853	24.90983083	11.88526533	16.53137359	13.75818766	1.008489	0.005271
Stk39	ENSMUSG00000027030	12.18608544	9.724442778	8.007411348	3.672662639	1.566554409	2.09119266	2.029045	0.005314
Sparcl1	ENSMUSG00000029309	286.6622735	357.6251684	262.9564212	40.86016867	116.3455107	121.6103255	1.702177	0.005587
mt-Tr	ENSMUSG00000064361	4.933174294	7.967735355	7.261099676	0.435860399	1.959676117	0	3.073219	0.005612
St8sia1	ENSMUSG00000030283	2.233738785	3.414443148	4.062582326	0.371234914	0.046364246	0.440122382	3.501004	0.005785
Efr3a	ENSMUSG00000015002	17.71597709	15.09268063	11.63333855	6.001024165	3.3551223	3.930476921	1.741949	0.00583
Thg1l	ENSMUSG00000011254	2.703674031	2.01267902	1.380314234	0	0.03314875	0	7.522923	0.006142
Ep300	ENSMUSG00000055024	3.398508806	2.462351956	2.876212637	1.150405167	0.81459808	0.035270103	2.126953	0.006266
B3galt1	ENSMUSG00000034780	13.70278554	13.33560406	18.08468371	1.128251879	2.478464006	6.504459768	2.157915	0.006281
Nrg1	ENSMUSG00000062991	5.271045429	3.814958365	6.339271848	1.55749558	1.219639027	0.964980969	2.04337	0.006594
Siah1a	ENSMUSG00000036840	10.38072225	9.268700274	6.809916363	3.283127314	3.504115983	2.259547745	1.548299	0.006648
Ece1	ENSMUSG00000057530	9.702902284	10.87019593	12.13758376	5.016177528	1.081149616	4.527525559	1.622319	0.006652
Mtx3	ENSMUSG00000021704	5.551667671	9.318460227	8.121919818	2.234571427	1.831921956	1.18954308	2.129088	0.006832
Mier1	ENSMUSG00000028522	3.52532922	2.36992317	3.153929037	0.888231453	0.867939346	1.404731528	1.517451	0.006875
Zfp369	ENSMUSG00000021514	3.254490956	4.789363419	4.5605429	1.820975602	1.777867085	1.486763795	1.309435	0.006884
Kin	ENSMUSG00000037262	10.13253746	12.13899669	9.887506655	3.128991955	5.314687604	6.17575544	1.137337	0.007141
Ptp4a2	ENSMUSG00000028788	35.65320136	60.4069751	55.67814739	13.62409786	9.034568052	12.47886795	2.110501	0.007281
Atg12	ENSMUSG00000032905	11.07165275	16.32467838	14.66344756	6.303611943	6.28184078	4.265265904	1.319631	0.007657
Gm41505	ENSMUSG00000116641	4.305016767	4.214046699	3.820721496	0	0	2.027624354	2.605455	0.007667
Ube2q2	ENSMUSG00000032307	22.02086499	21.59672713	20.20241053	1.266779399	6.732545823	11.43051	1.715735	0.007747
Syn2	ENSMUSG00000009394	33.76982677	45.51139611	54.14378236	3.743278955	4.885114962	17.16577894	2.370912	0.007968
Myo5a	ENSMUSG00000034593	12.26205239	16.63885763	18.65261943	6.665310426	6.681803192	6.512399536	1.259722	0.008092
Map3k20	ENSMUSG00000004085	2.482538148	1.572613005	2.994763974	0.273810287	0	0.42053922	3.343872	0.008135
Dlat	ENSMUSG00000000168	23.79635659	22.56231307	23.50972621	4.385722607	14.22991178	9.659170036	1.305123	0.008441
Mybbp1a	ENSMUSG00000040463	3.79794063	3.946292224	5.402968878	1.552663479	1.997836276	0.918104047	1.55686	0.00853
Epb41l4b	ENSMUSG00000028434	2.980399136	3.865673906	2.185847927	0.702651445	0.676970977	0.056831134	2.652522	0.008743
Nefm	ENSMUSG00000022054	112.4374682	61.4833932	116.7656732	7.135386013	16.13868863	12.40677124	3.026243	0.009052
Naa15	ENSMUSG00000063273	3.927097094	5.16273304	3.838419988	1.079720037	2.265307849	1.827378643	1.321619	0.009304
Malat1	ENSMUSG00000092341	1306.356265	2023.311404	2133.228523	703.5230003	487.1558943	458.7859679	1.727668	0.009317
Spry2	ENSMUSG00000022114	9.644154824	5.036433388	10.39288071	0.568059552	0.287331525	0.603030887	4.103682	0.009364
Set	ENSMUSG00000054766	46.00772889	50.42656373	67.1655543	21.65722855	26.66796595	17.57400419	1.311839	0.009452
Gm13629	ENSMUSG00000087301	184.0624052	225.0602989	175.5115136	36.94486385	25.24704684	106.9965897	1.788903	0.009632
Efnb2	ENSMUSG00000001300	6.121885789	9.152609085	4.753875324	1.041995656	0.325612122	0.25702927	3.623856	0.009775
Abcd4	ENSMUSG00000021240	7.847974153	3.749194683	6.030747395	0.610836083	0.374507177	0.062879114	4.071845	0.009884
Epha4	ENSMUSG00000026235	9.0174065	6.978314232	12.08594831	0.651355322	0	3.099174942	2.904462	0.009946
Ncoa7	ENSMUSG00000039697	7.668015925	5.654817043	8.021720853	2.940005251	3.45285101	3.966767426	1.042896	0.009964
Nrxn1	ENSMUSG00000024109	18.11976118	25.99491113	21.71240461	7.786666104	12.27460664	6.733826484	1.296712	0.010178
Speg	ENSMUSG00000026207	3.201587913	2.457922223	2.203397374	1.137526881	0.700752248	0	2.096707	0.01085
Klhl28	ENSMUSG00000020948	6.443784727	8.098097182	8.76237588	4.798848819	2.676360754	3.347049938	1.106592	0.011101
Abcb7	ENSMUSG00000031333	6.203518888	9.193727623	4.354024835	0.532037554	0.180535785	0.092355806	4.61694	0.01117
1700001O22Rik	ENSMUSG00000044320	7.231311586	5.208178232	9.070049595	0	1.816285181	2.257039923	2.400698	0.011417
Hcn1	ENSMUSG00000021730	11.52620383	9.277281616	11.23054663	6.477289638	2.399305444	1.926219985	1.568198	0.011674
Ndufa3	ENSMUSG00000035674	472.9205396	528.3050326	683.3472262	305.6652301	259.214145	201.7088753	1.135859	0.01183
Nfib	ENSMUSG00000008575	1.895167446	4.141638462	3.277582123	0.1565449	0.251690701	0.291695411	3.734176	0.011867
Nefl	ENSMUSG00000022055	117.9157094	154.7406497	211.8178781	40.52934318	44.87600329	39.91536374	1.950795	0.011917
Ak6	ENSMUSG00000078941	8.686234953	15.9535029	12.07448613	3.403772217	3.055934082	2.84823386	1.979805	0.012268
Rbms3	ENSMUSG00000039607	7.11059989	9.218905333	10.89258436	0.299163014	0.663527683	4.321360857	2.365061	0.012309
Clip1	ENSMUSG00000049550	10.71519863	15.93461313	12.77148354	7.234973003	6.236382915	5.184593911	1.079339	0.013176

Klf9	ENSMUSG00000033863	28.32738305	22.30987963	30.92467991	8.235996374	16.48081509	12.65059861	1.126116	0.013473
Actr2	ENSMUSG00000020152	23.13510862	25.81317614	41.44894342	3.195081188	4.756469837	8.020963579	2.500687	0.013527
Polg	ENSMUSG00000039176	6.470531794	3.275131561	4.737666201	0.529751757	0.992425812	1.116918891	2.456276	0.013727
Gnptab	ENSMUSG00000035311	5.023569702	7.042342192	8.045201817	3.330112734	0.974008663	1.89680873	1.697436	0.014473
Sh3kbp1	ENSMUSG00000040990	2.962639802	4.938838889	3.987750753	0.299319577	1.78446943	0	2.512374	0.014638
Zfp275	ENSMUSG00000031365	2.456859761	4.450118996	4.899377602	1.221572273	0.217322125	0	3.036531	0.01466
Phactr3	ENSMUSG00000027525	14.23082575	18.20959777	13.97076441	10.17710562	5.750870977	6.056584213	1.077982	0.014692
Nudt3	ENSMUSG00000024213	27.31995565	19.6321863	25.74494276	7.913037502	14.15160945	13.19443885	1.043902	0.014841
Gm50490	ENSMUSG00000111374	7.737724762	10.97581224	17.82895114	0	0	0.057943161	9.300721	0.014998
Rgs7bp	ENSMUSG00000021719	65.4511008	42.51560468	74.99164193	25.11127438	8.586355381	17.43950783	1.839072	0.015029
Nova1	ENSMUSG00000021047	7.869617106	4.290510562	5.560174417	1.904291872	1.831973432	0.805732546	1.964004	0.016518
Vcpip1	ENSMUSG00000045210	5.319671219	8.228885938	4.677585451	0.833003483	1.055921494	2.280639021	2.128041	0.016604
Scn2b	ENSMUSG00000070304	26.68660815	48.75788568	44.77455346	15.16299914	13.15121133	9.766907216	1.658518	0.017141
Map4	ENSMUSG00000032479	18.4508472	16.43939766	18.78346565	5.25910775	8.337973467	12.87364419	1.019818	0.017654
Scrt1	ENSMUSG00000048385	3.852381131	3.133733357	5.749199469	1.441324662	1.201755719	0.71833645	1.921693	0.018081
Epb41	ENSMUSG00000028906	8.405844795	7.133261033	7.427715902	3.219610808	1.223930513	5.035842756	1.276686	0.018217
Zfp644	ENSMUSG00000049606	6.056941602	8.425771538	8.488952839	1.888261436	4.792185622	2.268180307	1.360117	0.01824
Brinp3	ENSMUSG00000035131	45.48269762	33.17011198	23.62460643	0.524800069	2.606931701	13.16093312	2.650193	0.018251
Kansl1l	ENSMUSG00000026004	4.983512807	3.208775054	7.086452235	0.595972382	1.124814098	0.327347559	2.899144	0.018273
Ctnnal1	ENSMUSG00000038816	2.412689916	2.178256745	2.739143462	0.57644401	0.004348583	1.478492253	1.831688	0.018638
Dnlz	ENSMUSG00000075467	25.13618095	18.15929589	27.38244212	8.253246842	8.682789928	14.50152795	1.16877	0.018899
Kif5b	ENSMUSG00000006740	29.01035322	46.78770806	42.51539119	14.78397405	13.74155046	21.99778246	1.227593	0.019176
Hba-a1	ENSMUSG00000069919	1205.988575	1276.53587	467.1733279	0.292004996	2.420634415	0	10.08665	0.019182
Ube2h	ENSMUSG00000039159	13.90246951	19.10300667	12.30697803	6.925985871	7.520154605	7.415997313	1.051473	0.019192
Col11a1	ENSMUSG00000027966	5.669898816	3.415339166	2.601389401	0.127912578	0.546353519	0.477312701	3.343174	0.019289
Mtmr3	ENSMUSG00000034354	7.188339685	6.178740058	4.717609568	3.394288705	2.410339641	1.017258324	1.406526	0.019547
Gys1	ENSMUSG00000003865	9.274244059	5.166522069	8.35125016	3.496712577	2.003460904	2.382675057	1.53174	0.019813
Ssrp1	ENSMUSG00000027067	24.38944474	35.69511325	32.42748991	19.23290869	7.140730448	7.094711368	1.466844	0.020003
Abce1	ENSMUSG00000058355	6.928135517	12.05617624	9.163622509	2.680497897	0.460543471	4.255174361	1.92817	0.020065
Acyp1	ENSMUSG00000008822	44.67883014	87.97149661	70.10193354	19.15764374	21.94411234	20.45863854	1.719644	0.020135
Vwa8	ENSMUSG00000058997	3.208846626	2.526341787	4.157735237	0.694121221	1.468167826	1.579919613	1.402507	0.02021
Adra1b	ENSMUSG00000050541	20.88911958	12.1967762	10.28221279	0.368071348	1.566234342	3.497971418	2.997006	0.020268
Pacsin2	ENSMUSG00000016664	20.29275519	31.62374898	21.65099467	4.124511078	4.38491506	13.15451143	1.763773	0.020377
Atg7	ENSMUSG00000030314	5.531555309	8.910873719	5.785239782	0.085070342	2.902109336	2.004826725	2.018638	0.020605
4632415L05Rik	ENSMUSG00000048106	6.263879604	11.13340249	13.14813123	2.415239439	2.8069141	2.640692656	1.957832	0.02094
Clint1	ENSMUSG00000006169	9.010039874	13.18371499	6.443855393	2.326241424	0.672637302	2.80348342	2.303199	0.02117
Maf	ENSMUSG00000055435	2.332437849	2.77006346	4.626249545	0	0.477018601	0.941743358	2.777622	0.021306
Cox5b	ENSMUSG00000061518	466.3829816	665.8489666	701.274902	377.3073469	270.7773522	200.7666641	1.111022	0.021311
Plcb4	ENSMUSG00000039943	32.90982717	61.09461683	41.84609095	15.38757277	10.79657012	16.17235617	1.681365	0.021321
Pdp1	ENSMUSG00000049225	13.93328944	12.98314809	20.00122369	7.536216812	1.552261757	6.385241228	1.600311	0.021544
Capn7	ENSMUSG00000021893	7.380714046	7.386418933	12.73834011	1.422890536	3.627502614	0.797023275	2.233847	0.021868
Homer1	ENSMUSG00000007617	3.885080717	5.44701427	3.286515485	1.846219633	1.507720085	0.073561107	1.880324	0.022042
Gsk3a	ENSMUSG00000057177	3.504935281	4.421635206	4.438928423	1.57390693	2.52730644	0.216987148	1.517818	0.022057
Snap25	ENSMUSG00000027273	750.6880551	1109.467002	766.2614644	202.2672085	331.0894586	502.9715878	1.341615	0.022119
Gm36388	ENSMUSG00000103961	1.722340494	3.385613638	1.937118958	0.017694631	0	0.751358484	3.19546	0.022371
Cdk19	ENSMUSG00000038481	4.412684744	2.86071593	5.972548143	1.135652685	0.68384182	1.420846398	2.031334	0.022533
Ntng1	ENSMUSG00000059857	18.21952989	33.53505075	16.88321133	2.563660753	1.931860746	5.420072046	2.791232	0.02291
Gm44559	ENSMUSG00000108943	16.93853166	23.69955117	22.443399	7.761071531	3.818657414	13.59671776	1.325142	0.022935
Arih2	ENSMUSG00000064145	9.457651301	4.708081371	8.38493143	0.919442739	2.857264995	2.214558619	1.912237	0.023413
Ppp4r4	ENSMUSG00000021209	4.753358208	3.679248788	2.683815672	1.760493386	1.541749006	0.970713818	1.379386	0.023763
Pacsin3	ENSMUSG00000027257	3.69799957	2.078571682	3.928650714	0	1.509045718	0	2.685125	0.02377
Cd68	ENSMUSG00000018774	35.49373772	28.96528518	12.56973182	0	2.623814562	1.468446574	4.234427	0.023937
Hspa12a	ENSMUSG00000025092	17.58296665	17.33323386	19.41439121	9.518371742	1.975239254	11.41873623	1.245639	0.023989
Ddhd1	ENSMUSG00000037697	4.730604335	2.976674078	2.585733218	0.802235807	0.972737783	1.338159354	1.725226	0.024256
Atp2a2	ENSMUSG00000029467	48.03180567	73.90990637	87.81381226	33.00640853	27.14575197	20.03375508	1.387288	0.024292
Pcnp	ENSMUSG00000071533	23.08883938	45.21137742	35.70975853	13.13038197	12.26700629	10.42374218	1.537839	0.024445
Cd44	ENSMUSG00000005087	14.91629991	24.88341625	16.72324698	8.835564404	4.611717143	7.98031936	1.399367	0.024509
Chd6	ENSMUSG00000057133	7.363407927	9.572577667	12.07870683	4.636789441	3.664193022	5.400680151	1.082433	0.024542
Armh4	ENSMUSG00000036242	6.863981784	5.692148075	10.19509997	1.755100897	3.622156179	1.733057389	1.677959	0.024758
Tatdn3	ENSMUSG00000026632	12.7993385	8.621703583	8.861356388	1.170221028	4.747255604	5.059398839	1.464012	0.025017
Gm43884	ENSMUSG00000108123	4.200699398	7.75393208	4.858052377	0	0	2.476525952	2.76316	0.025078
Scn1a	ENSMUSG00000064329	41.49106742	79.3961786	68.48599378	27.55641929	17.17731894	23.30142691	1.47688	0.025623
Kcna2	ENSMUSG00000040724	27.85134467	22.6048297	50.70729218	3.32580594	1.096865584	6.116804414	3.262813	0.025984
Flt3	ENSMUSG00000042817	12.26456143	8.053398831	9.80497032	6.55060917	4.450203617	3.695152477	1.035442	0.026051
Plekhd1	ENSMUSG00000066438	8.805822567	7.574371827	4.781218065	1.399533467	0	3.535842034	2.100204	0.026333
Marchf11	ENSMUSG00000022269	9.199122907	5.098455376	8.643680653	0.50993218	2.397988507	3.711943074	1.793071	0.026491
Bola2	ENSMUSG00000047721	21.6848792	32.76953095	22.63002637	15.70207404	9.09747778	7.16350327	1.270034	0.026624
Gm42616	ENSMUSG00000106010	11.51325534	10.10058994	6.659148364	0.049586413	2.189647669	5.053352712	1.954922	0.026754
Rragb	ENSMUSG00000041658	7.309312629	9.949906542	14.36968226	0	4.809798512	0.8732019	2.476519	0.027014
Rims2	ENSMUSG00000037386	18.71177844	16.13579533	11.61009131	8.424536679	8.523431034	4.724467513	1.100055	0.027037
Rab24	ENSMUSG00000034789	33.06279514	35.76188514	50.65318424	23.91673082	19.04630128	15.69773694	1.026275	0.027346
Fbxw7	ENSMUSG00000028086	7.498480937	13.06198977	15.22649157	1.619475624	2.042557678	5.51326794	1.963607	0.027355
Sppl2a	ENSMUSG00000027366	2.779657262	5.410765096	4.452788122	1.658935483	0.734341414	1.752591718	1.608617	0.027442
Acsl3	ENSMUSG00000032883	14.94422329	24.80427763	19.98558226	7.23106107	4.228348675	11.85253495	1.357485	0.028183
mt-Tp	ENSMUSG00000064372	887.7063068	1619.487797	715.8917889	192.8714792	161.6001574	55.09962218	2.976256	0.028635
Rhobtb3	ENSMUSG00000021589	7.87430533	6.666747465	7.717396819	0.912585066	2.36273007	5.355427495	1.366797	0.028956
Eif5a2	ENSMUSG00000050192	2.876098512	6.746977493	4.069137674	0.436293918	0.60969434	1.093208908	2.678214	0.029482
Bfar	ENSMUSG00000022684	10.33804531	12.80936417	18.43960745	4.217083885	5.727213935	6.726378365	1.318821	0.029528
Pcdh20	ENSMUSG00000050505	18.06770594	34.56167215	13.42143749	1.160283873	0	1.428406538	4.673282	0.030206
Ctnnbl1	ENSMUSG00000027649	27.61775166	18.33394069	35.42465624	9.992724301	11.1582002	11.38333566	1.32265	0.030405
Ankrd40	ENSMUSG00000020864	12.69292413	9.193511985	19.76842614	3.273818649	4.877774325	1.547892655	2.102505	0.030735
Mbnl1	ENSMUSG00000027763	4.355172946	8.911898727	7.083034561	2.025305905	2.970662524	1.983571144	1.543833	0.030764
Slc25a36	ENSMUSG00000032449	7.695834169	13.57244559	10.32607561	4.392949462	3.420343608	5.793712168	1.215317	0.030802
Mgat4c	ENSMUSG00000019888	10.21020805	12.63588972	9.97907588	2.223373185	0.124645006	7.498650442	1.737095	0.031196
Gabrb3	ENSMUSG00000033676	50.64655228	76.60263427	73.64163719	17.10715422	32.86636261	42.76524152	1.115167	0.031401
Rasgrp1	ENSMUSG00000027347	20.86415594	10.19111293	20.63189608	6.001797694	6.157232371	0.007491223	2.086889	0.031541

Zfp322a	ENSMUSG00000046351	4.263497655	5.38662843	4.910405401	2.9576987	0.271282004	2.592204766	1.322678	0.031984
Cnksr2	ENSMUSG00000025658	2.700443138	7.222520957	4.319564607	0	1.01737367	0	3.807284	0.032032
Ier5	ENSMUSG00000056708	5.539731865	4.708000891	4.500032644	0.298556391	2.003344113	3.355043456	1.382402	0.032126
Mrpl9	ENSMUSG00000028140	25.01957076	30.57472284	26.98284659	20.70199832	7.062229025	5.385477063	1.316746	0.032261
Atp5g1	ENSMUSG00000006057	128.7052318	223.5317442	265.6243894	79.6836574	68.12108665	77.49633574	1.455429	0.0323
Atp5mpl	ENSMUSG00000021290	301.772446	569.0521236	450.0535099	203.840842	166.5177214	201.9638641	1.206597	0.033267
Il1rapl1	ENSMUSG00000052372	16.04744383	15.75579438	8.324382095	5.62503459	4.161637174	5.711997383	1.372451	0.03334
Map7d2	ENSMUSG00000041020	49.53285269	86.97469836	73.45473115	39.4484862	32.27973174	18.70024486	1.215281	0.033487
Bcl2l2	ENSMUSG00000089682	6.751218177	9.090813426	2.900813273	0.624388093	0.343448392	0.554706426	3.621785	0.033618
Fmc1	ENSMUSG00000019689	146.6463028	275.1231649	233.4150723	110.6819722	80.26423805	93.94798194	1.201475	0.033627
Rbm45	ENSMUSG00000042369	8.960807005	13.81633857	4.366715822	0	0.122866943	0.884106401	4.752529	0.033628
Isoc1	ENSMUSG00000024601	37.81701002	26.173118	56.71945089	10.08428922	14.58064025	10.0746634	1.796887	0.033701
Fkbp11	ENSMUSG00000003355	8.454578383	15.19032354	13.63286606	1.807226045	2.659250074	7.37938063	1.653933	0.033833
Cplx2	ENSMUSG00000025867	12.69531583	7.908667187	21.48440172	0.108257534	2.778461248	0.621430997	3.58464	0.033904
Lpin1	ENSMUSG00000020593	6.915187603	3.852115195	9.316060873	0.077603342	2.700721819	0.308399996	2.701852	0.034003
Runx1t1	ENSMUSG00000006586	4.669997286	2.718447012	5.654255709	0.577204247	2.151482587	1.054173859	1.785693	0.034585
Luzp1	ENSMUSG00000001089	17.54609443	10.65979268	25.79900018	4.01172101	2.242321711	5.538629064	2.195199	0.03479
BC030343	ENSMUSG00000105345	2.970826733	1.125522928	4.077749879	0	0.068295396	0	6.903128	0.034956
Rad50	ENSMUSG00000020380	4.39993257	4.623411235	5.57073497	0.507300959	3.563876101	0.905357851	1.55217	0.035197
Tnfrsf12a	ENSMUSG00000023905	3.542080424	4.281351792	8.944832934	0.414202739	0.362114065	0.130282139	4.209125	0.035454
Lgalsl	ENSMUSG00000042363	17.20892597	25.65733414	12.71883511	5.661206951	3.197867588	8.492115911	1.679663	0.035653
Nudcd1	ENSMUSG00000038736	2.171251667	3.057263813	2.58605076	1.871411472	0.595619725	1.167762762	1.104292	0.036121
Grk5	ENSMUSG00000003228	1.336117568	4.664165082	3.239375277	0	0	0.237850312	5.279714	0.036148
Grin2a	ENSMUSG00000059003	9.590599192	5.226813066	13.00555379	1.852716584	2.784722383	2.145147397	2.036369	0.036484
Gm22003	ENSMUSG00000119679	4.389141988	6.076329018	11.86593592	1.107981575	0	0	4.333068	0.036792
Onecut2	ENSMUSG00000045991	8.715983637	3.804723695	11.02321294	0.248295982	0.880369467	2.103485946	2.864788	0.036872
Kif2a	ENSMUSG00000021693	7.949563831	13.9441808	16.56110303	4.742717471	2.704555354	5.964855541	1.519627	0.037351
Fam13a	ENSMUSG00000037709	1.657321153	5.63210049	4.166381175	0.253923431	0.272731698	0.319473384	3.759058	0.038142
Tnr	ENSMUSG00000015829	3.767262813	6.543611801	5.100965048	1.577864101	2.286212652	3.1429718	1.13716	0.038275
Ankrd34b	ENSMUSG00000045034	4.479338883	1.901874488	2.243259462	0.455688926	0.049115724	0.591977014	2.97516	0.038287
Klhl9	ENSMUSG00000070923	10.88985816	15.87082274	18.04812454	7.881826285	1.125381805	7.738896384	1.419957	0.038326
Pafah1b1	ENSMUSG00000020745	11.85254096	23.68476974	15.504076	7.267904191	6.655972707	3.492996862	1.551182	0.038451
Slc4a10	ENSMUSG00000026904	6.673608014	6.415531867	8.723718446	5.393433194	1.540224893	2.05052405	1.27972	0.038826
Hsf2	ENSMUSG00000019878	4.033432324	4.076047668	1.93486785	0.172783302	1.141002345	1.449894001	1.861722	0.039314
Pet100	ENSMUSG00000087687	26.97905566	45.85755516	36.62921951	24.26777094	8.887591517	8.939190916	1.378775	0.039799
Ttc9	ENSMUSG00000042734	9.773012682	8.920483952	3.166608858	1.966389025	0.285871547	0.526800109	2.975631	0.041058
Immt	ENSMUSG00000052337	17.0963407	28.33827228	17.02056365	9.554913776	9.14072481	10.16531179	1.113702	0.041188
Uhrf1bp1l	ENSMUSG00000019951	8.274698763	12.89930483	8.019839382	5.860304713	4.235107303	2.284924407	1.237614	0.041515
Kdm1a	ENSMUSG00000036940	9.787097747	17.05239982	18.95180464	6.873112003	6.400448256	7.549370727	1.1369	0.041588
Paxbp1	ENSMUSG00000022974	18.59989419	9.010426648	12.1466544	2.652829342	5.758060689	5.019173801	1.565742	0.041945
mt-Nd6	ENSMUSG00000064368	3312.351839	3001.47303	939.3980251	210.781753	326.5975827	100.1485579	3.508062	0.041957
Trip11	ENSMUSG00000021188	11.01551088	6.51946535	13.19343344	5.60065583	2.878503569	3.364334074	1.375478	0.042108
Atp5j2	ENSMUSG00000038690	132.5486931	240.9774799	196.3540412	112.4308887	83.34669684	83.78749365	1.027475	0.042338
Kcnn4	ENSMUSG00000054342	59.39851787	23.84072493	46.49493384	6.238052286	4.197742523	19.03528912	2.138187	0.042552
Pum2	ENSMUSG00000020594	10.99241697	17.47809451	17.55151528	3.086323883	3.730672674	10.62061431	1.400122	0.042614
Fastkd1	ENSMUSG00000027086	6.596782601	3.295454254	5.778609651	2.774970427	1.240826873	2.128720667	1.350711	0.043264
Dynll2	ENSMUSG00000020483	61.69641254	65.4946293	92.83682934	48.40089542	38.5868536	19.45746601	1.047575	0.04336
Wapl	ENSMUSG00000041408	6.007106152	12.33100182	5.713842203	1.749759367	0.125753035	2.387961643	2.496052	0.043363
Lyrm4	ENSMUSG00000046573	25.61750586	39.44500088	57.17979246	13.63996493	13.83046586	14.82156064	1.531287	0.043452
Arel1	ENSMUSG00000042350	4.073868222	1.213176912	2.865103479	0.617877548	0	0.090231806	3.525136	0.043456
Zfyve9	ENSMUSG00000034557	6.850383476	5.435675657	3.696635754	2.675220569	1.201776916	2.890250453	1.23987	0.043623
Zzz3	ENSMUSG00000039068	2.806405162	1.900412398	4.140539862	0.391777814	1.5239085	0.072964342	2.153457	0.043662
Map2k7	ENSMUSG00000002948	5.239033677	4.02805195	9.514768601	1.091415384	2.105538664	0.525892071	2.334862	0.04384
Map1a	ENSMUSG00000027254	35.94721307	20.04049826	47.55959752	10.15641429	14.69660441	5.761948725	1.757981	0.044117
Adk	ENSMUSG00000039197	23.15849337	44.58069107	42.09350072	13.22365484	11.40570114	21.13287535	1.263078	0.044636
3110001I22Rik	ENSMUSG00000079737	6.000823158	17.8165728	7.839675779	0.052149866	0	0	9.245649	0.045469
Mkks	ENSMUSG00000027274	2.401310871	3.90642073	3.071076467	0.51433418	1.414343253	2.066992546	1.230967	0.045677
Crtac1	ENSMUSG00000042401	121.0744929	67.1119608	131.9733475	22.59276949	32.66243094	63.22485561	1.434148	0.045788
Cgnl1	ENSMUSG00000032232	9.754905639	2.431357897	9.770244158	0.007556988	0.16139097	0.721999563	4.623164	0.045831
Dlg3	ENSMUSG00000000881	9.413887701	17.30614661	9.088621976	3.022681425	5.119167625	4.048644536	1.554552	0.045989
Lrp11	ENSMUSG00000019796	51.66294923	33.40450125	51.73632151	32.80353203	7.729196528	9.757438479	1.44376	0.04605
mt-Nd2	ENSMUSG00000064345	3720.39284	5406.88986	1590.398682	428.5020005	557.2956393	231.9421202	3.137715	0.046053
St3gal6	ENSMUSG00000022747	3.174112404	3.218765417	6.868498786	0.009604182	1.705667547	0.006796962	2.945016	0.046288
Larp4	ENSMUSG00000023025	4.96857049	4.040767826	2.351646912	0.893829368	1.026195794	2.108568897	1.49574	0.046338
Thap12	ENSMUSG00000030753	9.498483275	15.60401292	13.15824421	0.359254632	4.207721544	8.502038671	1.549714	0.046373
Adora1	ENSMUSG00000042429	16.01605986	9.47184913	13.87561273	0.363756959	1.814013069	9.013812436	1.814445	0.046445
Ddx17	ENSMUSG00000055065	26.36947218	43.2518518	30.57069404	21.71603296	16.0840616	9.30350498	1.088858	0.046519
Zfhx4	ENSMUSG00000025255	9.890799727	19.01387786	11.24360116	3.358038903	4.894396016	6.453771667	1.448913	0.046576
Iqsec3	ENSMUSG00000040797	8.912051691	5.836941755	10.32303311	2.276862557	5.50620347	3.417370455	1.162524	0.046798
Klf13	ENSMUSG00000052040	8.788399893	6.321429765	11.52035163	5.391681685	3.883963049	1.804816755	1.265044	0.046961
Retreg1	ENSMUSG00000022270	4.794540424	7.595574273	8.565863741	4.550963432	1.560360172	0.060768974	1.76353	0.047077
Asap1	ENSMUSG00000022377	12.70625347	8.906995256	17.04488178	4.389129364	6.745717507	6.588111097	1.125152	0.047624
Gm15266	ENSMUSG00000081306	1.18396183	3.541215713	2.074599907	0.464917759	0.130645074	0.329026264	2.878603	0.047744
Pdzd4	ENSMUSG00000002006	7.030208283	6.277852944	7.799868569	1.567779974	5.697869375	1.363138409	1.290555	0.048177
mt-Rnr2	ENSMUSG00000064339	30417.23673	39079.51041	10642.45754	3282.361623	3755.398446	2597.372743	3.056132	0.04929
C130023A14Rik	ENSMUSG00000102854	2.803615478	8.061858046	6.969096547	2.217847472	0.312964148	1.127930173	2.285257	0.049371
Cadps	ENSMUSG00000054423	20.75708291	44.70301432	35.73441149	15.31539438	10.59584145	15.39089527	1.292843	0.049448
Wdr17	ENSMUSG00000039375	8.34172671	10.09788089	8.040140198	3.387386829	0.319405436	6.639610814	1.35576	0.049804
Zfp106	ENSMUSG00000027288	9.061071387	13.07108933	18.97834208	5.195051399	6.99895065	3.435231067	1.39526	0.049966

Table S2. Enriched genes in Drd2 neurons

Gene name	Gene ID	PV-1 FPKM	PV-2 FPKM	PV-3 FPKM	Drd2-1 FPKM	Drd2-2 FPKM	Drd2-3 FPKM	log2F.C.	P value
Tmem9b	ENSMUSG00000031021	82.6742844	70.3283318	68.80998154	189.2452731	186.7408849	193.5773928	-1.360516	1.78E-05
Zcchc12	ENSMUSG00000036699	27.1160147	22.54356561	36.42833653	314.7401468	318.2264757	283.5362187	-3.412257	1.93E-05
Cdh4	ENSMUSG00000000305	0	0.009954637	0	2.262606167	2.703904776	2.416350387	-9.534596	4.52E-05
Ptprr	ENSMUSG00000020151	0.333815066	0	0	6.348609218	7.147248499	6.035431599	-5.870594	5.22E-05
Gm47328	ENSMUSG00000111399	4.456055347	0	0	32.77930416	31.32556897	34.68771669	-4.470564	5.98E-05
B230216N24Rik	ENSMUSG00000089706	0.126089676	0.586269951	1.38590221	11.91515587	11.7537041	10.37124177	-4.019969	6.44E-05
Nme5	ENSMUSG00000035984	4.767310201	0	0.122246788	32.64345109	33.8052621	30.79061426	-4.313764	6.83E-05
Spring1	ENSMUSG00000032840	4.851609684	4.897898143	6.474264784	13.81451395	13.6702066	13.82766513	-1.348465	9.83E-05
Gng4	ENSMUSG00000021303	0.170741002	0.086515929	1.436070355	21.56334501	25.79379684	26.27580105	-5.442419	0.000104
Hpcal1	ENSMUSG00000071379	17.75244243	11.5500041	33.91573064	262.8821925	307.8298554	259.7982648	-3.715587	0.000111
Prdx2-ps1	ENSMUSG00000082431	1.460946759	0.726038196	0.82705993	5.014219799	5.524514078	4.989129411	-2.365087	0.000133
Gstm6	ENSMUSG00000068762	11.8058872	0	0.106389739	71.55561148	72.64201127	63.33755589	-4.122835	0.000184
Car8	ENSMUSG00000041261	2.072112452	0	1.226560755	17.90771985	20.6043185	16.69105919	-4.064791	0.000186
Wdr6	ENSMUSG00000066357	33.53752716	18.55042416	41.81691747	147.2456412	173.2785823	162.9966317	-2.364306	0.000218
Rnasek	ENSMUSG00000093989	0.341141544	0	0	2.310798861	2.409183746	1.990887735	-4.298058	0.000236
Tafa1	ENSMUSG00000059187	7.665970229	3.848614078	6.000782694	70.10132904	86.10065354	69.41700713	-3.687195	0.000238
Celf6	ENSMUSG00000032297	4.252257278	6.094881129	3.730383871	32.69976139	26.71783776	27.90930531	-2.633033	0.000241
Slc41a3	ENSMUSG00000030089	1.659417475	0	0	18.92751108	17.53273954	22.43815577	-5.14948	0.000257
Nudt21	ENSMUSG00000031754	4.263737931	5.239145909	5.735202361	12.88028581	11.45769513	12.45195285	-1.271629	0.000285
Uchl1	ENSMUSG00000029223	397.5521834	318.3552926	324.1562355	739.546132	674.8634628	698.2811306	-1.02241	0.000354
Cd83	ENSMUSG00000015396	3.991037741	0.379179941	0.12341127	40.41300331	35.73665976	47.72479911	-4.784854	0.000426
Tle6	ENSMUSG00000034758	0.010863208	0	0	8.59977409	11.20911893	11.80546876	-11.50691	0.000432
Necab2	ENSMUSG00000031837	3.280937483	16.44029669	1.362940874	159.0807277	123.2763432	128.2943417	-4.283682	0.000435
Nudt2	ENSMUSG00000028443	22.86657642	12.35360435	17.94713056	56.89190363	56.57550162	64.53976724	-1.743324	0.000482
Zfp57	ENSMUSG00000036036	0.088604293	0	0	5.730424517	4.989254646	6.947486038	-7.639478	0.00051
Armcx6	ENSMUSG00000050394	0.310357385	0	0.184427425	11.1591483	9.125973614	8.049298002	-5.839611	0.000535
Kitl	ENSMUSG00000019966	0.039725782	0	0.011933073	11.11130634	8.735825694	12.39150429	-9.285559	0.000558
Gde1	ENSMUSG00000033917	92.1908813	54.8850763	45.33589337	258.0757243	239.3642455	222.7213203	-1.904122	0.000559
Gm44644	ENSMUSG00000109121	0.010683307	0.149543058	0	6.352457102	5.15631977	4.555808729	-6.647628	0.000562
Gpc6	ENSMUSG00000058571	1.200853633	0	0.010978428	8.192683061	11.28803944	9.891912186	-4.599211	0.000663
Sqstm1	ENSMUSG00000015837	160.55101	42.42747851	144.8770511	483.6393224	493.8307903	458.4342746	-2.045399	0.000708
C1qtnf12	ENSMUSG00000023571	9.258188594	0	3.872788993	37.90517713	47.84995403	40.32318035	-3.263274	0.000725
As3mt	ENSMUSG00000003559	7.885092411	9.277500071	2.097206071	39.52108532	34.43465755	32.14229099	-2.461733	0.000729
Gm35595	ENSMUSG00000114571	1.026905669	1.105726539	0.899698939	6.351108671	4.929185335	6.635083214	-2.562699	0.000731
Gramd1c	ENSMUSG00000036292	0.31419739	0	0	2.073159825	2.927798296	2.52474219	-4.582083	0.000857
Calb1	ENSMUSG00000028222	4.11878316	0.544116636	18.53654589	261.7313814	223.4325978	181.0223271	-4.843763	0.000862
Drd2	ENSMUSG00000032259	0.013170626	0	0	42.74135716	55.02712061	37.74122454	-13.32878	0.000922
Btf3	ENSMUSG00000021660	44.79174746	26.66969175	42.02168323	88.0633504	89.0813094	94.95028207	-1.261632	0.000933
Vat1	ENSMUSG00000034993	0.897459618	0	1.542824298	25.08450658	20.78843696	17.2292712	-4.69257	0.000946
Dnajc3	ENSMUSG00000022136	8.821947323	6.737884432	7.312954585	14.72989413	15.18301097	17.05451737	-1.038028	0.00105
Bod1	ENSMUSG00000044502	32.30088891	36.09842923	49.7916597	102.7792756	107.3196838	91.09764592	-1.349586	0.001053
Rtn1	ENSMUSG00000021087	221.8589145	163.0930363	370.8259851	912.0328086	949.3225958	804.1337483	-1.818366	0.00109
Tubb2a	ENSMUSG00000058672	266.4686268	155.3401562	169.6378439	799.1903789	957.2847424	724.8637641	-2.068799	0.00121
Timp2	ENSMUSG00000017466	15.49847365	15.5106339	18.8018604	133.8829792	110.4269444	93.49569285	-2.761658	0.001226
Ndrg1	ENSMUSG00000005125	0.640804083	0	0.005346907	11.73228352	10.85903003	7.771973487	-5.554313	0.001251
Zfp282	ENSMUSG00000025821	0.468403708	0	0.257734453	2.278100052	3.008434013	3.257308493	-3.556569	0.00129
Pgrmc1	ENSMUSG00000006373	97.80064526	102.56482	93.63070449	381.0710804	472.6818996	345.6596317	-2.028459	0.001352
Eef1g	ENSMUSG00000071644	66.18802002	69.35116853	82.74461938	153.3792744	133.6680005	161.8173314	-1.040075	0.001407
Dlg2	ENSMUSG00000052572	2.252641423	0.713514342	1.219683639	6.559710891	5.906005425	5.300784353	-2.083946	0.001435
Khdrbs3	ENSMUSG00000022332	22.30570725	13.37500349	22.51694372	53.87053617	56.83114253	46.80807423	-1.436408	0.001443
Acsl1	ENSMUSG00000018796	1.996799119	0.885523502	1.180877387	4.752678669	5.49468283	6.174774354	-2.014953	0.001459
Tubb5	ENSMUSG00000001525	100.0317856	96.65762658	109.1755863	294.9508063	226.9531128	287.1495875	-1.40334	0.001537
Arntl	ENSMUSG00000055116	0.196199089	0.196854501	0.281282986	4.960106404	3.263695609	4.756622883	-4.266725	0.001561
Minpp1	ENSMUSG00000024896	3.374630672	4.160464953	12.49027072	30.66931534	28.84183006	28.78826005	-2.140575	0.001582
Adgra1	ENSMUSG00000025475	4.347348397	0.154246046	2.815693525	22.75440531	24.72299155	17.33023364	-3.146783	0.001622
Prokr2	ENSMUSG00000050558	0.32194273	2.554290208	0	8.38740933	8.047812476	10.26124434	-3.214396	0.001685
Saraf	ENSMUSG00000031532	67.89445422	29.76126066	93.44585728	207.8693949	237.3362754	252.2285936	-1.867718	0.001728
Stx1a	ENSMUSG00000007207	1.786749099	0	0.171975461	24.46900007	20.9808835	15.53094061	-4.960369	0.001788
Ldha	ENSMUSG00000063229	46.47735762	48.51700895	55.7685047	185.5495906	200.7324073	258.4729935	-2.09647	0.001821
Magi1	ENSMUSG00000045095	0.780056145	0.880902096	0.413390359	5.580858891	6.468283453	8.379066388	-3.299832	0.001869
Pqbp1	ENSMUSG00000031157	45.39726505	68.59785064	40.48513242	123.6990994	136.8419313	118.2880593	-1.294125	0.001891
Inpp5a	ENSMUSG00000025477	8.563061401	7.410788444	7.13605077	22.6446799	18.0519855	24.31352074	-1.492155	0.001906
Tox3	ENSMUSG00000043668	4.677989831	2.830651154	2.017727681	9.485028802	10.58201746	9.112684359	-1.614968	0.00191
Nrsn2	ENSMUSG00000059361	203.7865481	24.1547419	163.6395928	802.2970734	725.0424513	600.8053508	-2.442214	0.00194
Ppp1r9a	ENSMUSG00000032827	8.153809574	5.354300612	6.232794271	23.3971038	18.59338973	18.11297397	-1.60626	0.002012
Prpf8	ENSMUSG00000020850	6.586822452	6.685735529	4.769549955	19.19491576	16.60589775	14.45592173	-1.477949	0.00204
Sox5os4	ENSMUSG00000086282	0	0	0.579751207	2.517243071	3.377085692	2.413610474	-3.840985	0.002047
Rasgrf1	ENSMUSG00000032356	10.197397	7.058872403	10.21416503	38.01058837	29.26122553	28.57647978	-1.802873	0.002085
Ccdc115	ENSMUSG00000042111	6.226313886	4.062302194	9.527822382	20.01152841	24.13106485	20.05986316	-1.695931	0.002121
Gm13306	ENSMUSG00000073877	0.615515325	0.16568991	1.164821232	5.438258189	7.640938988	5.42666942	-3.249379	0.002216
Trit1	ENSMUSG00000028653	3.809441156	0	1.520909371	13.36360096	19.47469222	18.91506882	-3.27935	0.002314
Cacng5	ENSMUSG00000040373	0.367479024	0	0.073590398	3.068984698	2.832687804	1.94763982	-4.153489	0.002347
Celf4	ENSMUSG00000024268	21.4726139	21.76566588	21.10832288	59.73283192	59.39008185	44.98609012	-1.350719	0.002393
Slco3a1	ENSMUSG00000025790	5.787543729	7.122632624	5.47710044	13.11243371	14.98248013	11.80703023	-1.117751	0.002409
Trmt61a	ENSMUSG00000060950	0.027575491	0	0.014495766	2.728740484	4.477533717	3.293305427	-7.963281	0.002492
Tmem163	ENSMUSG00000026347	9.316481257	11.63898266	7.42834375	68.87569201	48.63667738	48.53865673	-2.548487	0.002614
Slc6a16	ENSMUSG00000094152	1.819271155	0	0	4.802535878	5.871899899	4.938658115	-3.101324	0.002682
Dcc	ENSMUSG00000060534	0.988207961	0.035651447	2.093973971	5.902498231	4.952530501	6.233225338	-2.454389	0.002763
Farsb	ENSMUSG00000026245	27.15655962	16.91592791	19.9628772	51.30176711	45.76292514	58.0426819	-1.276326	0.002893
Rph3al	ENSMUSG00000020847	0.008795382	0	0.073976295	6.916169729	4.646908967	4.33367446	-7.585379	0.00293
Msra	ENSMUSG00000054733	5.30324851	0	7.833727892	19.37026531	19.22079537	22.00588176	-2.205612	0.003094
Etnk2	ENSMUSG00000070644	0.036734106	0	0.20082636	11.88526206	20.80879037	17.0247312	-7.709352	0.003103
Gm10524	ENSMUSG00000097141	3.101150243	2.728588082	0.298093595	8.846147136	10.48887397	8.155262718	-2.165473	0.003123
Ptdss1	ENSMUSG00000021518	12.06203622	13.43369335	8.139446819	24.26851213	27.35605631	22.48971908	-1.139781	0.00318

Dynll1	ENSMUSG00000009013	56.1133992	27.07637203	44.77882571	126.5160439	103.0265714	132.323114	-1.499665	0.003184
Gm46376	ENSMUSG00000114181	0	0	0.053926909	30.27297059	17.67603886	21.80930973	-10.33714	0.003308
	ENSMUSG00002075948	5.229539898	0	0.134099614	23.3276463	15.45387174	19.93861631	-3.452571	0.003391
Tm6sf2	ENSMUSG00000036151	0.123523985	0	0	4.381073384	3.908012709	6.465859375	-6.900264	0.003445
Bex4	ENSMUSG00000047844	11.0215483	0.0863092	1.348366009	58.25011258	78.84828266	49.46892195	-3.904758	0.00345
Fam53a	ENSMUSG00000037339	0.434318527	0.142674392	0	2.266627109	2.789001417	3.709939001	-3.925222	0.003459
Chrm3	ENSMUSG00000046159	0.123388527	0.028882457	0.344052471	9.787864581	6.430608387	11.37832071	-5.797076	0.003479
Psme1	ENSMUSG00000022216	21.65504957	26.32586892	26.16775791	83.92433776	65.18118742	96.79650333	-1.729591	0.003541
Plpp2	ENSMUSG00000052151	0.02028149	0	0.042645947	27.30899932	40.86765919	24.5770087	-10.5255	0.00358
Ildr2	ENSMUSG00000040612	1.939309676	0.323949778	1.795254313	6.925589837	6.262365795	4.982648374	-2.162583	0.003618
Tesk1	ENSMUSG00000028458	0.824643389	0	0.652231004	3.402345541	2.359213151	3.264105278	-2.611486	0.00365
Sumo3	ENSMUSG00000020265	38.92571047	43.29513552	57.93235217	87.24734312	100.7535885	109.2899843	-1.084871	0.003697
Morn4	ENSMUSG00000049670	26.36030048	1.903497507	2.173348394	59.35259057	59.47904345	59.44076906	-2.550178	0.003703
Snu13	ENSMUSG00000063480	37.6986296	58.15829155	46.02717004	86.6449928	106.4719379	100.3721401	-1.048594	0.003755
Eif1	ENSMUSG00000035530	238.0969062	136.7341368	179.1819481	557.3536806	460.5276237	425.6271523	-1.381588	0.003808
Pacc1	ENSMUSG00000026627	1.000691989	0	0.015702671	18.01271693	10.30880379	13.3361113	-5.357048	0.003929
Klhl11	ENSMUSG00000048732	0	0	0.044214206	13.57451058	8.144161714	8.852978937	-9.433469	0.003953
Itm2c	ENSMUSG00000026223	109.6055873	46.24165254	43.67819837	533.4217389	550.2441695	346.0313482	-2.841065	0.004001
Tm4sf5	ENSMUSG00000018919	0	0	0.282145587	9.247214225	5.774512291	10.51567941	-6.500028	0.004059
Fscn1	ENSMUSG00000029581	3.898059369	0.095937318	1.112376776	22.49456418	21.85572381	13.8861209	-3.51155	0.004087
Pebp1	ENSMUSG00000032959	282.5760183	101.6805612	230.4401743	503.6534549	571.7166641	572.9328014	-1.423035	0.004138
Tspan7	ENSMUSG00000058254	85.90271053	23.41411542	60.87968765	391.805379	357.7477903	248.6479694	-2.552129	0.004148
Fam76a	ENSMUSG00000028878	16.31653788	8.316581799	10.26752279	33.69844512	26.52860774	30.8110508	-1.383217	0.004188
Ptov1	ENSMUSG00000038502	57.69554007	21.26242409	50.88063376	165.5408762	134.3571249	124.2892223	-1.707982	0.004203
Gm45606	ENSMUSG00000109715	0.144023979	0	0	1.675450493	1.716381801	2.701664675	-5.402889	0.004253
Lancl1	ENSMUSG00000026000	22.0688208	6.499882608	19.4198633	48.32506117	42.43556847	48.25906317	-1.534527	0.00427
Ahcy	ENSMUSG00000027597	2.798455236	1.880063615	0.062866664	19.632209	12.67257222	21.47644162	-3.503722	0.004283
Lmo1	ENSMUSG00000036111	7.175988586	0	0.181794837	17.87476976	17.4514714	15.43423622	-2.786363	0.004299
Dcun1d4	ENSMUSG00000051674	7.434676795	2.009107851	3.194605196	18.84370042	14.18175119	16.85402618	-1.980634	0.004317
Cadm1	ENSMUSG00000032076	6.278068496	20.60130306	11.2578433	51.64003667	79.15792591	64.22412522	-2.354366	0.004354
Yrdc	ENSMUSG00000028889	14.25814373	13.5656627	22.96616657	42.93917994	35.33253502	44.51321756	-1.273518	0.004424
Tspan31	ENSMUSG00000006736	22.85321673	14.59310289	31.82661408	62.80444261	51.45289153	58.80388004	-1.320919	0.004448
Mest	ENSMUSG00000051855	20.5695236	3.95538984	29.3052356	91.32717178	91.70141704	66.08986685	-2.210346	0.004468
Ube2e1	ENSMUSG00000021774	6.096460401	2.698697171	6.069263673	14.78267924	13.02083207	17.69522001	-1.613963	0.004474
H3f3a	ENSMUSG00000060743	36.43678575	32.59495025	13.51746304	76.28701365	66.29622334	72.68367651	-1.3828	0.004474
Tspan3	ENSMUSG00000032324	188.2367267	105.132845	167.4372986	382.8596113	305.5517464	368.1447081	-1.197135	0.004483
Ass1	ENSMUSG00000076441	32.6562242	16.47413451	24.35463412	71.46882768	102.9105972	106.7192054	-1.935555	0.004634
Marchf5	ENSMUSG00000023307	28.76728963	27.73530735	12.50790758	72.48387396	55.45037039	64.49420345	-1.479434	0.00464
Mageh1	ENSMUSG00000047238	8.089006359	0	0	35.56620856	40.89885751	24.69447123	-3.644526	0.004792
Tuba1a	ENSMUSG00000072235	281.1838209	154.9991885	242.6913688	836.354945	1363.640028	1149.029791	-2.302525	0.004839
Pcp4	ENSMUSG00000090223	28.93551939	182.46787	77.27379148	583.9017004	442.1800966	417.6685376	-2.322292	0.005014
Zfp64	ENSMUSG00000027551	1.037317014	0.006103481	0	8.093245611	7.644657458	4.586380384	-4.283812	0.005101
Slit1	ENSMUSG00000025020	3.637268315	0	0	8.214286472	10.23459966	11.81085371	-3.056472	0.005145
Mrgpre	ENSMUSG00000048965	1.159512384	0.297721991	0.68454626	9.141363539	11.02806302	6.123641569	-3.6178	0.005148
Fam221a	ENSMUSG00000047115	1.123854739	0	1.920045186	5.075847485	4.63333606	3.985191931	-2.169591	0.005187
Ppp2r1a	ENSMUSG00000007564	123.7577379	32.30822083	89.60252347	313.2332288	249.4826838	249.417535	-1.725004	0.005208
Ptprk	ENSMUSG00000019889	0	0.032521369	0	3.882715595	3.331949316	2.014446517	-8.14866	0.005225
Dnajc17	ENSMUSG00000034278	6.155153247	0.027614985	4.42200653	21.70771394	29.16291714	18.8265659	-2.716387	0.005316
Actg1	ENSMUSG00000062825	109.0547034	83.88684383	93.59739341	247.5673948	339.5997798	387.9585448	-1.766857	0.00537
Wbp2	ENSMUSG00000034341	116.7179411	37.78032424	91.83941	224.8589668	295.4367496	243.2277725	-1.632035	0.0054
Ankrd54	ENSMUSG00000033055	2.254703267	0	4.443627811	17.51934239	25.87139627	16.48560284	-3.160112	0.005409
Susd2	ENSMUSG00000006342	0.009765818	0	0	18.24040876	11.64794972	22.7157434	-12.39515	0.005492
Ndn	ENSMUSG00000033585	139.4766224	103.7263687	106.7020535	580.6948981	518.4399029	357.0137604	-2.057122	0.005498
Gm31282	ENSMUSG00000115591	0.658516107	0.417094691	0	12.38930668	9.348062401	6.636566135	-4.721338	0.005543
Atp6v0c	ENSMUSG00000024121	145.0172476	47.98975167	206.7212517	367.442166	436.4257781	433.7276429	-1.630449	0.005571
Atp11a	ENSMUSG00000031441	0.505440207	0.194369867	0	4.763426438	2.587475497	3.995677249	-4.01915	0.005591
Clu	ENSMUSG00000022037	160.7787096	97.84141011	129.0157285	295.4709946	345.2610637	256.5873557	-1.21092	0.00568
Gm49932	ENSMUSG00000117220	0.524149769	0	0	4.965479059	7.171870233	3.969307734	-4.941534	0.0057
Cers2	ENSMUSG00000015714	5.329331272	0.304606205	0.642809928	18.19752737	25.04930941	31.79408719	-3.579589	0.005702
BC004004	ENSMUSG00000052712	3.471740699	1.811669711	3.238128124	5.652641245	6.072339467	6.813185283	-1.121313	0.005745
Stmn1	ENSMUSG00000028832	212.9498881	660.5598071	442.6874542	1350.618443	1123.922174	1193.569308	-1.478661	0.005749
Hars2	ENSMUSG00000019143	4.94677828	5.841392145	2.044055436	10.92309552	14.82374306	12.87250861	-1.589552	0.005886
Pxmp4	ENSMUSG00000000876	3.748832257	0	5.334399955	11.12633746	12.17466094	11.62607015	-1.943068	0.005886
1700086L19Rik	ENSMUSG00000071265	0.062410391	12.96974373	1.246689805	61.84108976	111.8127389	82.26268751	-4.163722	0.005919
Ccnd2	ENSMUSG00000000184	1.294191458	2.426197574	0.38233141	4.608474432	6.012117206	5.015910808	-1.930265	0.006027
Pou6f2	ENSMUSG00000009734	0.004802518	0	0	6.203507148	3.40537562	3.975871417	-11.46591	0.006076
Mtch1	ENSMUSG00000024012	92.54160547	23.87227167	61.66124736	225.1312498	195.555783	167.9304054	-1.724844	0.006093
Tmem255a	ENSMUSG00000036502	13.83662	11.56013701	4.147351619	50.83433038	51.6931135	33.22432472	-2.200029	0.006126
Abca8b	ENSMUSG00000020620	2.24149404	0	0	5.03491271	4.456593362	5.504530061	-2.742049	0.0062
Mgrn1	ENSMUSG00000022517	25.99502592	12.43302946	15.53686508	38.62269781	47.7156287	46.41230126	-1.298625	0.006237
Cdk18	ENSMUSG00000026437	24.11705065	7.551620209	28.88675698	69.86575044	66.76884731	53.38958768	-1.649855	0.006261
Cops3	ENSMUSG00000019373	22.40580922	8.826177716	7.290530885	40.80228761	45.78572755	37.21407077	-1.684262	0.00627
Nipsnap1	ENSMUSG00000034285	17.10837509	11.48107853	31.66016454	54.91347414	53.72575248	66.90914903	-1.542845	0.006357
Cnpy4	ENSMUSG00000036968	0.013983153	0	0.573347119	9.253539742	5.457522358	5.595778069	-5.11165	0.006391
Grik3	ENSMUSG00000001985	2.456196308	0.217374525	0.978689558	4.640822748	5.747334971	4.852889887	-2.061101	0.006412
Gmcl1	ENSMUSG00000001157	1.425932776	3.838453133	1.238433581	7.161992559	7.839911722	6.305872222	-1.712243	0.00648
1700001L19Rik	ENSMUSG00000021534	7.16471973	10.04882987	8.363159868	31.3914966	20.48859625	24.85806041	-1.585113	0.006495
Hhat	ENSMUSG00000037375	0.010998553	0.003947585	0.408571479	2.477973547	1.565599171	2.911343052	-4.037539	0.006508
Gps1	ENSMUSG00000025156	57.64154566	30.77573528	32.27916042	88.52432192	114.7319235	119.106022	-1.4173	0.006518
H2-T22	ENSMUSG00000056116	6.111702985	0.518424023	4.390446494	13.90770018	14.42261925	11.66120546	-1.859495	0.006534
Prdx1	ENSMUSG00000028691	45.7379668	33.60396095	11.25383525	83.49039866	104.3992818	114.2447201	-1.737675	0.006537
Pithd1	ENSMUSG00000028669	27.11274185	24.29414084	24.57525655	80.99437011	84.5706654	55.14411118	-1.538414	0.006608
Camkv	ENSMUSG00000032936	10.14479829	18.71772005	14.68649135	34.07286257	48.72787003	38.34996533	-1.476091	0.006666
Bag3	ENSMUSG00000030847	1.806905057	8.670587888	4.143531509	22.09584255	28.19118773	18.42104012	-2.232435	0.006667
Gxylt1	ENSMUSG00000036197	0.978121794	1.037077842	0.010493365	2.328046975	2.403678903	2.602425455	-1.856214	0.006683
Gm18588	ENSMUSG00000093402	1.022731256	0	0	2.28915299	3.453331694	2.728510485	-3.050104	0.006694

Adap2	ENSMUSG00000020709	0.526205258	0	0	5.429575256	2.818200892	4.888390214	-4.641775	0.006705
Sulf2	ENSMUSG00000006800	8.547810501	0.024609368	0.708848137	26.28139467	28.25291527	18.23515004	-2.970939	0.006729
Ociad1	ENSMUSG00000029152	48.04652357	34.43718307	64.21178248	93.81675775	98.05878167	112.0388124	-1.05084	0.006839
Csf2ra	ENSMUSG00000059326	7.758365925	0.704542813	2.334015221	19.68674089	16.09446296	14.78727234	-2.227618	0.006863
Eif4a3	ENSMUSG00000025580	26.226261	29.54995989	15.10781921	78.57975548	53.78673723	75.4798965	-1.551985	0.006946
Sgsh	ENSMUSG00000005043	0.007599815	0	0	3.243180549	4.619046289	2.328490696	-10.389	0.006987
Gm38198	ENSMUSG00000102465	0.121779561	0	0	2.367107802	4.819019236	3.830753289	-6.4993	0.007027
Gabrg1	ENSMUSG00000001260	0.183309209	0.151477088	0.770889583	3.129952907	1.994043292	3.475912232	-2.959393	0.007032
Cst6	ENSMUSG00000024846	1.328700441	0.123639512	1.991920253	4.166782787	3.97493229	3.795064553	-1.793148	0.007081
Sptssa	ENSMUSG00000044408	16.79489114	13.37263831	9.874166226	40.22089901	27.78402463	34.57214212	-1.357133	0.00714
Gaa	ENSMUSG00000025579	142.6406255	53.73881839	123.2580399	304.1246689	279.6801571	238.399753	-1.363061	0.007183
Dpagt1	ENSMUSG00000032123	6.405492765	0.200375943	1.184187684	18.20712964	12.89781577	19.89143892	-2.710689	0.007255
Minar2	ENSMUSG00000050875	4.874681073	10.09236544	12.94112686	33.56296254	46.95929328	30.83352074	-1.996417	0.007261
Eid2	ENSMUSG00000046058	10.07331509	2.231576454	7.119514194	46.69342395	41.020864	26.94257094	-2.56138	0.007291
Scrn2	ENSMUSG00000020877	6.610934839	2.590096995	0.017203999	14.47226616	13.21204079	18.17187983	-2.314554	0.007337
1500011B03Rik	ENSMUSG00000072694	92.50260515	68.56572083	98.92906458	198.7860066	193.206746	147.8483123	-1.054038	0.007394
Crip2	ENSMUSG00000006356	19.30589181	0	28.48003637	224.8516377	183.2376527	119.0972403	-3.463656	0.007427
Nop58	ENSMUSG00000026020	1.072660467	0.45662525	2.29154775	6.807654873	5.682477827	4.433734428	-2.1471	0.00745
Lrrc8b	ENSMUSG00000070639	4.583466071	0	1.436736005	10.55370571	13.40801892	9.324489863	-2.467041	0.007453
Pik3c2b	ENSMUSG00000026447	3.665113979	1.024264895	1.419258194	9.872292565	6.612885379	8.410287955	-2.026961	0.007458
Mrap2	ENSMUSG00000042761	7.936793307	0	0	14.86521492	19.39532348	19.31188826	-2.754863	0.007465
Gm15520	ENSMUSG00000085449	0	0	0.117267493	2.636176445	3.600070007	5.230779141	-6.611544	0.007545
5530401A14Rik	ENSMUSG00000020703	0.789221112	0.095304486	0	10.49208366	5.947970342	6.383022715	-4.689446	0.007605
Prr36	ENSMUSG00000064125	2.146752845	0	0	19.19746281	13.59552852	10.13819325	-4.321798	0.007646
Doc2g	ENSMUSG00000024871	0.773703777	1.273437506	1.162049372	3.554667742	2.392935647	2.805938151	-1.447657	0.007677
Cyb561d2	ENSMUSG00000037190	16.33095612	1.061288357	16.25838935	42.68605662	35.45782769	37.46674077	-1.780568	0.007745
Mrpl2	ENSMUSG00000002767	72.36671542	30.84126485	65.92440167	118.4527003	146.6407214	130.3344744	-1.225262	0.007821
Adgrg1	ENSMUSG00000031785	4.29452797	2.37870926	3.239911975	10.77068051	8.363959844	13.2561896	-1.70817	0.007824
Lix1	ENSMUSG00000047786	3.331113731	1.472584752	0.677839574	5.946689404	7.324532021	5.839401771	-1.801722	0.007833
Tmeff1	ENSMUSG00000028347	14.92377152	20.92971016	19.77177542	40.87067578	39.88851928	30.56207377	-1.000917	0.007866
Oaz1	ENSMUSG00000035242	253.7032413	137.7743337	287.2331422	521.8370149	435.2894185	486.3215276	-1.08865	0.007912
Vps39	ENSMUSG00000027291	3.512443029	4.37390553	2.131256616	8.052856441	11.14612762	12.49993912	-1.661896	0.007936
Erp29	ENSMUSG00000029616	11.07818884	3.731644002	12.40615324	23.6322931	26.96414839	21.77647257	-1.410996	0.00826
Tusc3	ENSMUSG00000118664	20.11136665	34.03156621	12.21439231	50.6749784	61.03688643	57.697182	-1.352183	0.008291
Gm12371	ENSMUSG00000084898	6.856705315	0	0.032326488	32.49097574	40.55146472	21.26381241	-3.77498	0.008495
Cacng7	ENSMUSG00000069806	0.852038624	0	0	2.525526212	1.951647948	3.282512127	-3.187008	0.008588
Galntl6	ENSMUSG00000096914	1.615291869	0.194876721	0	3.425977962	5.792066732	5.018937524	-2.975447	0.008641
Qsox2	ENSMUSG00000036327	2.918263656	0.163335143	0.03797179	16.19140667	18.09197972	9.358544199	-3.806296	0.008641
Chodl	ENSMUSG00000022860	0.421534398	0	0	16.90544606	12.21308986	8.075404677	-6.463273	0.008659
Cacna1h	ENSMUSG00000024112	0.343599152	1.287503769	1.251191087	3.521527018	5.99886105	5.955533781	-2.424736	0.008662
Btbd11	ENSMUSG00000020042	0.555025131	0	0.601102555	6.100220993	6.355421259	3.257222281	-3.764574	0.008668
Amigo2	ENSMUSG00000048218	4.337699761	0.433947269	0.011150237	43.92833431	34.15709896	21.51257559	-4.38019	0.008874
Rbm4b	ENSMUSG00000033760	4.642394344	4.984246035	2.03925749	9.845841381	7.955550573	9.509251406	-1.227166	0.00895
Ctxn1	ENSMUSG00000048644	25.65739066	3.486870324	27.44052072	313.0139367	236.3624866	157.2464568	-3.642454	0.008975
Dhps	ENSMUSG00000060038	21.88257623	1.460980046	8.073443013	38.18317141	38.50375682	41.80643959	-1.915189	0.009052
Spef1	ENSMUSG00000027329	0.097693732	0	0.025677611	3.981292003	5.032950493	2.321270114	-6.521699	0.009093
Pag1	ENSMUSG00000027508	0.003501627	0	0	2.815348798	1.283199194	2.408451648	-10.85975	0.009097
Nhlh2	ENSMUSG00000048540	0.966053404	0.046675799	0.175787587	2.943606579	5.344341014	5.622358531	-3.54892	0.009143
Kctd16	ENSMUSG00000051401	18.32127302	0.072718666	10.91417357	32.63901174	38.98667703	37.5349699	-1.897078	0.009153
Spock3	ENSMUSG00000054162	14.27601566	17.09236937	17.7332655	82.95938739	60.99759319	48.52171436	-1.970855	0.009161
Trim23	ENSMUSG00000021712	2.358172337	0.553087737	1.914682079	6.085512165	4.214309575	5.445507767	-1.706041	0.009219
Rhog	ENSMUSG00000073982	16.27669592	0.203495213	0	34.62696009	59.68455824	50.69553266	-3.137318	0.00925
Baiap2	ENSMUSG00000025372	13.9706073	6.873874251	2.070028473	27.60798441	36.90487815	26.27810575	-1.986287	0.009272
Mutyh	ENSMUSG00000028687	3.185032591	0	0	6.059327262	5.918041811	7.259488367	-2.594493	0.009462
Msantd1	ENSMUSG00000051246	0.011547534	0	0	6.529653895	3.96792252	8.722310712	-10.7008	0.009586
Yipf3	ENSMUSG00000071074	17.68835164	0.147539697	14.20943011	70.46987046	42.25856348	55.50348086	-2.392266	0.009635
Wrap53	ENSMUSG00000041346	3.904432807	0	0.051311637	18.70014441	10.97019467	12.22718723	-3.404844	0.009683
Ube2d1	ENSMUSG00000019927	9.608573019	0.644564452	3.566356372	17.73157084	18.72654123	15.86473414	-1.920736	0.009776
Ogfod3	ENSMUSG00000025169	5.154940844	0	0	32.90146204	17.11570333	33.6440675	-4.020531	0.009839
Gdap2	ENSMUSG00000027865	4.975158756	1.19045124	0	11.35091762	13.05755824	8.776950616	-2.428234	0.009868
Tsc22d1	ENSMUSG00000022010	64.71530451	79.73074346	91.88004862	191.5957798	141.936635	147.0611862	-1.024038	0.009868
Fig4	ENSMUSG00000038417	5.079876856	6.768447949	1.37548123	11.22506531	14.89103068	13.80466685	-1.594002	0.009919
Kcnn3	ENSMUSG00000000794	0.121972274	0.003456167	0	1.508725078	2.613901547	3.319620312	-5.890802	0.00992
Smchd1	ENSMUSG00000024054	1.413541324	0.153649986	0.259454768	3.641404014	3.004339814	2.466832511	-2.318662	0.009987
Ephx4	ENSMUSG00000033805	0.122280383	0	0.235692699	13.01861522	8.439941672	18.32482395	-6.796171	0.010031
Ubl7	ENSMUSG00000055720	45.09521007	14.46620527	49.49587714	100.242811	82.39858817	94.67758153	-1.34646	0.010088
Tmem106c	ENSMUSG00000052369	24.72505335	0.192919147	2.883472262	48.68313538	41.7047096	50.70640793	-2.343427	0.010128
Ttpal	ENSMUSG00000017679	1.290865234	0	0.829700517	3.097824136	3.997067502	5.322280419	-2.549816	0.010135
Rtn4r	ENSMUSG00000043811	7.858460103	0	6.787945311	15.93566792	23.46454298	20.41261627	-2.029908	0.01023
Trp53i11	ENSMUSG00000068735	2.109099123	0	0.057346664	81.88742011	60.50778827	37.13048018	-6.372716	0.010279
C030006N10Rik	ENSMUSG00000112409	0	0	2.796897747	8.255212818	6.057180725	10.62971215	-3.156684	0.010283
Ftl2-ps	ENSMUSG00000082062	0.850793827	2.224807263	2.555666553	6.604232567	8.751089906	11.70367881	-2.264577	0.010368
Dyrk1b	ENSMUSG00000002409	4.518384945	0.040208238	0	11.15155704	8.183374775	12.81096419	-2.817974	0.0104
Tmc4	ENSMUSG00000019734	0.493778474	0	0.519134696	3.566315694	5.176200584	2.636630444	-3.48981	0.010484
Disp3	ENSMUSG00000041544	3.477661585	0	0.097068436	11.11579974	6.711800807	11.23820901	-3.023417	0.010606
Gm32710	ENSMUSG00000109590	0.651370586	1.227392242	1.141365645	11.70193485	6.57664768	7.05968975	-3.068636	0.01061
Slc35b1	ENSMUSG00000020873	36.80507526	0.796176164	9.899845172	72.64967402	68.39736867	60.35034375	-2.084012	0.010809
Pgap1	ENSMUSG00000073678	0.339272669	0	0.053079583	1.998382276	2.518993706	3.977640522	-4.436395	0.010914
Pou6f1	ENSMUSG00000009739	1.901994613	0.103958556	1.233295757	3.424913114	3.998314575	3.362587816	-1.735404	0.011047
Xbp1	ENSMUSG00000020484	48.32505946	19.11667736	38.1674007	73.84988861	83.10161031	98.56124584	-1.274661	0.011119
Rhno1	ENSMUSG00000048668	0.107345873	0	0.00705364	1.671611802	3.591302452	2.374418037	-6.060916	0.011131
Fbh1	ENSMUSG00000058594	6.997681213	1.913710357	1.413485958	11.72213097	11.99712569	15.49269857	-1.925169	0.011135
Cops7a	ENSMUSG00000030127	72.25107782	21.6109514	34.00629682	134.0565749	107.0954685	116.0418349	-1.482048	0.011249
Adcy3	ENSMUSG00000020654	0.445770665	0	0	1.342891053	2.784208431	2.38433418	-3.868602	0.011264
Pnck	ENSMUSG00000002012	66.75077886	18.71077051	114.5724376	227.7398227	386.5563052	300.5094028	-2.19322	0.011287
Phyhipl	ENSMUSG00000037747	10.47179283	5.433648377	8.327817507	18.74790895	18.8412428	14.35001802	-1.099835	0.011334

Kcnk4	ENSMUSG00000024957	1.773070125	0	0.363345381	5.901149572	4.155917839	3.645448891	-2.681177	0.011457
Slc1a4	ENSMUSG00000020142	2.845308169	0.082802752	1.500201003	8.500498989	12.30413048	7.020302496	-2.651549	0.011507
Tapbp	ENSMUSG00000024308	4.077619369	1.17480905	1.42900363	16.15086235	10.10086642	10.01362596	-2.440363	0.011551
Nup85	ENSMUSG00000020739	10.65332469	1.643291475	8.54355176	31.62608206	21.53385904	22.90256083	-1.867818	0.011567
Adcyap1	ENSMUSG00000024256	21.35122764	21.21129145	22.30757904	65.32713049	40.450164	54.57942032	-1.305659	0.011569
Amfr	ENSMUSG00000031751	17.66582609	13.63693718	6.640527196	34.85322027	35.43494702	26.10252469	-1.345049	0.011569
R3hdm4	ENSMUSG00000035781	5.526851429	0.187588039	3.202180624	10.08473559	12.79123838	9.909235216	-1.878476	0.011626
Cdh22	ENSMUSG00000053166	4.775398001	0	0	8.702608658	10.57388721	13.50767468	-2.779307	0.011633
Gm48261	ENSMUSG00000113073	3.236244215	0	0.621949557	10.02298063	6.021836698	10.3027629	-2.771673	0.011637
Asb5	ENSMUSG00000031519	1.112254151	0	0	2.882613426	2.076632914	2.132779665	-2.672711	0.011665
Dlg1	ENSMUSG00000022770	1.134225729	0	2.010444992	4.31768118	4.657698908	3.372166769	-1.973244	0.011712
Pld3	ENSMUSG00000003363	104.2735376	28.96796807	19.60014447	255.4851478	206.5407479	170.5580333	-2.049219	0.011738
Opcml	ENSMUSG00000062257	0.616893123	0.119414506	0.340074921	9.667881381	8.769749709	4.357178859	-4.404443	0.011743
Gm32647	ENSMUSG00000108532	0.005374173	0	0	10.62655863	9.729625527	4.556660593	-12.17856	0.011791
Lingo2	ENSMUSG00000045083	7.924472338	11.36680308	7.281478113	16.77860287	22.82667209	25.86219009	-1.30083	0.011817
Nradd	ENSMUSG00000032491	1.569276137	0	0	4.518363498	6.802243957	3.855691653	-3.273649	0.011833
Ttc39c	ENSMUSG00000024424	2.369614879	0.039945148	3.212848046	9.820777107	6.309214371	7.776622632	-2.08815	0.011884
Aven	ENSMUSG00000003604	1.348262872	0.043335813	0.028208917	3.081788384	2.19831694	3.1624615	-2.571986	0.011956
Nxt1	ENSMUSG00000036992	11.42233188	6.052432663	0.75845588	22.41478496	39.85201566	37.40241439	-2.450579	0.012285
Rgs4	ENSMUSG00000038530	78.20084451	94.3584246	84.6983203	293.7541515	265.322308	173.5584338	-1.509881	0.01232
Letmd1	ENSMUSG00000037353	3.598866165	2.749830349	2.481216353	11.77713322	14.06947992	7.676996658	-1.924706	0.012325
9330179D12Rik	ENSMUSG00000097166	3.792502229	0	0.023733646	36.69909362	20.62539332	19.73327271	-4.335718	0.012473
Sez6l	ENSMUSG00000058153	3.199817764	2.338602522	2.957415651	16.04971548	12.16774846	8.535304473	-2.113025	0.012533
Zfp236	ENSMUSG00000041258	1.623483361	2.613522662	0.669947641	4.970872187	3.797012774	5.005590004	-1.488993	0.012654
Nmnat2	ENSMUSG00000042751	9.765995483	12.74617135	13.82420648	60.19985309	32.78432041	46.57335184	-1.941374	0.012673
Setd6	ENSMUSG00000031671	12.70959902	0.417576882	0.111197683	31.9435513	26.41701073	21.33916004	-2.589849	0.012686
Taf10	ENSMUSG00000043866	7.896092152	2.182668167	3.169829352	19.28054718	14.63686538	12.85797174	-1.819911	0.012707
Plp2	ENSMUSG00000031146	0	0	0.951765475	5.909927209	6.518053171	11.44685827	-4.648741	0.012757
Echdc2	ENSMUSG00000028601	0	0	2.729545746	35.12039979	17.87358291	41.65735786	-5.11589	0.012806
Dnajc30	ENSMUSG00000061118	14.66230876	5.110351634	4.182922992	23.87496842	27.49799594	21.3163855	-1.601382	0.01289
Rnf215	ENSMUSG00000003581	10.41536315	0	2.923215371	25.4158166	20.7469295	17.21638773	-2.248403	0.012948
Fibp	ENSMUSG00000024911	17.36124044	9.333798087	24.01275354	50.7648178	39.62595026	36.94679151	-1.328379	0.013026
Gstp1	ENSMUSG00000060803	132.5137296	162.5884684	165.8379951	392.6144879	440.3154981	273.7916179	-1.263646	0.013146
Ddost	ENSMUSG00000028757	103.500789	52.54495964	43.08709147	157.4895202	142.3723186	144.9876738	-1.159586	0.013299
Mapre3	ENSMUSG00000029166	59.54608069	9.262444361	37.58270581	116.283371	127.2673384	91.45745883	-1.654817	0.013332
Rab36	ENSMUSG00000020175	1.813274875	1.174979844	2.916661174	7.957644171	8.955393189	14.15561087	-2.395471	0.013477
Rae1	ENSMUSG00000027509	29.42134332	0.014142678	10.90911384	59.70638966	45.39328077	58.04788541	-2.01573	0.013482
Atf4	ENSMUSG00000042406	37.99841309	35.46171613	46.15676859	101.233476	133.6959528	81.3042318	-1.40257	0.013697
Actr10	ENSMUSG00000021076	6.853287866	4.170595261	14.3596613	28.507941	20.16475695	26.43602218	-1.565087	0.013716
Slbp	ENSMUSG00000004642	9.84393983	10.24877717	5.148599737	17.44847597	16.88204855	23.01220848	-1.183823	0.013743
Mettl24	ENSMUSG00000045555	1.535769106	0	0	2.442414584	3.660454539	3.347288124	-2.621377	0.013748
Prmt2	ENSMUSG00000020230	29.9638176	67.62559413	14.61424016	137.1646617	107.5503407	104.5979458	-1.6384	0.013828
Rasa4	ENSMUSG00000004952	1.241433146	0	3.576294269	5.804950094	8.131674083	9.320542605	-2.271251	0.013948
Sec22a	ENSMUSG00000034473	2.793859766	0	0	5.720010566	7.992161778	10.76280629	-3.130976	0.01395
Vstm5	ENSMUSG00000031937	4.464882008	1.396921558	0.177138114	14.1865883	11.39486208	7.985621125	-2.474679	0.01403
Pigt	ENSMUSG00000017721	23.98474543	0	25.49993185	52.49766694	50.1618133	50.53467123	-1.630307	0.014035
Sult2b1	ENSMUSG00000003271	0.093418085	0.329198585	9.553663877	23.07301504	18.15850774	15.86697923	-2.516879	0.014085
Ssr2	ENSMUSG00000041355	49.00462902	21.49008793	25.99705184	84.87679778	75.74400511	64.74924449	-1.223818	0.014102
Prelid1	ENSMUSG00000021486	60.33529135	39.69798944	54.41600975	131.3944823	89.21342851	136.9327409	-1.210974	0.014134
Prpsap2	ENSMUSG00000020528	16.61891377	0	0.251658908	43.4607467	33.22959466	28.394866	-2.638979	0.014141
Lrrc55	ENSMUSG00000075224	1.336849086	0.719729941	0.397679717	9.40338178	11.38270941	5.093198499	-3.398439	0.014146
Tmem222	ENSMUSG00000028857	87.32235707	37.32862135	91.10217606	202.8528678	179.4625013	142.0841526	-1.281285	0.014216
Tstd2	ENSMUSG00000035495	0.132496028	0.479876054	0	4.240913857	1.913938613	4.240274295	-4.085355	0.014437
Mdga2	ENSMUSG00000034912	2.027078338	1.280446127	1.944597983	4.734467026	4.186114428	3.068067633	-1.190697	0.014548
Exoc7	ENSMUSG00000020792	10.61598322	4.185235296	4.29646933	20.78156832	14.44874092	20.74967557	-1.551513	0.01474
Ptma	ENSMUSG00000026238	97.97513597	44.32284411	76.44659426	227.6813089	180.2674158	149.4391382	-1.349434	0.014945
Agpat4	ENSMUSG00000023827	0.409849801	1.002972981	4.465651189	34.90562428	51.39633337	22.69166604	-4.212658	0.015027
6330403K07Rik	ENSMUSG00000018451	91.48795963	77.22371654	116.7908745	1193.435094	959.2795236	526.4524816	-3.230209	0.015137
Fads3	ENSMUSG00000024664	0.00884641	0.038101688	0	19.44636227	21.11147918	8.363618571	-10.02519	0.015197
Cpsf4	ENSMUSG00000029625	3.915827066	0	3.166100081	9.777906605	6.879563378	8.243260155	-1.813974	0.015356
Dbpht2	ENSMUSG00000029878	0.332880737	2.292607319	2.760177297	6.159659786	8.37072663	11.38116079	-2.266399	0.015373
Med19	ENSMUSG00000027080	7.250017199	10.26247409	6.097852666	13.67488187	20.53234234	17.97020328	-1.144007	0.015548
Calr	ENSMUSG00000003814	49.01382726	46.59280799	30.2106408	92.39392952	67.51968799	96.12279535	-1.025019	0.015613
Neu1	ENSMUSG00000007038	20.16509327	3.067282852	5.621371032	29.05097712	32.77011035	36.11841086	-1.763132	0.015627
Trpv2	ENSMUSG00000018507	3.844466174	0.096643211	2.933118843	11.10865786	11.28310287	6.809413124	-2.086757	0.01576
Gtf2ird2	ENSMUSG00000015942	2.423939059	3.845074868	0.091014706	8.657312325	6.340500468	10.13586635	-1.982517	0.015801
Prdm15	ENSMUSG00000014039	1.129122702	0.076384669	0	1.969110439	2.415880567	3.319399255	-2.67604	0.015871
Ankra2	ENSMUSG00000021661	8.255812274	3.444873815	3.081391039	13.19759649	12.64423377	10.82339155	-1.310563	0.015885
Ppil3	ENSMUSG00000026035	4.628055567	0.816518021	1.413686816	7.501146293	6.537125131	7.860284866	-1.674921	0.015893
Ap3d1	ENSMUSG00000020198	15.75295262	7.135744322	6.326435863	29.1763406	27.12175038	20.64128589	-1.397006	0.015955
Cops8	ENSMUSG00000034432	27.5574569	21.12166392	27.93387209	65.75377436	44.10725159	72.1407468	-1.248292	0.016031
Gabra2	ENSMUSG00000000560	10.00220459	6.727660941	9.223280164	18.39604723	20.83027981	13.89210042	-1.033303	0.016032
Alg12	ENSMUSG00000035845	0.199337094	0	0.134725716	3.585303282	2.531109687	1.495700888	-4.510106	0.016092
Ap4m1	ENSMUSG00000019518	0	0	0.730231305	1.713847642	2.547834832	3.591762648	-3.4269	0.016282
Stk3	ENSMUSG00000022329	0.548668387	0.005907818	1.245981495	2.90858755	2.593670124	1.893757645	-2.038304	0.016294
Zswim1	ENSMUSG00000017764	6.549893118	0.974120827	1.306229571	12.06428985	19.02316305	23.8038257	-2.636052	0.016393
Olfm2	ENSMUSG00000032172	41.99177194	9.243569601	24.12530046	108.0220712	82.41743297	66.99542668	-1.772325	0.016428
Ccdc107	ENSMUSG00000028461	34.02263941	25.84698326	41.44640255	83.41697482	57.45511956	66.13970265	-1.030851	0.016454
Tmem53	ENSMUSG00000048772	1.214142605	0	0	7.128467564	12.25226574	17.36319424	-4.919496	0.016519
Fxyd6	ENSMUSG00000066705	6.583331305	1.178508312	10.11588453	46.28716453	80.57289232	41.28690783	-3.233488	0.016539
Fnbp1l	ENSMUSG00000039735	2.260586462	0.598309798	1.473307901	7.962834212	7.854855644	4.223558297	-2.209799	0.016551
Tubb4a	ENSMUSG00000062591	544.7544066	72.97034185	280.5942932	1074.861479	1373.071523	858.2977951	-1.879888	0.016559
Urod	ENSMUSG00000028684	24.76754322	19.76793809	9.718831986	47.56345529	46.49371295	71.80702973	-1.612193	0.016563
Coq8b	ENSMUSG00000003762	1.843625968	0	0	2.891691082	3.166845025	3.161709149	-2.322259	0.016704
Gm9234	ENSMUSG00000058050	2.386372931	4.845419549	3.369116516	8.614444146	6.16182714	8.35606336	-1.125723	0.016762
Sypl	ENSMUSG00000020570	2.496322158	0.168807143	0.152145613	4.734347514	5.760672837	3.780352094	-2.341156	0.016766

Jak2	ENSMUSG00000024789	1.800657655	0	0	3.34685329	6.053568092	6.872266269	-3.175857	0.016883
Glt8d1	ENSMUSG00000021916	14.43134902	4.456765089	8.862048058	21.31908228	20.27273471	20.42758229	-1.160223	0.017058
Slc16a11	ENSMUSG00000040938	6.094421116	0	4.377454542	26.55164617	23.33579865	45.71229912	-3.190487	0.017074
Il34	ENSMUSG00000031750	21.8328847	0	20.05552478	76.34298468	43.50100848	77.77717331	-2.238114	0.017076
Bex1	ENSMUSG00000050071	22.93884451	47.44990188	58.3144495	390.4485965	312.4989423	175.6912863	-2.771222	0.017125
Fam110a	ENSMUSG00000027459	2.06714979	7.250266118	6.200299497	11.36120391	13.79675918	11.32882004	-1.233458	0.01717
Rbm19	ENSMUSG00000029594	1.772579218	0.588324701	1.683254925	10.62607841	6.063490288	5.96687261	-2.48601	0.017283
Ubxn6	ENSMUSG00000019578	1.528286115	0.006353632	0.297778984	5.286446127	2.877294028	3.637953986	-2.687173	0.017424
Tmem132a	ENSMUSG00000024736	1.261278599	0	0.069791949	7.605131447	4.124023772	4.08624142	-3.570671	0.017469
Zmat2	ENSMUSG00000001383	52.71264217	22.29654338	26.7504931	115.5467263	91.49918314	74.04519014	-1.465872	0.017511
Phc1	ENSMUSG00000040669	2.070871504	0.23184701	0.053265167	5.696054419	5.301891341	3.183734412	-2.589627	0.017619
Nkain1	ENSMUSG00000078532	0.258766786	0	0	5.175307346	13.4566259	9.590446479	-6.769043	0.017635
Asic2	ENSMUSG00000020704	2.753507228	2.126224313	0.016542307	9.216852827	14.5555587	20.55237405	-3.178357	0.017665
Cpne5	ENSMUSG00000024008	0.306510395	0	0.647099065	7.910299376	8.118121849	3.310688893	-4.341979	0.017862
Gm7908	ENSMUSG00000115602	0.04466789	0.288578431	0.328731543	5.48568907	4.923979804	2.178539414	-4.249146	0.017897
Arhgap24	ENSMUSG00000057315	3.105790076	0.924373301	3.674439235	13.02584071	8.089904989	16.48069056	-2.286803	0.018001
Scpep1	ENSMUSG00000000278	5.406966045	0.06889919	0.117728845	8.550928381	13.40840864	10.08260741	-2.518114	0.018013
Rnf19b	ENSMUSG00000028793	0	2.465041244	0.184200201	5.655258324	11.77375049	7.873087553	-3.255606	0.018148
Blmh	ENSMUSG00000020840	14.4409445	18.84809208	6.627380013	27.86754218	25.85534994	31.17650919	-1.088772	0.018314
Btbd17	ENSMUSG00000000202	0	0.793356768	0.022132537	3.161936751	4.317190466	1.90865173	-3.525046	0.018351
Ptpro	ENSMUSG00000030223	2.89900119	1.767605487	0.188852468	29.94710381	18.750862	13.31594692	-3.674912	0.018424
Rgs17	ENSMUSG00000019775	9.443539048	15.88374796	3.102649416	29.18489296	32.37102495	22.01686504	-1.555622	0.018559
Btg3	ENSMUSG00000022863	1.420714869	2.348635892	0.246016332	3.403924212	3.983795992	4.483536147	-1.563869	0.018615
Fbxl5	ENSMUSG00000039753	3.76096294	1.628013934	1.010249216	7.06881438	6.409790732	10.56954648	-1.909957	0.018634
Pdgfa	ENSMUSG00000025856	6.786554748	6.994780945	13.98741951	25.98001875	20.17668456	18.70908322	-1.223995	0.018921
Uap1l1	ENSMUSG00000026956	5.578570665	0	0.849001092	11.6704807	8.273385758	9.367064966	-2.189093	0.018985
Mast1	ENSMUSG00000053693	2.934394742	0	1.223111387	8.837864179	15.57667934	8.255074889	-2.974159	0.019047
Cyb561	ENSMUSG00000019590	21.80968104	0.079102984	13.97018427	36.52684344	42.99056246	34.48628488	-1.668675	0.019071
D630023F18Rik	ENSMUSG00000044816	1.119443975	0.013543457	0.105791371	11.10193799	4.734230679	6.968213715	-4.202321	0.019332
Tnfaip1	ENSMUSG00000017615	9.271392893	4.805226485	1.968153893	13.86683923	22.21259967	25.03071005	-1.929309	0.019356
Lhfpl5	ENSMUSG00000062252	7.57163753	0.121006366	16.05382399	35.90628188	33.45400192	58.51686643	-2.428974	0.019623
Dok5	ENSMUSG00000027560	0	0	0.159584608	9.002193625	8.290937418	17.93825884	-7.786396	0.019677
Pon2	ENSMUSG00000032667	1.384972382	0.423266715	0.163389937	4.014262991	9.219476398	6.270620116	-3.306337	0.019686
Oxsr1	ENSMUSG00000036737	3.969038398	0.486106321	0.316424958	6.5426312	9.14124807	12.38922261	-2.556653	0.019848
Ift22	ENSMUSG00000007987	19.17156227	5.726404922	18.04464919	28.48845419	33.26832645	32.24215728	-1.130234	0.020038
Ryr3	ENSMUSG00000057378	2.577302167	1.633098142	1.403145377	9.202196555	4.868055637	6.370993623	-1.864499	0.020065
Tmem109	ENSMUSG00000034659	13.33697568	0	0.092859922	24.00422536	41.12997954	26.26622386	-2.766759	0.020248
Sgsm1	ENSMUSG00000042216	6.577918126	1.291742148	5.314321354	12.42149596	12.00321308	9.428545959	-1.360508	0.020334
B3galt6	ENSMUSG00000050796	5.541764389	4.254130057	0.044306782	10.70486281	8.935482998	9.631303519	-1.572744	0.020353
Nmral1	ENSMUSG00000063445	1.189743191	0	0	3.046878142	7.551627781	6.016081619	-3.803728	0.020389
Krt1	ENSMUSG00000046834	6.89906939	1.90624083	4.113957461	14.35940357	15.35560482	9.349860731	-1.596347	0.020784
Spock1	ENSMUSG00000056222	0.653667709	0.272036487	2.453806513	37.53620377	19.87320084	17.16148288	-4.463726	0.020935
P4htm	ENSMUSG00000006675	43.67824911	13.51961512	39.49312914	87.99360382	88.05645943	62.11745207	-1.300523	0.021066
Spats1	ENSMUSG00000023935	1.23654443	0.007438303	0.530185026	2.770980691	5.762803737	3.749313908	-2.79146	0.021068
Ergic3	ENSMUSG00000005881	92.90276636	19.39704664	75.14238033	131.0606122	173.6406087	169.4450358	-1.338886	0.021177
Rbmxl1	ENSMUSG00000037070	4.373790164	7.735519351	1.664575737	20.33603764	29.50150765	14.87460771	-2.232101	0.021211
Basp1	ENSMUSG00000045763	96.76290006	51.31290313	37.53280649	244.7795472	181.082267	142.6621196	-1.614957	0.021317
Tmem59l	ENSMUSG00000035964	355.9503285	55.10370144	285.4539692	590.3641303	621.2161507	529.3425191	-1.32164	0.021323
Adprh	ENSMUSG00000002844	44.41453576	20.52018694	28.81147835	106.0730793	66.97718397	74.01510158	-1.398061	0.021375
Selenoh	ENSMUSG00000076437	13.05044871	25.22998781	28.15842898	96.69857764	69.16218557	52.1881911	-1.714553	0.021387
Rab15	ENSMUSG00000021062	8.470005798	9.871656859	4.158192446	15.69928726	18.03632553	13.15623712	-1.059421	0.021625
Gm32743	ENSMUSG00000111278	0	0	0.890611071	4.989647665	3.75769545	2.048105408	-3.599484	0.021656
Arg2	ENSMUSG00000021125	2.466587147	0	0	9.588928778	5.01583768	5.787336972	-3.047422	0.021692
Mapk1	ENSMUSG00000063358	48.28016502	26.32064611	24.67781817	67.73920406	78.48819814	58.21196531	-1.042118	0.021756
Atp1b3	ENSMUSG00000032412	41.50722053	5.533723943	23.45800222	70.31856778	99.14861961	64.44521338	-1.730295	0.021792
Drc1	ENSMUSG00000073102	0.025006027	0	0.486367254	2.739601106	4.010652836	6.723393568	-4.71962	0.021871
Phc2	ENSMUSG00000028796	2.117968714	3.46682211	4.238774131	10.58158479	6.079046256	9.276221076	-1.400685	0.021893
Eif1-ps1	ENSMUSG00000074553	1.32149275	0.109455758	0.427493314	3.861987293	2.220966266	2.542475679	-2.214503	0.021937
Arid5b	ENSMUSG00000019947	2.904394106	0.032314473	4.561026456	10.22027646	6.742376715	8.673734126	-1.773666	0.022005
Unk	ENSMUSG00000020770	4.111941944	0	1.168189538	6.818593011	6.020329918	6.078052212	-1.841036	0.022036
Eml1	ENSMUSG00000058070	0.004511241	0.004857504	0.071143488	5.743556855	2.083275915	3.791147167	-7.172936	0.022039
Dalrd3	ENSMUSG00000019039	32.83591456	2.409986023	14.09006137	75.85673245	98.91341624	51.67907644	-2.198476	0.022157
Gnpda1	ENSMUSG00000052102	3.268185057	0	0.016361957	7.624473303	14.7909862	8.349299047	-3.227512	0.022165
Plxna1	ENSMUSG00000030084	9.562751866	0.726078291	5.188561995	14.63920962	13.64370927	15.69334267	-1.506563	0.022207
Ccnl1	ENSMUSG00000027829	4.883324308	2.607992883	4.62832619	7.917440124	14.61015828	12.38868652	-1.526553	0.022234
Pigu	ENSMUSG00000038383	5.316360167	0.995383109	0.143704862	15.28642712	8.016054526	16.12179303	-2.610495	0.022254
Rmst	ENSMUSG00000112117	25.84044943	0.257898587	5.630178517	42.11598723	35.13662581	48.27484166	-1.984151	0.022578
Eml4	ENSMUSG00000032624	1.866730147	0	1.682219468	3.08754902	4.171208588	3.543380729	-1.605853	0.022786
Mfap2	ENSMUSG00000060572	3.795000142	0	11.91138989	24.37650958	29.60065692	16.83576587	-2.172661	0.022807
Spint2	ENSMUSG00000074227	3.71092514	2.862042252	19.84803792	58.39878808	46.72511338	29.56632467	-2.349888	0.022812
Sybu	ENSMUSG00000022340	11.51142322	3.681448223	5.609392764	15.70048158	14.8102866	15.98075696	-1.160227	0.022851
Gm26618	ENSMUSG00000097342	7.019599317	5.814148938	0	15.44541921	14.00951558	10.88865087	-1.652397	0.022855
Rmdn2	ENSMUSG00000036368	0.015887087	0.36779001	0.008351456	5.755049928	4.007521417	2.096052351	-4.918834	0.022863
Ramp2	ENSMUSG00000001240	1.046711686	0	0	11.07964792	7.226045954	4.282773709	-4.43165	0.022906
Ngb	ENSMUSG00000021032	4.302712901	3.039731886	0	52.39633936	150.8723137	113.7274604	-5.432061	0.022933
Ift46	ENSMUSG00000002031	5.66238603	1.18787476	5.315515567	10.84651103	8.468818531	10.06271137	-1.27191	0.022972
L3mbtl4	ENSMUSG00000041565	0.005517366	0	0	7.706986805	2.602694843	5.588846621	-11.49263	0.023206
Carnmt1	ENSMUSG00000024726	3.063555627	1.014406914	0.851073646	5.949897937	3.887961541	6.449277553	-1.724356	0.023261
Fkbp1a	ENSMUSG00000032966	92.62651863	66.13488897	95.81940661	210.6201301	170.5543164	133.1942166	-1.014679	0.023369
Kat8	ENSMUSG00000030801	12.74059237	0.049976341	5.77027252	23.58983221	19.00228315	31.60461726	-1.999094	0.023431
Tfg	ENSMUSG00000022757	53.34801834	12.35532016	15.60294642	79.15240059	81.16410751	68.21588706	-1.49096	0.023436
Tmem50a	ENSMUSG00000028822	93.37294777	109.6126163	55.98479372	315.8251315	183.7281227	216.7900945	-1.467864	0.023445
Gm45867	ENSMUSG00000109243	0.028956051	0	0	3.031647039	1.236532794	3.747874701	-8.112884	0.023499
Rpl18	ENSMUSG00000059070	39.68551422	11.56960632	20.9969215	64.47456597	49.44849825	58.08579052	-1.251373	0.023572
Lipo3	ENSMUSG00000024766	1.775430413	0	1.641200366	3.344469049	3.160452296	4.532736	-1.691788	0.023698
Ctdsp2	ENSMUSG00000078429	3.680055146	0.006538813	0.01276908	5.290291099	8.895524734	6.690568014	-2.496523	0.02375

A830052D11Rik	ENSMUSG00000097413	0.879622019	0	0	1.783962587	2.898638929	1.575031864	-2.830662	0.023762
Gm16536	ENSMUSG00000089810	3.356669633	4.296259064	1.198541671	10.10954743	6.184880885	8.078640469	-1.461299	0.023815
Cdk5r2	ENSMUSG00000090071	3.140029768	1.729236741	1.902643073	15.75637678	11.72375012	6.909375225	-2.344334	0.023867
Cd55	ENSMUSG00000026399	0.877867158	0	0.145345965	3.939179291	1.771098176	2.619226697	-3.025124	0.023883
Lrrc3b	ENSMUSG00000045201	0.711974414	0	0.104446787	34.23203686	54.37859408	19.50528109	-7.04905	0.024062
Zmym4	ENSMUSG00000042446	2.629571613	0.949719334	1.473299848	5.141856748	5.513530335	3.31421498	-1.467196	0.024189
Enoph1	ENSMUSG00000029326	10.9634834	8.018757654	19.00462506	27.21686745	25.54686586	37.26920756	-1.244952	0.024275
Mcfd2	ENSMUSG00000024150	38.2801761	64.49797686	39.29778035	112.2006733	124.443246	78.04015329	-1.147242	0.024324
Fxyd7	ENSMUSG00000036578	30.30236123	0	31.55307796	729.842892	442.4626301	281.3067734	-4.554598	0.024326
Akr1b3	ENSMUSG00000001642	4.013146702	4.691688442	5.111028279	25.94783425	19.52219811	11.77866148	-2.050917	0.024369
Mtpap	ENSMUSG00000024234	2.316474744	0	0.603166995	3.959452066	3.085272596	3.702341762	-1.88008	0.024398
Arf1	ENSMUSG00000048076	241.3226684	60.43121061	146.7116536	359.4502528	328.9826216	323.9191908	-1.174642	0.024457
Hyi	ENSMUSG00000006395	10.61492261	0	1.772191048	28.33704258	14.64389935	26.92906449	-2.496659	0.024473
Zfp467	ENSMUSG00000068551	10.88753306	6.93545641	5.768899202	23.48790741	13.92866858	19.67894102	-1.275087	0.024653
Jund	ENSMUSG00000071076	41.81627237	7.583767304	16.7137833	82.21263166	88.67211147	53.07398916	-1.760209	0.02466
Plch2	ENSMUSG00000029055	0.525183595	1.353624468	0	7.690958766	7.158180846	3.040815957	-3.25126	0.024708
Rad51ap2	ENSMUSG00000086022	0.126786464	0.05688252	0	2.92184338	5.246377006	2.047994189	-5.797609	0.024867
Tuba1b	ENSMUSG00000023004	345.9936385	141.8809323	446.373129	578.5752736	688.4284715	766.0113105	-1.121744	0.025009
Atp6v0e2	ENSMUSG00000039347	213.8548667	75.97268567	238.9231586	498.7758682	415.3887852	336.981072	-1.24259	0.025022
Faim2	ENSMUSG00000023011	55.56724372	13.08354754	39.6288351	90.05655657	80.91803212	74.98881742	-1.183682	0.025078
Tmem130	ENSMUSG00000043388	124.1406207	41.96834658	99.57221987	204.0799583	241.3493362	164.7253615	-1.199479	0.02512
Ache	ENSMUSG00000023328	68.05624996	17.29060043	41.94344688	104.3406237	130.4403322	89.88171241	-1.350819	0.025156
Hdac4	ENSMUSG00000026313	3.065412674	0.127916424	2.464513961	4.687656127	5.618779198	5.136080636	-1.448584	0.025199
Cgref1	ENSMUSG00000029161	10.79516911	0.329629013	16.88875784	26.60632557	49.33169307	37.31650903	-2.015372	0.025232
Uap1	ENSMUSG00000026670	12.76254376	3.899310227	3.851276717	16.72938922	17.7494574	17.14221916	-1.331412	0.025286
Egr1	ENSMUSG00000038418	27.740194	17.19827266	22.61286139	59.32328312	102.6552996	59.47402882	-1.712942	0.025326
1110017D15Rik	ENSMUSG00000028441	0	0	0.024819281	6.507539919	2.977438942	2.922684041	-8.965554	0.025343
Gm5148	ENSMUSG00000058174	0.017198454	0.111111203	1.175305254	8.197884951	5.738872074	3.129376307	-3.710547	0.025349
Mast4	ENSMUSG00000034751	1.323146134	1.558480281	0.924128899	3.157684226	5.029922057	2.89374238	-1.541879	0.025413
Chsy3	ENSMUSG00000058152	1.062802851	0	0	1.709913873	3.973034562	3.772558337	-3.153281	0.0255
Fgf13	ENSMUSG00000031137	26.41746135	35.28457116	6.546423495	48.01600396	57.02174773	57.11745313	-1.248507	0.025544
Rpl10-ps3	ENSMUSG00000058443	1.572449306	2.116429704	2.663483735	3.897772422	6.203089375	7.374172624	-1.45993	0.025599
Galnt2	ENSMUSG00000089704	6.878333443	3.399467845	1.902604819	15.47681036	12.54934115	8.983869282	-1.603354	0.025639
Gm49628	ENSMUSG00000110869	1.93027669	0	0	6.010490552	5.862606484	11.95247645	-3.625631	0.025705
Slc3a2	ENSMUSG00000010095	30.28513302	0.020470615	8.234909465	68.42304267	54.16791343	40.91663963	-2.08491	0.025757
Nop16	ENSMUSG00000025869	21.87281246	30.69744587	12.53980388	60.28906085	39.00233442	49.5224653	-1.192556	0.025954
Jmjd8	ENSMUSG00000025736	2.918064678	0.248745054	0.294008178	12.80503824	7.679600644	6.021216415	-2.937126	0.02597
Chst8	ENSMUSG00000060402	4.444764755	0.027223696	7.623544851	12.38884237	17.07907658	11.55956458	-1.762116	0.026003
Anks1b	ENSMUSG00000058589	13.71804153	7.260945231	11.28747734	36.89433089	19.9413701	36.58440333	-1.533698	0.026048
Tomm40	ENSMUSG00000002984	29.60604162	2.961344583	20.4825943	43.7911734	43.2726709	49.40588542	-1.363157	0.026064
Tssc4	ENSMUSG00000045752	27.47283427	6.53886921	24.41056335	39.27704604	44.25681559	51.09135883	-1.204358	0.026153
Pipox	ENSMUSG00000017453	0.013581209	0	0	13.93129829	4.423948999	10.27541031	-11.04173	0.02617
Plekhm3	ENSMUSG00000051344	2.389750507	3.677857807	3.416697549	6.847170908	4.722171659	7.513584843	-1.008668	0.026193
Tmem134	ENSMUSG00000024845	18.5154204	7.525083391	10.17205074	27.56958537	21.7048976	25.47015814	-1.04548	0.026203
Vps72	ENSMUSG00000008958	8.536194529	0.516147517	8.732246986	31.91087412	26.19202179	15.80506007	-2.055102	0.026255
Tmem191c	ENSMUSG00000055692	25.89933645	5.584981974	3.725341321	38.43325525	66.88365054	44.96816131	-2.093658	0.026333
Cacnb1	ENSMUSG00000020882	2.777565566	0	0.817655296	12.44699505	10.8130506	5.02965574	-2.976125	0.026412
Gapdh	ENSMUSG00000057666	786.1094999	172.561651	831.2901249	1197.566362	1552.876184	1679.718884	-1.307431	0.026614
Itpa	ENSMUSG00000074797	33.23798431	14.38927815	19.99669121	77.75767281	44.2439561	60.32972282	-1.430956	0.026739
B230303A05Rik	ENSMUSG00000113701	0.017767789	0	0.205482088	1.538439459	3.661418169	4.766131911	-5.480282	0.02676
Adi1	ENSMUSG00000020629	39.75311925	10.68673561	15.52099931	49.07797337	54.42309473	59.34510305	-1.303828	0.027005
Efcab1	ENSMUSG00000068617	0.03785329	0.065213993	1.018806003	4.622028532	11.44316404	6.295940959	-4.317012	0.027086
Xrcc3	ENSMUSG00000021287	8.465685864	0	0.012824799	11.14407868	20.5823001	17.00916229	-2.523091	0.027122
Gal3st3	ENSMUSG00000047658	1.371752554	0	0.131108544	12.19328918	16.63660604	5.631567877	-4.5192	0.027144
Uck1	ENSMUSG00000002550	16.91302314	4.674250272	20.02505487	29.63850713	47.89488321	36.47634682	-1.454074	0.027218
Trim27	ENSMUSG00000021326	5.367293631	0	2.840693073	7.808399424	8.455824292	11.45639541	-1.755859	0.027355
Kctd13	ENSMUSG00000030685	37.96977174	0.595471014	0.709051034	62.6377135	77.3855375	49.32218803	-2.269363	0.02806
Vwa1	ENSMUSG00000042116	5.898023122	0	0.165486992	10.32398454	13.82050358	7.966377011	-2.404836	0.028135
Cdipt	ENSMUSG00000030682	49.12771659	0.445930868	81.87065969	128.7537	117.0505774	127.4386507	-1.505663	0.028139
Nell2	ENSMUSG00000022454	52.83968409	39.314774	36.46142655	67.66084037	85.69743389	110.4286474	-1.036304	0.028149
Ppp2cb	ENSMUSG00000009630	11.39499456	7.647001011	18.4623857	40.85634918	43.90528275	23.7312153	-1.532469	0.028195
Srp72	ENSMUSG00000036323	12.01702965	6.861422896	15.55822922	23.71287942	19.22998603	28.39038546	-1.050629	0.028209
Tppp3	ENSMUSG00000014846	26.79290246	9.851018257	46.52806697	216.5311065	200.445811	87.85161499	-2.601624	0.028332
Abhd16a	ENSMUSG00000007036	35.10826774	3.275514559	35.8048884	54.70237512	71.53304703	67.186499	-1.382481	0.028506
Dmwd	ENSMUSG00000030410	10.36815818	0.098921284	22.27029431	43.86638203	36.91183371	29.19818461	-1.748183	0.028507
Rabl2	ENSMUSG00000022621	32.75227744	17.30362419	11.46216449	48.92356278	81.04651054	52.29683504	-1.566971	0.028525
Rps2	ENSMUSG00000044533	101.9199201	113.1575163	88.96065789	189.3949193	180.8267042	288.6355935	-1.115714	0.028545
Smad2	ENSMUSG00000024563	2.070573645	1.410920999	0.004016422	3.638573134	8.460462543	6.855650597	-2.443112	0.028793
Crb3	ENSMUSG00000044279	1.522849456	0	0	4.415455346	2.385692664	5.740077764	-3.041833	0.028834
Metrnl	ENSMUSG00000039208	3.764889133	0	0	12.57674357	12.68723478	26.50362573	-3.78137	0.028847
Mamld1	ENSMUSG00000059401	2.237766594	5.614725562	2.66050624	8.265768198	6.750131263	7.07898777	-1.071538	0.028975
Cers1	ENSMUSG00000087408	14.21799637	0	1.545972747	30.71338203	37.64296183	18.69332993	-2.465208	0.029115
Wdr91	ENSMUSG00000058486	0.526403207	3.990947064	1.69062946	4.816575965	6.47383976	6.465769244	-1.516126	0.029225
B3gntl1	ENSMUSG00000046605	2.793876197	0	0	3.732194047	7.518961974	5.843068751	-2.613169	0.029513
Acbd7	ENSMUSG00000026644	0.628194479	0.946976786	0	7.659389483	8.110270072	2.828147104	-3.561552	0.029604
Tmie	ENSMUSG00000049555	4.607910054	4.786478382	0.273573614	9.857150562	13.86847713	8.189499877	-1.722957	0.029633
Pmf1	ENSMUSG00000028066	0.049538152	0.044450406	3.289846715	13.0502804	5.305064634	13.44326976	-3.232233	0.02966
Trim66	ENSMUSG00000031026	0.569860805	0.898996629	0.257762635	1.536811481	3.662126598	3.086753806	-2.262672	0.029735
Samd5	ENSMUSG00000060487	0.785088819	0.052182029	1.951418665	8.687739233	7.585039371	3.399945987	-2.818538	0.029794
Mto1	ENSMUSG00000032342	3.347977728	5.234712252	2.153453018	8.801430146	10.88153654	16.99431144	-1.772411	0.029824
Abhd12	ENSMUSG00000032046	84.91560461	49.65755348	39.2660105	110.7428173	146.6522199	195.6666047	-1.381954	0.029999
Dnajb9	ENSMUSG00000014905	16.75305216	4.492023102	11.74684208	54.42834519	26.21768338	40.25569868	-1.87365	0.030015
Cacna1g	ENSMUSG00000020866	0.218408664	0.851610503	0.034791554	2.131446355	4.719306833	2.549247588	-3.088862	0.030099
2610035D17Rik	ENSMUSG00000087259	3.321345069	0	5.986115716	21.96687091	10.38231048	25.77178494	-2.642599	0.030132
Arsg	ENSMUSG00000020604	2.782583104	0	0.009029237	3.664976688	8.094074448	7.399932094	-2.77885	0.030201
Dusp28	ENSMUSG00000047067	29.6395924	9.208777848	26.46456899	80.72771463	49.870327	105.6114213	-1.854626	0.030279

Hps4	ENSMUSG00000042328	6.746503203	4.601965891	3.466074212	8.53570988	9.071195804	12.38973901	-1.017787	0.03045
Ifitm10	ENSMUSG00000045777	5.484692673	0	14.92767555	33.35183022	19.87461042	24.90421025	-1.936445	0.030582
Gm37067	ENSMUSG00000104416	2.555069143	0	0.021663513	4.423929504	3.417395882	3.607566842	-2.151593	0.030595
Rbfa	ENSMUSG00000024570	12.74141726	0	9.742332402	20.30749439	18.81129857	23.14823405	-1.469586	0.030683
Pcbp3	ENSMUSG00000001120	19.42052631	4.396516501	0.132270775	36.63129504	41.26544721	23.48560981	-2.08175	0.030996
Gm44643	ENSMUSG00000108867	2.986253875	4.340878965	1.412821005	22.55868569	14.33412946	9.64745037	-2.412782	0.031133
Cib2	ENSMUSG00000037493	26.19505222	54.39485261	7.851050452	70.40438121	74.99829561	76.23168879	-1.325395	0.031311
Rars	ENSMUSG00000018848	16.76240053	22.93891591	23.0751038	56.94486407	31.7251408	51.30791533	-1.156905	0.031409
Gm10132	ENSMUSG00000063556	2.795465433	1.389246164	1.35646917	9.879502377	7.403220886	4.392289715	-1.967768	0.031443
Far2	ENSMUSG00000030303	1.697233218	0	1.263539996	4.275758926	4.314074362	2.363428096	-1.887315	0.031508
Lrrc8c	ENSMUSG00000054720	0	1.302486344	1.308618808	2.183971521	3.725791332	2.784299862	-1.73537	0.031559
Bmerb1	ENSMUSG00000044117	82.38627646	82.19464781	206.7791408	254.1734021	248.7945885	293.0078485	-1.099906	0.031867
Mgme1	ENSMUSG00000027424	2.324544733	0	0.042136438	3.372840865	8.149513314	6.202410427	-2.904829	0.031989
Cnrip1	ENSMUSG00000044629	24.52020542	10.99041907	9.946988764	29.83101305	31.74156384	29.77326035	-1.006817	0.032022
Sil1	ENSMUSG00000024357	11.46173371	0	7.123437051	31.26879754	26.09118257	15.81092814	-1.977118	0.032077
Hnrnph3	ENSMUSG00000020069	14.43599287	0.768031361	8.551222976	27.851355	19.88719873	36.79852295	-1.831338	0.032202
Dcaf13	ENSMUSG00000022300	1.137296052	1.214579741	3.766285208	6.220664037	4.237788288	6.603625515	-1.479623	0.032284
Mocs3	ENSMUSG00000074576	0	0	0.053626252	1.457138972	2.566549968	4.443855742	-7.30286	0.032373
Pcsk1n	ENSMUSG00000039278	290.820185	32.10153221	195.2536238	866.0444765	757.2856932	409.6950874	-1.972116	0.032386
Sppl3	ENSMUSG00000029550	3.406027378	0.293396753	1.61805062	5.880443879	5.176575254	9.313997096	-1.937705	0.032655
Omg	ENSMUSG00000049612	24.96345613	126.9239739	44.62345214	169.1177474	167.5742972	161.1756126	-1.341153	0.032688
2610028E06Rik	ENSMUSG00000085562	1.231238547	0	0	8.422381445	2.873103174	5.828343538	-3.797823	0.032858
Jagn1	ENSMUSG00000051256	12.96794457	3.556991352	5.540373492	25.1823072	16.44244076	18.073783	-1.435915	0.033016
Tmem14c	ENSMUSG00000021361	65.51298641	49.55687302	87.55310966	143.5878661	107.0875091	180.3794698	-1.089074	0.033147
Fam13c	ENSMUSG00000043259	1.928373831	0	0.004268207	2.338655047	4.479293576	4.934796677	-2.604351	0.033209
Rnf31	ENSMUSG00000047098	0.936047023	0	4.145581747	4.946643063	6.611770868	7.462480935	-1.904222	0.033217
Tubb3	ENSMUSG00000062380	284.6346496	61.70246107	310.9756404	509.9949329	562.8330384	427.7504365	-1.190867	0.033424
Mvb12b	ENSMUSG00000038740	2.932024607	1.070264692	1.549363711	3.248334267	4.478266838	4.780121875	-1.171715	0.033485
Fbxw9	ENSMUSG00000008167	7.268375391	0	0.083401001	9.426850036	16.29780891	20.91962216	-2.665535	0.033496
Polr3a	ENSMUSG00000025280	6.354551485	1.035021765	5.773140236	12.74435464	8.751628396	11.17873508	-1.311718	0.033584
Gpr101	ENSMUSG00000036357	0	0	0.097786132	8.151959078	2.309230175	5.831245514	-7.380357	0.033611
Ampd3	ENSMUSG00000005686	0.941923309	0	0.313383672	6.420710893	2.196494983	4.592451182	-3.395481	0.033615
Tbrg1	ENSMUSG00000011114	12.67526085	6.953709461	2.992507993	21.11392241	14.83395951	18.61494979	-1.270225	0.033631
Doc2a	ENSMUSG00000052301	0.87364528	0	0	2.33682129	4.69469323	2.140196705	-3.392071	0.033654
Tmem39b	ENSMUSG00000053730	0.542951291	0.268611901	0.323986075	15.59716714	4.517713533	12.31365682	-4.835802	0.033737
Rbp1	ENSMUSG00000046402	6.046300801	0	5.864734354	11.99174893	9.08234508	11.65828639	-1.45842	0.033806
Srprb	ENSMUSG00000032553	30.67382645	0	17.26800758	42.22121835	44.71462524	46.17822604	-1.473306	0.033822
Sf3b4	ENSMUSG00000068856	15.59357684	1.486917495	0.212065948	23.18164457	39.16995546	23.51731204	-2.311984	0.033967
Phlda3	ENSMUSG00000041801	52.76059968	11.30644986	51.46578026	75.85480658	84.60043726	90.17145223	-1.117237	0.034043
Mir99ahg	ENSMUSG00000090386	2.314863496	1.28541451	1.087413515	2.596451754	4.274075122	3.742521356	-1.17889	0.034066
Zbtb20	ENSMUSG00000022708	3.214830732	5.47741833	8.663834361	12.02032914	15.19587576	10.33276843	-1.113332	0.034107
Mir8109	ENSMUSG00000098388	0.286714403	0	0	5.066411475	1.708435589	6.095422462	-5.488284	0.034233
Mrm2	ENSMUSG00000029557	4.137986254	0	1.099839868	30.11091577	18.67735664	10.78569984	-3.507642	0.034421
Fdft1	ENSMUSG00000021273	12.59181952	16.78773706	18.25103563	55.05734342	32.05993943	33.11426323	-1.335855	0.034556
Npdc1	ENSMUSG00000015094	75.53339012	2.700244977	69.98561341	118.091164	134.8955548	121.5672444	-1.337441	0.034561
Adipor2	ENSMUSG00000030168	2.311553956	3.660973836	0.176960353	5.512296359	5.906232418	4.785161201	-1.397784	0.034722
Evl	ENSMUSG00000021262	10.82814978	3.931472139	2.57913696	17.21856129	13.54109535	13.3700494	-1.34775	0.034923
Txlng	ENSMUSG00000038344	1.895029743	2.227288971	0.402209928	3.325046683	3.118954085	3.426829705	-1.125404	0.035025
Cep57l1	ENSMUSG00000019813	1.611059552	0.20372128	0.090415822	3.221686983	2.99494511	6.072871108	-2.689415	0.035056
Emc10	ENSMUSG00000008140	130.4333313	27.50202571	106.8081128	202.4197254	175.4121745	188.5271429	-1.097122	0.035086
Snx1	ENSMUSG00000032382	14.14805737	4.1539709	4.226656645	22.33646424	24.44649662	41.94118039	-1.977564	0.035105
Mettl23	ENSMUSG00000090266	9.96292686	0	0.679994927	12.35212533	15.24289417	14.23249038	-1.974558	0.03526
Mpp3	ENSMUSG00000052373	5.751182374	2.032133335	3.945829339	8.365733126	6.801339422	10.19157539	-1.11238	0.035332
2610042L04Rik	ENSMUSG00000079388	0.035573261	0.191518559	0	2.938706699	2.684943312	6.672987599	-5.758844	0.035609
Ltbp4	ENSMUSG00000040488	1.147079426	0	0.381985748	10.28153071	3.463923868	11.63822865	-4.05318	0.035652
Cfap20	ENSMUSG00000031796	8.697282704	22.01632126	2.694043465	39.46029954	30.19336525	27.22893775	-1.536059	0.035737
Mzt2	ENSMUSG00000022671	12.12151398	14.376416	23.00919897	83.25499673	42.06941066	48.00792312	-1.807833	0.035898
Shmt2	ENSMUSG00000025403	13.21754322	0.092944098	4.072461639	21.75460704	15.70892512	19.76776885	-1.719132	0.03593
Itpa-ps1	ENSMUSG00000081476	1.348566239	0	0.76798422	5.113511281	8.816901256	14.82684938	-3.76414	0.035935
Olfm1	ENSMUSG00000026833	127.0445805	145.7015414	159.9923508	396.6788431	286.1595953	225.4341555	-1.06963	0.035993
Glce	ENSMUSG00000032252	3.060764437	1.370120631	0.005165231	10.93429986	7.084453027	4.866003541	-2.367039	0.036061
Ccdc142os	ENSMUSG00000087578	0	0	1.335156487	6.294419797	6.95205507	2.285410964	-3.540131	0.036095
Rab3gap2	ENSMUSG00000039318	3.540922882	0.568561856	2.730019804	5.181250135	5.259371504	7.718179295	-1.408705	0.036195
Coa8	ENSMUSG00000037787	7.810040378	5.423456515	4.206540599	9.682699541	10.71771071	14.94812363	-1.019247	0.036309
Cdk9	ENSMUSG00000009555	8.068109734	3.125577903	7.006217688	15.20813443	9.717630538	14.76604369	-1.12491	0.036399
Usp10	ENSMUSG00000031826	5.639476453	2.482592633	3.213578913	10.11101284	6.87967477	7.285114432	-1.098652	0.036422
Ftx	ENSMUSG00000086370	3.313468876	1.398094866	1.700338425	11.80602954	4.873938219	9.356210844	-2.021693	0.036466
Tmem245	ENSMUSG00000055296	1.091360174	0.21346755	0.103183729	1.567771237	1.715433277	2.847453745	-2.122383	0.036908
Ruvbl1	ENSMUSG00000030079	17.87684688	20.27611727	55.25165367	62.44532229	75.73389707	72.76782288	-1.175315	0.037027
Ago2	ENSMUSG00000036698	1.649665297	1.083792211	1.862571095	3.958560499	4.910154199	2.511489767	-1.308067	0.037056
Tubb2b	ENSMUSG00000045136	16.5826912	21.91787148	8.378887595	25.21986309	32.54480428	36.39153833	-1.006101	0.037062
Hsd17b4	ENSMUSG00000024507	4.355801603	2.865014123	4.942106536	11.24939812	10.40343922	6.149669067	-1.192725	0.037071
Ttc9b	ENSMUSG00000007944	12.80028909	1.003344452	4.399931188	36.71592842	24.82351916	17.54085194	-2.119097	0.03708
Gm45183	ENSMUSG00000109353	6.655336542	0.072385572	0.023559251	10.1566828	8.078264073	13.53524377	-2.23444	0.037294
Sorcs3	ENSMUSG00000063434	2.550029057	0.187441067	3.136050183	4.43954076	7.77961185	9.360981808	-1.877407	0.037449
Gm31728	ENSMUSG00000102923	2.853302649	0	0	5.58702893	3.369741921	4.436181702	-2.230769	0.037502
Ctsl	ENSMUSG00000021477	26.95433522	17.91825682	5.603561809	52.48514185	36.34355108	36.66254631	-1.313913	0.037575
Caly	ENSMUSG00000025468	114.6641473	41.59637933	154.4250604	557.1817144	473.3405447	230.3365425	-2.02088	0.037577
Prkcz	ENSMUSG00000029053	18.66829338	21.71912008	10.57822666	41.88233486	25.71090103	36.57917466	-1.031376	0.037841
Bub3	ENSMUSG00000066979	18.32414424	13.51292737	2.411285619	26.63065978	25.92501017	38.21443284	-1.406182	0.037902
Plcxd2	ENSMUSG00000087141	0.784609626	0.202355184	0.90887234	3.04272069	2.043662236	1.468867252	-1.789816	0.037926
Derl1	ENSMUSG00000022365	14.93826841	8.704495499	8.636893545	30.33315964	19.8105119	19.67706575	-1.11303	0.038003
Cirbp	ENSMUSG00000045193	48.90132158	9.113326797	11.29557507	109.7216623	95.29941079	55.00007584	-1.907489	0.038093
Kcnh5	ENSMUSG00000034402	0	0.026703105	0	3.330497598	2.889773751	7.62930664	-9.018619	0.03811
Slc26a11	ENSMUSG00000039908	0	0	0.009769584	5.755289058	12.93820514	4.799363024	-11.23164	0.038118
Gstm4	ENSMUSG00000027890	5.227372971	0	0	8.525194494	15.86683281	23.54359289	-3.19694	0.038156

Rps6ka2	ENSMUSG00000023809	1.926400112	0	0.802353281	4.161510171	2.497418471	2.917808078	-1.811292	0.03819
Cetn4	ENSMUSG00000045031	0.931821811	0.125418057	0.629789258	6.784807064	2.354819143	4.421902456	-3.006963	0.038331
Wdr48	ENSMUSG00000032512	3.930624187	1.638709769	6.858473448	9.472237128	8.273578265	12.39211247	-1.278008	0.038502
Tubb4b	ENSMUSG00000036752	188.0673469	25.56798449	187.809913	258.8401233	364.8000912	378.1001062	-1.319233	0.038674
Ciao2b	ENSMUSG00000031879	20.20217398	17.2881098	31.92493055	73.49027605	40.83812042	88.15136517	-1.544454	0.039119
2410002F23Rik	ENSMUSG00000045411	6.846299444	0.911120359	9.85999891	12.99835159	19.44883989	25.38054405	-1.714759	0.039154
Mras	ENSMUSG00000032470	11.00326558	0.802050181	1.030430418	15.82375303	19.12955362	12.68167456	-1.891854	0.03925
Med22	ENSMUSG00000015776	11.32418759	0.299837302	3.171600578	18.94436953	37.35205579	20.07001035	-2.367768	0.039423
Plcxd1	ENSMUSG00000064247	0.572149839	0.388595227	0	2.854193681	2.980908541	6.736793337	-3.709905	0.039449
Alg1	ENSMUSG00000039427	9.255679734	0	12.24261474	15.84698703	21.12899115	23.08704426	-1.482255	0.039607
Hspa1a	ENSMUSG00000091971	0.384411829	0.085218336	0	2.817006744	4.41273884	8.483488111	-5.064311	0.039752
Mboat7	ENSMUSG00000035596	10.71656541	0.79699648	10.64516794	28.63977581	18.77261149	17.81364136	-1.557573	0.039756
Ube2s	ENSMUSG00000060860	12.85446466	1.573812422	14.99635903	21.45779489	28.68118078	22.14639241	-1.296679	0.039816
Fam118a	ENSMUSG00000022434	4.728446068	0	0.234694605	6.927007414	6.576908573	5.70172858	-1.952205	0.039836
Rpn2	ENSMUSG00000027642	39.51261028	7.411612607	14.73621564	60.86933135	43.21107956	57.84103474	-1.392877	0.03992
Mir135a-2	ENSMUSG00000065524	0	0	5.642911748	25.19273106	9.661203256	15.10230553	-3.146153	0.039937
Mchr1	ENSMUSG00000050164	0.091947698	0	16.88491181	28.31161188	18.52158026	30.26057109	-2.183045	0.040048
Plxnb2	ENSMUSG00000036606	1.264477369	2.652884321	0	5.639762562	5.243148989	3.043066746	-1.829824	0.04006
Akirin2	ENSMUSG00000028291	10.13621715	14.56638001	7.977085208	16.59479242	29.32298536	24.34669361	-1.104401	0.040065
Mad2l2	ENSMUSG00000029003	18.41041079	3.154680268	0.941561361	24.89092018	34.69942146	22.54338132	-1.867623	0.040106
Rbbp7	ENSMUSG00000031353	55.16743884	41.18644473	43.9852265	64.23884065	111.1895573	112.426544	-1.036425	0.040193
Pde1b	ENSMUSG00000022489	28.93897314	8.461116743	46.94801136	75.73308679	55.13314166	66.65800163	-1.227602	0.040317
Gpr83	ENSMUSG00000031932	2.218814958	5.373751405	2.941900752	144.0972444	133.6568345	37.37289544	-4.902744	0.040427
A330040F15Rik	ENSMUSG00000086213	0	0	0.045546533	4.057273038	2.122490362	1.264123723	-7.352571	0.040538
Rhoc	ENSMUSG00000002233	14.49219039	0	0	39.56685748	39.51206054	16.51587801	-2.721656	0.040692
Plpp1	ENSMUSG00000021759	21.64169468	26.21309049	4.93754778	40.98582129	34.51381577	37.46810087	-1.09751	0.040742
H13	ENSMUSG00000019188	8.329756972	0.004065781	4.239805934	15.71668247	19.49220393	10.02022748	-1.846851	0.0408
Tmem185a	ENSMUSG00000073139	4.258273648	12.20303217	13.66940918	24.30758583	16.58433425	25.94175045	-1.14934	0.040804
Dpp6	ENSMUSG00000061576	8.723971334	9.598366672	11.51422271	31.79652267	28.24730198	15.22489293	-1.334969	0.041277
Arhgap39	ENSMUSG00000033697	3.729311019	0.911957982	2.427172065	4.232348242	5.598390601	5.42109515	-1.109519	0.041366
Cd9	ENSMUSG00000030342	12.65344725	8.040500061	4.814005558	17.54530021	18.75977621	30.14960702	-1.381424	0.041633
Myg1	ENSMUSG00000001285	6.511859967	24.60486352	23.54961177	35.27643782	40.3479472	33.88166314	-1.002286	0.041709
Dcakd	ENSMUSG00000020935	17.56024765	9.609496161	26.27177222	106.4133724	54.72909243	53.14430677	-2.00351	0.041787
D630045J12Rik	ENSMUSG00000063455	2.558942038	0.789546296	1.224553804	6.207559449	3.125865532	7.326583168	-1.865163	0.041823
Cckbr	ENSMUSG00000030898	5.825351447	5.616204795	1.565010901	16.36227857	21.23187877	9.043911129	-1.842268	0.042083
Magee1	ENSMUSG00000031227	53.90917283	18.20264679	33.60910289	125.7424101	81.70027737	71.44643135	-1.399431	0.042199
Washc3	ENSMUSG00000020056	5.657489567	1.958910597	3.117845584	7.9976924	6.867074929	11.51428718	-1.297172	0.042477
Zfp623	ENSMUSG00000050846	0.323851185	0	1.801714079	7.487120868	12.99881704	4.370583192	-3.547706	0.042658
Pcdh1	ENSMUSG00000051375	3.004879065	0.110861749	0.024054699	5.970152852	12.25861283	5.674833462	-2.928485	0.042693
Ppp5c	ENSMUSG00000003099	36.33748498	3.190481784	32.97396613	52.29268493	54.89030307	59.83614358	-1.203922	0.042934
Zfp825	ENSMUSG00000069208	2.788260625	0	0	17.2063756	8.00664063	27.38157844	-4.237477	0.042943
Gtf2f2	ENSMUSG00000067995	1.486341539	0	1.298215893	27.15347893	7.675732084	30.42651876	-4.550585	0.042981
Pcdhgb7	ENSMUSG00000104063	0	0	0.014966348	2.892788729	0.664452034	2.634723365	-8.692531	0.042994
Tom1	ENSMUSG00000042870	13.83973523	0	2.258067926	18.82205315	16.41062109	25.70098014	-1.920375	0.043067
Mpped2	ENSMUSG00000016386	10.80314758	0	5.653209032	22.79352636	13.11180668	17.31927009	-1.693448	0.043141
Ly6h	ENSMUSG00000022577	17.45132289	6.972852289	18.70624577	195.4304402	166.4384869	55.61688104	-3.274949	0.043332
Magef1	ENSMUSG00000116632	3.273864672	0	1.115008335	7.272128554	16.46262624	24.13255008	-3.447118	0.043353
Gfra4	ENSMUSG00000027316	34.11888157	10.71099167	18.97803749	81.33134913	45.36310775	51.34861389	-1.480419	0.043512
Slc12a2	ENSMUSG00000024597	0.387064445	0.06284685	0.875245584	1.829336429	4.295495195	2.208950367	-2.652808	0.043578
Clvs1	ENSMUSG00000041216	0.013301184	0	0.213559414	0	4.301226649	2.073899903	7.57399674	-10.0344	0.043755
Unc5b	ENSMUSG00000020099	0	0	0	2.505396336	4.040540258	1.09938306	-5.161867	0.043828
Celf5	ENSMUSG00000034818	1.900033146	0.290729112	1.429862867	15.35600697	11.40111465	4.589644202	-3.114006	0.043853
Sarm1	ENSMUSG00000050132	2.908197828	0	0.375926791	6.360511035	5.072041454	3.174819959	-2.153116	0.043967
Maf1	ENSMUSG00000022553	49.92895972	0	20.9240431	55.23428257	83.76965424	79.88401663	-1.627292	0.044087
Magel2	ENSMUSG00000056972	0.333210304	0	0	8.422430444	9.431073551	2.192205871	-5.910717	0.04415
Samd8	ENSMUSG00000021770	0.821183981	0.316804139	0.272394778	2.386134558	3.017885828	6.101242691	-3.028135	0.0442
Prkcsh	ENSMUSG00000003402	15.6075249	4.021827025	6.707145104	24.70222243	20.7679828	16.93734851	-1.244657	0.044466
Exoc6b	ENSMUSG00000033769	2.194550277	0.651613644	0.86230314	5.227329382	2.459016043	4.18039251	-1.678029	0.044501
Kcnq5	ENSMUSG00000028033	1.108151728	0	3.137397285	3.843099442	7.642966237	5.232262181	-1.977408	0.044765
Lipa	ENSMUSG00000024781	5.115949129	5.679107182	6.569872362	16.63778144	19.08458623	36.8709598	-2.06366	0.044966
Cck	ENSMUSG00000032532	254.8693312	107.7245961	106.8856129	589.1363027	279.1014274	511.1574293	-1.554902	0.045001
Vps26b	ENSMUSG00000031988	3.934155706	0.85714227	3.001843129	4.51058669	6.513934204	6.680543077	-1.183885	0.045005
Stx1b	ENSMUSG00000030806	18.54018807	2.58755054	17.62675473	24.75019025	33.89725481	30.35801734	-1.19953	0.045041
Emc8	ENSMUSG00000031819	6.709117039	2.203373107	1.254976401	8.639660141	9.002057867	7.543822198	-1.308635	0.045041
Marchf8	ENSMUSG00000025702	4.739001604	0	2.167196748	6.439007206	8.935504719	13.97345583	-2.087297	0.045395
Pomgnt2	ENSMUSG00000066235	21.20432271	0.447927802	9.9256349	63.03415192	48.19871676	26.06935276	-2.120368	0.045711
Adcyap1r1	ENSMUSG00000029778	4.192562913	1.165527232	0.945685672	6.26355362	4.446306499	5.71334843	-1.381448	0.045732
Lzic	ENSMUSG00000028990	3.847135708	0.832356491	0.018900428	8.799428162	4.552623776	6.0026134	-2.042442	0.045732
Syvn1	ENSMUSG00000024807	7.891048339	1.895596228	12.34640986	17.35978417	21.83714002	14.60811608	-1.281539	0.045827
Tmeff2	ENSMUSG00000026109	23.70748082	8.659848463	41.48364655	67.58923014	44.14778926	67.33902448	-1.277883	0.045928
Tiprl	ENSMUSG00000040843	11.21659436	1.042759591	1.836549708	17.15058507	11.9854264	21.36250966	-1.840965	0.046023
Rac1	ENSMUSG00000001847	53.28793805	45.85748327	13.97078768	66.58116593	86.06491274	110.4102297	-1.217566	0.046098
Kifc3	ENSMUSG00000031788	1.608263239	0	0.006656889	2.981752416	2.54040309	1.694503893	-2.159868	0.046331
Sfmbt2	ENSMUSG00000061186	3.18624259	0	0.158413249	4.665921861	3.919781611	3.888082604	-1.89897	0.046353
Arhgap26	ENSMUSG00000036452	3.750288091	0.05572416	3.221038795	6.233749571	6.338765183	4.983362157	-1.320963	0.046554
Fam122b	ENSMUSG00000036022	1.803198841	0	0	5.216484811	2.478374694	7.488164648	-3.073829	0.046667
Nudt9	ENSMUSG00000029310	26.22501853	17.53168257	12.72427059	78.18519231	36.15569665	51.9825734	-1.558155	0.046703
Nkain2	ENSMUSG00000069670	0.192299533	2.331337937	0.284541728	10.13120944	26.28379186	11.21361327	-4.084122	0.046844
Sv2b	ENSMUSG00000053025	0.780502221	2.891873116	0.037873021	6.885356567	3.068701749	7.177844774	-2.207098	0.046903
Atxn7l1	ENSMUSG00000020564	4.196119076	1.218510866	6.485782734	8.432330768	8.927846781	13.55502097	-1.377305	0.04694
Resp18	ENSMUSG00000033061	99.14150664	568.2662973	142.0296828	952.2519195	711.5808052	645.3601493	-1.512397	0.047151
Atp6v0a1	ENSMUSG00000019302	37.08639437	7.559058752	34.98308334	58.37737074	48.96051702	56.7833788	-1.043405	0.047269
Decr1	ENSMUSG00000028223	2.092815576	0	0	3.805098363	14.69479227	13.70242983	-3.943648	0.047313
Rtca	ENSMUSG00000000339	42.85983811	40.40489456	32.17285122	93.67660073	51.19445379	93.072867	-1.043509	0.047329
Plxna3	ENSMUSG00000031398	1.587881127	0	0.281966561	2.369576078	8.074959837	8.065423324	-3.307309	0.047496
Gcsh	ENSMUSG00000034424	49.43671739	96.21431511	40.1597345	104.307273	119.0797999	152.2611235	-1.015548	0.047614

Imp3	ENSMUSG00000032288	26.79290246	0.078182685	33.01622472	156.3078412	72.43378617	77.0914947	-2.352422	0.047631
Spg20	ENSMUSG00000036580	1.148313017	0.343197868	2.827698846	4.255014105	2.983258084	4.226208109	-1.408332	0.047638
Snx17	ENSMUSG00000029146	29.61692074	0.198656359	37.61865099	58.37568147	49.27656632	74.44205926	-1.433133	0.047777
Fam174b	ENSMUSG00000078670	7.311744658	0	0.043247331	7.623147604	12.06144004	11.21432866	-2.070761	0.047926
Cdk4	ENSMUSG00000006728	14.08315734	0.366873872	28.4077794	31.9555834	44.96277505	41.92502485	-1.471432	0.04808
Tubg2	ENSMUSG00000045007	84.68634621	16.51571324	27.74806946	117.4556802	89.40212269	116.3095651	-1.325468	0.048126
Lrrc6	ENSMUSG00000022375	0	0.017332246	0	6.172318664	1.534640798	3.623393842	-9.352519	0.048213
Gabarap	ENSMUSG00000018567	123.8312356	37.23692024	94.86456843	183.9709071	139.0572995	210.7779898	-1.060551	0.048249
2610507I01Rik	ENSMUSG00000085882	6.339128967	0	0	7.213489603	11.48860124	8.421015954	-2.097166	0.04838
Caln1	ENSMUSG00000060371	3.190398592	0.144715903	6.476321852	17.03443723	8.631323635	10.5327324	-1.883393	0.048436
Wdr31	ENSMUSG00000028391	0.007080115	2.416666713	0.014887378	6.449409213	3.529733217	3.320297228	-2.44722	0.048449
Hap1	ENSMUSG00000006930	9.076726318	6.67546431	1.975993061	37.98860168	69.24743997	24.13900402	-2.889575	0.04854
Fbxo42	ENSMUSG00000028920	4.05328355	0.127827504	4.320859497	8.505961854	10.76236686	5.811189356	-1.56064	0.048636
Gla	ENSMUSG00000031266	2.013762542	0	0.356462649	5.310421645	2.152942797	5.730498782	-2.476771	0.048785
Acaca	ENSMUSG00000020532	2.528591317	0.008462083	0.204495235	3.240898021	3.43408161	2.953316162	-1.812289	0.049061
Rapgef1	ENSMUSG00000039844	2.402195546	0.004825705	0.01413555	5.020925457	3.271363137	2.841560494	-2.201184	0.049101
Sap30l	ENSMUSG00000020519	7.082169594	13.11757369	4.504279034	18.59166129	17.19271019	13.63129228	-1.000222	0.049136
Terf2ip	ENSMUSG00000033430	4.682038111	1.602760803	12.46977649	12.655248	22.77184754	20.9077344	-1.586785	0.049174
Thap4	ENSMUSG00000026279	0.905663905	0.016622366	1.785320675	5.005675157	2.555184383	2.876519309	-1.946669	0.049231
Mid1ip1	ENSMUSG00000008035	37.94371408	9.912741429	18.82738412	48.32970853	51.52161641	40.90602024	-1.077801	0.049255
Garnl3	ENSMUSG00000038860	1.87661912	0	0.650999777	1.998775619	2.802279732	2.975141149	-1.621286	0.049297
4732471J01Rik	ENSMUSG00000053714	2.121766007	0.858818381	3.549615539	6.875969452	7.997939461	3.86389396	-1.520753	0.049323
Sdhaf2	ENSMUSG00000024668	9.417940528	6.044331054	1.529350648	12.75753392	10.92733924	13.41610881	-1.126634	0.049361
Cyp51	ENSMUSG00000001467	3.639487046	3.755212622	2.971635292	9.002318927	21.57330426	11.76334104	-2.03008	0.049395
Cnih2	ENSMUSG00000024873	82.55301426	9.920577431	36.16986895	246.2442514	199.8139592	95.48491203	-2.073698	0.049442
Syt5	ENSMUSG00000004961	15.01094464	0.015176639	2.963714153	192.3887801	89.20725638	65.05906831	-4.268247	0.049481
Slc6a1	ENSMUSG00000030310	2.676215081	5.507871548	0.253518749	17.96262576	18.40031031	6.644112734	-2.349668	0.049531
Prelid3b	ENSMUSG00000016257	32.67989268	26.6436501	12.40075364	46.40179181	88.22967601	54.8518624	-1.401537	0.049759
Zfp3	ENSMUSG00000043602	1.416043625	0	1.676993423	6.428147835	2.925727031	8.956027866	-2.565528	0.0498
Dleu7	ENSMUSG00000048281	0	0	7.737879472	19.04505634	8.480447363	13.51931648	-2.40719	0.049868
Tmem80	ENSMUSG00000025505	12.10723292	0.659214354	2.319400085	13.64415857	22.17122555	15.86687229	-1.776473	0.049904
Ddx39b	ENSMUSG00000019432	30.02520525	15.59202053	25.22752972	38.69537053	45.2207049	67.32318332	-1.0941	0.049913
Arpc4	ENSMUSG00000079426	27.12842026	11.90525903	30.61844887	73.08107553	41.84047823	97.71059376	-1.61012	0.049922
Wars	ENSMUSG00000021266	22.85031449	0.461066902	20.3915055	33.34439532	40.843357	32.55541639	-1.288343	0.049933
Tspan6	ENSMUSG00000067377	13.69506658	4.979705669	2.208542523	28.41555902	19.85931972	15.45664765	-1.609656	0.049965
Rps10-ps2	ENSMUSG00000099764	1.082115651	0.291293551	0.711052388	7.051096455	3.358315926	11.58723039	-3.399537	0.049998

Table S3. Intrinsic physiological properties of PV and Drd2 neurons.

	PV	Drd2	N	*P* Value	Test
Resting membrane potential (RMP，mV)	-58.94±1.71	-59.09±0.64	n=21 neurons per group	*P* =0.9154	unpaired t test
Membrane resistance (MR, MΩ)	361.5±21.17	296.7±14.35	n=21 neurons per group	**P* <0.05	unpaired t test
Cell capacitance (CC, Pf)	13.62±0.79	11.94±0.53	n=21 neurons per group	*P* =0.0865	unpaired t test
Amplitudes (AP,mV)	99.93±3.44	114.0±2.61	n=21 neurons per group	*****P* <0.0001	unpaired t test
Half width (HW,ms)	0.61±0.01	0.79±0.03	n=21 neurons per group	*****P* <0.0001	unpaired t test
Threshold (mV)	-33.68±1.64	-37.46±1.05	n=21 neurons per group	*P* =0.0598	unpaired t test
Amplitudes of afterhyperpolarization (AHP, mV)	-58.88±2.46	-59.87±2.20	n=21 neurons per group	*P* =0.7648	unpaired t test

	Frequency(HZ)				
current injection (pA)	**PV**	**Drd2**	**N**	***P* Value**	**Test**
0	0±0	0±0	n = 27 neurons per group	*P* >0.9999	two-way ANOVA
50	13.74±5.05	40.43±10.76	n = 27 neurons per group	***P<0.001	two-way ANOVA
100	29.59±7.14	103.29±11.25	n = 27 neurons per group	*****P* <0.0001	two-way ANOVA
150	52.41±10.40	138.57±9.14	n = 27 neurons per group	*****P* <0.0001	two-way ANOVA
200	71.81±12.26	160.86±10.18	n = 27 neurons per group	*****P* <0.0001	two-way ANOVA
250	87.44±12.13	170.86±9.08	n = 27 neurons per group	*****P* <0.0001	two-way ANOVA
300	105.19±12.26	183.08±7.86	n = 27 neurons per group	*****P* <0.0001	two-way ANOVA

Table S4. The sgRNAs and primer sequence

Target name	sgRNA index	sgRNA sequence 5'---3'	Exon	PAM	On-target score	Off-target score
Kcnn4	1	GCTGCCATTTGCGTACTGAG	1	AGG	71	84.5
Kcnn4	2	GCTGGAGCAGGAGAAGAGGG	1	TGG	69.3	32.7
Kcnn4	3	CAGGCGCTTTCTCCGTCTCA	1	GGG	54.8	81.3
Kcnn4	4	TGTGGTCTTCCATGCCAAGG	2	AGG	71.4	65.6
Kcnn4	5	GGGTGATCAAACACTTAACC	2	AGG	59.2	82.7
Cacna1h	1	CCCGAGGCAGAAGAAGACTG	2	TGG	74.5	36.2
Cacna1h	2	TCTGCCTCGGGCAAACCACG	2	CGG	70.1	46.6
Cacna1h	3	GCCGAAGGCGAAGCGCCCAG	2	GGG	68	47.1
Cacna1h	4	AGACTGTGGCAGCCAGAGCG	2	GGG	67	35.1
Cacna1h	5	CCCTCGGTCATGGTGGCAGA	2	GGG	54.3	39.3
Zyf2 (Control)	1	TAACGACTTTTCTTCTTCTG	3	GGG	63.6	31.8
Zyf2 (Control)	2	TTAGTAAGAACTAAAGGCCA	3	TGG	54.2	30.7
Zyf2 (Control)	3	TCTAAATCGAGAACTTGCTC	4	AGG	54.9	42.9
Zyf2 (Control)	4	ATAACTGAGTCTGGATCATC	4	AGG	43.7	41.8
Zyf2 (Control)	5	CTTGTACTTCTACAGAAATC	4	TGG	42	33.6

Kcnn4	Forward primer	TCACATTCCTGACCATTGGCTAT
	Reverse primer	GACTCCTTCATCTCTTTGGCATAAT
Cacna1h	Forward primer	GTTTGTAACCCGTGGTTCGAGCA
	Reverse primer	GAACGGCACTCAACATCCTCACA

Table S5. The exploration time (s) in trainning and testing sessions of SRM tasks.

Figure S6C, D; Figure 4D, G (trainning session)

PRM (n = 11)	PV^{Kcnn4+}			PV^{Kcnn4-}			Drd2$^{Cacna1h+}$			Drd2$^{Cacna1h-}$		
	Total	A	A'	Total	A	A'	Total	A	A'	Total	A	A'
	31.13	14.94	16.19	26.2	9.17	17.03	38.33	18.78	19.55	35.13	16.16	18.97
	29.77	16.67	13.1	34.09	15.34	18.75	27.42	10.42	17	33.05	14.87	18.18
	33	17.82	15.18	35.94	17.97	17.97	39.93	16.37	23.56	34.63	14.2	20.43
	32.19	11.59	20.6	29.68	10.09	19.59	32.45	15.9	16.55	31.87	19.44	12.43
	29.08	11.63	17.45	37.55	23.28	14.27	37.56	25.54	12.02	29.27	16.1	13.17
	28.81	16.42	12.39	35.55	20.62	14.93	26.76	13.38	13.38	34.75	14.94	19.81
	36.5	21.9	14.6	31.4	16.64	14.76	28.74	11.21	17.53	37.05	21.49	15.56
	40.91	22.5	18.41	27.11	11.93	15.18	28.65	12.89	15.76	35.04	18.22	16.82
	33.27	14.64	18.63	41.1	24.25	16.85	33.41	14.7	18.71	30.34	12.44	17.9
	31.31	13.15	18.16	46	24.84	21.16	32.73	14.73	18	27.11	12.47	14.64
	36.06	17.67	18.39	36.34	17.08	19.26	40.85	24.51	16.34	36.31	17.43	18.88
Mean	32.91	16.27	16.65	34.63	17.38	17.25	33.35	16.22	17.13	33.14	16.16	16.98

ORM (n = 13)	PV^{Kcnn4+}			PV^{Kcnn4-}			Drd2$^{Cacna1h+}$			Drd2$^{Cacna1h-}$		
	Total	A	B	Total	A	B	Total	A	B	Total	A	B
	31.19	17.88	13.31	36.11	17.55	18.56	36.78	21.29	15.49	34.92	15.27	19.65
	30.11	14.52	15.59	43.45	22.43	21.02	28.74	14.84	13.9	31.68	13.73	17.95
	29.92	15.37	14.55	37.8	18.86	18.94	32.33	13.64	18.69	36.19	14.61	21.58
	38.71	21.61	17.1	47.54	24.05	23.49	37.19	15.8	21.39	44	18.98	25.02
	31.09	11.21	19.88	27.7	13.48	14.22	36.25	12.82	23.43	35.76	21.5	14.26
	37.81	15.65	22.16	29.92	14.24	15.68	35.41	16.02	19.39	33.04	19.24	13.8
	37.11	18.68	18.43	32.24	15.59	16.65	40.78	15.81	24.97	29.61	17.45	12.16
	38.59	21.22	17.37	29.16	16	13.16	30.34	18.02	12.32	37.9	22.63	15.27
	38.74	22.89	15.85	28.65	12.96	15.69	35.59	18.96	16.63	41.02	24.73	16.29
	28.28	13.31	14.97	32.82	13.83	18.99	32.26	17.22	15.04	34.42	18.87	15.55
	25.21	11.85	13.36	34.48	17.24	17.24	38	19.76	18.24	33.81	16.57	17.24
	36.43	17.85	18.58	26.05	12.24	13.81	40.02	19.21	20.81	42.67	21.76	20.91
	32.28	15.97	16.31	39.6	18.3	21.3	37.43	22.96	14.47	36.04	16.54	19.5
Mean	33.5	16.77	16.73	34.27	16.67	17.6	35.47	17.41	18.06	36.24	18.61	17.63

Figure 4D, G (testing session)

PRM (n = 11)	PV^{Kcnn4+}			PV^{Kcnn4-}			Drd2$^{Cacna1h+}$			Drd2$^{Cacna1h-}$		
	Total	FL	NL	Total	FL	NL	Total	FL	NL	Total	FL	NL
	16.23	6.15	10.08	22.91	13.45	9.46	18.09	6.68	11.41	22.79	3.38	19.41
	17.03	3.61	13.42	22.89	11.22	11.67	25.41	7.61	17.8	19.28	2.59	16.69
	22.29	5.65	16.64	15.46	6.64	8.82	16.91	3.57	13.34	18.74	6.25	12.49
	26.06	7.71	18.35	16.01	8.07	7.94	22.61	6.85	15.76	24.21	8.78	15.43
	13.8	2.45	11.35	22.24	8.92	13.32	27.43	8.72	18.71	27.64	9.93	17.71
	19.09	5.65	13.44	24.52	9.16	15.36	37.61	11.29	26.32	17.17	5.7	11.47
	16.48	7.62	8.86	17.4	8.74	8.66	24.54	7.78	16.76	15.8	6.76	9.04
	25.42	9.85	15.57	22.41	13.36	9.05	18.61	6.42	12.19	20.51	2.86	17.65
	18.51	2.46	16.05	26.79	16.59	10.2	18.95	3.4	15.55	34.61	9.22	25.39
	33.8	5.76	28.04	24.14	8.13	16.01	12.22	2.39	9.83	18.57	10.76	7.81
	25.7	11.67	14.03	17.61	10.64	6.97	23.41	13.26	10.15	24.21	7.8	16.41
Mean	21.31	6.23	15.08	21.13	10.45	10.68	22.35	7.09	15.26	22.14	6.73	15.41

ORM (n = 13)	PV^{Kcnn4+}			PV^{Kcnn4-}			Drd2$^{Cacna1h+}$			Drd2$^{Cacna1h-}$		
	Total	FO	NO	Total	FO	NO	Total	FO	NO	Total	FO	NO
	14.02	2.9	11.12	17.53	5.04	12.49	17.54	5.6	11.94	22	8.29	13.71
	23.65	5.06	18.59	24.31	8.7	15.61	22.68	8.53	14.15	21.91	10.64	11.27
	26.15	8.56	17.59	29.09	11.78	17.31	27.34	11.73	15.61	21.43	11.52	9.91
	18.26	3.53	14.73	16.08	1.7	14.38	31.96	14.53	17.43	26.95	15.67	11.28
	22.99	10.66	12.33	13.53	2.49	11.04	13	1.55	11.45	22.25	6.27	15.98
	10.72	1.82	8.9	19.51	2.65	16.86	17.94	1.63	16.31	12.75	5.78	6.97
	13.16	2.92	10.24	17.97	2.87	15.1	27.59	1.84	25.75	16.23	8.84	7.39
	20.03	3.32	16.71	20.26	3.8	16.46	17.52	3.13	14.39	19.74	11.07	8.67
	31.03	13.01	18.02	16.08	4.91	11.17	15.75	3.14	12.61	26.45	14.87	11.58
	25.2	8.55	16.65	22.1	5.68	16.42	21.71	3.56	18.15	13.8	4.75	9.05
	21.82	7.53	14.29	20.85	5.78	15.07	12.72	4.85	7.87	9.98	5.4	4.58
	21.13	4.87	16.26	25.81	7.4	18.41	24.64	6.1	18.54	13.7	6.06	7.64
	17.52	4.98	12.54	19.93	12.57	7.36	18.05	2.51	15.54	24.03	11.61	12.42
Mean	20.44	5.98	14.46	20.23	5.8	14.44	20.65	5.28	15.36	19.32	9.29	10.03

Figure S6E (training session)

PRM (n = 11)	PV^{Kcnn4+}			PV^{Kcnn4-}			Drd2$^{Cacna1h+}$			Drd2$^{Cacna1h-}$		
	Total	A	A'	Total	A	A'	Total	A	A'	Total	A	A'
	34.98	16.44	18.54	25.6	11.52	14.08	40	15.6	24.4	32.52	12.36	20.16
	38.67	14.31	24.36	36.76	24.26	12.5	44.85	27.36	17.49	54	36.18	17.82
	37.66	14.31	23.35	25.93	11.67	14.26	47.08	24.48	22.6	48.54	30.58	17.96
	42.23	29.14	13.09	36.07	16.23	19.84	39.28	18.46	20.82	38.71	25.16	13.55
	32.54	21.15	11.39	37.32	13.81	23.51	50.14	28.58	21.56	58.89	38.28	20.61
	28.86	12.41	16.45	31.95	18.85	13.1	27.53	10.46	17.07	24.51	7.35	17.16
	32.21	18.68	13.53	41.07	18.89	22.18	26.7	9.88	16.82	34.24	17.12	17.12
	29.13	16.02	13.11	28.48	9.97	18.51	36.02	17.29	18.73	37.58	15.03	22.55
	34.02	17.35	16.67	58.86	34.14	24.72	41.58	18.71	22.87	30.55	8.25	22.3
	33.58	11.75	21.83	32.49	13.97	18.52	22.12	7.74	14.38	34.92	16.76	18.16
	26.4	11.09	15.31	45.2	23.05	22.15	38.54	20.04	18.5	32.57	17.59	14.98
Mean	33.66	16.6	17.06	36.34	17.85	18.49	37.62	18.05	19.57	38.82	20.42	18.4

ORM (n = 11)	PV^{Kcnn4+}			PV^{Kcnn4-}			Drd2$^{Cacna1h+}$			Drd2$^{Cacna1h-}$		
	Total	A	B	Total	A	B	Total	A	B	Total	A	B
	38	17.1	20.9	37.24	20.11	17.13	48.98	25.47	23.51	44.19	21.21	22.98
	31.88	13.07	18.81	36.86	13.27	23.59	43.04	18.51	24.53	32.52	17.56	14.96
	38.86	16.71	22.15	45.36	24.04	21.32	33.36	15.01	18.35	36.92	22.52	14.4
	32.31	17.77	14.54	31.26	17.82	13.44	23.41	10.3	13.11	32.5	17.55	14.95
	29.18	16.34	12.84	30.41	16.42	13.99	36.49	20.8	15.69	48.46	28.59	19.87
	38.62	18.15	20.47	33.31	19.32	13.99	30.8	17.25	13.55	27.54	11.29	16.25
	29.51	15.64	13.87	31.04	10.24	20.8	36.94	19.58	17.36	34.44	9.64	24.8
	42.25	19.01	23.24	41.65	22.49	19.16	38.29	19.91	18.38	48	25.44	22.56
	31.94	16.93	15.01	35.02	16.81	18.21	25.9	9.84	16.06	24.87	11.69	13.18
	34.29	10.29	24	45.67	24.66	21.01	38.39	19.58	18.81	34.53	15.54	18.99
	33.09	13.9	19.19	27.22	11.43	15.79	41.05	16.42	24.63	38.22	19.49	18.73
Mean	34.54	15.9	18.64	35.91	17.87	18.04	36.06	17.52	18.54	36.56	18.23	18.33

Figure S6F (testing session)

PRM (n = 11)	PV^{Kcnn4+}			PV^{Kcnn4-}			Drd2$^{Cacna1h+}$			Drd2$^{Cacna1h-}$		
	Total	FL	NL	Total	FL	NL	Total	FL	NL	Total	FL	NL
	22.08	6.4	15.68	23.3	7.92	15.38	21.51	4.09	17.42	24.52	9.32	15.2
	20.51	6.56	13.95	21.51	5.81	15.7	22.64	6.34	16.3	21.1	2.95	18.15
	26.74	9.09	17.65	22.53	7.21	15.32	24.51	7.35	17.16	20.83	5.83	15
	22.74	7.28	15.46	21.31	2.77	18.54	22.56	2.26	20.3	20.22	3.84	16.38
	27.49	5.5	21.99	22.92	8.25	14.67	21.22	7.64	13.58	21.31	2.77	18.54
	20.75	5.81	14.94	24.77	6.69	18.08	24.07	4.33	19.74	23.8	9.28	14.52
	20.71	7.87	12.84	21.3	5.11	16.19	20.03	7.01	13.02	21.87	6.56	15.31
	20.48	3.28	17.2	22.44	2.24	20.2	26.51	6.1	20.41	24.34	8.03	16.31
	22.08	2.21	19.87	18.87	3.02	15.85	20.96	6.71	14.25	23.07	2.54	20.53
	26.6	5.85	20.75	21.33	5.33	16	22.13	7.97	14.16	21.7	2.17	19.53
	23.89	7.17	16.72	27.98	11.19	16.79	24.88	4.23	20.65	28.46	10.53	17.93
Mean	23.1	6.09	17	22.57	5.96	16.61	22.82	5.82	17	22.84	5.8	17.04

ORM (n = 11)	PV^{Kcnn4+}			PV^{Kcnn4-}			$Drd2^{Cacna1h+}$			$Drd2^{Cacna1h-}$		
	Total	FO	NO	Total	FO	NO	Total	FO	NO	Total	FO	NO
	20.29	6.49	13.8	24.72	9.89	14.83	22.04	6.17	15.87	25.6	7.68	17.92
	23.74	6.17	17.57	20.2	2.83	17.37	21.01	2.73	18.28	22.72	7.5	15.22
	22.58	7.45	15.13	21.89	6.35	15.54	21.53	7.75	13.78	20.93	3.77	17.16
	21.97	7.91	14.06	20.91	2.51	18.4	20.63	4.13	16.5	20.88	5.22	15.66
	21.01	2.52	18.49	24.61	4.43	20.18	23.62	5.2	18.42	23.3	2.33	20.97
	29.36	7.93	21.43	21.62	4.97	16.65	21.64	8.44	13.2	23.44	9.14	14.3
	22.43	5.83	16.6	24.99	4.25	20.74	22.65	7.25	15.4	21.84	5.02	16.82
	21.08	4.85	16.23	27.53	7.16	20.37	24.24	3.64	20.6	20.91	3.14	17.77
	23.04	9.91	13.13	20.1	5.63	14.47	26.29	9.99	16.3	25.07	8.27	16.8
	23.91	2.87	21.04	23.59	5.66	17.93	20.15	2.42	17.73	20.14	6.04	14.1
	23.32	5.36	17.96	21.52	7.96	13.56	21.45	6.65	14.8	27.41	7.4	20.01
Mean	22.98	6.12	16.86	22.88	5.6	17.28	22.3	5.85	16.44	22.93	5.96	16.98

Figure S9C; Figure 6B (training session)

PRM (n = 11)	DS^{GFP}						DS^{hM4Di}						VS^{tdT}						VS^{hM4Di}					
	CNO			Saline			CNO			Saline			CNO			Saline			CNO			Saline		
	Total	A	A'	Total	A	A'	Total	A	A'	Total	A	A'	Total	A	A'	Total	A	A'	Total	A	A'	Total	A	A'
	28.62	10.59	18.03	32	13.12	18.88	37.6	21.43	16.17	30.07	13.23	16.84	32.98	13.52	19.46	28.81	13.25	15.56	26.09	12	14.09	33.33	19.33	14
	30.13	16.57	13.56	35.97	15.11	20.86	42.18	23.62	18.56	32.3	13.89	18.41	36	15.48	20.52	37.89	23.49	14.4	32.8	14.76	18.04	26.42	10.83	15.59
	34.96	19.23	15.73	30.58	12.23	18.35	40.88	24.53	16.35	36.47	24.07	12.4	39.09	21.5	17.59	30.05	17.73	12.32	35.65	22.46	13.19	35.53	17.41	18.12
	30.46	17.97	12.49	41.09	22.6	18.49	26.77	9.64	17.13	32.63	11.75	20.88	32.38	17.16	15.22	31.58	11.05	20.53	41.64	18.74	22.9	37.32	16.42	20.9
	30.89	14.52	16.37	31.71	15.22	16.49	28.72	10.34	18.38	39.03	25.76	13.27	34.47	18.27	16.2	24.28	11.41	12.87	35.08	16.84	18.24	24.74	9.65	15.09
	29.47	11.2	18.27	33.75	20.25	13.5	26.81	12.6	14.21	32.29	17.76	14.53	32.56	12.37	20.19	36.18	19.9	16.28	35.95	21.21	14.74	34.8	22.62	12.18
	38.91	18.29	20.62	24.7	11.61	13.09	36.3	21.78	14.52	29.64	10.67	18.97	35.97	23.38	12.59	39.94	18.77	21.17	32.54	19.85	12.69	33.77	13.17	20.6
	36.92	17.72	19.2	36.11	16.25	19.86	24.78	12.14	12.64	23.44	8.91	14.53	29.93	17.06	12.87	40.37	19.38	20.99	38	25.08	12.92	36.62	22.34	14.28
	30.29	15.45	14.84	30.21	17.52	12.69	36.57	17.19	19.38	35.9	17.59	18.31	28.88	16.75	12.13	30.36	13.66	16.7	30.57	14.37	16.2	27.77	11.11	16.66
	43.18	25.91	17.27	33.65	14.47	19.18	28.98	12.46	16.52	27.5	14.3	13.2	30.18	15.09	15.09	32.13	17.03	15.1	24.59	10.82	13.77	33.7	18.2	15.5
	27.3	10.65	16.65	31.91	18.19	13.72	31.25	10.31	20.94	40.61	20.71	19.9	31.52	15.13	16.39	31.23	12.18	19.05	30.39	10.03	20.36	40.04	22.02	18.02
Mean	32.83	16.19	16.64	32.88	16.05	16.83	32.8	16	16.8	32.72	16.24	16.48	33.08	16.88	16.2	32.99	16.17	16.82	33.02	16.92	16.1	33.1	16.65	16.45
ORM (n = 11)	DS^{GFP}						DS^{hM4Di}						VS^{tdT}						VS^{hM4Di}					
	CNO			Saline			CNO			Saline			CNO			Saline			CNO			Saline		
	Total	A	B	Total	A	B	Total	A	B	Total	A	B	Total	A	B	Total	A	B	Total	A	B	Total	A	B
	34.61	13.55	21.06	30.79	16.89	13.9	35.25	17.45	17.8	38.77	21.99	16.78	46.76	25.25	21.51	37.36	20.92	16.44	32.65	16	16.65	32.46	14.28	18.18
	28.35	16.94	11.41	36.79	22.53	14.26	40.61	20.53	20.08	32.85	12.72	20.13	29.79	17.28	12.51	25.36	14.71	10.65	27.55	12.12	15.43	35.06	23.84	11.22
	27.67	12.76	14.91	31.22	14.95	16.27	38.37	16.05	22.32	33.83	16.74	17.09	41.44	19.06	22.38	21.24	8.07	13.17	23.85	11.21	12.64	27.61	10.49	17.12
	32	18.13	13.87	33.91	13.73	20.18	39.49	22.26	17.23	42.92	22.78	20.14	27.97	9.51	18.46	40.14	20.07	20.07	32.79	11.15	21.64	34.95	20.97	13.98
	29.18	13.11	16.07	33.02	15.38	17.64	30.05	13.56	16.49	36.39	17.7	18.69	36.43	21.86	14.57	32.41	14.26	18.15	33.52	15.42	18.1	34.88	14.3	20.58
	36.58	21.16	15.42	31.19	12.27	18.92	25.09	11.86	13.23	34.31	16.81	17.5	27.44	10.15	17.29	33.79	14.53	19.26	40.5	24.3	16.2	28.29	9.9	18.39
	32.5	13.29	19.21	37.9	14.81	23.09	27.13	14.18	12.95	36.12	19.13	16.99	41.4	23.6	17.8	29.89	19.43	10.46	38.42	25.74	12.68	39.43	18.14	21.29
	34.42	16.61	17.81	29.2	13.89	15.31	30.66	16.28	14.38	34.32	18.6	15.72	31.76	15.56	16.2	36.05	20.55	15.5	33.56	19.13	14.43	30.72	16.59	14.13
	35.99	15.58	20.41	31.1	17.57	13.53	31.3	15.31	15.99	28.3	13.86	14.44	26.76	10.17	16.59	31.41	17.59	13.82	35.49	20.94	14.55	27.3	10.65	16.65
	32.52	14.73	17.79	36.86	19.78	17.08	27.11	13.42	13.69	30.25	14.5	15.75	20.97	8.81	12.16	26.3	9.47	16.83	31.47	14.79	16.68	39.85	21.52	18.33
	36.33	21.53	14.8	28.61	12.32	16.29	32.91	11.41	21.5	32.97	16.63	16.34	32.42	16.86	15.56	41.43	17.4	24.03	31.82	10.82	21	38.17	24.43	13.74
Mean	32.74	16.13	16.61	32.78	15.83	16.95	32.54	15.66	16.88	34.64	17.41	17.23	33.01	16.19	16.82	32.31	16.09	16.22	32.87	16.51	16.36	33.52	16.83	16.69

Figure S9D; Figure 6B (testing session)

PRM (n = 11)	DS^{GFP}						DS^{hM4Di}						VS^{tdT}						VS^{hM4Di}					
	CNO			Saline			CNO			Saline			CNO			Saline			CNO			Saline		
	Total	FL	NL	Total	FL	NL	Total	FL	NL	Total	FL	NL	Total	FL	NL	Total	FL	NL	Total	FL	NL	Total	FL	NL
	21.85	7.65	14.2	20.09	3.82	16.27	20.08	6.83	13.25	20.07	4.82	15.25	28.8	10.37	18.43	20.76	5.81	14.95	21.1	6.33	14.77	21.04	5.47	15.57
	25.74	5.92	19.82	21.72	6.3	15.42	20.34	9.56	10.78	20.9	6.48	14.42	25.43	10.68	14.75	28.06	8.7	19.36	22.07	2.87	19.2	20.68	9.1	11.58
	21.73	6.3	15.43	20.82	7.29	13.53	22.02	10.79	11.23	22.53	4.51	18.02	21.38	7.7	13.68	27.3	9.01	18.29	21.26	6.38	14.88	27.16	8.15	19.01
	22.72	7.04	15.68	23.23	10.22	13.01	20.58	11.73	8.85	21.31	7.46	13.85	24.34	3.41	20.93	22.54	6.99	15.55	30.72	12.28	18.44	22.63	8.6	14.03
	23.04	4.84	18.2	24.27	9.71	14.56	20.22	6.67	13.55	26.01	5.46	20.55	26.01	7.02	18.99	22.63	7.92	14.71	20.51	4.72	15.79	20.35	6.51	13.84
	23.87	3.1	20.77	20.42	3.47	16.95	22.89	10.3	12.59	20.91	7.32	13.59	20.87	9.39	11.48	22	6.82	15.18	21.59	4.75	16.84	25.28	3.79	21.49
	20.4	6.73	13.67	23.16	7.64	15.52	26.53	10.08	16.45	21.6	10.15	11.45	20.16	2.62	17.54	20.86	3.96	16.9	20.12	4.43	15.69	21.13	7.61	13.52
	20.44	4.29	16.15	20.99	4.83	16.16	22.68	12.02	10.66	25.13	5.53	19.6	20	4.4	15.6	20.93	8.16	12.77	24.61	2.46	22.15	21.07	6.11	14.96
	23.9	4.06	19.84	22.78	4.1	18.68	21.18	7.2	13.98	22.33	5.36	16.97	20.64	5.16	15.48	22.51	6.75	15.76	22.61	9.27	13.34	20.65	9.29	11.36
	24.85	15.16	9.69	22.96	6.2	16.76	24.57	10.81	13.76	20.79	6.03	14.76	25.68	6.93	18.75	20.78	5.82	14.96	22.56	4.29	18.27	23.73	10.44	13.29
	22.85	7.31	15.54	22.79	8.66	14.13	25.49	13.51	11.98	22.28	6.24	16.04	22.14	9.3	12.84	24.3	10.45	13.85	33.1	14.85	18.25	25.08	9.53	15.55
Mean	22.85	6.58	16.27	22.11	6.57	15.54	22.42	9.95	12.46	22.17	6.31	15.86	23.22	7	16.22	22.97	7.31	15.66	23.66	6.6	17.06	22.62	7.69	14.93
ORM (n = 11)	DS^{GFP}						DS^{hM4Di}						VS^{tdT}						VS^{hM4Di}					
	CNO			Saline			CNO			Saline			CNO			Saline			CNO			Saline		
	Total	FO	NO	Total	FO	NO	Total	FO	NO	Total	FO	NO	Total	FO	NO	Total	FO	NO	Total	FO	NO	Total	FO	NO
	24.04	7.93	16.11	23.81	5	18.81	22.21	9.55	12.66	20.99	3.57	17.42	21.27	5.53	15.74	21.93	6.36	15.57	21.19	8.05	13.14	25.73	5.66	20.07
	20.48	5.94	14.54	24.82	8.69	16.13	24.64	5.67	18.97	26.35	8.43	17.92	20.53	2.67	17.86	28.85	12.98	15.87	23.47	13.38	10.09	20.14	6.24	13.9
	21.35	5.55	15.8	23.02	7.83	15.19	21.21	4.67	16.54	28.15	11.54	16.61	20	4.4	15.6	23.7	11.14	12.56	22.63	11.09	11.54	21.74	10.22	11.52
	21.23	2.55	18.68	20.64	9.08	11.56	20.18	2.62	17.56	21.33	7.68	13.65	21.79	6.97	14.82	20.71	4.97	15.74	21.87	7	14.87	22.84	8.91	13.93
	24.02	4.56	19.46	25.91	6.74	19.17	20.25	8.1	12.15	20.71	5.18	15.53	21.75	7.61	14.14	23.59	7.55	16.04	20.91	11.71	9.2	25.05	6.76	18.29
	26.78	8.3	18.48	20.33	4.27	16.06	23.72	2.85	20.87	20.78	2.91	17.87	20.82	4.58	16.24	21.27	2.55	18.72	21.53	8.83	12.7	26.63	6.39	20.24
	21.55	8.19	13.36	26.56	10.89	15.67	20.34	3.46	16.88	21.9	6.35	15.55	26.79	8.84	17.95	20.47	5.12	15.35	22.54	7.89	14.65	23.44	4.69	18.75
	20.18	9.08	11.1	20.67	2.48	18.19	22.84	7.08	15.76	22.02	9.03	12.99	25.17	6.04	19.13	20.15	2.62	17.53	25.62	11.53	14.09	20.64	6.4	14.24
	21.18	5.51	15.67	21.73	7.39	14.34	21.2	9.33	11.87	24.2	8.47	15.73	23.31	6.53	16.78	22.12	6.86	15.26	22.62	13.12	9.5	20.87	6.26	14.61
	20.64	6.81	13.83	21.92	7.67	14.25	24.82	4.47	20.35	23.9	10.04	13.86	22.8	10.26	12.54	27.3	7.37	19.93	25.67	13.35	12.32	21.26	5.95	15.31
	23.44	4.92	18.52	20.93	5.86	15.07	21.61	5.19	16.42	21.32	7.46	13.86	25.12	4.77	20.35	21.66	9.1	12.56	23.05	9.45	13.6	20.35	4.27	16.08
Mean	22.26	6.3	15.96	22.76	6.9	15.86	22.09	5.73	16.37	22.88	7.33	15.54	22.67	6.2	16.47	22.89	6.97	15.92	22.83	10.49	12.34	22.61	6.52	16.09

Figure S10F (training session)

PRM (n = 11)	DS^{hM4Di}-PV^{tdT}			DS^{hM4Di}-PV^{ChR2}			VS^{hM4Di}-$Drd2^{GFP}$			VS^{hM4Di}-$Drd2^{ChR2}$		
	Total	A	A'	Total	A	A'	Total	A	A'	Total	A	A'
	42.59	21.72	20.87	33.67	19.53	14.14	29.02	11.03	17.99	33.25	15.63	17.62
	33.89	17.96	15.93	42.55	22.55	20	39.39	21.27	18.12	31.41	18.53	12.88
	26.28	11.04	15.24	36.89	20.29	16.6	38.02	19.39	18.63	34.81	14.62	20.19
	28.52	13.12	15.4	27.28	9	18.28	31.13	14.63	16.5	36.52	18.99	17.53
	38.42	15.75	22.67	33.33	15.33	18	39.9	24.34	15.56	34.42	17.21	17.21
	38.27	21.05	17.22	37.19	23.8	13.39	37.94	20.11	17.83	32.98	14.18	18.8
	39	23.01	15.99	42.08	20.62	21.46	32.71	19.3	13.41	41.43	19.06	22.37
	22.64	9.51	13.13	38.15	22.89	15.26	27.55	11.57	15.98	31.68	18.69	12.99
	38.48	18.47	20.01	30.71	10.44	20.27	34.98	12.24	22.74	43.6	22.67	20.93
	42.89	24.02	18.87	39.04	20.3	18.74	29.95	11.98	17.97	26.07	10.95	15.12
	36.27	20.31	15.96	32.04	10.57	21.47	38.23	24.85	13.38	41.42	18.64	22.78
Mean	35.2	17.81	17.39	35.72	17.76	17.96	34.44	17.34	17.1	35.2	17.2	18
ORM (n = 11)	DS^{hM4Di}-PV^{tdT}			DS^{hM4Di}-PV^{ChR2}			VS^{hM4Di}-$Drd2^{GFP}$			VS^{hM4Di}-$Drd2^{ChR2}$		
	Total	A	B	Total	A	B	Total	A	B	Total	A	B
	31.55	17.01	14.54	34.05	16.27	17.78	36.13	18.37	17.76	36.39	22.39	14
	31.99	14.54	17.45	36.78	15.69	21.09	36.24	21.19	15.05	33.82	18.14	15.68
	32.12	15.98	16.14	37.1	18.59	18.51	30.37	18.36	12.01	37.52	19.52	18
	27.52	12.3	15.22	39.49	21.91	17.58	42.8	23.36	19.44	41.96	21.64	20.32
	41.37	21.61	19.76	35.51	19.23	16.28	32.23	14.16	18.07	34.98	13.11	21.87
	42.08	19.94	22.14	33.17	17.46	15.71	32.44	11.27	21.17	36.74	18.98	17.76
	34.2	16.79	17.41	26.08	13.94	12.14	33.53	15.59	17.94	35.23	16.83	18.4
	34.59	18.88	15.71	29.01	14.92	14.09	32.03	13.18	18.85	28.75	15.43	13.32
	28.2	13.99	14.21	37.27	18.25	19.02	31.13	16.86	14.27	29.88	14.85	15.03
	34.22	15.8	18.42	38.68	20.46	18.22	28.86	14.16	14.7	31.73	15.62	16.11
	37.76	22.23	15.53	30.27	13.01	17.26	34.45	12.09	22.36	34.78	17.1	17.68
Mean	34.15	17.19	16.96	34.31	17.25	17.06	33.66	16.24	17.42	34.71	17.6	17.11

Figure S10F (testing session)

PRM (n = 11)	DS^{hM4Di}-PV^{tdT}			DS^{hM4Di}-PV^{ChR2}			VS^{hM4Di}-$Drd2^{GFP}$			VS^{hM4Di}-$Drd2^{ChR2}$		
	Total	FL	NL	Total	FL	NL	Total	FL	NL	Total	FL	NL

	21.34	6.03	15.31	20.15	7.86	12.29	20.2	4.65	15.55	25.06	4.01	21.05
	26.26	15.13	11.13	21.34	11.31	10.03	26.64	9.06	17.58	21.42	7.16	14.26
	21.19	5.72	15.47	21.34	6.83	14.51	29.39	11.97	17.42	20	7.6	12.4
	22.81	8.44	14.37	21.11	13.3	7.81	24.26	5.37	18.89	22.33	4.02	18.31
	21.96	12.54	9.42	22.46	13.7	8.76	21.15	9.94	11.21	21.12	5.7	15.42
	22.77	13.89	8.88	24.66	13.19	11.47	26.04	8.71	17.33	23.45	5.39	18.06
	23.7	11.85	11.85	25.14	16.34	8.8	21.64	5.67	15.97	20.35	5.3	15.05
	20.23	7.08	13.15	26.31	15.66	10.65	20.71	5.34	15.37	20.1	4.42	15.68
	29.35	15.85	13.5	20.15	5.53	14.62	20.18	3.03	17.15	27.26	7.63	19.63
	22.5	14.47	8.03	29.93	13.77	16.16	20.25	6.31	13.94	28	10.92	17.08
	20.85	9.73	11.12	24.02	8.41	15.61	25.77	7.73	18.04	26.09	9.39	16.7
Mean	23	10.98	12.02	23.33	11.45	11.88	23.29	7.07	16.22	23.2	6.5	16.69

ORM (n = 11)	DS^{hM4Di}-PV^{tdT}			DS^{hM4Di}-PV^{ChR2}			VS^{hM4Di}-$Drd2^{GFP}$			VS^{hM4Di}-$Drd2^{ChR2}$		
	Total	FO	NO	Total	FO	NO	Total	FO	NO	Total	FO	NO
	24.74	10.1	14.64	21.62	6.32	15.3	20.06	10.03	10.03	25.26	14.65	10.61
	27.12	10.95	16.17	22.47	5.62	16.85	30.62	19.12	11.5	23.51	11.36	12.15
	20.91	4.27	16.64	20.01	5.86	14.15	25.98	15.69	10.29	25.78	12.63	13.15
	21.01	6.2	14.81	21.59	6.31	15.28	25.94	9.85	16.09	23.75	10.45	13.3
	24.39	5.22	19.17	21.12	3.8	17.32	26	15.08	10.92	24.57	11.55	13.02
	21.69	8.14	13.55	28.98	11.01	17.97	23.75	14.25	9.5	24.21	12.58	11.63
	24.31	5.33	18.98	26.44	10.31	16.13	21.12	5.7	15.42	22.67	12.4	10.27
	24.01	5.76	18.25	22.58	4.97	17.61	22.82	13.58	9.24	22.45	6.16	16.29
	21.89	5.58	16.31	21.52	4.09	17.43	20.84	8.56	12.28	21.93	9.62	12.31
	26.51	6.14	20.37	20.71	3.96	16.75	21	7.22	13.78	25.39	11.52	13.87
	20.09	6.17	13.92	21.31	9.34	11.97	22.92	14.21	8.71	24.04	12.27	11.77
Mean	23.33	6.71	16.62	22.58	6.51	16.07	23.73	12.12	11.61	23.96	11.38	12.58

Figure 6D (training session)

PRM (n = 11)	DS^{hM4Di}-PV^{tdT}			DS^{hM4Di}-PV^{ChR2}			VS^{hM4Di}-$Drd2^{GFP}$			VS^{hM4Di}-$Drd2^{ChR2}$		
	Total	A	A'	Total	A	A'	Total	A	A'	Total	A	A'
	23.61	10.15	13.46	43.1	25	18.1	40.96	22.53	18.43	43.23	20.32	22.91
	35.24	21.85	13.39	30.02	12.91	17.11	35.37	15.21	20.16	41.54	19.11	22.43
	46.9	28.61	18.29	27.22	13.88	13.34	24.4	11.47	12.93	41.3	21.89	19.41
	42.98	21.49	21.49	32.49	15.27	17.22	42.95	26.63	16.32	35.15	20.74	14.41
	34.73	11.81	22.92	30.96	17.03	13.93	36.89	19.92	16.97	27.55	12.12	15.43
	32.02	13.77	18.25	29.3	15.53	13.77	31.06	15.53	15.53	29.5	10.03	19.47
	29.47	10.61	18.86	27.21	11.97	15.24	36.44	20.77	15.67	34.1	20.46	13.64
	23.71	9.72	13.99	45.09	24.8	20.29	37.26	14.53	22.73	37	18.87	18.13
	31.65	12.03	19.62	44.7	25.48	19.22	37.85	19.68	18.17	33.79	19.6	14.19
	35.62	21.73	13.89	35.98	13.67	22.31	36.9	14.02	22.88	33.73	14.84	18.89
	45.44	26.81	18.63	34.83	16.37	18.46	30	16.8	13.2	32.5	16.25	16.25
Mean	34.67	17.14	17.53	34.63	17.45	17.18	35.46	17.92	17.54	35.4	17.66	17.74

ORM (n = 11)	DS^{hM4Di}-PV^{tdT}			DS^{hM4Di}-PV^{ChR2}			VS^{hM4Di}-$Drd2^{GFP}$			VS^{hM4Di}-$Drd2^{ChR2}$		
	Total	A	B	Total	A	B	Total	A	B	Total	A	B
	35.88	18.66	17.22	34.59	11.76	22.83	42.71	24.77	17.94	25.93	11.15	14.78
	39.35	18.1	21.25	41.21	23.49	17.72	53.4	27.77	25.63	34.25	19.18	15.07
	26.79	11.25	15.54	23.95	9.82	14.13	29.49	10.91	18.58	43.17	20.29	22.88
	30.17	15.69	14.48	31.9	19.14	12.76	26.17	13.87	12.3	29.63	15.41	14.22
	40.75	19.15	21.6	28.15	10.7	17.45	37.03	24.81	12.22	39.02	18.73	20.29
	23.13	8.79	14.34	25.39	11.68	13.71	35.15	15.82	19.33	38.28	19.14	19.14
	33.24	13.63	19.61	32.07	18.92	13.15	31.55	18.3	13.25	39.7	21.44	18.26
	50.85	30	20.85	34.48	20.69	13.79	28.34	12.47	15.87	39.77	18.69	21.08
	37.41	19.08	18.33	35.98	19.79	16.19	32.65	13.06	19.59	38.2	18.72	19.48
	48.26	27.51	20.75	43.19	25.05	18.14	33.54	13.08	20.46	32.62	17.94	14.68
	30.74	17.52	13.22	33.51	12.4	21.11	41.81	17.98	23.83	25.13	10.05	15.08
Mean	36.05	18.13	17.93	33.13	16.68	16.45	35.62	17.53	18.09	35.06	17.34	17.72

Figure S10H; Figure 6D (testing session)

PRM (n = 11)	DS^{hM4Di}-PV^{tdT}			DS^{hM4Di}-PV^{ChR2}			VS^{hM4Di}-$Drd2^{GFP}$			VS^{hM4Di}-$Drd2^{ChR2}$		
	Total	FL	NL	Total	FL	NL	Total	FL	NL	Total	FL	NL
	28.45	19.06	9.39	20.22	4.65	15.57	21.31	8.31	13	23.2	2.55	20.65
	21.08	9.91	11.17	25.58	4.86	20.72	23.02	2.53	20.49	22.57	4.74	17.83
	20.5	6.92	13.58	21.35	11.1	10.25	23.91	3.35	20.56	20.31	8.33	11.98
	25.78	16.24	9.54	23.13	3.93	19.2	26.87	8.06	18.81	25.67	11.04	14.63
	21.63	8.65	12.98	20.95	7.54	13.41	20.89	5.01	15.88	22.16	5.98	16.18
	21.32	6.61	14.71	24.81	10.42	14.39	20.2	2.42	17.78	21.23	8.07	13.16
	20.69	13.45	7.24	22.97	8.5	14.47	20.7	6.83	13.87	20.66	3.1	17.56
	21.49	7.95	13.54	20.8	9.15	11.65	22.2	8.66	13.54	22.31	3.35	18.96
	23.47	11.5	11.97	20.92	5.65	15.27	24.3	4.13	20.17	23.68	9	14.68
	25.95	15.31	10.64	21.29	5.32	15.97	21.27	7.87	13.4	22.35	5.81	16.54
	25.75	12.62	13.13	20.86	4.8	16.06	23.4	10.53	12.87	22.1	3.09	19.01
Mean	23.29	11.66	11.63	22.08	6.9	15.18	22.55	6.15	16.4	22.38	5.91	16.47

ORM (n = 11)	DS^{hM4Di}-PV^{tdT}			DS^{hM4Di}-PV^{ChR2}			VS^{hM4Di}-$Drd2^{GFP}$			VS^{hM4Di}-$Drd2^{ChR2}$		
	Total	FO	NO	Total	FO	NO	Total	FO	NO	Total	FO	NO
	21.17	5.08	16.09	22.33	10.05	12.28	22.41	11.43	10.98	24.04	7.93	16.11
	23.44	6.8	16.64	25.61	11.27	14.34	28.49	17.95	10.54	23.07	7.61	15.46
	21.07	5.27	15.8	21.88	4.38	17.5	20.89	7.73	13.16	25.9	7.25	18.65
	20.12	6.64	13.48	20.36	3.87	16.49	26.2	17.03	9.17	27.61	9.39	18.22
	29.82	9.54	20.28	23.15	10.42	12.73	23	13.57	9.43	23.26	11.63	11.63
	20.64	4.95	15.69	23.94	5.27	18.67	20.54	11.09	9.45	21.98	8.35	13.63
	20.83	3.33	17.5	21.99	6.38	15.61	26.76	13.11	13.65	20.7	4.97	15.73
	22.49	2.7	19.79	26.96	6.74	20.22	22.51	7.2	15.31	24.88	5.47	19.41
	27.08	9.21	17.87	22.4	5.15	17.25	20.13	9.66	10.47	20.04	5.81	14.23
	21.02	2.94	18.08	20.83	3.96	16.87	21.67	7.8	13.87	20.8	4.37	16.43
	21.01	6.51	14.5	23.43	2.81	20.62	24.12	7.48	16.64	21.1	8.02	13.08
Mean	22.61	5.72	16.88	22.99	6.39	16.6	23.34	11.28	12.06	23.04	7.35	15.69

Figure 6F (training session)

PRM (n = 11)	PV^{tdT}-AVT^{GFP}			PV^{tdT}-AVT^{hM4Di}			PV^{ChR2}-AVT^{GFP}			PV^{ChR2}-AVT^{hM4Di}			$Drd2^{GFP}$-AMT^{tdT}			$Drd2^{GFP}$-AMT^{hM4Di}			$Drd2^{ChR2}$-AMT^{tdT}			$Drd2^{ChR2}$-AMT^{hM4Di}		
	Total	A	A'	Total	A	A'	Total	A	A'	Total	A	A'	Total	A	A'	Total	A	A'	Total	A	A'	Total	A	A'
	31.46	16.99	14.47	29.16	16.04	13.12	37.9	21.98	15.92	34.91	19.55	15.36	36.07	16.59	19.48	24.21	8.96	15.25	38.2	15.28	22.92	31.85	15.29	16.56
	25.43	10.17	15.26	37.24	20.11	17.13	38.57	20.83	17.74	23.44	10.55	12.89	31.89	16.9	14.99	38.87	17.88	20.99	35.5	15.62	19.88	39.26	22.38	16.88
	38.02	19.01	19.01	32.37	16.51	15.86	43.58	24.84	18.74	39.31	15.33	23.98	42.98	25.79	17.19	36.93	21.79	15.14	31.5	16.38	15.12	33.25	14.3	18.95
	35.29	18.35	16.94	25.56	12.27	13.29	35.57	17.43	18.14	45.42	23.62	21.8	41.33	19.84	21.49	32.65	15.02	17.63	38.98	15.98	23	43.08	21.97	21.11
	30.93	13.61	17.32	34.05	19.07	14.98	40.02	19.21	20.81	26.13	12.28	13.85	38.22	17.2	21.02	22.53	9.69	12.84	30.21	12.99	17.22	34.62	14.54	20.08
	32.22	14.82	17.4	34.91	19.9	15.01	29.73	16.35	13.38	36.38	17.46	18.92	29.12	16.89	12.23	34.31	16.47	17.84	31.63	17.08	14.55	31.73	16.5	15.23
	38.07	16.75	21.32	33.4	11.69	21.71	24.57	11.3	13.27	34.36	16.15	18.21	22.74	8.87	13.87	40.59	17.86	22.73	25.64	12.05	13.59	35.31	18.36	16.95
	44.2	24.31	19.89	29.85	13.43	16.42	24.26	10.43	13.83	44.6	21.41	23.19	37.62	21.82	15.8	38.24	22.56	15.68	42.76	19.67	23.09	31.4	17.9	13.5
	42.18	21.51	20.67	45.57	24.61	20.96	36.54	23.75	12.79	30.91	17	13.91	29.53	15.06	14.47	27.1	10.03	17.07	33.68	15.83	17.85	23.21	9.05	14.16
	38	20.9	17.1	31.8	17.49	14.31	34.18	13.33	20.85	37.28	19.76	17.52	24.98	9.99	14.99	34.92	16.76	18.16	33.41	20.38	13.03	30.55	12.22	18.33
	34.33	13.73	20.6	35.53	17.41	18.12	35.36	14.5	20.86	39.5	20.54	18.96	33.59	14.11	19.48	40.65	24.39	16.26	37.1	21.52	15.58	38.9	15.95	22.95
Mean	35.47	17.29	18.18	33.59	17.14	16.45	34.57	17.63	16.94	35.65	17.6	18.05	33.46	16.64	16.82	33.73	16.49	17.24	34.42	16.62	17.8	33.92	16.22	17.7

ORM (n = 11)	PV^{tdT}-AVT^{GFP}			PV^{tdT}-AVT^{hM4Di}			PV^{ChR2}-AVT^{GFP}			PV^{ChR2}-AVT^{hM4Di}			$Drd2^{GFP}$-AMT^{tdT}			$Drd2^{GFP}$-AMT^{hM4Di}			$Drd2^{ChR2}$-AMT^{tdT}			$Drd2^{ChR2}$-AMT^{hM4Di}		
	Total	A	B	Total	A	B	Total	A	B	Total	A	B	Total	A	B	Total	A	B	Total	A	B	Total	A	B
	37.68	17.71	19.97	37.46	24.35	13.11	36.46	14.95	21.51	19.22	7.11	12.11	24.4	9.27	15.13	33.55	14.76	18.79	32.5	19.5	13	28.1	13.49	14.61
	21.98	8.79	13.19	25.44	9.92	15.52	42	28.56	13.44	27.18	11.69	15.49	22.67	8.16	14.51	25.17	12.08	13.09	30.94	10.21	20.73	41.87	19.26	22.61
	43.44	22.59	20.85	29.41	16.47	12.94	51.82	28.5	23.32	36.9	21.77	15.13	38.25	15.68	22.57	45.94	22.97	22.97	28.65	15.47	13.18	32.53	18.54	13.99
	35.49	23.07	12.42	29.68	12.76	16.92	23.75	10.69	13.06	49.4	29.64	19.76	49.21	30.51	18.7	35.94	16.53	19.41	23.18	8.11	15.07	39.04	20.3	18.74
	47.53	30.42	17.11	44.59	27.2	17.39	47.15	25.46	21.69	29.36	16.15	13.21	31	16.43	14.57	28.33	15.3	13.03	38.2	16.81	21.39	36.62	17.21	19.41
	23.44	9.61	13.83	27.75	11.93	15.82	40.08	24.05	16.03	29.92	15.56	14.36	33.88	16.94	16.94	41.36	26.47	14.89	47.49	30.87	16.62	36.19	22.8	13.39
	19.62	8.24	11.38	43.37	28.19	15.18	30.86	19.75	11.11	37.5	14.25	23.25	25.92	10.63	15.29	23.16	9.03	14.13	31.81	13.68	18.13	34	16.66	17.34
	57.24	33.2	24.04	33.17	13.27	19.9	24.18	9.43	14.75	28.89	10.98	17.91	27.22	13.61	13.61	38.32	20.31	18.01	42.27	23.67	18.6	26.87	14.24	12.63

	35.32	18.72	16.6	33.46	13.72	19.74	29.21	12.85	16.36	30.3	13.03	17.27	37.9	14.78	23.12	24.82	12.41	12.41	36.53	20.82	15.71	31.38	10.67	20.71
	29.51	12.1	17.41	45.67	21.92	23.75	38.47	21.93	16.54	27.32	14.48	12.84	42.83	25.27	17.56	36.17	17.36	18.81	25.08	9.53	15.55	31.55	11.04	20.51
	33.22	11.63	21.59	35.28	19.05	16.23	37.22	14.89	22.33	54.63	29.5	25.13	37.34	16.43	20.91	43.73	24.49	19.24	43.92	22.84	21.08	42.21	26.17	16.04
Mean	34.95	17.83	17.13	35.02	18.07	16.95	36.48	19.19	17.29	33.69	16.74	16.95	33.7	16.16	17.54	34.23	17.43	16.8	34.6	17.41	17.19	34.58	17.31	17.27

Figure S11G; Figure 6F (testing session)

PRM (n = 11)	PV^{tdT}-AVT^{GFP}			PV^{tdT}-AVT^{hM4Di}			PV^{ChR2}-AVT^{GFP}			PV^{ChR2}-AVT^{hM4Di}			Drd2^{GFP}-AMT^{tdT}			Drd2^{GFP}-AMT^{hM4Di}			Drd2^{ChR2}-AMT^{tdT}			Drd2^{ChR2}-AMT^{hM4Di}		
	Total	FL	NL	Total	FL	NL	Total	FL	NL	Total	FL	NL	Total	FL	NL	Total	FL	NL	Total	FL	NL	Total	FL	NL
	21.49	2.79	18.7	21.97	7.47	14.5	20.75	3.11	17.64	22.26	10.46	11.8	22.14	9.3	12.84	24.6	8.61	15.99	25.63	5.13	20.5	23.59	7.55	16.04
	20.58	4.94	15.64	20.03	6.21	13.82	21.92	7.67	14.25	27.94	18.72	9.22	24.2	9.92	14.28	23.05	10.37	12.68	23.12	9.25	13.87	20.23	9.71	10.52
	28.86	14.43	14.43	26.25	17.85	8.4	21.64	5.84	15.8	22.69	13.84	8.85	22.98	10.11	12.87	20.69	6	14.69	25.44	9.41	16.03	22.67	4.31	18.36
	21.18	4.45	16.73	22.6	11.75	10.85	20.33	3.66	16.67	21.81	16.14	5.67	22.59	2.94	19.65	20.61	4.33	16.28	22.34	5.36	16.98	23.71	7.59	16.12
	21.03	6.73	14.3	22.18	13.75	8.43	20.05	4.21	15.84	20.57	4.94	15.63	25.32	5.57	19.75	20.36	7.94	12.42	20.43	2.45	17.98	23.63	4.96	18.67
	20.5	4.51	15.99	21.68	12.14	9.54	20.24	3.64	16.6	20.6	6.8	13.8	23.71	7.59	16.12	25.89	11.13	14.76	20.89	3.13	17.76	26.59	4.79	21.8
	21.56	12.29	9.27	24.95	14.47	10.48	20.39	5.1	15.29	22.29	10.03	12.26	20.37	6.72	13.65	27.74	16.09	11.65	22.87	7.55	15.32	24.9	5.48	19.42
	22.09	4.64	17.45	27.19	14.14	13.05	22.02	8.37	13.65	22.09	12.59	9.5	21.64	6.06	15.58	20.07	5.62	14.45	25.66	4.36	21.3	21.63	4.33	17.3
	21.77	4.79	16.98	21.4	10.27	11.13	21.89	6.13	15.76	21.37	10.9	10.47	22.72	3.18	19.54	20.31	2.44	17.87	24.76	3.47	21.29	22	9.9	12.1
	20.33	3.46	16.87	21.93	9.87	12.06	21.97	7.03	14.94	21.25	10.2	11.05	21.59	3.89	17.7	24.88	5.97	18.91	22.18	7.54	14.64	21.92	8.99	12.93
	21.73	11.3	10.43	22.26	11.8	10.46	20.34	2.44	17.9	23.8	8.09	15.71	23.91	8.37	15.54	24.39	10.49	13.9	21.87	12.03	9.84	23.93	5.98	17.95
Mean	21.92	6.76	15.16	22.95	11.79	11.16	21.05	5.2	15.85	22.43	11.16	11.27	22.84	6.7	16.14	22.96	8.09	14.87	23.19	6.33	16.86	23.16	6.69	16.47
ORM (n = 11)	PV^{tdT}-AVT^{GFP}			PV^{tdT}-AVT^{hM4Di}			PV^{ChR2}-AVT^{GFP}			PV^{ChR2}-AVT^{hM4Di}			Drd2^{GFP}-AMT^{tdT}			Drd2^{GFP}-AMT^{hM4Di}			Drd2^{ChR2}-AMT^{tdT}			Drd2^{ChR2}-AMT^{hM4Di}		
	Total	FO	NO	Total	FO	NO	Total	FO	NO	Total	FO	NO	Total	FO	NO	Total	FO	NO	Total	FO	NO	Total	FO	NO
	24.87	7.71	17.16	20.58	7.2	13.38	26.01	8.58	17.43	21.27	5.53	15.74	28.89	12.42	16.47	20.4	6.73	13.67	23.47	3.29	20.18	24.11	10.85	13.26
	26.31	12.63	13.68	24.48	8.08	16.4	22.17	5.54	16.63	26.67	15.2	11.47	24.94	8.73	16.21	23.24	13.48	9.76	21.38	6.2	15.18	21.03	13.46	7.57
	20.12	4.43	15.69	32.16	13.51	18.65	22.83	3.88	18.95	25.76	7.73	18.03	23.54	7.53	16.01	29.55	23.05	6.5	20.16	8.47	11.69	22.21	7.33	14.88
	21.91	3.07	18.84	22.68	7.03	15.65	20.11	3.22	16.89	28.78	11.8	16.98	20.92	5.65	15.27	22	9.46	12.54	21.97	7.47	14.5	22.27	9.8	12.47
	20.58	4.32	16.26	25.28	7.08	18.2	25.3	9.87	15.43	21.68	3.25	18.43	21.79	5.23	16.56	21.69	11.93	9.76	25.12	4.52	20.6	26.27	10.77	15.5
	25.39	3.81	21.58	20.83	8.75	12.08	23.87	11.22	12.65	20.06	3.61	16.45	20.26	6.28	13.98	25.58	12.28	13.3	20.34	2.64	17.7	23.03	16.35	6.68
	28.11	12.37	15.74	23.38	7.95	15.43	27.85	8.91	18.94	23.16	7.64	15.52	21.65	7.36	14.29	22.89	10.3	12.59	24.25	10.43	13.82	23.82	14.77	9.05
	21.37	5.77	15.6	20.18	3.43	16.75	23.4	8.89	14.51	21.66	9.53	12.13	20.67	2.69	17.98	23.56	8.01	15.55	22.44	7.18	15.26	22.36	12.97	9.39
	22.39	9.63	12.76	23.3	8.39	14.91	23.98	10.07	13.91	22.79	9.8	12.99	20.77	3.95	16.82	24.74	13.11	11.63	20.08	5.22	14.86	20.6	10.71	9.89
	22.74	4.09	18.65	23.77	12.6	11.17	23.42	6.79	16.63	20.36	4.07	16.29	24.8	12.65	12.15	23.05	9.45	13.6	20.33	2.44	17.89	20.66	4.75	15.91
	21.95	7.46	14.49	22.15	5.76	16.39	23.08	8.31	14.77	26.35	6.06	20.29	25.9	8.03	17.87	24.8	14.63	10.17	23.01	6.21	16.8	22.79	8.66	14.13
Mean	23.25	6.84	16.4	23.52	8.16	15.36	23.82	7.75	16.07	23.51	7.66	15.85	23.1	7.32	15.78	23.77	12.04	11.73	22.05	5.82	16.23	22.65	10.95	11.7

Figure 7F, G (training session)

PRM (n = 11)	ON						OFF						ON						OFF					
	DS^{tdT}			DS^{ChR2}			DS^{tdT}			DS^{ChR2}			VS^{tdT}			VS^{ChR2}			VS^{tdT}			VS^{ChR2}		
	Total	A	A'	Total	A	A'	Total	A	A'	Total	A	A'	Total	A	A'	Total	A	A'	Total	A	A'	Total	A	A'
	28.62	10.59	18.03	32	13.12	18.88	37.6	21.43	16.17	30.07	13.23	16.84	32.98	13.52	19.46	28.81	13.25	15.56	26.09	12	14.09	33.33	19.33	14
	30.13	16.57	13.56	35.97	15.11	20.86	42.18	23.62	18.56	32.3	13.89	18.41	36	15.48	20.52	37.89	23.49	14.4	32.8	14.76	18.04	26.42	10.83	15.59
	34.96	19.23	15.73	30.58	12.23	18.35	40.88	24.53	16.35	36.47	24.07	12.4	39.09	21.5	17.59	30.05	17.73	12.32	35.65	22.46	13.19	35.53	17.41	18.12
	30.46	17.97	12.49	41.09	22.6	18.49	26.77	9.64	17.13	32.63	11.75	20.88	32.38	17.16	15.22	31.58	11.05	20.53	41.64	18.74	22.9	37.32	16.42	20.9
	30.89	14.52	16.37	31.71	15.22	16.49	28.72	10.34	18.38	39.03	25.76	13.27	34.47	18.27	16.2	24.28	11.41	12.87	35.08	16.84	18.24	24.74	9.65	15.09
	29.47	11.2	18.27	33.75	20.25	13.5	26.81	12.6	14.21	32.29	17.76	14.53	32.56	12.37	20.19	36.18	19.9	16.28	35.95	21.21	14.74	34.8	22.62	12.18
	38.91	18.29	20.62	24.7	11.61	13.09	36.3	21.78	14.52	29.64	10.67	18.97	35.97	23.38	12.59	39.94	18.77	21.17	32.54	19.85	12.69	33.77	13.17	20.6
	36.92	17.72	19.2	36.11	16.25	19.86	24.78	12.14	12.64	23.44	8.91	14.53	29.93	17.06	12.87	40.37	19.38	20.99	38	25.08	12.92	36.62	22.34	14.28
	30.29	15.45	14.84	30.21	17.52	12.69	36.57	17.19	19.38	35.9	17.59	18.31	28.88	16.75	12.13	30.36	13.66	16.7	30.57	14.37	16.2	27.77	11.11	16.66
	43.18	25.91	17.27	33.65	14.47	19.18	28.98	12.46	16.52	27.5	14.3	13.2	30.18	15.09	15.09	32.13	17.03	15.1	24.59	10.82	13.77	33.7	18.2	15.5
	27.3	10.65	16.65	31.91	18.19	13.72	31.25	10.31	20.94	40.61	20.71	19.9	31.52	15.13	16.39	31.23	12.18	19.05	30.39	10.03	20.36	40.04	22.02	18.02
Mean	32.83	16.19	16.64	32.88	16.05	16.83	32.8	16	16.8	32.72	16.24	16.48	33.08	16.88	16.2	32.99	16.17	16.82	33.02	16.92	16.1	33.1	16.65	16.45
ORM (n = 11)	ON						OFF						ON						OFF					
	DS^{tdT}			DS^{ChR2}			DS^{tdT}			DS^{ChR2}			VS^{tdT}			VS^{ChR2}			VS^{tdT}			VS^{ChR2}		
	Total	A	B	Total	A	B	Total	A	B	Total	A	B	Total	A	B	Total	A	B	Total	A	B	Total	A	B
	34.61	13.55	21.06	30.79	16.89	13.9	35.25	17.45	17.8	38.77	21.99	16.78	30.02	13.51	16.51	37.36	20.92	16.44	35.43	18.78	16.65	32.46	14.28	18.18
	28.35	16.94	11.41	36.79	22.53	14.26	40.61	20.53	20.08	32.85	12.72	20.13	39.38	20.87	18.51	25.36	14.71	10.65	32.15	16.72	15.43	35.06	23.84	11.22
	27.67	12.76	14.91	31.22	14.95	16.27	38.37	16.05	22.32	33.83	16.74	17.09	27.85	9.47	18.38	21.24	8.07	13.17	46.04	24.4	21.64	27.61	10.49	17.12
	32	18.13	13.87	33.91	13.73	20.18	39.49	22.26	17.23	42.92	22.78	20.14	35.65	13.19	22.46	40.14	20.07	20.07	30.1	17.46	12.64	34.95	20.97	13.98
	29.18	13.11	16.07	33.02	15.38	17.64	30.05	13.56	16.49	36.39	17.7	18.69	35.91	23.34	12.57	32.41	14.26	18.15	34.57	22.47	12.1	34.88	14.3	20.58
	36.58	21.16	15.42	31.19	12.27	18.92	25.09	11.86	13.23	34.31	16.81	17.5	31.36	20.07	11.29	33.79	14.53	19.26	32.06	11.86	20.2	28.29	9.9	18.39
	32.5	13.29	19.21	37.9	14.81	23.09	27.13	14.18	12.95	36.12	19.13	16.99	27.83	15.03	12.8	29.89	19.43	10.46	23.05	10.37	12.68	39.43	18.14	21.29
	34.42	16.61	17.81	29.2	13.89	15.31	30.66	16.28	14.38	34.32	18.6	15.72	33.75	17.55	16.2	36.05	20.55	15.5	38.55	18.12	20.43	30.72	16.59	14.13
	35.99	15.58	20.41	31.1	17.57	13.53	31.3	15.31	15.99	28.3	13.86	14.44	35.5	14.91	20.59	31.41	17.59	13.82	24.66	10.11	14.55	27.3	10.65	16.65
	32.52	14.73	17.79	36.86	19.78	17.08	27.11	13.42	13.69	30.25	14.5	15.75	42.32	21.16	21.16	26.3	9.47	16.83	29.19	10.51	18.68	39.85	21.52	18.33
	36.33	21.53	14.8	28.61	12.32	16.29	32.91	11.41	21.5	32.97	16.63	16.34	25.93	10.37	15.56	41.43	17.4	24.03	46.67	25.67	21	38.17	24.43	13.74
Mean	32.74	16.13	16.61	32.78	15.83	16.95	32.54	15.66	16.88	34.64	17.41	17.23	33.23	16.32	16.91	32.31	16.09	16.22	33.86	16.95	16.91	33.52	16.83	16.69

Figure 7F, G (testing session)

PRM (n = 11)	ON						OFF						ON						OFF					
	DS^{tdT}			DS^{ChR2}			DS^{tdT}			DS^{ChR2}			VS^{tdT}			VS^{ChR2}			VS^{tdT}			VS^{ChR2}		
	Total	FL	NL	Total	FL	NL	Total	FL	NL	Total	FL	NL	Total	FL	NL	Total	FL	NL	Total	FL	NL	Total	FL	NL
	22.63	11.09	11.54	24.96	10.73	14.23	23.27	12.1	11.17	23.21	16.71	6.5	22.33	6.7	15.63	21.61	9.29	12.32	22.85	8.68	14.17	20.63	7.84	12.79
	23.68	9	14.68	21.97	5.05	16.92	25.92	7.52	18.4	20.72	6.42	14.3	21.72	9.12	12.6	22.43	7.4	15.03	25.11	9.79	15.32	26.66	13.33	13.33
	23.73	7.12	16.61	20.16	4.23	15.93	22.76	11.61	11.15	21.34	10.67	10.67	24.12	11.58	12.54	25.4	13.97	11.43	28.91	12.14	16.77	24.84	11.18	13.66
	22.91	10.08	12.83	20.8	5.2	15.6	22.53	8	14.53	22.82	8.67	14.15	22.51	6.53	15.98	20.64	6.81	13.83	24.05	9.62	14.43	20.49	7.17	13.32
	21.34	5.55	15.79	23.1	4.62	18.48	20.4	5.1	15.3	20.75	5.19	15.56	22.45	6.06	16.39	22.93	12.38	10.55	24.79	13.14	11.65	21.46	8.37	13.09
	24.18	9.19	14.99	23.2	6.73	16.47	23.89	8.84	15.05	26.9	11.3	15.6	21.44	11.79	9.65	21.59	10.58	11.01	23.82	11.67	12.15	25.4	13.97	11.43
	26.51	8.48	18.03	29.23	7.31	21.92	21.29	10.75	10.54	24.84	9.44	15.4	23.63	10.4	13.23	23.49	9.63	13.86	20.04	6.61	13.43	25.28	6.07	19.21
	28.02	14.57	13.45	26	9.88	16.12	21.89	5.91	15.98	26.47	14.56	11.91	21.22	6.79	14.43	28	10.92	17.08	25.56	12.27	13.29	20.88	6.89	13.99
	26.22	11.01	15.21	20.67	6.41	14.26	21.93	12.06	9.87	20.91	3.14	17.77	20.35	5.09	15.26	27.66	12.17	15.49	27.41	11.24	16.17	21.32	7.89	13.43
	22.23	8.67	13.56	25.88	6.99	18.89	20.98	9.02	11.96	22.95	10.33	12.62	20.74	6.64	14.1	24.39	4.88	19.51	23.37	5.84	17.53	24.51	13.97	10.54
	20.85	6.88	13.97	20.27	3.65	16.62	23.36	7.71	15.65	20.05	7.62	12.43	26.58	15.15	11.43	21.88	6.13	15.75	21.37	10.47	10.9	23.92	9.09	14.83
Mean	23.85	9.24	14.61	23.29	6.44	16.86	22.57	8.97	13.6	22.81	9.46	13.36	22.46	8.71	13.75	23.64	9.47	14.17	24.3	10.13	14.16	23.22	9.62	13.6
ORM (n = 11)	ON						OFF						ON						OFF					
	DS^{tdT}			DS^{ChR2}			DS^{tdT}			DS^{ChR2}			VS^{tdT}			VS^{ChR2}			VS^{tdT}			VS^{ChR2}		
	Total	FO	NO	Total	FO	NO	Total	FO	NO	Total	FO	NO	Total	FO	NO	Total	FO	NO	Total	FO	NO	Total	FO	NO
	27.41	12.06	15.35	21.24	6.37	14.87	21.58	6.04	15.54	24.79	13.14	11.65	21.77	8.49	13.28	24.55	8.59	15.96	20.3	3.65	16.65	27.59	8.83	18.76
	23.88	11.94	11.94	20.66	9.71	10.95	22.19	10.43	11.76	25.44	13.99	11.45	21.06	4.63	16.43	22.97	5.28	17.69	22.75	8.42	14.33	26.97	18.07	8.9
	22.2	10.88	11.32	27.22	10.89	16.33	20.68	7.03	13.65	22.79	6.61	16.18	40.08	20.04	20.04	23.35	4.9	18.45	21.43	9	12.43	22.93	8.94	13.99
	21.75	8.7	13.05	22.02	7.49	14.53	26	12.22	13.78	23.84	10.73	13.11	22.68	7.94	14.74	23.62	8.74	14.88	25.59	15.61	9.98	21.37	9.83	11.54
	20.7	8.28	12.42	21.38	9.62	11.76	29.77	11.61	18.16	26.48	11.65	14.83	21.6	6.91	14.69	28.18	9.02	19.16	22.93	12.61	10.32	22.89	6.64	16.25
	24.09	12.77	11.32	21.36	5.77	15.59	20.72	8.29	12.43	21.49	7.95	13.54	20.77	6.02	14.75	20.59	5.56	15.03	25.12	10.55	14.57	20.33	8.13	12.2
	28.55	12.56	15.99	25.42	9.66	15.76	23.18	12.75	10.43	24.44	10.51	13.93	22.13	9.96	12.17	20.22	3.84	16.38	20.11	5.03	15.08	24.08	12.52	11.56
	25.95	9.6	16.35	23.33	9.8	13.53	23.82	7.86	15.96	20.77	5.19	15.58	23.76	9.03	14.73	22.89	8.24	14.65	30.98	10.53	20.45	28.53	14.55	13.98
	20.99	5.67	15.32	20.47	5.73	14.74	23.2	7.19	16.01	21.27	5.74	15.53	24.16	11.83	12.32	22.2	6.44	15.76	21.38	10.05	11.33	20.59	5.56	15.03
	24.52	7.11	17.41	20.54	8.01	12.53	25.88	12.94	12.94	23.47	7.04	16.43	23.5	13.63	9.87	23.42	7.26	16.16	23.51	5.88	17.63	20.91	6.48	14.43
	20.18	9.89	10.29	25.64	14.1	11.54	29.3	14.65	14.65	24.37	11.94	12.43	21.4	8.13	13.27	20.63	5.16	15.47	20.3	4.87	15.43	25.29	3.79	21.5
Mean	23.66	9.95	13.71	22.66	8.83	13.83	24.21	10.09	14.12	23.56	9.5	14.06	23.9	9.69	14.21	22.97	6.64	16.33	23.13	8.75	14.38	23.77	9.39	14.38

Table S6. Details about statistical analysis

Related to Figure 2F (unpaired t-test)

Unpaired t test (n=36 neurons per group)	
P value	<0.0001
P value summary	****
Significantly different (P < 0.05)?	Yes
One- or two-tailed P value?	Two-tailed
t, df	t=14.24, df=70

Related to Figure 2G (unpaired t-test)

Unpaired t test (n=17 neurons per group)	
P value	<0.0001
P value summary	****
Significantly different (P < 0.05)?	Yes
One- or two-tailed P value?	Two-tailed
t, df	t=15.95, df=32

Related to Figure 2H (unpaired t-test)

Unpaired t test (n=17 neurons per group)	
P value	<0.0001
P value summary	****
Significantly different (P < 0.05)?	Yes
One- or two-tailed P value?	Two-tailed
t, df	t=13.88, df=32

Related to Figure 3C (Ordinary one-way ANOVA)

ANOVA table (n = 18 neurons per group)	F (DFn, DFd)	P value
Group	F (2, 51) = 60.83	P<0.0001

Bonferroni's multiple comparisons test	t	Summary	Adjusted P Value
PV^{Kcnn4+} vs. PV^{Kcnn4-}	9.93	****	<0.0001
PV^{Kcnn4+} vs. PV^{Zfy2-}	0.8076	ns	0.8462

Related to Figure 3D (Ordinary one-way ANOVA)

ANOVA table (n = 18 neurons per group)	F (DFn, DFd)	P value
Group	F (2, 51) = 38.17	P<0.0001

Bonferroni's multiple comparisons test	t	Summary	Adjusted P Value
$Drd2^{Cacna1h+}$ vs. $Drd2^{Cacna1h-}$	7.542	****	<0.0001
$Drd2^{Cacna1h+}$ vs. $Drd2^{Zfy2-}$	0.05033	ns	>0.9999

Related to Figure 3E (unpaired t-test)

EPSC,Amplitude, PV

Unpaired t test (n = 18 neurons per group)	
P value	0.8531
P value summary	ns
Significantly different (P < 0.05)?	No
One- or two-tailed P value?	Two-tailed
t, df	t=0.1865, df=34

EPSC, Frequency, PV

Unpaired t test (n = 18 neurons per group)	
P value	0.7024
P value summary	ns
Significantly different (P < 0.05)?	No
One- or two-tailed P value?	Two-tailed
t, df	t=0.3853, df=34

EPSC, Amplitude, Drd2

Unpaired t test (n = 18 neurons per group)	
P value	0.8853
P value summary	ns
Significantly different (P < 0.05)?	No
One- or two-tailed P value?	Two-tailed
t, df	t=0.1454, df=34

EPSC, Frequency, Drd2

Unpaired t test (n = 18 neurons per group)	
P value	0.857
P value summary	ns
Significantly different (P < 0.05)?	No
One- or two-tailed P value?	Two-tailed
t, df	t=0.1815, df=34

Related to Figure 4A (Repeated Measures two-way ANOVA)

Latency, PV group

ANOVA table (n=11 mice per group)	F (DFn, DFd)	P value
Interaction	F (5, 100) = 0.9970	P=0.4236
Day	F (3.377, 67.54) = 177.8	P<0.0001
PV group	F (1, 20) = 0.7743	P=0.3893
Subject	F (20, 100) = 1.911	P=0.0193

Bonferroni's multiple comparisons test	t	Summary	Adjusted P Value
Day 1			
PV^{Kcnn4+} vs. PV^{Kcnn4-}	0.6646	ns	0.5149
Day2			
PV^{Kcnn4+} vs. PV^{Kcnn4-}	1.634	ns	0.1181

Day3			
PV^{Kcnn4+} vs. PV^{Kcnn4-}	0.6223	ns	0.5412
Day4			
PV^{Kcnn4+} vs. PV^{Kcnn4-}	0.746	ns	0.4644
Day5			
PV^{Kcnn4+} vs. PV^{Kcnn4-}	0.2205	ns	0.8283
Day6			
PV^{Kcnn4+} vs. PV^{Kcnn4-}	0.6966	ns	0.4949

Latency, Drd2 group

ANOVA table (n=11 mice per group)	F (DFn, DFd)	P value
Interaction	F (5, 100) = 1.686	P=0.1449
Day	F (2.985, 59.70) = 213.4	P<0.0001
Drd2 group	F (1, 20) = 0.06258	P=0.8050
Subject	F (20, 100) = 1.685	P=0.0483

Bonferroni's multiple comparisons test	t	Summary	Adjusted P Value
Day 1			
$Drd2^{Cacna1h+}$ vs. $Drd2^{Cacna1h-}$	2.029	ns	0.0562
Day2			
$Drd2^{Cacna1h+}$ vs. $Drd2^{Cacna1h-}$	0.3279	ns	0.7466
Day3			
$Drd2^{Cacna1h+}$ vs. $Drd2^{Cacna1h-}$	1.237	ns	0.2313
Day4			
$Drd2^{Cacna1h+}$ vs. $Drd2^{Cacna1h-}$	0.03992	ns	0.9687
Day5			
$Drd2^{Cacna1h+}$ vs. $Drd2^{Cacna1h-}$	1.707	ns	0.1036
Day6			
$Drd2^{Cacna1h+}$ vs. $Drd2^{Cacna1h-}$	1.931	ns	0.0709

Length, PV group

ANOVA table (n=11 mice per group)	F (DFn, DFd)	P value
Interaction	F (5, 100) = 0.9323	P=0.4635
Day	F (2.667, 53.33) = 183.0	P<0.0001
PV group	F (1, 20) = 0.7626	P=0.3929
Subject	F (20, 100) = 1.214	P=0.2591

Bonferroni's multiple comparisons test	t	Summary	Adjusted P Value
Day 1			
PV^{Kcnn4+} vs. PV^{Kcnn4-}	1.184	ns	0.2502
Day 2			
PV^{Kcnn4+} vs. PV^{Kcnn4-}	0.6927	ns	0.4969
Day 3			
PV^{Kcnn4+} vs. PV^{Kcnn4-}	0.8845	ns	0.3875
Day 4			
PV^{Kcnn4+} vs. PV^{Kcnn4-}	0.02827	ns	0.9777
Day 5			
PV^{Kcnn4+} vs. PV^{Kcnn4-}	1.03	ns	0.3157
Day 6			
PV^{Kcnn4+} vs. PV^{Kcnn4-}	0.9617	ns	0.3483

Length, Drd2 group

ANOVA table (n=11 mice per group)	F (DFn, DFd)	P value
Interaction	F (5, 100) = 0.6687	P=0.6481
Day	F (5, 100) = 146.7	P<0.0001
Drd2 group	F (1, 20) = 1.750	P=0.2008
Subject	F (20, 100) = 1.544	P=0.0833

Bonferroni's multiple comparisons test	t	Summary	Adjusted P Value
Day 1			
$Drd2^{Cacna1h+}$ vs. $Drd2^{Cacna1h-}$	0.6522	ns	0.5155
Day 2			
$Drd2^{Cacna1h+}$ vs. $Drd2^{Cacna1h-}$	1.37	ns	0.1731
Day 3			
$Drd2^{Cacna1h+}$ vs. $Drd2^{Cacna1h-}$	1.271	ns	0.2063
Day 4			
$Drd2^{Cacna1h+}$ vs. $Drd2^{Cacna1h-}$	0.6646	ns	0.5076
Day 5			
$Drd2^{Cacna1h+}$ vs. $Drd2^{Cacna1h-}$	1.081	ns	0.2819

Day 6			
Drd2$^{Cacna1h+}$ vs. Drd2$^{Cacna1h-}$	0.1206	ns	0.9042

Related to Figure 4B (unpaired t-test)

PV group

Unpaired t test (n=11 mice per group)	
P value	0.0005
P value summary	***
Significantly different (P < 0.05)?	Yes
One- or two-tailed P value?	Two-tailed
t, df	t=4.171, df=20

Drd2 group

Unpaired t test (n=11 mice per group)	
P value	0.8651
P value summary	ns
Significantly different (P < 0.05)?	No
One- or two-tailed P value?	Two-tailed
t, df	t=0.1721, df=20

Related to Figure 4D (unpaired t-test)

PV^{Kcnn4+}

Unpaired t test (n=11 mice per group)	
P value	<0.0001
P value summary	****
Significantly different (P < 0.05)?	Yes
One- or two-tailed P value?	Two-tailed
t, df	t=4.960, df=20

PV^{Kcnn4-}

Unpaired t test (n=11 mice per group)	
P value	0.8585
P value summary	ns
Significantly different (P < 0.05)?	No
One- or two-tailed P value?	Two-tailed
t, df	t=0.1806, df=20

Drd2$^{Cacna1h+}$

Unpaired t test (n=11 mice per group)	
P value	0.0001
P value summary	***
Significantly different (P < 0.05)?	Yes
One- or two-tailed P value?	Two-tailed
t, df	t=4.683, df=20

Drd2$^{Cacna1h-}$

Unpaired t test (n=11 mice per group)	
P value	<0.0001
P value summary	****
Significantly different (P < 0.05)?	Yes
One- or two-tailed P value?	Two-tailed
t, df	t=4.984, df=20

Related to Figure 4E (unpaired t-test)

PV group

Unpaired t test (n=11 mice per group)	
P value	0.0003
P value summary	***
Significantly different (P < 0.05)?	Yes
One- or two-tailed P value?	Two-tailed
t, df	t=4.390, df=20

Drd2 group

Unpaired t test (n=11 mice per group)	
P value	0.9861
P value summary	ns
Significantly different (P < 0.05)?	No
One- or two-tailed P value?	Two-tailed
t, df	t=0.01761, df=20

Related to Figure 4G (unpaired t-test)

PV^{Kcnn4+}

Unpaired t test (n=13 mice per group)	
P value	<0.0001
P value summary	****
Significantly different (P < 0.05)?	Yes
One- or two-tailed P value?	Two-tailed
t, df	t=6.568, df=24

PV^{Kcnn4-}

Unpaired t test (n=13 mice per group)	
P value	<0.0001
P value summary	****
Significantly different (P < 0.05)?	Yes
One- or two-tailed P value?	Two-tailed
t, df	t=6.686, df=24

Drd2$^{Cacna1h+}$

Unpaired t test (n=13 mice per group)	
P value	<0.0001
P value summary	****
Significantly different (P < 0.05)?	Yes
One- or two-tailed P value?	Two-tailed
t, df	t=6.130, df=24

Drd2$^{Cacna1h-}$

Unpaired t test (n=13 mice per group)	
P value	0.5772
P value summary	ns
Significantly different (P < 0.05)?	No
One- or two-tailed P value?	Two-tailed
t, df	t=0.5652, df=24

Related to Figure 4H (unpaired t-test)

PV group

Unpaired t test (n=13 mice per group)	
P value	0.9739
P value summary	ns
Significantly different (P < 0.05)?	No
One- or two-tailed P value?	Two-tailed
t, df	t=0.03312, df=24

Drd2 group

Unpaired t test (n=13 mice per group)	
P value	<0.0001
P value summary	****
Significantly different (P < 0.05)?	Yes
One- or two-tailed P value?	Two-tailed
t, df	t=5.123, df=24

Related to Figure 5C (Ordinary one-way ANOVA)

ANOVA table (n=5 mice per group)	F (DFn, DFd)	P value
Brain Region	F (3, 16) = 19.56	P<0.0001

Bonferroni's multiple comparisons test	t	Summary	Adjusted P Value
DS vs. VS	5.619	***	0.0002
DS vs. DTg	6.977	****	<0.0001

DS vs. VTg	5.82	***	0.0002
VS vs. DTg	1.359	ns	>0.9999
VS vs. VTg	0.2016	ns	>0.9999
DTg vs. VTg	1.157	ns	>0.9999

Related to Figure 5F (Ordinary one-way ANOVA)

ANOVA table (n=5 mice per group)	F (DFn, DFd)	P value
Treatment	F (3, 16) = 31.72	P<0.0001

Bonferroni's multiple comparisons test	t	Summary	Adjusted P Value
DS vs. VS	7.678	****	<0.0001
DS vs. DTg	0.8786	ns	>0.9999
DS vs. VTg	0.1825	ns	>0.9999
VS vs. DTg	8.557	****	<0.0001
VS vs. VTg	7.496	****	<0.0001
DTg vs. VTg	1.061	ns	>0.9999

Related to Figure 5G (Ordinary one-way ANOVA)

ANOVA table (n=15 neurons per group)	F (DFn, DFd)	P value
Treatment	F (3, 56) = 128.5	P<0.0001

Bonferroni's multiple comparisons test	t	Summary	Adjusted P Value
CNQX vs. TTX	14.82	****	<0.0001
CNQX vs. TTX+4-AP	1.839	ns	0.4277
CNQX vs. TTX+4-AP+AP-5	14.65	****	<0.0001
TTX vs. TTX+4-AP	12.99	****	<0.0001
TTX vs. TTX+4-AP+AP-5	0.1725	ns	>0.9999
TTX+4-AP vs. TTX+4-AP+AP-5	12.81	****	<0.0001

Related to Figure 5H (Ordinary one-way ANOVA)

ANOVA table (n=15 neurons per group)	F (DFn, DFd)	P value
Treatment	F (3, 56) = 185.7	P<0.0001

Bonferroni's multiple comparisons test	t	Summary	Adjusted P Value
CNQX vs. TTX	17.72	****	<0.0001
CNQX vs. TTX+4-AP	2.263	ns	0.165
CNQX vs. TTX+4-AP+AP-5	17.78	****	<0.0001
TTX vs. TTX+4-AP	15.45	****	<0.0001
TTX vs. TTX+4-AP+AP-5	0.05989	ns	>0.9999
TTX+4-AP vs. TTX+4-AP+AP-5	15.51	****	<0.0001

Related to Figure 6B (Ordinary two-way ANOVA)

PRM, DS group

ANOVA table (n=11 mice per group)	F (DFn, DFd)	P value
Interaction	F (1, 40) = 7.383	P=0.0097
GFP or hM4Di in DS	F (1, 40) = 6.268	P=0.0165
CNO or Saline	F (1, 40) = 6.268	P=0.0165

Tukey's multiple comparisons test	q	Summary	Adjusted P Value
DS^{GFP}:CNO vs. DS^{GFP}:Saline	0.2137	ns	0.9987
DS^{GFP}:CNO vs. DS^{hM4Di}:CNO	5.221	**	0.0036
DS^{GFP}:CNO vs. DS^{hM4Di}:Saline	0	ns	>0.9999
DS^{GFP}:Saline vs. DS^{hM4Di}:CNO	5.007	**	0.0055
DS^{GFP}:Saline vs. DS^{hM4Di}:Saline	0.2137	ns	0.9987
DS^{hM4Di}:CNO vs. DS^{hM4Di}:Saline	5.221	**	0.0036

ORM, DS group

ANOVA table (n=11 mice per group)	F (DFn, DFd)	P value
Interaction	F (1, 40) = 0.3990	P=0.5312
GFP or hM4Di in DS	F (1, 40) = 0.02262	P=0.8812
CNO or Saline	F (1, 40) = 1.376	P=0.2477

Tukey's multiple comparisons test	q	Summary	Adjusted P Value
DS^{GFP}:CNO vs. DS^{GFP}:Saline	0.5414	ns	0.9806
DS^{GFP}:CNO vs. DS^{hM4Di}:CNO	0.782	ns	0.9452
DS^{GFP}:CNO vs. DS^{hM4Di}:Saline	1.023	ns	0.8873
DS^{GFP}:Saline vs. DS^{hM4Di}:CNO	1.323	ns	0.7858
DS^{GFP}:Saline vs. DS^{hM4Di}:Saline	0.4813	ns	0.9862
DS^{hM4Di}:CNO vs. DS^{hM4Di}:Saline	1.805	ns	0.5832

PRM, VS group

ANOVA table (n=11 mice per group)	F (DFn, DFd)	P value
Interaction	F (1, 40) = 0.9568	P=0.3339
tdT or hM4Di in VS	F (1, 40) = 0.006026	P=0.9385
CNO or Saline	F (1, 40) = 2.459	P=0.1247

Tukey's multiple comparisons test	q	Summary	Adjusted P Value
VS^{tdT}:CNO vs. VS^{tdT}:Saline	0.59	ns	0.9752
VS^{tdT}:CNO vs. VS^{hM4Di}:CNO	1.056	ns	0.8776
VS^{tdT}:CNO vs. VS^{hM4Di}:Saline	1.49	ns	0.719
VS^{tdT}:Saline vs. VS^{hM4Di}:CNO	1.646	ns	0.6528
VS^{tdT}:Saline vs. VS^{hM4Di}:Saline	0.9005	ns	0.9195
VS^{hM4Di}:CNO vs. VS^{hM4Di}:Saline	2.546	ns	0.2881

ORM, VS group

ANOVA table (n=11 mice per group)	F (DFn, DFd)	P value
Interaction	F (1, 40) = 11.30	P=0.0017
tdT or hM4Di in VS	F (1, 40) = 9.861	P=0.0032
CNO or Saline	F (1, 40) = 6.121	P=0.0177

Tukey's multiple comparisons test	q	Summary	Adjusted P Value
VS^{tdT}:CNO vs. VS^{tdT}:Saline	0.8881	ns	0.9224
VS^{tdT}:CNO vs. VS^{hM4Di}:CNO	6.502	***	0.0002
VS^{tdT}:CNO vs. VS^{hM4Di}:Saline	0.6661	ns	0.965
VS^{tdT}:Saline vs. VS^{hM4Di}:CNO	5.614	**	0.0016
VS^{tdT}:Saline vs. VS^{hM4Di}:Saline	0.222	ns	0.9986
VS^{hM4Di}:CNO vs. VS^{hM4Di}:Saline	5.836	**	0.001

Related to Figure 6D (unpaired t-test)

PRM, DS-PV group

Unpaired t test (n=11 mice per group)	
P value	0.0026
P value summary	**
Significantly different (P < 0.05)?	Yes
One- or two-tailed P value?	Two-tailed
t, df	t=3.432, df=20

ORM, DS-PV group

Unpaired t test (n=11 mice per group)	
P value	0.5437
P value summary	ns
Significantly different (P < 0.05)?	No
One- or two-tailed P value?	Two-tailed
t, df	t=0.6177, df=20

PRM, VS-Drd2 group

Unpaired t test (n=11 mice per group)	
P value	0.8354
P value summary	ns
Significantly different (P < 0.05)?	No
One- or two-tailed P value?	Two-tailed
t, df	t=0.2106, df=20

ORM, VS-Drd2 group

Unpaired t test (n=11 mice per group)	
P value	0.002
P value summary	**
Significantly different (P < 0.05)?	Yes
One- or two-tailed P value?	Two-tailed
t, df	t=3.556, df=20

Related to Figure 6F (Ordinary two-way ANOVA)

PRM, PV-AVT group

ANOVA table (n=11 mice per group)	F (DFn, DFd)	P value
Interaction	F (1, 40) = 0.2657	P=0.6090
tdT or ChR2 in PV	F (1, 40) = 0.8785	P=0.3542
GFP or hM4Di in AVT	F (1, 40) = 34.32	P<0.0001

Tukey's multiple comparisons test	q	Summary	Adjusted P Value
PV^{tdT}:AVT^{GFP} vs. PV^{tdT}:AVT^{hM4Di}	5.342	**	0.0028
PV^{tdT}:AVT^{GFP} vs. PV^{ChR2}:AVT^{GFP}	1.453	ns	0.7346
PV^{tdT}:AVT^{GFP} vs. PV^{ChR2}:AVT^{hM4Di}	4.921	**	0.0065
PV^{tdT}:AVT^{hM4Di} vs. PV^{ChR2}:AVT^{GFP}	6.795	***	0.0001
PV^{tdT}:AVT^{hM4Di} vs. PV^{ChR2}:AVT^{hM4Di}	0.4218	ns	0.9906
PV^{ChR2}:AVT^{GFP} vs. PV^{ChR2}:AVT^{hM4Di}	6.374	***	0.0003

ORM, PV-AVT group

ANOVA table (n=11 mice per group)	F (DFn, DFd)	P value
Interaction	F (1, 40) = 0.7375	P=0.3956
tdT or ChR2 in PV	F (1, 40) = 0.01801	P=0.8939
GFP or hM4Di in AVT	F (1, 40) = 0.5646	P=0.4568

Tukey's multiple comparisons test	q	Summary	Adjusted P Value
PV^{tdT}:AVT^{GFP} vs. PV^{tdT}:AVT^{hM4Di}	1.61	ns	0.6682
PV^{tdT}:AVT^{GFP} vs. PV^{ChR2}:AVT^{GFP}	0.993	ns	0.8956

PV^{tdT}:AVT^{GFP} vs. PV^{ChR2}:AVT^{hM4Di}	0.8856	ns	0.923
PV^{tdT}:AVT^{hM4Di} vs. PV^{ChR2}:AVT^{GFP}	0.6172	ns	0.9718
PV^{tdT}:AVT^{hM4Di} vs. PV^{ChR2}:AVT^{hM4Di}	0.7246	ns	0.9557
PV^{ChR2}:AVT^{GFP} vs. PV^{ChR2}:AVT^{hM4Di}	0.1073	ns	0.9998

PRM, Drd2-AMT

ANOVA table (n=11 mice per group)	F (DFn, DFd)	P value
Interaction	F (1, 40) = 0.1653	P=0.6865
GFP or ChR2 in Drd2	F (1, 40) = 0.8538	P=0.3610
tdT or hM4Di in AMT	F (1, 40) = 0.8999	P=0.3485

Tukey's multiple comparisons test	q	Summary	Adjusted P Value
$Drd2^{GFP}$:AMT^{tdT} vs. $Drd2^{GFP}$:AMT^{hM4Di}	1.355	ns	0.7736
$Drd2^{GFP}$:AMT^{tdT} vs. $Drd2^{ChR2}$:AMT^{tdT}	0.5174	ns	0.983
$Drd2^{GFP}$:AMT^{tdT} vs. $Drd2^{ChR2}$:AMT^{hM4Di}	0.02464	ns	>0.9999
$Drd2^{GFP}$:AMT^{hM4Di} vs. $Drd2^{ChR2}$:AMT^{tdT}	1.873	ns	0.5534
$Drd2^{GFP}$:AMT^{hM4Di} vs. $Drd2^{ChR2}$:AMT^{hM4Di}	1.331	ns	0.7831
$Drd2^{ChR2}$:AMT^{tdT} vs. $Drd2^{ChR2}$:AMT^{hM4Di}	0.5421	ns	0.9806

ORM, Drd2-AMT

ANOVA table (n=11 mice per group)	F (DFn, DFd)	P value
Interaction	F (1, 40) = 0.1718	P=0.6807
GFP or ChR2 in Drd2	F (1, 40) = 0.6475	P=0.4258
tdT or hM4Di in AMT	F (1, 40) = 29.83	P<0.0001

Tukey's multiple comparisons test	q	Summary	Adjusted P Value
$Drd2^{GFP}$:AMT^{tdT} vs. $Drd2^{GFP}$:AMT^{hM4Di}	5.048	**	0.005
$Drd2^{GFP}$:AMT^{tdT} vs. $Drd2^{ChR2}$:AMT^{tdT}	1.219	ns	0.8242
$Drd2^{GFP}$:AMT^{tdT} vs. $Drd2^{ChR2}$:AMT^{hM4Di}	4.657	*	0.0107
$Drd2^{GFP}$:AMT^{hM4Di} vs. $Drd2^{ChR2}$:AMT^{tdT}	6.267	***	0.0004
$Drd2^{GFP}$:AMT^{hM4Di} vs. $Drd2^{ChR2}$:AMT^{hM4Di}	0.3901	ns	0.9925
$Drd2^{ChR2}$:AMT^{tdT} vs. $Drd2^{ChR2}$:AMT^{hM4Di}	5.877	***	0.0009

Related to Figure 7B (Ordinary one-way ANOVA)
Peak

ANOVA table (n=11 mice per group)	F (DFn, DFd)	P value
Treatment	F (3, 40) = 158.5	P<0.0001

Bonferroni's multiple comparisons test	t	Summary	Adjusted P Value
NL vs. FL	18.54	****	<0.0001
NL vs. NO	15.95	****	<0.0001
NL vs. FO	18.44	****	<0.0001
FL vs. NO	2.587	ns	0.0806
FL vs. FO	0.09706	ns	>0.9999
NO vs. FO	2.489	ns	0.1023

AUC

ANOVA table (n=11 mice per group)	F (DFn, DFd)	P value
Treatment	F (3, 40) = 99.99	P<0.0001

Bonferroni's multiple comparisons test	t	Summary	Adjusted P Value
NL vs. FL	14.47	****	<0.0001
NL vs. NO	13.61	****	<0.0001
NL vs. FO	14.29	****	<0.0001
FL vs. NO	0.8577	ns	>0.9999
FL vs. FO	0.1757	ns	>0.9999
NO vs. FO	0.6821	ns	>0.9999

Related to Figure 7C (Ordinary one-way ANOVA)
Peak

ANOVA table (n=11 mice per group)	F (DFn, DFd)	P value
Treatment	F (3, 40) = 136.0	P<0.0001

Bonferroni's multiple comparisons test	t	Summary	Adjusted P Value
NP vs. FP	1.178	ns	>0.9999
NP vs. NO	15.74	****	<0.0001
NP vs. FO	0.9903	ns	>0.9999
FP vs. NO	16.92	****	<0.0001
FP vs. FO	0.1879	ns	>0.9999
NO vs. FO	16.73	****	<0.0001

AUC

ANOVA table (n=11 mice per group)	F (DFn, DFd)	P value
Treatment	F (3, 40) = 188.4	P<0.0001

Bonferroni's multiple comparisons test	t	Summary	Adjusted P Value
NP vs. FP	1.236	ns	>0.9999
NP vs. NO	18.66	****	<0.0001
NP vs. FO	0.921	ns	>0.9999
FP vs. NO	19.9	****	<0.0001
FP vs. FO	0.315	ns	>0.9999
NO vs. FO	19.58	****	<0.0001

Related to Figure 7D (unpaired t-test)

Peak DS group

Unpaired t test (n=9 mice per group)	
P value	<0.0001
P value summary	****
Significantly different (P < 0.05)?	Yes
One- or two-tailed P value?	Two-tailed
t, df	t=26.99, df=16

Peak VS group

Unpaired t test (n=9 mice per group)	
P value	<0.0001
P value summary	****
Significantly different (P < 0.05)?	Yes
One- or two-tailed P value?	Two-tailed
t, df	t=15.61, df=16

AUC DS group

Unpaired t test (n=9 mice per group)	
P value	<0.0001
P value summary	****
Significantly different (P < 0.05)?	Yes
One- or two-tailed P value?	Two-tailed
t, df	t=25.38, df=16

AUC VS group

Unpaired t test (n=9 mice per group)	
P value	<0.0001
P value summary	****
Significantly different (P < 0.05)?	Yes
One- or two-tailed P value?	Two-tailed
t, df	t=15.85, df=16

Related to Figure 7F (Ordinary two-way ANOVA)

PRM, DS group

ANOVA table (n=11 mice per group)	F (DFn, DFd)	P value
Interaction	F (1, 40) = 3.455	P=0.0704
light ON or OFF	F (1, 40) = 5.176	P=0.0283
tdT or ChR2 in DS	F (1, 40) = 2.414	P=0.1281

Tukey's multiple comparisons test	q	Summary	Adjusted P Value
ON:DS^{tdT} vs. ON:DS^{ChR2}	3.413	ns	0.0908
ON:DS^{tdT} vs. OFF:DS^{tdT}	0.4162	ns	0.991
ON:DS^{tdT} vs. OFF:DS^{ChR2}	0.7213	ns	0.9562
ON:DS^{ChR2} vs. OFF:DS^{tdT}	3.829	*	0.047
ON:DS^{ChR2} vs. OFF:DS^{ChR2}	4.134	*	0.0279
OFF:DS^{tdT} vs. OFF:DS^{ChR2}	0.3052	ns	0.9964

ORM, DS group

ANOVA table (n=11 mice per group)	F (DFn, DFd)	P value
Interaction	F (1, 40) = 0.1064	P=0.7460
light ON or OFF	F (1, 40) = 0.004254	P=0.9483
tdT or ChR2 in DS	F (1, 40) = 0.7754	P=0.3838

Tukey's multiple comparisons test	q	Summary	Adjusted P Value
ON:DS^{tdT} vs. ON:DS^{ChR2}	1.207	ns	0.8286
ON:DS^{tdT} vs. OFF:DS^{tdT}	0.2609	ns	0.9977
ON:DS^{tdT} vs. OFF:DS^{ChR2}	0.8153	ns	0.9385
ON:DS^{ChR2} vs. OFF:DS^{tdT}	0.9458	ns	0.9082
ON:DS^{ChR2} vs. OFF:DS^{ChR2}	0.3914	ns	0.9925
OFF:DS^{tdT} vs. OFF:DS^{ChR2}	0.5544	ns	0.9793

PRM, VS group

ANOVA table (n=11 mice per group)	F (DFn, DFd)	P value
Interaction	F (1, 40) = 0.1275	P=0.7229
light ON or OFF	F (1, 40) = 0.5099	P=0.4793
tdT or ChR2 in VS	F (1, 40) = 0.03187	P=0.8592

Tukey's multiple comparisons test	q	Summary	Adjusted P Value
ON:VS^{tdT} vs. ON:VS^{ChR2}	0.5356	ns	0.9812
ON:VS^{tdT} vs. OFF:VS^{tdT}	1.071	ns	0.873
ON:VS^{tdT} vs. OFF:VS^{ChR2}	0.8926	ns	0.9214
ON:VS^{ChR2} vs. OFF:VS^{tdT}	0.5356	ns	0.9812

ON:VS^{ChR2} vs. OFF:VS^{ChR2}	0.357	ns	0.9943
OFF:VS^{tdT} vs. OFF:VS^{ChR2}	0.1785	ns	0.9993

ORM, VS group

ANOVA table (n=11 mice per group)	F (DFn, DFd)	P value
Interaction	F (1, 40) = 3.248	P=0.0790
light ON or OFF	F (1, 40) = 1.332	P=0.2554
tdT or ChR2 in VS	F (1, 40) = 1.715	P=0.1978

Tukey's multiple comparisons test	q	Summary	Adjusted P Value
ON:VS^{tdT} vs. ON:VS^{ChR2}	3.112	ns	0.1406
ON:VS^{tdT} vs. OFF:VS^{tdT}	0.6483	ns	0.9676
ON:VS^{tdT} vs. OFF:VS^{ChR2}	0.1556	ns	0.9995
ON:VS^{ChR2} vs. OFF:VS^{tdT}	2.464	ns	0.3161
ON:VS^{ChR2} vs. OFF:VS^{ChR2}	2.956	ns	0.1737
OFF:VS^{tdT} vs. OFF:VS^{ChR2}	0.4927	ns	0.9853

Related to Figure S2B (unpaired t-test)

PV group

Unpaired t test (n = 3 biological replicates)	
P value	0.0035
P value summary	**
Significantly different (P < 0.05)?	Yes
One- or two-tailed P value?	Two-tailed
t, df	t=6.168, df=4

Drd2 group

Unpaired t test (n = 3 biological replicates)	
P value	0.0009
P value summary	***
Significantly different (P < 0.05)?	Yes
One- or two-tailed P value?	Two-tailed
t, df	t=8.795, df=4

Related to Figure S3A (unpaired t-test）

AP

Unpaired t test (n=21 neurons per group)	
P value	0.0023
P value summary	**
Significantly different (P < 0.05)?	Yes
One- or two-tailed P value?	Two-tailed
t, df	t=3.263, df=40

HW

Unpaired t test (n=21 neurons per group)	
P value	<0.0001
P value summary	****
Significantly different (P < 0.05)?	Yes
One- or two-tailed P value?	Two-tailed
t, df	t=5.200, df=40

Threshold

Unpaired t test (n=21 neurons per group)	
P value	0.0598
P value summary	ns
Significantly different (P < 0.05)?	No
One- or two-tailed P value?	Two-tailed
t, df	t=1.938, df=40

Related to Figure S3B (unpaired t-test）

Unpaired t test (n=21 neurons per group)	
P value	0.7648
P value summary	ns
Significantly different (P < 0.05)?	No
One- or two-tailed P value?	Two-tailed
t, df	t=0.3012, df=40

Related to Figure S3C (Ordinary two-way ANOVA)

ANOVA table (n=27 neurons per group)	F (DFn, DFd)	P value
Interaction	F (6, 364) = 10.18	P<0.0001
injected currents	F (6, 364) = 85.89	P<0.0001
neural type	F (1, 364) = 274.3	P<0.0001

Bonferroni's multiple comparisons test	t	Summary	Adjusted P Value
0 pA			
Drd2 vs. PV	0	ns	>0.9999
50 pA			
Drd2 vs. PV	3.844	***	0.0001
100 pA			
Drd2 vs. PV	7.711	****	<0.0001
150 pA			
Drd2 vs. PV	8.451	****	<0.0001
200 pA			
Drd2 vs. PV	8.495	****	<0.0001
250 pA			
Drd2 vs. PV	8.021	****	<0.0001

300 pA			
Drd2 vs. PV	7.293	****	<0.0001

Related to Figure S4 (Ordinary one-way ANOVA)

Kcnn4

ANOVA table (n = 5 mice per group)	F (DFn, DFd)	P value
Treatment	F (2, 12) = 33.90	P<0.0001

Bonferroni's multiple comparisons test	t	Summary	Adjusted P Value
PV^{Kcnn4+} vs. PV^{Kcnn4-}	7.215	****	<0.0001
PV^{Kcnn4+} vs. PV^{Zfy2-}	0.1713	ns	>0.9999

Cacna1h

ANOVA table (n = 5 mice per group)	F (DFn, DFd)	P value
Treatment	F (2, 12) = 39.22	P<0.0001

Bonferroni's multiple comparisons test	t	Summary	Adjusted P Value
$Drd2^{Cacna1h+}$ vs. $Drd2^{Cacna1h-}$	8.157	****	<0.0001
$Drd2^{Cacna1h+}$ vs. $Drd2^{Zfy2-}$	1.09	ns	0.594

Related to Figure S5A (unpaired t-test)

Drinking

PV group

Unpaired t test (n=11 mice per group)	
P value	0.8691
P value summary	ns
Significantly different (P < 0.05)?	No
One- or two-tailed P value?	Two-tailed
t, df	t=0.1670, df=20

Drd2 group

Unpaired t test (n=11 mice per group)	
P value	0.7299
P value summary	ns
Significantly different (P < 0.05)?	No
One- or two-tailed P value?	Two-tailed
t, df	t=0.3501, df=20

Eating

PV group

Unpaired t test (n=11 mice per group)	
P value	0.8534
P value summary	ns
Significantly different (P < 0.05)?	No
One- or two-tailed P value?	Two-tailed
t, df	t=0.1871, df=20

Drd2 group

Unpaired t test (n=11 mice per group)	
P value	0.5943
P value summary	ns
Significantly different (P < 0.05)?	No
One- or two-tailed P value?	Two-tailed
t, df	t=0.5412, df=20

Static

PV group

Unpaired t test (n=11 mice per group)	
P value	0.9427
P value summary	ns
Significantly different (P < 0.05)?	No
One- or two-tailed P value?	Two-tailed
t, df	t=0.07275, df=20

Drd2 group

Unpaired t test (n=11 mice per group)	
P value	0.8207
P value summary	ns
Significantly different (P < 0.05)?	No
One- or two-tailed P value?	Two-tailed
t, df	t=0.2296, df=20

Digging

PV group

Unpaired t test (n=11 mice per group)	
P value	0.5592
P value summary	ns
Significantly different (P < 0.05)?	No
One- or two-tailed P value?	Two-tailed
t, df	t=0.5939, df=20

Drd2 group

Unpaired t test (n=11 mice per group)	
P value	0.8369
P value summary	ns
Significantly different (P < 0.05)?	No
One- or two-tailed P value?	Two-tailed
t, df	t=0.2086, df=20

Groming

PV group

Unpaired t test (n=11 mice per group)	
P value	0.8767
P value summary	ns
Significantly different (P < 0.05)?	No
One- or two-tailed P value?	Two-tailed
t, df	t=0.1572, df=20

Drd2 group

Unpaired t test (n=11 mice per group)	
P value	0.5668
P value summary	ns
Significantly different (P < 0.05)?	No
One- or two-tailed P value?	Two-tailed
t, df	t=0.5824, df=20

Sniffing

PV group

Unpaired t test (n=11 mice per group)	
P value	0.2051
P value summary	ns
Significantly different (P < 0.05)?	No

Drd2 group

Unpaired t test (n=11 mice per group)	
P value	0.9452
P value summary	ns
Significantly different (P < 0.05)?	No

One- or two-tailed P value?	Two-tailed
t, df	t=1.310, df=20

One- or two-tailed P value?	Two-tailed
t, df	t=0.06965, df=20

Jumping

PV group

Unpaired t test (n=11 mice per group)	
P value	0.7253
P value summary	ns
Significantly different (P < 0.05)?	No
One- or two-tailed P value?	Two-tailed
t, df	t=0.3564, df=20

Drd2 group

Unpaired t test (n=11 mice per group)	
P value	0.8145
P value summary	ns
Significantly different (P < 0.05)?	No
One- or two-tailed P value?	Two-tailed
t, df	t=0.2377, df=20

Standing

PV group

Unpaired t test (n=11 mice per group)	
P value	0.88
P value summary	ns
Significantly different (P < 0.05)?	No
One- or two-tailed P value?	Two-tailed
t, df	t=0.1529, df=20

Drd2 group

Unpaired t test (n=11 mice per group)	
P value	0.9339
P value summary	ns
Significantly different (P < 0.05)?	No
One- or two-tailed P value?	Two-tailed
t, df	t=0.08397, df=20

Moving

PV group

Unpaired t test (n=11 mice per group)	
P value	0.5717
P value summary	ns
Significantly different (P < 0.05)?	No
One- or two-tailed P value?	Two-tailed
t, df	t=0.5750, df=20

Drd2 group

Unpaired t test (n=11 mice per group)	
P value	0.7353
P value summary	ns
Significantly different (P < 0.05)?	No
One- or two-tailed P value?	Two-tailed
t, df	t=0.3428, df=20

Related to Figure S5B (unpaired t-test)

NC, PV group

Unpaired t test (n=11 mice per group)	
P value	0.9578
P value summary	ns
Significantly different (P < 0.05)?	No
One- or two-tailed P value?	Two-tailed
t, df	t=0.05360, df=20

NC, Drd2 group

Unpaired t test (n=11 mice per group)	
P value	0.5514
P value summary	ns
Significantly different (P < 0.05)?	No
One- or two-tailed P value?	Two-tailed
t, df	t=0.6059, df=20

TC, PV group

Unpaired t test (n=11 mice per group)	
P value	0.6775
P value summary	ns
Significantly different (P < 0.05)?	No
One- or two-tailed P value?	Two-tailed
t, df	t=0.4220, df=20

TC, Drd2 group

Unpaired t test (n=11 mice per group)	
P value	0.7665
P value summary	ns
Significantly different (P < 0.05)?	No
One- or two-tailed P value?	Two-tailed
t, df	t=0.3011, df=20

Related to Figure S5D (unpaired t-test)

NO, PV group

Unpaired t test (n=11 mice per group)	
P value	0.5175
P value summary	ns
Significantly different (P < 0.05)?	No
One- or two-tailed P value?	Two-tailed
t, df	t=0.6588, df=20

NO, Drd2 group

Unpaired t test (n=11 mice per group)	
P value	0.5385
P value summary	ns
Significantly different (P < 0.05)?	No
One- or two-tailed P value?	Two-tailed
t, df	t=0.6259, df=20

TO, PV group

Unpaired t test (n=11 mice per group)	
P value	0.4734
P value summary	ns
Significantly different (P < 0.05)?	No
One- or two-tailed P value?	Two-tailed
t, df	t=0.7307, df=20

TO, Drd2 group

Unpaired t test (n=11 mice per group)	
P value	0.7333
P value summary	ns
Significantly different (P < 0.05)?	No
One- or two-tailed P value?	Two-tailed
t, df	t=0.3455, df=20

Related to Figure S5E (Ordinary two-way ANOVA)

Quadrant, Length, PV group

ANOVA table (n=11 mice per group)	F (DFn, DFd)	P value
Interaction	F (3, 80) = 11.04	P<0.0001
Quadrant	F (3, 80) = 46.13	P<0.0001
PV group	F (1, 80) = 0.6944	P=0.4071

Bonferroni's multiple comparisons test	t	Summary	Adjusted P Value

Q1			
PV^{Kcnn4+} vs. PV^{Kcnn4-}	2.113	*	0.0377
Q2			
PV^{Kcnn4+} vs. PV^{Kcnn4-}	4.563	****	<0.0001
Q3			
PV^{Kcnn4+} vs. PV^{Kcnn4-}	2.233	*	0.0283
Q4			
PV^{Kcnn4+} vs. PV^{Kcnn4-}	1.883	ns	0.0633

Quadrant, Length, Drd2 group

ANOVA table (n=11 mice per group)	F (DFn, DFd)	P value
Interaction	F (3, 80) = 0.1475	P=0.9310
Quadrant	F (3, 80) = 56.13	P<0.0001
Drd2 group	F (1, 80) = 0.7486	P=0.3895

Bonferroni's multiple comparisons test	t	Summary	Adjusted P Value
Q1			
Drd2$^{Cacna1h+}$ vs. Drd2$^{Cacna1h-}$	0.645	ns	0.5208
Q2			
Drd2$^{Cacna1h+}$ vs. Drd2$^{Cacna1h-}$	0.2558	ns	0.7988
Q3			
Drd2$^{Cacna1h+}$ vs. Drd2$^{Cacna1h-}$	0.8423	ns	0.4021
Q4			
Drd2$^{Cacna1h+}$ vs. Drd2$^{Cacna1h-}$	0.01279	ns	0.9898

Quadrant, Latency, PV group

ANOVA table (n=11 mice per group)	F (DFn, DFd)	P value
Interaction	F (3, 80) = 13.91	P<0.0001
Quadrant	F (3, 80) = 16.44	P<0.0001
PV group	F (1, 80) = 0.000	P>0.9999

Bonferroni's multiple comparisons test	t	Summary	Adjusted P Value
Q1			
PV^{Kcnn4+} vs. PV^{Kcnn4-}	1.675	ns	0.0978
Q2			
PV^{Kcnn4+} vs. PV^{Kcnn4-}	5.591	****	<0.0001
Q3			
PV^{Kcnn4+} vs. PV^{Kcnn4-}	1.955	ns	0.0541
Q4			
PV^{Kcnn4+} vs. PV^{Kcnn4-}	1.961	ns	0.0534

Quadrant, Latency, Drd2 group

ANOVA table (n=11 mice per group)	F (DFn, DFd)	P value
Interaction	F (3, 80) = 0.03660	P=0.9906
Quadrant	F (3, 80) = 34.42	P<0.0001
Drd2 group	F (1, 80) = 0.000	P>0.9999

Bonferroni's multiple comparisons test	t	Summary	Adjusted P Value
Q1			
Drd2$^{Cacna1h+}$ vs. Drd2$^{Cacna1h-}$	0.2019	ns	0.8405
Q2			
Drd2$^{Cacna1h+}$ vs. Drd2$^{Cacna1h-}$	0.2522	ns	0.8015
Q3			
Drd2$^{Cacna1h+}$ vs. Drd2$^{Cacna1h-}$	0.07096	ns	0.9436
Q4			
Drd2$^{Cacna1h+}$ vs. Drd2$^{Cacna1h-}$	0.02062	ns	0.9836

Related to Figure S5F (Ordinary two-way ANOVA)
Annulus, Length, PV group

ANOVA table (n=11 mice per group)	F (DFn, DFd)	P value
Interaction	F (2, 60) = 4.713	P=0.0126
Annulus	F (2, 60) = 45.90	P<0.0001
PV group	F (1, 60) = 0.2939	P=0.5897

Bonferroni's multiple comparisons test	t	Summary	Adjusted P Value
Inner			
PV^{Kcnn4+} vs. PV^{Kcnn4-}	1.165	ns	0.2484
Middle			
PV^{Kcnn4+} vs. PV^{Kcnn4-}	2.155	*	0.0352
Outer			

PV^{Kcnn4+} vs. PV^{Kcnn4-}	1.928	ns	0.0586

Annulus, Length, Drd2 group

ANOVA table (n=11 mice per group)	F (DFn, DFd)	P value
Interaction	F (2, 60) = 0.07363	P=0.9291
Annulus	F (2, 60) = 39.85	P<0.0001
Drd2 group	F (1, 60) = 0.4565	P=0.5019

Bonferroni's multiple comparisons test	t	Summary	Adjusted P Value
Inner			
$Drd2^{Cacna1h+}$ vs. $Drd2^{Cacna1h-}$	0.199	ns	0.843
Middle			
$Drd2^{Cacna1h+}$ vs. $Drd2^{Cacna1h-}$	0.7007	ns	0.4862
Outer			
$Drd2^{Cacna1h+}$ vs. $Drd2^{Cacna1h-}$	0.2706	ns	0.7876

Annulus, Latency, PV group

ANOVA table (n=11 mice per group)	F (DFn, DFd)	P value
Interaction	F (2, 60) = 13.79	P<0.0001
Annulus	F (2, 60) = 121.7	P<0.0001
PV group	F (1, 60) = 4.653e-007	P=0.9995

Bonferroni's multiple comparisons test	t	Summary	Adjusted P Value
Inner			
PV^{Kcnn4+} vs. PV^{Kcnn4-}	2.483	*	0.0158
Middle			
PV^{Kcnn4+} vs. PV^{Kcnn4-}	4.268	****	<0.0001
Outer			
PV^{Kcnn4+} vs. PV^{Kcnn4-}	1.786	ns	0.0792

Annulus, Latency, Drd2 group

ANOVA table (n=11 mice per group)	F (DFn, DFd)	P value
Interaction	F (2, 60) = 0.006089	P=0.9939
Annulus	F (2, 60) = 59.59	P<0.0001
Drd2 group	F (1, 60) = 2.143e-008	P=0.9999

Bonferroni's multiple comparisons test	t	Summary	Adjusted P Value
Inner			
$Drd2^{Cacna1h+}$ vs. $Drd2^{Cacna1h-}$	0.05629	ns	0.9553
Middle			
$Drd2^{Cacna1h+}$ vs. $Drd2^{Cacna1h-}$	0.03296	ns	0.9738
Outer			
$Drd2^{Cacna1h+}$ vs. $Drd2^{Cacna1h-}$	0.089	ns	0.9294

Related to Figure S6B (Ordinary one-way ANOVA)

PRM

ANOVA table (n=11 mice per group)	F (DFn, DFd)	P value
Treatment	F (6, 70) = 9.633	P<0.0001

Bonferroni's multiple comparisons test	t	Summary	Adjusted P Value
Con vs. 20	5.924	****	<0.0001
Con vs. 40	5.338	****	<0.0001
Con vs. 60	4.731	****	<0.0001
Con vs. 90	3.385	**	0.007
Con vs. 120	2.756	*	0.0447
Con vs. 150	1.15	ns	>0.9999

ORM

ANOVA table (n=11 mice per group)	F (DFn, DFd)	P value
Treatment	F (6, 70) = 11.61	P<0.0001

Bonferroni's multiple comparisons test	t	Summary	Adjusted P Value
Con vs. 12	6.745	****	<0.0001
Con vs. 18	6.77	****	<0.0001
Con vs. 24	5.165	****	<0.0001
Con vs. 36	3.435	**	0.006
Con vs. 48	2.984	*	0.0235
Con vs. 60	3.034	*	0.0203

Related to Figure S6C (unpaired t-test)

PV^{Kcnn4+} group **PV^{Kcnn4-} group**

Unpaired t test (n=11 mice per group)

P value	0.7807
P value summary	ns
Significantly different (P < 0.05)?	No
One- or two-tailed P value?	Two-tailed
t, df	t=0.2822, df=20

Unpaired t test (n=11 mice per group)

P value	0.9419
P value summary	ns
Significantly different (P < 0.05)?	No
One- or two-tailed P value?	Two-tailed
t, df	t=0.07377, df=20

Drd2$^{Cacna1h+}$ group

Unpaired t test (n=11 mice per group)

P value	0.611
P value summary	ns
Significantly different (P < 0.05)?	No
One- or two-tailed P value?	Two-tailed
t, df	t=0.5168, df=20

Drd2$^{Cacna1h-}$ group

Unpaired t test (n=11 mice per group)

P value	0.493
P value summary	ns
Significantly different (P < 0.05)?	No
One- or two-tailed P value?	Two-tailed
t, df	t=0.6984, df=20

Related to Figure S6D (unpaired t-test)

PV^{Kcnn4+} group

Unpaired t test (n=13 mice per group)

P value	0.9732
P value summary	ns
Significantly different (P < 0.05)?	No
One- or two-tailed P value?	Two-tailed
t, df	t=0.03396, df=24

PV^{Kcnn4-} group

Unpaired t test (n=13 mice per group)

P value	0.4935
P value summary	ns
Significantly different (P < 0.05)?	No
One- or two-tailed P value?	Two-tailed
t, df	t=0.6954, df=24

Drd2$^{Cacna1h+}$ group

Unpaired t test (n=13 mice per group)

P value	0.636
P value summary	ns
Significantly different (P < 0.05)?	No
One- or two-tailed P value?	Two-tailed
t, df	t=0.4794, df=24

Drd2$^{Cacna1h-}$ group

Unpaired t test (n=13 mice per group)

P value	0.4804
P value summary	ns
Significantly different (P < 0.05)?	No
One- or two-tailed P value?	Two-tailed
t, df	t=0.7169, df=24

Related to Figure S6F (unpaired t-test)

PRM, PV^{Kcnn4+} group

Unpaired t test (n=11 mice per group)

P value	<0.0001
P value summary	****
Significantly different (P < 0.05)?	Yes
One- or two-tailed P value?	Two-tailed
t, df	t=10.40, df=20

PRM, PV^{Kcnn4-} group

Unpaired t test (n=11 mice per group)

P value	<0.0001
P value summary	****
Significantly different (P < 0.05)?	Yes
One- or two-tailed P value?	Two-tailed
t, df	t=11.17, df=20

PRM, Drd2$^{Cacna1h+}$ group

Unpaired t test (n=11 mice per group)

P value	<0.0001
P value summary	****
Significantly different (P < 0.05)?	Yes
One- or two-tailed P value?	Two-tailed
t, df	t=10.68, df=20

PRM, Drd2$^{Cacna1h-}$ group

Unpaired t test (n=11 mice per group)

P value	<0.0001
P value summary	****
Significantly different (P < 0.05)?	Yes
One- or two-tailed P value?	Two-tailed
t, df	t=10.03, df=20

ORM, PV^{Kcnn4+} group

Unpaired t test (n=11 mice per group)

P value	<0.0001
P value summary	****
Significantly different (P < 0.05)?	Yes
One- or two-tailed P value?	Two-tailed
t, df	t=10.04, df=20

ORM, PV^{Kcnn4-} group

Unpaired t test (n=11 mice per group)

P value	<0.0001
P value summary	****
Significantly different (P < 0.05)?	Yes
One- or two-tailed P value?	Two-tailed
t, df	t=11.64, df=20

ORM, Drd2$^{Cacna1h+}$ group

Unpaired t test (n=11 mice per group)

P value	<0.0001
P value summary	****
Significantly different (P < 0.05)?	Yes
One- or two-tailed P value?	Two-tailed
t, df	t=10.69, df=20

ORM, Drd2$^{Cacna1h-}$ group

Unpaired t test (n=11 mice per group)

P value	<0.0001
P value summary	****
Significantly different (P < 0.05)?	Yes
One- or two-tailed P value?	Two-tailed
t, df	t=11.72, df=20

Related to Figure S6G (unpaired t-test)

PRM, PV group

Unpaired t test (n=11 mice per group)

P value	0.8708
P value summary	ns

PRM, Drd2 group

Unpaired t test (n=11 mice per group)

P value	0.8065
P value summary	ns

Significantly different (P < 0.05)?	No
One- or two-tailed P value?	Two-tailed
t, df	t=0.1647, df=20

Significantly different (P < 0.05)?	No
One- or two-tailed P value?	Two-tailed
t, df	t=0.2483, df=20

ORM, PV group

Unpaired t test (n=11 mice per group)

P value	0.5673
P value summary	ns
Significantly different (P < 0.05)?	No
One- or two-tailed P value?	Two-tailed
t, df	t=0.5817, df=20

ORM, Drd2 group

Unpaired t test (n=11 mice per group)

P value	0.9464
P value summary	ns
Significantly different (P < 0.05)?	No
One- or two-tailed P value?	Two-tailed
t, df	t=0.06806, df=20

Related to Figure S9A (unpaired t-test)

DS group

Unpaired t test (n=15 neurons per group)

P value	<0.0001
P value summary	****
Significantly different (P < 0.05)?	Yes
One- or two-tailed P value?	Two-tailed
t, df	t=14.41, df=28

VS group

Unpaired t test (n=15 neurons per group)

P value	<0.0001
P value summary	****
Significantly different (P < 0.05)?	Yes
One- or two-tailed P value?	Two-tailed
t, df	t=8.418, df=28

Related to Figure S9B (unpaired t-test)

EPSC, Frequency, DS group

Unpaired t test (n=12 neurons per group)

P value	<0.0001
P value summary	****
Significantly different (P < 0.05)?	Yes
One- or two-tailed P value?	Two-tailed
t, df	t=8.784, df=22

EPSC, Frequency, VS group

Unpaired t test (n=12 neurons per group)

P value	<0.0001
P value summary	****
Significantly different (P < 0.05)?	Yes
One- or two-tailed P value?	Two-tailed
t, df	t=7.388, df=22

EPSC, Amplitude, DS group

Unpaired t test (n=12 neurons per group)

P value	0.9547
P value summary	ns
Significantly different (P < 0.05)?	No
One- or two-tailed P value?	Two-tailed
t, df	t=0.05749, df=22

EPSC, Amplitude, VS group

Unpaired t test (n=12 neurons per group)

P value	0.8554
P value summary	ns
Significantly different (P < 0.05)?	No
One- or two-tailed P value?	Two-tailed
t, df	t=0.1844, df=22

Related to Figure S9C (unpaired t-test)

Training, PRM, DS^{GFP}-CNO group

Unpaired t test (n=11 mice per group)

P value	0.7746
P value summary	ns
Significantly different (P < 0.05)?	No
One- or two-tailed P value?	Two-tailed
t, df	t=0.2902, df=20

Training, PRM, DS^{GFP}-Saline group

Unpaired t test (n=11 mice per group)

P value	0.5773
P value summary	ns
Significantly different (P < 0.05)?	No
One- or two-tailed P value?	Two-tailed
t, df	t=0.5666, df=20

Training, PRM, DS^{hM4Di}-CNO group

Unpaired t test (n=11 mice per group)

P value	0.6801
P value summary	ns
Significantly different (P < 0.05)?	No
One- or two-tailed P value?	Two-tailed
t, df	t=0.4185, df=20

Training, PRM, DS^{hM4Di}-Saline group

Unpaired t test (n=11 mice per group)

P value	0.9012
P value summary	ns
Significantly different (P < 0.05)?	No
One- or two-tailed P value?	Two-tailed
t, df	t=0.1257, df=20

Training, PRM, VS^{tdT}-CNO group

Unpaired t test (n=11 mice per group)

P value	0.6166
P value summary	ns
Significantly different (P < 0.05)?	No
One- or two-tailed P value?	Two-tailed
t, df	t=0.5086, df=20

Training, PRM, VS^{tdT}-Saline group

Unpaired t test (n=11 mice per group)

P value	0.6833
P value summary	ns
Significantly different (P < 0.05)?	No
One- or two-tailed P value?	Two-tailed
t, df	t=0.4139, df=20

Training, PRM, VS^{hM4Di}-CNO group

Unpaired t test (n=11 mice per group)

P value	0.6561
P value summary	ns
Significantly different (P < 0.05)?	No
One- or two-tailed P value?	Two-tailed
t, df	t=0.4521, df=20

Training, PRM, VS^{hM4Di}-Saline group

Unpaired t test (n=11 mice per group)

P value	0.9077
P value summary	ns
Significantly different (P < 0.05)?	No
One- or two-tailed P value?	Two-tailed
t, df	t=0.1174, df=20

Training, ORM, DS^{GFP}-CNO group

Unpaired t test (n=11 mice per group)	
P value	0.7091
P value summary	ns
Significantly different (P < 0.05)?	No
One- or two-tailed P value?	Two-tailed
t, df	t=0.3784, df=20

Training, ORM, VS^{tdT}-CNO group

Unpaired t test (n=11 mice per group)	
P value	0.4047
P value summary	ns
Significantly different (P < 0.05)?	No
One- or two-tailed P value?	Two-tailed
t, df	t=0.8513, df=20

Training, ORM, VS^{tdT}-CNO group

Unpaired t test (n=11 mice per group)	
P value	0.7415
P value summary	ns
Significantly different (P < 0.05)?	No
One- or two-tailed P value?	Two-tailed
t, df	t=0.3345, df=20

Training, ORM, VS^{hM4Di}-CNO group

Unpaired t test (n=11 mice per group)	
P value	0.9835
P value summary	ns
Significantly different (P < 0.05)?	No
One- or two-tailed P value?	Two-tailed
t, df	t=0.02096, df=20

Related to Figure S9D (unpaired t-test)

Testing, PRM, DS^{GFP}-CNO group

Unpaired t test (n=11 mice per group)	
P value	<0.0001
P value summary	****
Significantly different (P < 0.05)?	Yes
One- or two-tailed P value?	Two-tailed
t, df	t=7.046, df=20

Testing, PRM, DS^{hM4Di}-CNO group

Unpaired t test (n=11 mice per group)	
P value	0.0128
P value summary	*
Significantly different (P < 0.05)?	Yes
One- or two-tailed P value?	Two-tailed
t, df	t=2.734, df=20

Testing, PRM, VS^{tdT}-CNO group

Unpaired t test (n=11 mice per group)	
P value	<0.0001
P value summary	****
Significantly different (P < 0.05)?	Yes
One- or two-tailed P value?	Two-tailed
t, df	t=7.535, df=20

Testing, PRM, VS^{hM4Di}-CNO group

Unpaired t test (n=11 mice per group)	
P value	<0.0001
P value summary	****
Significantly different (P < 0.05)?	Yes
One- or two-tailed P value?	Two-tailed
t, df	t=7.421, df=20

Testing, ORM, DS^{GFP}-CNO group

Unpaired t test (n=11 mice per group)	
P value	<0.0001
P value summary	****
Significantly different (P < 0.05)?	Yes
One- or two-tailed P value?	Two-tailed

Training, ORM, DS^{GFP}-Saline group

Unpaired t test (n=11 mice per group)	
P value	0.3958
P value summary	ns
Significantly different (P < 0.05)?	No
One- or two-tailed P value?	Two-tailed
t, df	t=0.8677, df=20

Training, ORM, VS^{tdT}-Saline group

Unpaired t test (n=11 mice per group)	
P value	0.8765
P value summary	ns
Significantly different (P < 0.05)?	No
One- or two-tailed P value?	Two-tailed
t, df	t=0.1573, df=20

Training, ORM, VS^{tdT}-Saline group

Unpaired t test (n=11 mice per group)	
P value	0.9453
P value summary	ns
Significantly different (P < 0.05)?	No
One- or two-tailed P value?	Two-tailed
t, df	t=0.06944, df=20

Training, ORM, VS^{hM4Di}-Saline group

Unpaired t test (n=11 mice per group)	
P value	0.9425
P value summary	ns
Significantly different (P < 0.05)?	No
One- or two-tailed P value?	Two-tailed
t, df	t=0.07299, df=20

Testing, PRM, DS^{GFP}-Saline group

Unpaired t test (n=11 mice per group)	
P value	<0.0001
P value summary	****
Significantly different (P < 0.05)?	Yes
One- or two-tailed P value?	Two-tailed
t, df	t=10.31, df=20

Testing, PRM, DS^{hM4Di}-Saline group

Unpaired t test (n=11 mice per group)	
P value	<0.0001
P value summary	****
Significantly different (P < 0.05)?	Yes
One- or two-tailed P value?	Two-tailed
t, df	t=10.07, df=20

Testing, PRM, VS^{tdT}-Saline group

Unpaired t test (n=11 mice per group)	
P value	<0.0001
P value summary	****
Significantly different (P < 0.05)?	Yes
One- or two-tailed P value?	Two-tailed
t, df	t=10.62, df=20

Testing, PRM, VS^{hM4Di}-Saline group

Unpaired t test (n=11 mice per group)	
P value	<0.0001
P value summary	****
Significantly different (P < 0.05)?	Yes
One- or two-tailed P value?	Two-tailed
t, df	t=6.614, df=20

Testing, ORM, DS^{GFP}-Saline group

Unpaired t test (n=11 mice per group)	
P value	<0.0001
P value summary	****
Significantly different (P < 0.05)?	Yes
One- or two-tailed P value?	Two-tailed

t, df t=9.738, df=20

Testing, ORM, DS^{hM4Di}-CNO group
Unpaired t test (n=11 mice per group)
P value <0.0001
P value summary ****
Significantly different (P < 0.05)? Yes
One- or two-tailed P value? Two-tailed
t, df t=8.882, df=20

Testing, ORM, VS^{tdT}-CNO group
Unpaired t test (n=11 mice per group)
P value <0.0001
P value summary ****
Significantly different (P < 0.05)? Yes
One- or two-tailed P value? Two-tailed
t, df t=10.86, df=20

Testing, ORM, VS^{hM4Di}-CNO group
Unpaired t test (n=11 mice per group)
P value 0.062
P value summary ns
Significantly different (P < 0.05)? No
One- or two-tailed P value? Two-tailed
t, df t=1.977, df=20

Related to Figure S10C (paried t-test)
AVT
Paired t test (n = 5 mice per group)
P value <0.0001
P value summary ****
Significantly different (P < 0.05)? Yes
One- or two-tailed P value? Two-tailed
t, df t=26.30, df=4
Number of pairs 5

Related to Figure S10D (paried t-test)
AVT
Paired t test (n = 5 mice per group)
P value 0.0972
P value summary ns
Significantly different (P < 0.05)? No
One- or two-tailed P value? Two-tailed
t, df t=2.157, df=4
Number of pairs 5

Related to Figure S10F (unparied t-test)
Training, PRM, DS^{hM4Di}-PV^{tdT}
Unpaired t test (n=11 mice per group)
P value 0.8063
P value summary ns
Significantly different (P < 0.05)? No
One- or two-tailed P value? Two-tailed
t, df t=0.2485, df=20

Training, PRM, VS^{hM4Di}-Drd2^{GFP}
Unpaired t test (n=11 mice per group)
P value 0.8945
P value summary ns
Significantly different (P < 0.05)? No
One- or two-tailed P value? Two-tailed
t, df t=0.1344, df=20

Training, ORM, DS^{hM4Di}-PV^{tdT}
Unpaired t test (n=11 mice per group)
P value 0.8492
P value summary ns
Significantly different (P < 0.05)? No
One- or two-tailed P value? Two-tailed
t, df t=0.1926, df=20

t, df t=9.106, df=20

Testing, ORM, DS^{hM4Di}-Saline group
Unpaired t test (n=11 mice per group)
P value <0.0001
P value summary ****
Significantly different (P < 0.05)? Yes
One- or two-tailed P value? Two-tailed
t, df t=8.553, df=20

Testing, ORM, VS^{tdT}-Saline group
Unpaired t test (n=11 mice per group)
P value <0.0001
P value summary ****
Significantly different (P < 0.05)? Yes
One- or two-tailed P value? Two-tailed
t, df t=7.548, df=20

Testing, ORM, VS^{hM4Di}-Saline group
Unpaired t test (n=11 mice per group)
P value <0.0001
P value summary ****
Significantly different (P < 0.05)? Yes
One- or two-tailed P value? Two-tailed
t, df t=9.534, df=20

AMT
Paired t test (n = 5 mice per group)
P value 0.0512
P value summary ns
Significantly different (P < 0.05)? No
One- or two-tailed P value? Two-tailed
t, df t=2.753, df=4
Number of pairs 5

AMT
paired t test (n = 5 mice per group)
P value 0.0007
P value summary ***
Significantly different (P < 0.05)? Yes
One- or two-tailed P value? Two-tailed
t, df t=9.579, df=4
Number of pairs 5

Training, PRM, DS^{hM4Di}-PV^{ChR2}
Unpaired t test (n=11 mice per group)
P value 0.9116
P value summary ns
Significantly different (P < 0.05)? No
One- or two-tailed P value? Two-tailed
t, df t=0.1124, df=20

Training, PRM, VS^{hM4Di}-Drd2^{ChR2}
Unpaired t test (n=11 mice per group)
P value 0.5556
P value summary ns
Significantly different (P < 0.05)? No
One- or two-tailed P value? Two-tailed
t, df t=0.5994, df=20

Training, ORM, DS^{hM4Di}-PV^{ChR2}
Unpaired t test (n=11 mice per group)
P value 0.8687
P value summary ns
Significantly different (P < 0.05)? No
One- or two-tailed P value? Two-tailed
t, df t=0.1674, df=20

Training, ORM, VS^{hM4Di}-$Drd2^{GFP}$

Unpaired t test (n=11 mice per group)

P value	0.4344
P value summary	ns
Significantly different (P < 0.05)?	No
One- or two-tailed P value?	Two-tailed
t, df	t=0.7977, df=20

Training, ORM, VS^{hM4Di}-$Drd2^{ChR2}$

Unpaired t test (n=11 mice per group)

P value	0.6764
P value summary	ns
Significantly different (P < 0.05)?	No
One- or two-tailed P value?	Two-tailed
t, df	t=0.4236, df=20

Testing, PRM, DS^{hM4Di}-PV^{tdT}

Unpaired t test (n=11 mice per group)

P value	0.4539
P value summary	ns
Significantly different (P < 0.05)?	No
One- or two-tailed P value?	Two-tailed
t, df	t=0.7638, df=20

Testing, PRM, DS^{hM4Di}-PV^{ChR2}

Unpaired t test (n=11 mice per group)

P value	0.7628
P value summary	ns
Significantly different (P < 0.05)?	No
One- or two-tailed P value?	Two-tailed
t, df	t=0.3059, df=20

Testing, PRM, VS^{hM4Di}-$Drd2^{GFP}$

Unpaired t test (n=11 mice per group)

P value	<0.0001
P value summary	****
Significantly different (P < 0.05)?	Yes
One- or two-tailed P value?	Two-tailed
t, df	t=8.863, df=20

Testing, PRM, VS^{hM4Di}-$Drd2^{ChR2}$

Unpaired t test (n=11 mice per group)

P value	<0.0001
P value summary	****
Significantly different (P < 0.05)?	Yes
One- or two-tailed P value?	Two-tailed
t, df	t=10.07, df=20

Testing, ORM, DS^{hM4Di}-PV^{tdT}

Unpaired t test (n=11 mice per group)

P value	<0.0001
P value summary	****
Significantly different (P < 0.05)?	Yes
One- or two-tailed P value?	Two-tailed
t, df	t=10.51, df=20

Testing, ORM, DS^{hM4Di}-PV^{ChR2}

Unpaired t test (n=11 mice per group)

P value	<0.0001
P value summary	****
Significantly different (P < 0.05)?	Yes
One- or two-tailed P value?	Two-tailed
t, df	t=10.12, df=20

Testing, ORM, VS^{hM4Di}-$Drd2^{GFP}$

Unpaired t test (n=11 mice per group)

P value	0.7328
P value summary	ns
Significantly different (P < 0.05)?	No
One- or two-tailed P value?	Two-tailed
t, df	t=0.3462, df=20

Testing, ORM, VS^{hM4Di}-$Drd2^{ChR2}$

Unpaired t test (n=11 mice per group)

P value	0.1604
P value summary	ns
Significantly different (P < 0.05)?	No
One- or two-tailed P value?	Two-tailed
t, df	t=1.458, df=20

Related to Figure S10H (unpaird t-test）

Testing, PRM, DS^{hM4Di}-PV^{tdT}

Unpaired t test (n=11 mice per group)

P value	0.9832
P value summary	ns
Significantly different (P < 0.05)?	No
One- or two-tailed P value?	Two-tailed
t, df	t=0.02131, df=20

Testing, PRM, DS^{hM4Di}-PV^{ChR2}

Unpaired t test (n=11 mice per group)

P value	<0.0001
P value summary	****
Significantly different (P < 0.05)?	Yes
One- or two-tailed P value?	Two-tailed
t, df	t=6.994, df=20

Testing, PRM, VS^{hM4Di}-$Drd2^{GFP}$

Unpaired t test (n=11 mice per group)

P value	<0.0001
P value summary	****
Significantly different (P < 0.05)?	Yes
One- or two-tailed P value?	Two-tailed
t, df	t=7.979, df=20

Testing, PRM, VS^{hM4Di}-$Drd2^{ChR2}$

Unpaired t test (n=11 mice per group)

P value	<0.0001
P value summary	****
Significantly different (P < 0.05)?	Yes
One- or two-tailed P value?	Two-tailed
t, df	t=8.968, df=20

Testing, ORM, DS^{hM4Di}-PV^{tdT}

Unpaired t test (n=11 mice per group)

P value	<0.0001
P value summary	****
Significantly different (P < 0.05)?	Yes
One- or two-tailed P value?	Two-tailed
t, df	t=11.91, df=20

Testing, ORM, DS^{hM4Di}-PV^{ChR2}

Unpaired t test (n=11 mice per group)

P value	<0.0001
P value summary	****
Significantly different (P < 0.05)?	Yes
One- or two-tailed P value?	Two-tailed
t, df	t=8.462, df=20

Testing, ORM, VS^{hM4Di}-$Drd2^{GFP}$

Unpaired t test (n=11 mice per group)

P value	0.5781
P value summary	ns
Significantly different (P < 0.05)?	No
One- or two-tailed P value?	Two-tailed

Testing, ORM, VS^{hM4Di}-$Drd2^{ChR2}$

Unpaired t test (n=11 mice per group)

P value	<0.0001
P value summary	****
Significantly different (P < 0.05)?	Yes
One- or two-tailed P value?	Two-tailed

t, df	t=0.5655, df=20

t, df	t=8.571, df=20

Related to Figure S11B (unparied t-test)

AVT

Unpaired t test (n=15 neurons per group)	
P value	<0.0001
P value summary	****
Significantly different (P < 0.05)?	Yes
One- or two-tailed P value?	Two-tailed
t, df	t=6.214, df=28

AMT

Unpaired t test (n=15 neurons per group)	
P value	<0.0001
P value summary	****
Significantly different (P < 0.05)?	Yes
One- or two-tailed P value?	Two-tailed
t, df	t=6.925, df=28

Related to Figure S11C (Ordinary one-way ANOVA)

ANOVA table (n=15 neurons per group)	F (DFn, DFd)	P value
Treatment	F (3, 56) = 134.2	P<0.0001

Bonferroni's multiple comparisons test	t	Summary	Adjusted P Value
Control vs. TTX	14.76	****	<0.0001
Control vs. TTX+4-AP	0.5855	ns	>0.9999
Control vs. TTX+4-AP+CNQX	14.18	****	<0.0001
TTX vs. TTX+4-AP	14.17	****	<0.0001
TTX vs. TTX+4-AP+CNQX	0.5837	ns	>0.9999
TTX+4-AP vs. TTX+4-AP+CNQX	13.59	****	<0.0001

Related to Figure S11D (Ordinary one-way ANOVA)

ANOVA table (n=15 neurons per group)	F (DFn, DFd)	P value
Treatment	F (3, 56) = 159.6	P<0.0001

Bonferroni's multiple comparisons test	t	Summary	Adjusted P Value
Control vs. TTX	14.56	****	<0.0001
Control vs. TTX+4-AP	1.79	ns	0.4735
Control vs. TTX+4-AP+CNQX	14.49	****	<0.0001
TTX vs. TTX+4-AP	16.35	****	<0.0001
TTX vs. TTX+4-AP+CNQX	0.07627	ns	>0.9999
TTX+4-AP vs. TTX+4-AP+CNQX	16.28	****	<0.0001

Related to Figure S11G (unparied t-test)

PRM, PV^{tdT}-AVT^{GFP}

Unpaired t test (n=11 mice per group)	
P value	<0.0001
P value summary	****
Significantly different (P < 0.05)?	Yes
One- or two-tailed P value?	Two-tailed
t, df	t=5.638, df=20

PRM, PV^{tdT}-AVT^{hM4Di}

Unpaired t test (n=11 mice per group)	
P value	0.5925
P value summary	ns
Significantly different (P < 0.05)?	No
One- or two-tailed P value?	Two-tailed
t, df	t=0.5440, df=20

PRM, PV^{tChR2}-AVT^{GFP}

Unpaired t test (n=11 mice per group)	
P value	<0.0001
P value summary	****
Significantly different (P < 0.05)?	Yes
One- or two-tailed P value?	Two-tailed
t, df	t=14.96, df=20

PRM, PV^{ChR2}-AVT^{hM4Di}

Unpaired t test (n=11 mice per group)	
P value	0.941
P value summary	ns
Significantly different (P < 0.05)?	No
One- or two-tailed P value?	Two-tailed
t, df	t=0.07498, df=20

PRM, Drd2^{GFP}-AMT^{tdT}

Unpaired t test (n=11 mice per group)	
P value	<0.0001
P value summary	****
Significantly different (P < 0.05)?	Yes
One- or two-tailed P value?	Two-tailed
t, df	t=8.379, df=20

PRM, Drd2^{GFP}-AMT^{hM4Di}

Unpaired t test (n=11 mice per group)	
P value	<0.0001
P value summary	****
Significantly different (P < 0.05)?	Yes
One- or two-tailed P value?	Two-tailed
t, df	t=5.076, df=20

PRM, Drd2^{ChR2}-AMT^{tdT}

Unpaired t test (n=11 mice per group)	
P value	<0.0001
P value summary	****
Significantly different (P < 0.05)?	Yes
One- or two-tailed P value?	Two-tailed
t, df	t=7.548, df=20

PRM, Drd2^{ChR2}-AMT^{hM4Di}

Unpaired t test (n=11 mice per group)	
P value	<0.0001
P value summary	****
Significantly different (P < 0.05)?	Yes
One- or two-tailed P value?	Two-tailed
t, df	t=8.067, df=20

ORM, PV^{tdT}-AVT^{GFP}

Unpaired t test (n=11 mice per group)	
P value	<0.0001

ORM, PV^{tdT}-AVT^{hM4Di}

Unpaired t test (n=11 mice per group)	
P value	<0.0001

P value summary	****
Significantly different (P < 0.05)?	Yes
One- or two-tailed P value?	Two-tailed
t, df	t=7.440, df=20

P value summary	****
Significantly different (P < 0.05)?	Yes
One- or two-tailed P value?	Two-tailed
t, df	t=6.483, df=20

ORM, PV^{tChR2}-AVT^{GFP}

Unpaired t test (n=11 mice per group)

P value	<0.0001
P value summary	****
Significantly different (P < 0.05)?	Yes
One- or two-tailed P value?	Two-tailed
t, df	t=8.413, df=20

ORM, PV^{ChR2}-AVT^{hM4Di}

Unpaired t test (n=11 mice per group)

P value	<0.0001
P value summary	****
Significantly different (P < 0.05)?	Yes
One- or two-tailed P value?	Two-tailed
t, df	t=5.898, df=20

ORM, $Drd2^{GFP}$-AMT^{tdT}

Unpaired t test (n=11 mice per group)

P value	<0.0001
P value summary	****
Significantly different (P < 0.05)?	Yes
One- or two-tailed P value?	Two-tailed
t, df	t=7.830, df=20

ORM, $Drd2^{GFP}$-AMT^{hM4Di}

Unpaired t test (n=11 mice per group)

P value	0.8431
P value summary	ns
Significantly different (P < 0.05)?	No
One- or two-tailed P value?	Two-tailed
t, df	t=0.2005, df=20

ORM, $Drd2^{ChR2}$-AMT^{tdT}

Unpaired t test (n=11 mice per group)

P value	<0.0001
P value summary	****
Significantly different (P < 0.05)?	Yes
One- or two-tailed P value?	Two-tailed
t, df	t=9.340, df=20

ORM, $Drd2^{ChR2}$-AMT^{hM4Di}

Unpaired t test (n=11 mice per group)

P value	0.6003
P value summary	ns
Significantly different (P < 0.05)?	No
One- or two-tailed P value?	Two-tailed
t, df	t=0.5324, df=20

Related to Figure S12B (Ordinary one-way ANOVA)

Peak

ANOVA table (n=11 mice per group)	F (DFn, DFd)	P value
Treatment	F (3, 40) = 1.611	P=0.2019

Bonferroni's multiple comparisons test	t	Summary	Adjusted P Value
PRM-A vs. PRM-A'	0.627	ns	>0.9999
PRM-A vs. ORM-A	1.243	ns	>0.9999
PRM-A vs. ORM-B	1.104	ns	>0.9999
PRM-A' vs. ORM-A	1.87	ns	0.4132
PRM-A' vs. ORM-B	1.731	ns	0.5472
ORM-A vs. ORM-B	0.1389	ns	>0.9999

AUC

ANOVA table (n=11 mice per group)	F (DFn, DFd)	P value
Treatment	F (3, 40) = 0.7402	P=0.5344

Bonferroni's multiple comparisons test	t	Summary	Adjusted P Value
PRM-A vs. PRM-A'	0.8276	ns	>0.9999
PRM-A vs. ORM-A	1.359	ns	>0.9999
PRM-A vs. ORM-B	1.208	ns	>0.9999
PRM-A' vs. ORM-A	0.5315	ns	>0.9999
PRM-A' vs. ORM-B	0.3804	ns	>0.9999
ORM-A vs. ORM-B	0.1512	ns	>0.9999

Related to Figure S12C (Ordinary one-way ANOVA)

Peak

ANOVA table (n=11 mice per group)	F (DFn, DFd)	P value
Treatment	F (3, 40) = 1.286	P=0.2923

Bonferroni's multiple comparisons test	t	Summary	Adjusted P Value
PRM-A vs. PRM-A'	0.9961	ns	>0.9999
PRM-A vs. ORM-A	0.2877	ns	>0.9999
PRM-A vs. ORM-B	0.9308	ns	>0.9999
PRM-A' vs. ORM-A	1.284	ns	>0.9999
PRM-A' vs. ORM-B	1.927	ns	0.3667
ORM-A vs. ORM-B	0.6431	ns	>0.9999

AUC

ANOVA table (n=11 mice per group)	F (DFn, DFd)	P value
Treatment	F (3, 40) = 0.2458	P=0.8638

Bonferroni's multiple comparisons test	t	Summary	Adjusted P Value
PRM-A vs. PRM-A'	0.5684	ns	>0.9999
PRM-A vs. ORM-A	0.003777	ns	>0.9999
PRM-A vs. ORM-B	0.6383	ns	>0.9999
PRM-A' vs. ORM-A	0.5722	ns	>0.9999
PRM-A' vs. ORM-B	0.06987	ns	>0.9999
ORM-A vs. ORM-B	0.6421	ns	>0.9999

Related to Figure S13A (unparied t-test)

**Peak**		**AUC**	
Unpaired t test (n=9 mice per group)		Unpaired t test (n=9 mice per group)	
P value	0.001	P value	<0.0001
P value summary	***	P value summary	****
Significantly different (P < 0.05)?	Yes	Significantly different (P < 0.05)?	Yes
One- or two-tailed P value?	Two-tailed	One- or two-tailed P value?	Two-tailed
t, df	t=4.028, df=16	t, df	t=5.821, df=16

Related to Figure S13B (unparied t-test)

**Peak**		**AUC**	
Unpaired t test (n=9 mice per group)		Unpaired t test (n=9 mice per group)	
P value	0.0006	P value	<0.0001
P value summary	***	P value summary	****
Significantly different (P < 0.05)?	Yes	Significantly different (P < 0.05)?	Yes
One- or two-tailed P value?	Two-tailed	One- or two-tailed P value?	Two-tailed
t, df	t=4.237, df=16	t, df	t=5.887, df=16
